# Synthesis and QSAR Studies of Claramine Derivatives, a New Class of Broad-Spectrum Antimicrobial Agents

**DOI:** 10.3390/molecules30234614

**Published:** 2025-12-01

**Authors:** Marine Blanchet, Carole Di Giorgio, Jean Michel Brunel

**Affiliations:** 1 Aix Marseille University, INSERM, SSA, MCT, 13385 Marseille, France; 2 Aix Marseille University, CNRS, IRD, IMBE UMR 7263, Laboratoire de Mutagénèse Environnementale, 13385 Marseille, France

**Keywords:** antibiotics, polyamine derivative, claramine series, antibacterial agents, bile acids

## Abstract

The development of new antibiotics has been recognized for over two decades as a major challenge in combating multidrug-resistant bacteria. Herein, we report the synthesis and QSAR studies of claramine derivatives, a new class of broad-spectrum antimicrobial agents active against both susceptible and resistant Gram-positive and Gram-negative strains. The observed antimicrobial activities were rationalized based on key topological parameters of the derivatives, while cytotoxicity was interpreted by correlating half maximal inhibitory concentration (IC_50_) values with QSAR models. Owing to the low cytotoxicity observed for several analogues, this molecular class represents a promising alternative for the development of novel agents to counteract multidrug resistance.

## 1. Introduction

Although antibiotic resistance is a natural phenomenon that arises when microorganisms are exposed to antimicrobial agents, the misuse and overuse of antibiotics have boosted the emergence of resistance phenotypes by bacteria [1,2]. Under this selective pressure, bacteria evolve mechanisms to survive and proliferate, leading to the spreading of multidrug-resistant strains that significantly compromise the effectiveness of antibiotic treatments [3,4,5]. Since 1987, only a few new antibiotic classes have been developed, and in response to this growing threat, the World Health Organization has highlighted a group of particularly dangerous pathogens known as the ESKAPEE pathogens (*Enterococcus faecium*, *Staphylococcus aureus*, *Klebsiella pneumoniae*, *Acinetobacter baumannii*, *Pseudomonas aeruginosa*, *Enterobacter* spp. and *Escherichia coli*) which constitute the main cause of nosocomial infections and for which there is an urgent medical need of effective antibiotics [6,7,8]. Remarkably, 9 of these 12 bacterial superbugs are Gram-negative species, including *Acinetobacter baumannii*, *Pseudomonas aeruginosa*, and *Enterobacterales* [9].

**Figure 1:**
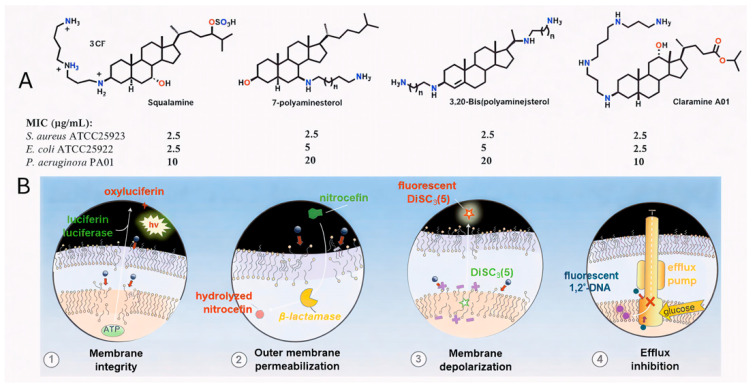
(**A**) Structure of squalamine, 7-polyaminesterol, 3,20-bispolyaminesterol derivatives and Claramine A01. (**B**) Claramine A01 exerts its bactericidal effect rapidly by disrupting membrane integrity (1 and 2) via membrane depolarization (3) and collapse of the proton gradient, which in turn strongly inhibits efflux pump activity (4).

To combat resistant bacteria, novel agents must overcome or circumvent intrinsic and acquire defense mechanisms such as biofilm formation, impermeability, efflux pumps, target mutations and degradative enzymes. A promising strategy in the search for next-generation antibiotics involves targeting the bacterial membrane—an essential and highly conserved structure across bacterial species that represents the first barrier to external aggression [10,11]. In 1993, squalamine, a water-soluble cationic steroid isolated from the dogfish shark (*Squalus acanthias*), was fully characterized and shown to possess potent antimicrobial activity against fungi, protozoa, and both Gram-positive and Gram-negative bacteria [12,13]. However, the synthetic route to this natural product requires approximately 15–20 steps, which limits its potential for large-scale production [14]. Over the years, many research teams [15,16,17,18], including our own group [19,20,21], have, developed significant expertise in the synthesis and biological evaluation of polyamine sterol derivatives, simplified analogues of squalamine accessible in only a few steps. (Figure 1A). In line with our ongoing efforts to extend the repertoire of antimicrobial polyamine derivatives, we recently investigated the role of molecular amphiphilicity in antibacterial properties of a series of polyamine derivatives, which ranged from weak to potent activity [22,23,24]. Interestingly, we showed that incorporation of polyamino groups onto a carefully designed lipophilic scaffold modulates antimicrobial activity, affording promising results against both Gram-positive and Gram-negative reference strains. Recently, Claramine A01 was synthesized from deoxycholic acid and shown to display broad-spectrum antibacterial activity against both sensitive and resistant strains. Claramine A01 also exhibits a versatile multifaceted mechanism of action (Figure 1B) [21].

**Figure 2:**
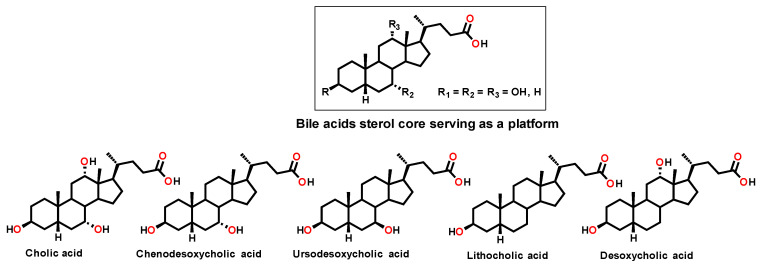
Structure of the different bile acids considered as a platform in this study.

Against Gram-positive bacteria, it acts rapidly through membrane depolarization, thereby compromising membrane integrity. In contrast, in Gram-negative bacteria, which possess both outer and inner membranes and are generally more difficult to eradicate, Claramine A01 induces alterations in transmembrane potential that lead to an increased membrane permeabilization and concomitant disruption of proton homeostasis. From these findings, we now report the development of a new class of antimicrobial agents based on a bile acid sterol core as a versatile platform (Figure 2), together with the rationalization and understanding of structure-activity relationships through the establishment of a QSAR model.

## 2. Results and Discussion

**Figure 3:**
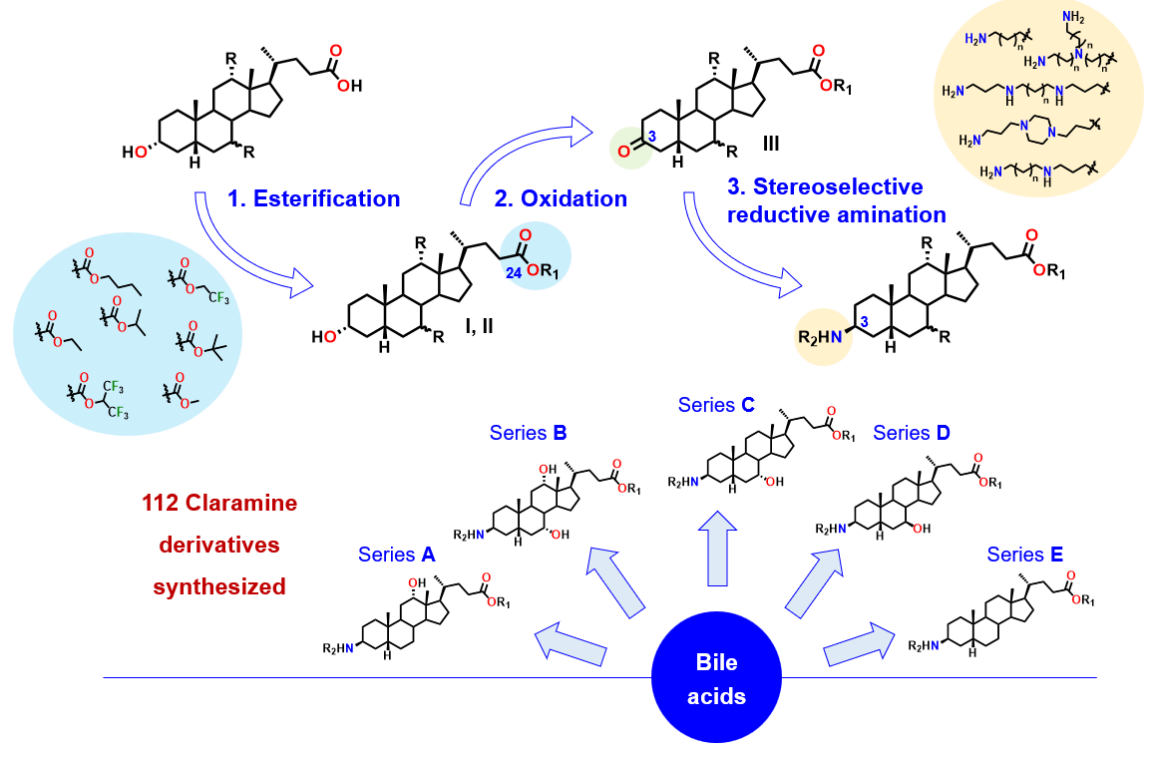
General synthetic route to Claramine analogues (series A–E).

In order to further investigate these antibacterial activities we were interested in designing a rational and economical synthesis of Claramine derivatives. For this purpose, we envisioned the preparation of a library of derivatives through a general three-step sequence starting from various bile acids, with an esterification, selective oxidation and a stereoselective titanium-mediated reductive amination controlling the β-stereochemistry at the C-3 position as the key synthetic step. (Figure 3).

**Figure 4:**
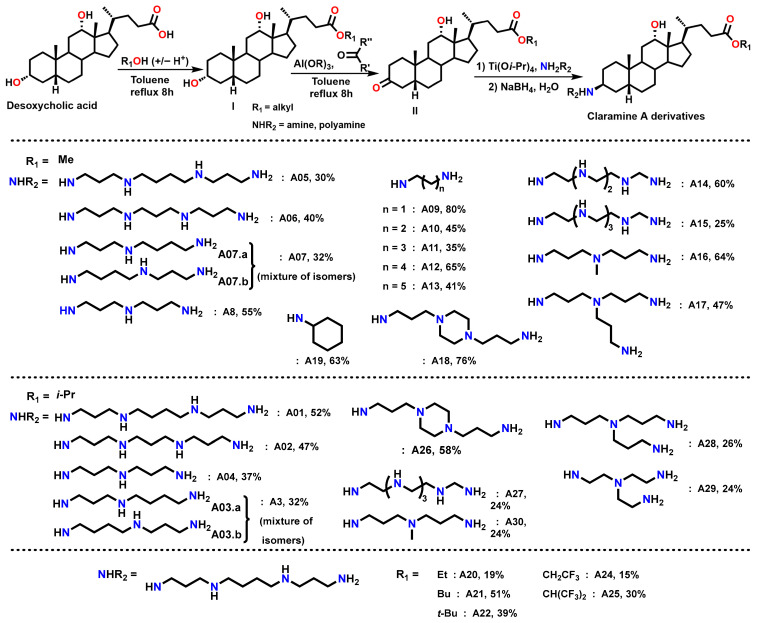
Synthetic route to Claramine derivatives (**A1**–**A30**).

Thus, a first series of 30 Claramine A derivatives was prepared (Figure 4), incorporating diverse β-C3 polyamine substituents and varied C-24 ester groups. These compounds were synthesized through a three-step sequence: (i) Fischer esterification to obtain the corresponding esters, (ii) Oppenauer oxidation of the C-3 hydroxyl group to afford ketosteroid intermediates in 25–80% yield (see Supplementary Data), and (iii) a stereoselective titanium-mediated reductive amination, developed in our laboratory, which introduced diverse amine substituents at C-3 to furnish the expected Claramine A derivatives (**A01**–**A30**) in isolated yields ranging from 19 to 80%. The series features a broad variety of C-3 polyamine moieties, including linear chains (e.g., spermine in **A01** and **A05**), cyclic motifs (e.g., 1,4-bis(3-aminopropyl)piperazine in **A18** and **A26**), and branched frameworks (e.g., tris(3-aminopropyl)amine in **A17** and **A28**).

**Figure 5:**
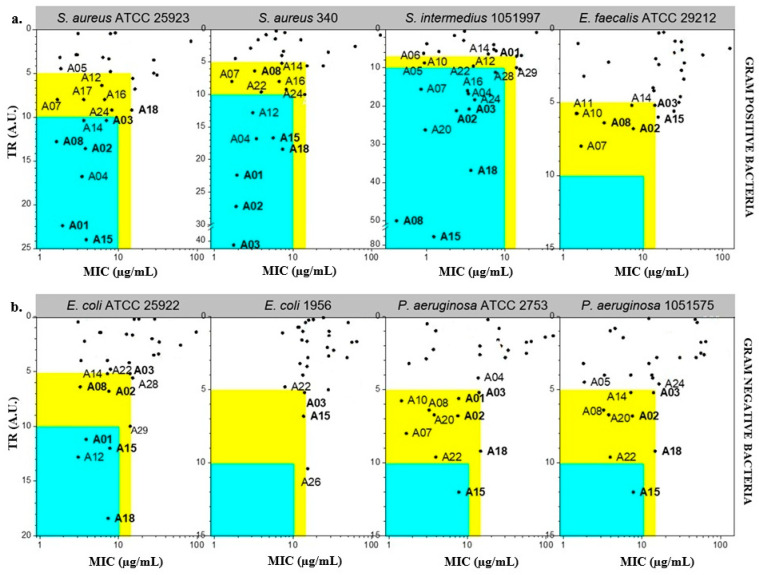
Antibacterial potential of Claramine A derivatives against (**a**) Gram-positive and (**b**) Gram-negative bacteria. In the blue zone, derivatives show strong antibacterial potential (TR > 10 and MIC < 10 µg/mL) against the indicated strain; in the yellow zone, derivatives display moderate potential (5 < TR < 10 and MIC < 15 µg/mL).

Claramine derivatives were evaluated for their antimicrobial activities against both Gram-positive and Gram-negative human and veterinary reference strains, including *S. aureus* ATCC 25923, *S. aureus* 340, *S. intermedius* 1051997, *E. faecalis* ATCC29212, *P. aeruginosa* ATCC 27853, *P. aeruginosa* 1051575, *E. coli* 1956 and *E. coli* ATCC 25922, and displayed moderate to excellent minimum inhibitory concentrations (MICs). Typically, cytotoxicity was expressed as the half maximal inhibitory concentration (IC_50_), defined as the concentration of a compound that decreases cell viability by 50% relative to untreated cells. This parameter provides a quantitative measure of a compound’s toxic potential toward mammalian cells. To facilitate data interpretation, we defined the Therapeutic Ratio (TR) as the ratio of IC_50_ (µg/mL) to MIC (µg/mL). This dimensionless parameter provides a straightforward measure of antibacterial selectivity, allowing direct comparison of cytotoxicity and antibacterial potency. A high TR value indicates compounds with strong antibacterial activity at concentrations far below their cytotoxic threshold, whereas a TR close to unity reflects poor selectivity and limited therapeutic potential. Specifically, compounds with TR > 10 and MIC < 10 µg/mL were classified as highly active, while those with 5 ≤ TR ≤ 10 and MIC < 15 µg/mL were considered moderately active. Within the Claramine A series, variations in IC_50_ strongly impacted TR values (Figure 5). Against Gram-positive strains, around 15 derivatives displayed high potential (TR > 10; MIC < 15 µg/mL vs. *S. aureus* and *S. intermedius*) (Figure 5a). Activity against *E. faecalis* was weaker, with the best compounds reaching TR values of 5–10 at MIC < 15 µg/mL. For Gram-negative bacteria, the most active analogues gave TR values of 5–10, with only a limited number reaching the MIC threshold of <15 µg/mL among all tested strains (Figure 5b).

Several derivatives (**A01**, **A02**, **A03**, **A08**, **A15**, **A18**) combined MIC < 15 µg/mL with TR > 5 against at least six of the eight pathogens tested (Table 1), identifying them as the most promising members of the series A.

**Figure 6:**
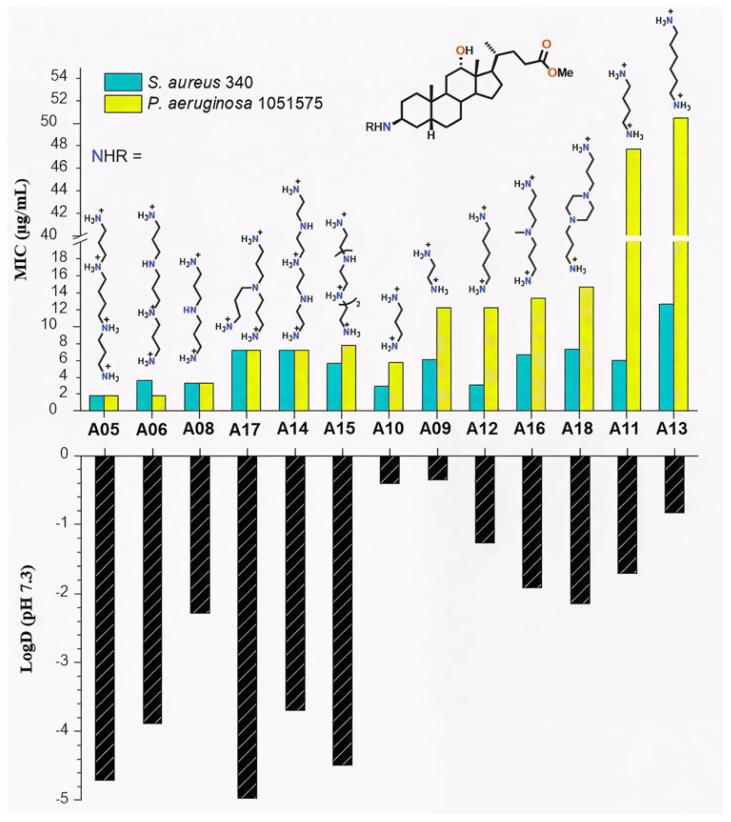
Influence of the βC-3 polyamine chain on LogD values and antibacterial activity (MIC) of Claramine A derivatives against *S. aureus* 340 and *P. aeruginosa* 1051575.

On the other hand, it is noteworthy that comparison of Claramine A structures with their MIC values indicates that the polyamine substituent at βC-3 strongly impacts antibacterial activity against both Gram-positive and Gram-negative strains. As illustrated in Figure 6 for the C-24 methyl ester series, derivatives bearing linear polyamines such as spermine or norspermine (**A05**, **A06**; MIC = 2–4 µg/mL) were significantly more active against *S. aureus* and *P. aeruginosa* strains than analogues containing branched chains (**A16**, **A17**; MIC = 7–14 µg/mL) or diamines such as cadaverine or putrescine (**A12**, **A11**; MIC = 6–50 µg/mL). Furthermore, this trend was more pronounced against Gram-negative *P. aeruginosa* than against Gram-positive *S. aureus*.

To rationalize these results, LogD values were calculated at physiological pH (7.3), accounting for the protonated polyamine side chains. LogD values ranged from −4.97 to −0.35. A strong correlation was observed between increased hydrophilicity (negative LogD) and low MICs (Figure 6).

Linear polyamine derivatives **A05** and **A06**, with LogD values of −4.71 and −3.89, displayed the lowest MICs, whereas **A11** and **A13** were less hydrophilic (LogD −1.71 and −0.83) and less active. The correlation was stronger for Gram-negative strains, consistent with the role of electrostatic interactions between cationic polyamines and the anionic LPS of the outer membrane. In contrast, activity against Gram-positive *S. aureus* appeared less dependent on hydrophilicity. While LogD strongly influences antibacterial activity, discrepancies between compounds with similar hydrophilicity (e.g., A06 vs. A14) suggest that steric and electronic features, such as molecular shape and charge distribution, play additional roles in modulating potency.

**Figure 7:**
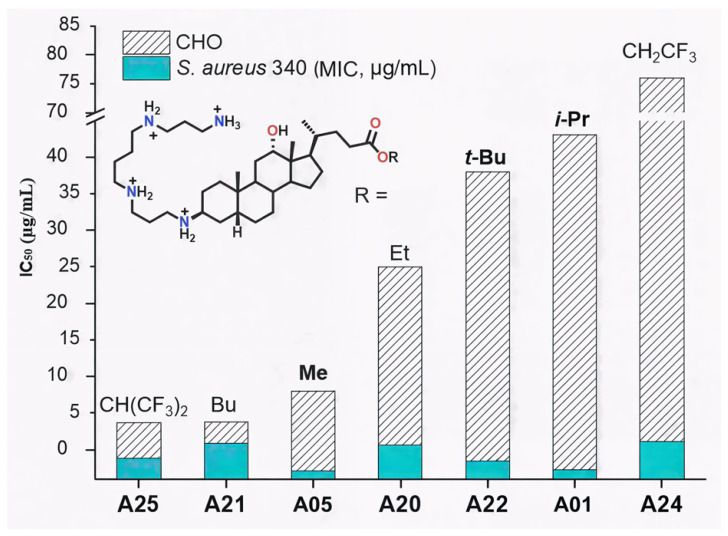
Influence of the ester group on the cytotoxicity (IC_50_, CHO cells) of Claramine A derivatives.

On the other hand, the ester substituent at C-24 also modulated cytotoxicity. For these linear alkyl esters, the butyl ester A21 (IC_50_ = 4 µg/mL) is more cytotoxic than the corresponding methyl (A05, IC_50_ = 8 µg/mL) and ethyl (A20, IC_50_ = 25 µg/mL) derivatives, consistent with the higher lipophilicity of the butyl analogue. (Figure 7).

**Figure 8:**
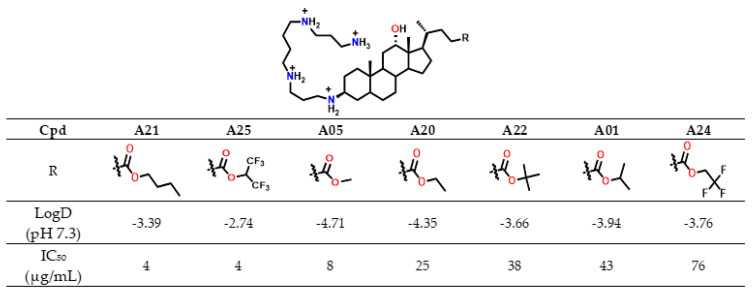
IC_50_ in CHO cells and LogD values of Claramines A bearing a spermine chain variation de l ester moiety.

This trend paralleled increasing lipophilicity (LogD −3.39 vs. −4.71 and −4.35), suggesting improved membrane permeability for less hydrophilic analogues. Similarly, perfluorinated derivative **A25** (LogD −2.74) was markedly more cytotoxic (IC_50_ = 4 µg/mL) than its trifluorinated analogue **A24** (LogD −3.76, IC_50_ = 76 µg/mL) (Figure 8). In contrast, branched alkyl esters (**A01**, **A22**; isopropyl and *tert*-butyl, respectively) were among the least cytotoxic derivatives (IC_50_ > 35 µg/mL), despite having higher LogD values than **A05** or **A20**.

Overall, LogD values, which remained negative for all analogues, did not consistently account for cytotoxicity, indicating that other structural factors influence this parameter. Notably, **A01** and **A22** combined low cytotoxicity with strong antibacterial activity (MIC < 4 µg/mL against *S. aureus* 340). Moreover, derivatives bearing isopropyl, methyl, or ethyl esters showed the most favorable antibacterial selectivity (TR), while *tert*-butyl and halogenoalkyl esters were not pursued further due to synthetic limitations or insufficient activity. In summary, several derivatives exhibited antibacterial activity against both Gram-positive and Gram-negative strains with MIC values below 15 µg/mL. Among them, compounds such as **A01** and **A15** emerged as the most promising candidates, combining favorable selectivity profiles with low cytotoxicity.

**Figure 9:**
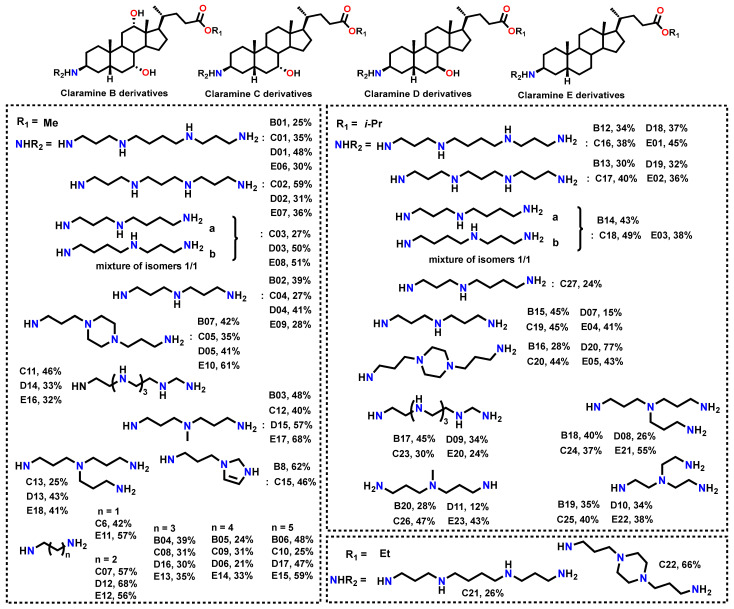
Isolated yields of the synthesized Claramine B–E derivatives.

Based on these results, subsequent series were designed to (i) further probe the influence of βC-3 polyamine structure, (ii) explore the role of hydroxyl substituents on the steroidal core, and (iii) refine ester functionalities. Using a synthetic strategy analogous to that developed for Claramine A derivatives, we constructed Claramine B–E libraries from the corresponding bile acids, namely cholic, chenodeoxycholic, ursodeoxycholic, and lithocholic acids (Figure 9).

**Figure 10:**
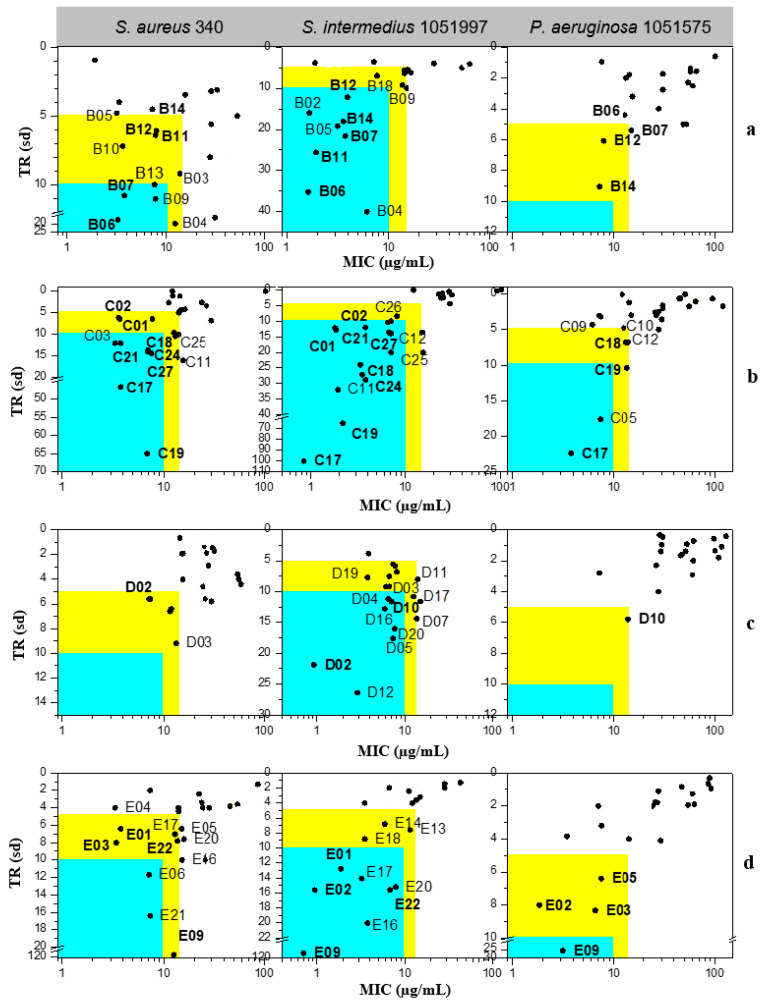
Antibacterial potential of (**a**) Claramine B, (**b**) Claramine C, (**c**) Claramine D, and (**d**) Claramine E derivatives against Gram-positive (*S. aureus* 340, *S. intermedius* 1051997) and Gram-negative (*P. aeruginosa* 1051575) strains. In the blue zone, derivatives exhibit strong antibacterial potential (TR > 10 and MIC < 10 µg/mL) against the indicated strain; in the yellow zone, derivatives display moderate potential (5 < TR < 10 and MIC < 15 µg/mL).

The antibacterial activity of Claramine B–E derivatives against three bacterial species is shown in Figure 10. TR values, calculated from MIC and IC_50_, generally indicated stronger activity against Gram-positive (*S. aureus* 340, *S. intermedius* 1051997) than against Gram-negative *P. aeruginosa* 1051575, in line with observations for Claramines A. The number of active derivatives was markedly higher for Gram-positive strains.

Across all series, numerous derivatives displayed MIC < 15 µg/mL against Gram-positive strains, with TR values frequently > 5. The trend was particularly striking for *S. intermedius*, where most compounds exhibited MIC < 10 µg/mL; some analogues (**C17**, **C19**, **E09**) reached TR values of 65–114. Against *P. aeruginosa*, most derivatives showed MIC = 10–100 µg/mL with low TR (<5), although a few (**C17**, **E09**) achieved MIC < 10 µg/mL with TR > 10.

Several derivatives, highlighted in Figure 10, were active across all three strains with MIC < 15 µg/mL and TR > 5. Their broader activity spectrum is summarized in Table 2, which identifies **B06**, **B07**, **C17**, **C19**, **C24**, and **E09** as the most promising, combining antibacterial activity (MIC = 0.7–30 µg/mL) with low cytotoxicity (IC_50_ > 60 µg/mL in CHO cells).

**Figure 11:**
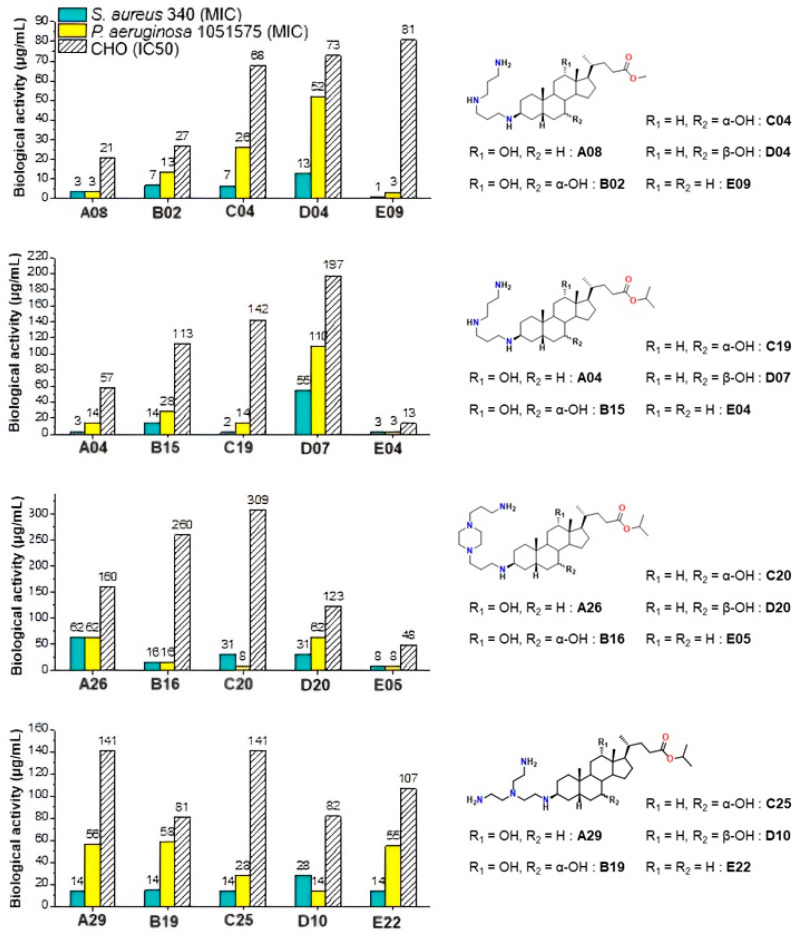
Effect of hydroxyl group modification on the antibacterial activity against *S. aureus* 340 and *P. aeruginosa* 1051575 (MIC) and cytotoxicity against CHO (IC_50_) of Claramine derivatives.

These results underline the importance of hydroxyl substitution patterns in modulating activity and cytotoxicity. In this context, Claramine C compounds, bearing a 7α-OH, showed the best overall activity (Figure 10b, Table 2), with several compounds (**C17**, **C19**, **C24**) active across multiple pathogens (MIC < 20 µg/mL). By contrast, Claramine D derivatives, bearing a 7β-OH, were mainly active against *S. intermedius* and displayed weak activity against Gram-negative strains (MIC > 20 µg/mL). On the other hand, Claramine B (7α-OH and 12α-OH) and E derivatives (no OH groups) showed comparable activities, largely confined to Gram-positive bacteria (MIC = 0.7–32 µg/mL). Some exceptions (**B07**, **E09**) also showed promising activity against Gram-negative strains. Overall, derivatives with α-OH substituents at C-7 and/or C-12, especially in combination with linear or cyclic polyamines at βC-3, yielded the most favorable antibacterial profiles (Figure 11).

Moreover, when compared with the parent compound Claramine A01, several derivatives from Series B–E (e.g., C17, C19, and E09) exhibited comparable or enhanced antibacterial activity, particularly against *Staphylococcus intermedius* and *Pseudomonas aeruginosa*, whereas derivatives lacking α-hydroxyl groups (Series D and E) were generally less active. This trend underscores the role of the bile acid core and hydroxyl substitution pattern in modulating antibacterial potency. Finally, it is noteworthy that cytotoxicity trends paralleled ester substitution but were also influenced by hydroxyl groups. Isopropyl esters generally conferred reduced cytotoxicity relative to methyl esters, confirming earlier observations. Moreover, Claramines C consistently displayed higher IC_50_ values than series A and B, whereas Claramines E were the most cytotoxic (IC_50_ < 60 µg/mL for most derivatives except **E09**, **E22**). Overall, cytotoxicity was minimized by the presence of hydroxyl groups (especially α-OH at C-7 and/or C-12) and by isopropyl esters at C-24.

### Development of a QSAR Model for Cytotoxicity

The development of molecules of interest requires not only strong antimicrobial activity but also reduced cytotoxicity. The latter criterion appears to be of primary importance and will be considered as the preferential parameter for the identification of promising candidates. Thus, to better rationalize the cytotoxicity of Claramines, a Quantitative Structure–Activity Relationship (QSAR) model was constructed. Cytotoxicity was expressed as Log(IC_50_) and correlated with molecular descriptors according to the general relation:

Cytotoxicity = f (Descriptors)

In the first step, over 1600 molecular descriptors were computed using E-Dragon 5, after 3D optimization of Claramine derivatives structures (MM+ force field followed by PM3 refinement). These descriptors covered constitutional, topological, geometrical, and quantum classes, including molecular properties such as LogD and hydrogen-bond donors (Table S1). A reduced dataset of 95 Claramine derivatives with reliable IC_50_ values was then divided into a training set (83 compounds) and a test set (12 compounds). Multiple linear regression (MLR) with stepwise selection was applied to minimize collinearity and retain the most significant descriptors (Table 3).

The best model included six variables:

Log(IC_50_) = 2.49 − 2.134 × **MATS4m**1.082 × **GATS6m** − 1.352 × **EEig06d** + 1.706 × **EEig07d** − 0.286 × **Mor27u** − 8.901.10^−2^ × **Hy**

with N*_training_*_(*test*)_ = 83 (12); R^2^*_training_* = 0.72; R^2^*_test_* = 0.79; F = 32; MCE = 0.044; RMCE = 0.210

with R^2^training = 0.72, R^2^test = 0.79, F = 32, and low residual errors (MSE = 0.044; RMSE = 0.210).

**Figure 12:**
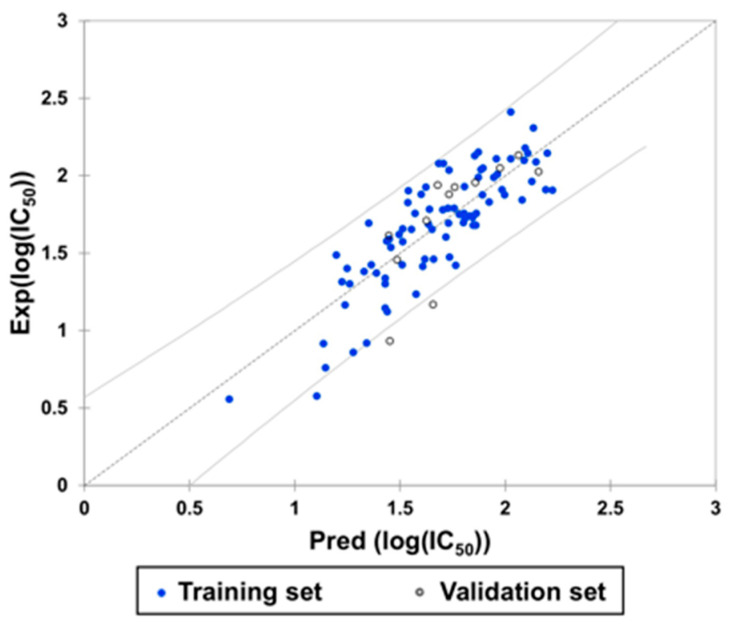
Experimental and predicted Log(IC_50_) values according to the derived equation.

A subsequent model evaluation step revealed a strong correlation between predicted and experimental Log(IC_50_) values for both the training and test sets (Figure 12, Table 4).

**Figure 13:**
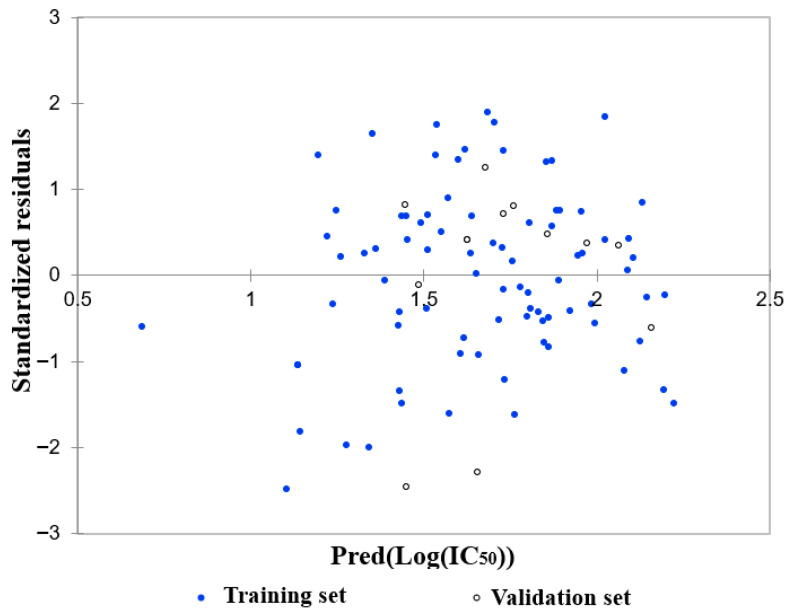
Residual distribution as a function of the predicted Log(IC_50_).

The residuals were normally distributed (Figure 13; Durbin–Watson = 1.98), further confirming the statistical robustness and predictive power of the model.

The most influential descriptors in the model, MATS4m and EEig07d, describe structural and electronic characteristics that can be directly related to the Claramine framework. MATS4m, a Moran autocorrelation descriptor weighted by atomic mass, reflects the degree of molecular branching and compactness, indicating that steric effects influence cytotoxicity. EEig07d and EEig06d are edge adjacency indices weighted by atomic dipole moments and are associated with the global charge distribution and electronic polarization of the molecule. Similarly, GATS6m, a Geary autocorrelation parameter based on electronegativity, measures how electronic effects propagate through the molecular scaffold. Mor27u represents a 3D-MoRSE signal sensitive to molecular size and shape, while Hy corresponds to the number of hydrophilic groups. Together, these descriptors suggest that cytotoxicity is predominantly modulated by a combination of steric bulk, molecular polarity, and charge distribution, consistent with the amphiphilic behavior of Claramine derivatives. Although the model accounted for 72% of the variance in cytotoxicity, additional descriptors may explain the remaining variability. Alternative statistical approaches such as principal component analysis could help reduce descriptor redundancy and further refine the predictive power.

## 3. Conclusions

The Claramine family (A–E) was synthesized in good yields via a concise route involving esterification, oxidation, and stereoselective reductive amination. Most derivatives exhibited promising antibacterial activity against Gram-positive and Gram-negative bacteria, with selected compounds (**A01**, **A15**, **C17**, **C19**) combining broad-spectrum activity (MIC < 15 µg/mL) and low cytotoxicity. SAR analysis highlighted three key factors: (i) Polyamine chain—Linear spermine-type side chains at βC-3 favored the lowest MIC values across strains, (ii) Ester function—Isopropyl esters at C-24 reduced cytotoxicity and were associated with improved antibacterial selectivity, (iii) Hydroxyl substitution—α-OH groups at C-7 and/or C-12 enhanced antibacterial activity, particularly against Gram-negative strains, while β-OH at C-7 reduced potency. Finally, the MLR-based QSAR model established here demonstrates that Claramine cytotoxicity is primarily driven by electronic (molecular polarity, charge distribution) and steric (2D/3D architecture) factors rather than simple physicochemical parameters. Taking them together, these results identify specific substitution patterns (linear polyamine at βC-3, isopropyl ester at C-24, α-OH at C-7 and/or C-12) as favorable for maximizing antibacterial potential while limiting cytotoxicity. The model is predictive (R^2^test = 0.79) and provides a useful framework for guiding the design of new derivatives exhibiting better selectivity. Studies are now under current investigation to improve the structure of Claramine derivatives and will be reported in due course.

## 4. Experimental Section

All solvents were purified according to reported procedures, and reagents were used as commercially available. Methanol, ethyl acetate, dichloromethane, ammonia and petroleum ether (35–60 °C) were purchased from SDS and used without further purification. Column chromatography was performed on SDS silica gel (70–230 mesh). ^1^H NMR and ^13^C NMR spectra were recorded in CDCl_3_ on a Bruker AC 250 spectrometer working at 250 MHz and 63 MHz, respectively (the usual abbreviations are used: s: singlet, d: doublet, t: triplet, q: quadruplet, m: multiplet). Tetramethylsilane was used as internal standard. All chemical shifts are given in ppm. The purity of the compounds was verified by analytical HPLC (C18 column, eluent CH3CN/water/TFA, 2.3 mL/min) with a PDA detector from 210 to 310 nm. All compounds showed a purity greater than 95%, as determined by analytical HPLCPDA at 214 and 254 nm. Molecular masses of the compounds were determined by electrospray ionization mass spectrometry (ESI-MS) using either a QStar Elite (Applied Biosystems SCIEX, Concord, ON, Canada) or a 3200 QTRAP (Applied Biosystems SCIEX, Concord, ON, Canada) spectrometer. Samples were ionized in positive electrospray mode under the following conditions: ion spray voltage (ISV), 5500 V; orifice voltage (OR), 20 V; nebulizing gas pressure (air), 20 psi. Software Marvin sketch version 20.6 (Chemaxon, Budapest, Hungary) was used for LogD calculation at pH 7.4 involving default settings parameters.

### 4.1. General Synthetic Procedure for the Preparation of Claramine Derivatives A–E Exemplified for Claramine A01 (See Supporting Information for the Other Compounds)

#### 4.1.1. Synthesis of Isopropyl Deoxycholate

In a 250 mL two necked round flask was introduced in 70 mL of isopropanol and 30 mL of dichloromethane 10 g of deoxycholic acid (0.0255 mol) and 2.2 g of para-toluene sulfonic acid (0.013 mol). The mixture was heated at reflux under vigorous stirring for 8 h. The solvents were subsequently removed, and 100 mL of dichloromethane was added. The organic phase was washed 3 times with 50 mL of NHCO_3_ (10%) solution. The aqueous phases were extracted twice with dichloromethane and the combined organic phases were dried over Na_2_SO_4_, filtered, and concentrated in vacuo to afford the expected product as a white solid in 90% yield. NMR ^1^H (250 MHz, CDCl_3_): δ (ppm) = 4.94 (m, 1H), 3.92 (m, 1H), 3.53 (m, 1H), 2.31–2.09 (m, 2H), 1.78–0.85 (m, 38H), 0.61 (s, 3H). NMR ^13^C (63 MHz, CDCl_3_): δ (ppm) = 173.68, 72.86, 71.37, 67.17, 47.99, 47.05, 46.32, 41.95, 36.23, 35.86, 35.17, 35.11, 33.97, 33.38, 31.61, 30.80, 30.20, 28.49, 27.42, 27.05, 26.01, 23.58, 22.99, 21.70, 17.06, 12.53.

#### 4.1.2. Synthesis of 3-oxo Isopropyl Deoxycholate

In a 250 mL two necked round flask was introduced in 100 mL of toluene and 50 mL of acetone 11 g of isopropyl deoxycholate **1** (0.025 mol). 2 equivalents of aluminum *tert*-butoxide (12.3 g, 0.050 mol) were subsequently added and stirring was maintained under reflux for 12 h. 50 mL of a 2N H_2_SO_4_ solution was added and the mixture was stirred for an additional 1 h.

The organic phase was washed 3 times of a 2N H_2_SO_4_ solution and 50 mL of water. The combined organic phases were dried over Na_2_SO_4_, filtered, and concentrated in vacuo to afford a crude product which was purified by flash chromatography on silica gel (ethylacetate/petroleum ether (1/1)). The expected 3-oxo isopropyl deoxycholate **2** was successfully obtained as a white solid in 70% yield. NMR ^1^H (250 MHz, CDCl_3_): δ (ppm) = 5.00 (m, 1H), 4.04 (m, 1H), 2.46–2.12 (m, 4H), 2.06–0.96 (m, 35H), 0.71 (s, 3H). NMR ^13^C (63 MHz, CDCl_3_): δ (ppm) = 212.74, 173.57, 72.98, 67.34, 48.22, 47.59, 46.70, 44.30, 42.33, 37.04, 36.92, 35.87, 35.05, 34.45, 34.11, 31.76, 31.00, 29.06, 27.40, 26.64, 25.55, 23.58, 22.38, 21.81, 17.42, 12.75.

#### 4.1.3. Synthesis of 3-Spermino Isopropyl Deoxycholate (Claramine A01)

In a 100 mL two necked round flask was introduced 100 mL of methanol. A mixture of the ketone **2** (3.5 g, 8 × 10^−3^ mol), titanium (IV) isopropoxide (7.15 mL, 2.4 × 10^−2^ mol), and spermine (3.2 g, 1.6 × 10^−2^ mol) was stirred under argon at room temperature for 24 h. After cooling the flask at −20 °C, sodium borohydride (0.9 g, 2.4 × 10^−2^ mol) was then added and the resulting mixture was stirred for additional 12 h. The reaction was then quenched by adding water (4 mL). Stirring was continued at room temperature for 1 h then the reaction mixture was filtered over a pad of Celite which was subsequently rinsed with NH_4_OH and methanol. The mixture was concentrated in vacuo to afford the expected crude compound which was purified by flash chromatography on silica gel using CH_2_Cl_2_/MeOH/NH_4_OH (32%) 7:3:1 as eluent. The expected Claramine A01 was obtained as a viscous yellow oil in 52% yield. NMR ^1^H (250 MHz, CD_3_OD): δ (ppm) = 4.96 (m, 1H), 3.94 (m, 1H), 2.88–2.46 (m, 13H), 2.39–2.18 (m, 2H), 2.04–1.12 (m, 40H), 1.01–0.95 (m, 7H), 0.71 (s, 3H). NMR ^13^C (63 MHz, CD_3_OD): δ (ppm) = 175.66, 74.15, 68.95, 59.06, 50.74, 50.70, 49.30, 48.97, 48.42, 48.27, 47.77, 45.83, 44.08, 40.78, 37.58, 37.14, 36.82, 35.97, 35.92, 34.94, 34.59, 33.56, 32.72, 32.45, 30.52, 29.98, 28.84, 28.65, 28.40, 28.29, 27.64, 25.04, 24.10, 22.27, 17.71, 13.37. MS (ESI^+^): m/z 619.5519 ([M + H]^+^).

#### 4.1.4. Synthesis of Claramine A01 Hydrochloride Salt

Claramine A01 is dissolved in a minimum of anhydrous methanol and an anhydrous HCl solution in diethyl ether (2M, 8 equivalents) was slowly added under vigorous. The formed precipitate was filtrated, washed with anhydrous diethyl ether, and dried under vacuum. The Claramine A01 hydrochloride salt is obtained in a quantitative yield as a white solid stable to air and moisture.

### 4.2. Determination of Minimal Inhibitory Concentrations

Antimicrobial activity of the compounds was studied by determination of minimal inhibitory concentrations (MIC) according to the NCCLS guidelines M7-A2 using the microbroth dilution methods [25]. The bacteria strains were grown on trypticase soy agar (Becton Dickinson) at 37 °C for 24 h. Inocula were prepared in TCE (tryptone 0.1%, NaCl 8%, wt/vol) by ajusting the turbidity at 623 nm to obtain 1–3 10^5^ CFU/mL.

Antimicrobial activities of the compounds were determined by using a broth microdilution method performed in sterile 96-well microplates. All compounds were solubilized in water at a concentration of 10 mM and were transferred to each microplate well in order to obtain a two-fold serial dilution in 100 µL of broth and 100 µL of inoculum containing 2–6 10^5^ CFU of each bacterium were added to each well. Some wells were reserved for positive controls and inoculum viability. After 24 h incubation, MIC was defined for each agent from duplicate observations as the lowest concentration of compound allowing no visible growth.

### 4.3. Cytotoxicity Assays

The cytotoxic activities of compounds were assessed on Chinese hamster Ovary cells (CHO-K1) provided from ATCC-LGC Standards Sarl (Molsheim, France). Cells were maintained in Mc Coy’s 5A (CHO) medium supplemented with 10% bovine calf serum, 2 mM glutamine, and 100 U mL^−1^/10 µg mL^−1^ penicillin/streptomycin mixtures. For the cytotoxicity experiments, they were seeded in 96-well plates and incubated at 37 °C in humidified atmosphere containing 5% CO_2_ overnight, then concentrations of compounds were incorporated in triplicate cultures. After a 24-h incubation period at 37 °C, cells were submitted to three successive washes in phosphate buffer saline (PBS) and cell viability was evaluated by two different vital dyes:

i. A first set of cell cultures was incubated in PBS containing 10% WST-1 for 30 min at 37 °C, 5% CO_2_. Cell viability was evaluated by the assessment of WST-1 absorbance at 450 nm in a microplate spectrophotometer.
ii. A second set of cell cultures was placed into Neutral Red medium (50 μg mL^−1^ Neutral Red in complete medium) and incubated for 3 h at 37 °C, 5% CO_2_. Then the Neutral Red medium was removed and the distaining solution (50% ethanol, 1% acetic acid, 49% distilled water; 50 µL per well) was added into the wells. The plates were shaken for 15–20 min at room temperature in the dark. Cell viability was evaluated by the assessment of absorbance at 540 nm in a microplate spectrophotometer.

Results were expressed as percentages of cell viability about the control (culture medium-only), which corresponded to 100% cell viability. Dose-response curves were calculated by non-linear regression analysis with TableCurve 2D software (V 5.0.1) (Systat Software Inc., San Jose, CA, USA). The Inhibitory Concentration 50% (IC_50_) was defined as the concentration of compounds that induced a 50% decrease of viable cells.

### 4.4. SQAR Analysis

A quantitative structure–activity relationship (QSAR) study was performed to rationalize the cytotoxicity of Claramine derivatives based on their molecular features. Three-dimensional molecular structures were first generated using HyperChem (version 6.03). Each compound was pre-optimized with the MM+ molecular mechanics force field and subsequently refined by the semi-empirical PM3 method (Parametric Method 3) until the energy gradient reached a convergence limit of 0.01 kcal·Å^−1^. The optimized geometries were exported as HyperChem (.HIN) files for descriptor calculation.

More than 1600 theoretical molecular descriptors were computed using the E-Dragon (version 5) software, freely available online. The descriptor set encompassed constitutional, topological, geometrical, electronic, and hybrid parameters. Prior to statistical analysis, the dataset was carefully curated: descriptors showing constant or redundant values were removed to avoid noise and multicollinearity. The relationship between cytotoxicity (expressed as Log(IC_50_)) and molecular descriptors was established through multiple linear regression (MLR) implemented in XLSTAT (Version 2022.1, Lumivero, Denver, CO, USA). A stepwise regression procedure was applied to select the most relevant descriptors and construct the final predictive model, expressed as Cytotoxicity = f (Descriptors). The main statistical parameters, including the coefficient of determination (R^2^), standard deviation, mean squared error (MSE), root mean squared error (RMSE), and F-statistic, were calculated to assess model performance.

Model quality was evaluated through both internal and external validation. The coefficient of determination (R^2^) was used as the principal indicator of fit, reflecting the proportion of variance in the dependent variable explained by the model. In accordance with accepted standards, R^2^ > 0.7 was considered indicative of good predictive ability. The present model accounted for 72% of the variance in Log(IC_50_) (R^2^ training = 0.72), and external validation confirmed its robustness (R^2^ test = 0.79). Residuals were normally distributed and randomly dispersed around zero (Durbin–Watson ≈ 2), further validating the model’s reliability.

## Figures and Tables

**Table 1:** Antibacterial activity and cytotoxicity of Claramines A derivatives with the highest antibacterial potential.

	MIC (µg/mL)								IC_50_ (µg/mL)
	*S. aureus*		*S. intermedius*	*E. faecalis*	*E. coli*		*P. aeruginosa*		CHO
	25923 ^a^	340	1051997	29212 ^a^	25922 ^a^	1956	27853 ^a^	1051575	
**A01**	**2**	**2**	**8**	**30**	**4**	**15**	**8**	**15**	**43**
**A02**	4	2	2	8	8	15	8	8	51
**A03**	7	2	4	14	14	14	14	14	73
**A08**	2	3	0.4	3	3	13	3	3	21
**A15**	**4**	**6**	**1**	**16**	**8**	**14**	**8**	**8**	**93**
**A18**	**15**	**7**	**4**	**30**	**7**	**ND ^c^**	**15**	**15**	**135**
**Ery ^b^**	**1**	**1**	**1**	**1**	**ND ^c^**	**ND ^c^**	**ND ^c^**	**ND ^c^**	**ND ^c^**
**Col ^b^**	**ND ^c^**	**ND ^c^**	**ND ^c^**	**ND ^c^**	**0.5**	**0.5**	**0.5**	**0.5**	**ND ^c^**

^a^: ATCC strains; ^b^ Ery: Erythromycine; Col: Colistine; ^c^ ND: Not Determined.

**Table 2:** Antibacterial activity and cytotoxicity of Claramine B, C, D, and E derivatives with the highest antibacterial potential.

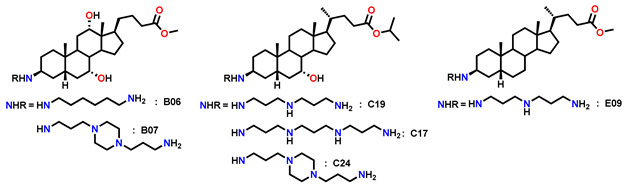									
	**MIC (µg/mL)**								**IC_50_** **(µg/mL)**
**Cpd**	*S. a.*		*S. i.*	*E. f.*	*E. c.*		*P. a.*		CHO
	25923 ^a^	340	1051997	29212 ^a^	25922 ^a^	1956	27853 ^a^	1051575	
**B06**	**3**	**3**	**2**	**7**	**7**	**ND ^b^**	**13**	**13**	**60**
**B07**	**4**	**8**	**4**	**8**	**15**	**ND ^b^**	**30**	**15**	**80**
**B11**	8	8	2	30	30	16	8	16	50
**B12**	8	8	4	32	4	62	16	8	48
**B14**	7	14	4	30	4	30	15	7	67
**C01**	4	4	2	15	30	60	30	7	24
**C02**	4	4	2	15	15	29	29	7	22
**C17**	**4**	**2**	**0.8**	**8**	**16**	**10**	**16**	**4**	**85**
**C18**	7	7	4	7	14	14	14	14	95
**C19**	**7**	**2**	**2**	**4**	**14**	**14**	**28**	**14**	**140**
**C21**	4	4	4	2	15	30	15	15	45
**C24**	**8**	**8**	**4**	**15**	**15**	**30**	**30**	**30**	**110**
**C27**	7	7	7	28	56	28	56	56	100
**D02**	7	4	1	30	4	60	30	7	20
**D10**	28	28	7	56	110	56	110	14	80
**E01**	4	4	2	8	15	8	15	8	24
**E02**	7	7	1	14	14	7	28	2	15
**E03**	3	7	2	7	100	14	27	7	56
**E05**	15	8	15	30	15	15	60	8	50
**E09**	**16**	**0.7**	**0.7**	**32**	**32**	**3**	**32**	**3**	**80**
**E22**	14	14	7	55	110	55	110	55	105

^a^: ATCC strain *S. a.*: *Staphylococcus aureus*; *S.i.*: *Staphylococcus intermedius*; *E. f.*: *Enterococccus faecalis*; *E. c.*: *Escherichia coli*; *P. a.*: *Pseudomonas aeruginosa*. ^b^ ND: not determined

**Table 3:** Correlation matrix of the descriptors included in the derived equation. Bold correlation coefficients (r) indicate the relative contribution of each descriptor to the explanation of the dependent variable Log(IC_50_). (EEig07d > MATS4m > Mor27u > EEig06d > GATS6m > Hy).

	Log(IC_50_)	MATS4m	GATS6m	EEig06d	EEig07d	Mor27u	Hy
**Log(IC_50_)**	1	**−0.412**	**−0.125**	**0.270**	**0.480**	**−0.352**	**−0.084**
MATS4m		1	−0.154	0.003	0.001	0.045	−0.288
GATS6m			1	0.234	0.180	−0.312	0.002
EEig06d				1	0.807	−0.394	0.041
EEig07d					1	−0.251	0.183
Mor27u						1	0.380
Hy							1

**Table 4:** Experimental and predicted Log(IC_50_) values from derived equation for Claramine derivatives in the validation set.

	Log(IC_50_)		Difference
	Experimental	Predicted	
**A02**	1.711	1.625	−0.086
**A11**	0.934	1.450	0.517
**A17**	1.460	1.485	0.025
**A19**	2.135	2.062	−0.073
**C12**	1.958	1.857	−0.101
**C17**	1.928	1.759	−0.169
**D01**	1.617	1.446	−0.171
**D11**	2.051	1.973	−0.077
**D16**	1.882	1.732	−0.151
**D18**	1.172	1.655	0.483
**E13**	1.942	1.678	−0.264
**E22**	2.028	2.158	0.130

## Data Availability

The data are contained within the article.

## Supplementary Materials

The following supporting information can be downloaded at: https://www.mdpi.com/article/10.3390/molecules30234614/s1 and includes the experimental procedures and spectroscopic data for all compounds reported in this study.
