# Supplementary material for: Synthesis and QSAR Studies of Claramine Derivatives, a New Class of Broad-Spectrum Antimicrobial Agents
Source: Molecules. 2025 Dec 1;30(23):4614. doi: 10.3390/molecules30234614 (PMC12693182; doi:10.3390/molecules30234614)
Supplement: Supplementary file 1 [file molecules-30-04614-s001.zip › molecules-3946838-supplementary.pdf]

# Supporting information

## Material and reagents

All syntheses were carried out using solvents purified according to standard procedures. Commercial reagents, including bile acids and amines, were purchased from VWR and Sigma-Aldrich and used without further purification. Reaction progress was monitored by thin-layer chromatography (TLC) using phosphomolybdic acid (10% w/w solution in absolute ethanol) as staining reagent. “Yield” refers to isolated yield.
The chemical structures of all synthesized products were confirmed by proton ($^1H$) and carbon ($^{13}C$) NMR spectroscopy, recorded on a Bruker Avance 250 MHz spectrometer. Tetramethylsilane (TMS) was used as an internal standard. Chemical shifts (δ) are reported in parts per million (ppm) relative to the residual solvent peak: $CDCl_3$, δ$^1H$ 7.27 ppm and δ$^{13}C$ 77.00 ppm; $CD_3OD$, δ$^1H$ 3.31 ppm and δ$^{13}C$ 49.15 ppm. Coupling constants (J) are reported in hertz (Hz). Signal multiplicities in the $^1H$ NMR spectra are designated as follows: s = singlet, d = doublet, t = triplet, q = quartet, m = multiplet.
Molecular masses of the compounds were determined by electrospray ionization mass spectrometry (ESI-MS) using either a QStar Elite (Applied Biosystems SCIEX) or a 3200 QTRAP (Applied Biosystems SCIEX) spectrometer. Samples were ionized in positive electrospray mode under the following conditions: ion spray voltage (ISV), 5500 V; orifice voltage (OR), 20 V; nebulizing gas pressure (air), 20 psi.

## 1. Esterification procedure

All intermediate esters were prepared from the corresponding bile acid precursor and the appropriate alcohol following the procedure described for the synthesis of methyl deoxycholate

**162**.

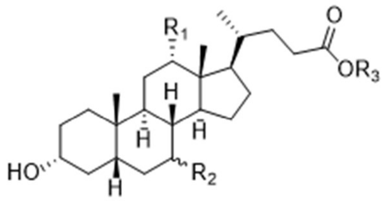

$R_1$ = OH, $R_2$ = H :
$R_3$ = Me : **162**
*i*-Pr : **163**
Et : **164**
Bu : **165**
*t*-Bu : **166**
$CH_2CF_3$ : **167**
$CH(CF_3)_2$ : **168**

$R_1$ = $R_2$ = α-OH :
$R_3$ = Me : **176**
*i*-Pr : **177**
Et : **178**

$R_1$ = H, $R_2$ = α-OH :
$R_3$ = Me : **182**
*i*-Pr : **183**
Et : **184**

$R_1$ = H, $R_2$ = β-OH
$R_3$ = Me : **190**
*i*-Pr : **191**

$R_1$ = $R_2$ = H :
$R_3$ = Me : **194**
*i*-Pr : **195**

In a round-bottom flask equipped with a magnetic stir bar, deoxycholic acid 103 (8 g, 20.4 mmol) and p-toluenesulfonic acid (1.5 g, 9 mmol) were dissolved in methanol (40 mL) and dichloromethane (30 mL). The reaction mixture was heated to reflux ($CH_2Cl_2$) and stirred for 8 h. After completion, the solvents were evaporated, and the residue was dissolved in $CH_2Cl_2$ (100 mL) and washed with aqueous sodium bicarbonate solution (10%, 3 × 50 mL). The aqueous layers were extracted with $CH_2Cl_2$ (2 × 50 mL). The combined organic phases were dried over anhydrous sodium sulfate, filtered, and concentrated under reduced pressure to afford the desired product **162** (98% yield).

**Methyl desoxycholate 162** ($C_{25}H_{42}O_4$). **NMR $^1$H** (250 MHz, $CD_3OD$) : δ (ppm) = 3.95 (m, 1H), 3.65 (s, 3H), 3.53 (m, 1H), 2.44-2.18 (m, 2H), 1.95-1.73 (m, 8H), 1.62-0.99 (m, 21H), 0.93 (s, 3H), 0.71 (s, 3H). **NMR $^{13}$C** (63 MHz, $CD_3OD$) : δ (ppm) = 176.53, 74.07, 72.63, 52.15, 49.36, 48.14, 47.67, 43.74, 37.56, 37.32, 36.82, 36.59, 35.43, 34.91, 32.33, 31.96, 31.18, 30.04, 28.78,28.56, 27.61, 25.02, 23.91, 17.70, 13.38

**Isopropyl desoxycholate 163** ($C_{27}H_{46}O_4$). **NMR $^1$H** (250 MHz, $CDCl_3$) : δ (ppm) = 4.94 (m, 1H), 3.92 (m, 1H), 3.53 (m, 1H), 2.31-2.09 (m, 2H), 1.78-0.85 (m, 38H), 0.61 (s, 3H). **NMR $^{13}$C** (63 MHz, $CDCl_3$) : δ (ppm) = 173.68, 72.86, 71.37, 67.17, 47.99, 47.05, 46.32, 41.95, 36.23, 35.86, 35.17, 35.11, 33.97, 33.38, 31.61, 30.80, 30.20, 28.49, 27.42, 27.05, 26.01, 23.58, 22.99, 21.70, 17.06, 12.53. Yield : 90%

**Ethyl desoxycholate 164** ($C_{26}H_{44}O_4$). **NMR $^1$H** (250 MHz, $CDCl_3$) : δ (ppm) = 4.10 (q, *J* = 7.1 Hz, 2H), 3.92 (m, 1H), 3.54 (m, 1H), 2.37-2.10 (m, 2H), 1.83-0.85 (m, 35H), 0.62 (s, 3H). **NMR $^{13}$C** (63 MHz, $CDCl_3$) : δ (ppm) = 174.20, 72.88, 71.39, 60.05, 47.97, 47.01, 46.31, 41.95, 36.22, 35.85, 35.16, 33.97, 33.37, 31.28, 30.76, 30.20, 28.46, 27.42, 27.05, 26.01, 23.58, 22.98, 17.06, 14.11, 12.53. Yield : 96%

**Butyl desoxycholate 165** ($C_{28}H_{48}O_4$). **NMR $^1$H** (250 MHz, $CDCl_3$) : δ (ppm) = 4.04 (m, 2H), 3.95 (m, 1H), 3.57 (m, 1H), 2.53-0.88 (m, 41H), 0.65 (s, 3H). **NMR $^{13}$C** (63 MHz, $CDCl_3$) : δ

(ppm) = 174.21, 72.75, 71.26, 63.90, 47.90, 46.91, 46.25, 41.91, 36.17, 35.80, 35.13, 33.92, 33.31, 31.20, 30.73, 30.49, 30.16, 28.45, 27.01, 25.95, 22.95, 18.96, 16.97, 13.54, 12.47. Yield : 96%

**Trifluoroethyl desoxycholate 167** ($C_{26}H_{41}F_3O_4$). **NMR $^1$H** (250 MHz, $CDCl_3$) : δ (ppm) = 3.97 (m, 1H), 3.66-3.60 (m, 3H), 2.78-0.80 (m, 34H), 0.67 (s, 3H). **NMR $^{13}$C** (63 MHz, $CDCl_3$) : δ (ppm) = 174.52, 73.16, 71.84, 51.32, 48.36, 47.48, 46.64, 42.28, 36.65, 36.20, 35.36, 35.14, 34.21, 33.85, 31.17, 31.03, 30.66, 28.84, 27.43, 27.21, 26.20, 23.68, 23.15, 17.38, 12.75. Yield : 80%

**Hexafluoroisopropyl desoxycholate 168** ($C_{27}H_{40}F_6O_4$). **NMR $^1$H** (250 MHz, $CDCl_3$) : δ (ppm) = 3.95 (m, 1H), 3.63-3.55 (m, 3H), 2.72-2.16 (m, 3H), 2.02-0.88 (m, 30H), 0.65 (s, 3H). **NMR $^{13}$C** (63 MHz, $CDCl_3$) : δ (ppm) = 174.55, 73.07, 71.72, 51.28, 48.27, 47.35, 46.56, 42.19, 36.60, 36.11, 35.30, 35.11, 34.13, 33.73, 31.12, 30.96, 30.48, 28.75, 27.39, 27.16, 26.14, 23.63, 23.07, 17.28, 12.67. Yield : 60%

**Methyl cholate 176** ($C_{25}H_{42}O_5$). Obtained after purification by flash column chromatography on silica gel (eluent: petroleum ether/ethyl acetate, 1:1). **NMR $^1$H** (250 MHz, $CDCl_3$) : δ (ppm) = 3.88 (m, 1H), 3.66-3.53 (m, 4H), 2.45-0.68 (m, 37H). **NMR $^{13}$C** (63 MHz, $CDCl_3$) : δ (ppm) = 174.87, 73.36, 72.87, 68.72, 51.53, 50.48, 46.96, 46.33,41.61, 41.29, 39.35, 38.38, 35.34, 35.12, 34.64, 34.13, 33.17, 30.94, 29.31, 27.97, 26.36, 23.13, 22.40, 17.24, 12.44. Yield : 78%

**Isopropyl cholate 177** ($C_{27}H_{46}O_5$). **NMR $^1$H** (250 MHz, $CDCl_3$) : δ (ppm) = 4.92 (m, 1H), 4.05-3.78 (m, 2H), 3.36-3.18 (m, 4H), 2.26-0.81 (m, 36H), 0.60 (m, 3H). **NMR $^{13}$C** (63 MHz, $CDCl_3$) : δ (ppm) = 173.98, 73.11, 71.88, 68.47, 67.38, 47.06, 46.44, 41.59, 39.50, 35.31, 34.81, 31.76, 30.97, 30.40, 28.15, 2755, 26.30, 25.85, 23.36, 22.47, 21.89, 17.34, 12.48. Yield : 90%

**Methyl chenodesoxycholate 182** ($C_{25}H_{42}O_4$). **NMR $^1$H** (250 MHz, $CDCl_3$) : δ (ppm) = 3.76 (m, 1H), 3.59 (s, 3H), 3.36 (m, 1H), 2.28-0.83 (m, 34), 0.59 (s, 3H). **NMR $^{13}$C** (63 MHz, $CDCl_3$) : δ (ppm) = 174.53, 71.56, 68.04, 55.67, 51.29, 50.22, 42.39, 41.40, 39.53, 39.19, 35.25, 35.13, 34.84, 34.58, 32.67, 30.78, 30.74, 30.44, 27.98, 23.41, 22.65, 20.44, 18.10, 11.55. Yield : 90%

**Isopropyl chenodesoxycholate 183** ($C_{27}H_{46}O_4$). **NMR $^1$H** (250 MHz, $CDCl_3$) : δ (ppm) = 4.97 (m, 1H), 3.82 (m, 1H), 3.42 (m, 1H), 2.34-0.88 (m, 40), 0.64 (s, 3H). **NMR $^{13}$C** (63 MHz, $CDCl_3$) : δ (ppm) = 173.63, 71.94, 68.44, 67.24, 56.02, 50.52, 42.74, 41.67, 39.97, 39.75, 39.59,

35.44, 35.28, 35.09, 34.76, 32.98, 31.69, 31.08, 30.76, 28.06, 23.67, 22.75, 21.79, 20.65, 18.28, 11.75. Yield : 72%

**Ethyl chenodesoxycholate 184** ($C_{26}H_{44}O_4$). **NMR $^1$H** (250 MHz, $CDCl_3$) : δ (ppm) = 4.12 (q, *J* = 7.1 Hz, 2H), 3.82 (m, 1H), 3.43 (m, 1H), 2.39-0.89 (m, 37H), 0.64 (s, 3H). **NMR $^{13}$C** (63 MHz, $CDCl_3$) : δ (ppm) = 174.11, 71.99, 68.48, 60.05, 56.01, 50.55, 42.76, 41.68, 40.02, 39.77, 39.61, 35.45, 35.33, 35.11, 34.78, 33.01, 31.38, 31.08, 30.80, 28.08, 23.71, 22.76, 20.67, 18.31, 14.21, 11.78. Yield : 75%

**Methyl ursodesoxycholate 190** ($C_{25}H_{42}O_4$). **NMR $^1$H** (250 MHz, $CDCl_3$) : δ (ppm) = 3.63 (s, 3H), 3.54 (m, 2H), 2.39-2.12 (m, 3H), 1.99-0.97 (m, 25H), 0.89 (m, 6H), 0.64 (s, 3H). **NMR $^{13}$C** (63 MHz, $CDCl_3$) : δ (ppm) = 174.67, 71.13, 71.10, 55.71, 54.83, 51.44, 43.63, 43.55, 42.35, 40.05, 39.15, 37.21, 36.87, 35.19, 34.84, 33.95, 30.97, 30.90, 30.16, 28.52, 26.80, 23.32, 21.08, 18.27, 12.03. Yield : 89%

**Isopropyl ursodesoxycholate 191** ($C_{27}H_{46}O_4$). **NMR $^1$H** (250 MHz, $CDCl_3$) : δ (ppm) = 4.92 (m, 1H), 3.49 (m, 2H), 2.24-0.85 (m, 40H), 0.61 (s, 3H). **NMR $^{13}$C** (63 MHz, $CDCl_3$) : δ (ppm) = 173.64, 70.90, 70.87, 67.19, 55.74, 54.84, 43.55, 43.39, 42.33, 40.02, 39.15, 37.16, 36.92, 35.09, 34.79, 33.86, 31.50, 30.84, 30.04, 28.46, 26.72, 23.25, 21.64, 21.02, 18.18, 11.93. Yield : 40%

**Methyl lithocholate 194** ($C_{25}H_{42}O_3$). **NMR $^1$H** (250 MHz, $CDCl_3$) : δ (ppm) = 3.64-3.59 (m, 4H), 2.39-0.99 (m, 29H), 0.91 (m, 6H), 0.61 (s, 3H). **NMR $^{13}$C** (63 MHz, $CDCl_3$) : δ (ppm) = 174.51, 71.73, 56.56, 56.11, 51.23, 42.78, 42.22, 40.57, 40.24, 36.58, 35.93, 35.44, 35.31, 34.59, 31.09, 31.05, 30.62. 28.08, 27.23, 26.43, 24.17, 23.31, 20.86, 18.25, 12.01. Yield : 91%

**Isopropyl lithocholate 195** ($C_{27}H_{46}O_3$). **NMR $^1$H** (250 MHz, $CDCl_3$) : δ (ppm) = 4.95 (m, 1H), 3.57 (m, 1H), 2.42-2.07 (m, 3H), 1.94-1.02 (m, 32H), 0.88 (m, 6H), 0.60 (s, 3H). **NMR $^{13}$C** (63 MHz, $CDCl_3$) : δ (ppm) = 173.59, 71.62, 67.15, 56.52, 56.11, 42.74, 42.17, 40.52, 40.21, 36.49, 35.89, 35.41, 35.22, 34.55, 31.66, 31.02, 30.54, 28.04, 27.20, 26.40, 24.12, 23.28, 21.73, 20.81, 18.21, 11.96. Yield : 85%

**Synthesis of *tert*-Butyl deoxycholate 166**

In a round-bottom flask equipped with magnetic stirring, deoxycholic acid 103 (360 mg, 0.92 mmol) was dissolved in tert-butanol (500 µL). Magnesium chloride (18 mg, 0.184 mmol, 0.2

equiv) was added under stirring, followed by di-tert-butyl dicarbonate (275 µL, 1.2 mmol, 1.3 equiv). The reaction mixture was refluxed in tert-butanol for 12 h and then stirred at room temperature for an additional 12 h. The mixture was diluted with EtOAc (15 mL), washed with water (3 × 5 mL), dried over anhydrous $Na_2SO_4$, and filtered. Evaporation of the filtrate under reduced pressure afforded the crude product, which was purified by silica gel column chromatography (eluent: EtOAc/petroleum ether, 1:1) to give the desired product **166** in 26% yield.

***tert*-Butyl deoxycholate 166** ($C_{28}H_{48}O_4$). **NMR $^1$H** (250 MHz, $CDCl_3$) : δ (ppm) = 3.96 (m, 1H), 3.56 (m, 1H), 2.30-0.86 (m, 43H), 0.66 (s, 3H). **NMR $^{13}$C** (63 MHz, $CDCl_3$) : δ (ppm) = 173.64, 79.86, 73.07, 71.64, 48.14, 47.27, 46.41, 42.00, 36.32, 35.95, 35.16, 35.03, 34.05, 33.52, 32.53, 30.89, 30.34, 28.53, 28.05, 27.43, 27.07, 26.06, 23.60, 23.08, 17.23, 12.65

## 2. Oxidation procedure

Ketosteroids were obtained following the same procedure, starting from the appropriate precursor previously prepared according to the esterification protocols described above. As a representative example, the synthesis of 3-oxo-methyl deoxycholate **169** is described below:

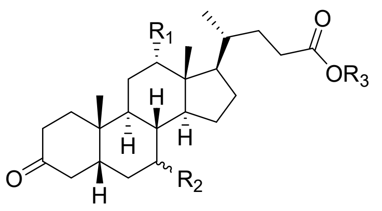


$R_1$ = OH, $R_2$ = H :
$R_3$ = Me : **169**
*i*-Pr : **170**
Et : **171**
Bu : **172**
*t*-Bu : **173**
$CH_2CF_3$ : **174**
$CH(CF_3)_2$ : **175**

$R_1$ = $R_2$ = α-OH :
$R_3$ = Me : **179**
*i*-Pr : **180**
Et : **181**

$R_1$ = H, $R_2$ = α-OH :
$R_3$ = Me : **185**
*i*-Pr : **186**
Et : **187**

$R_1$ = H, $R_2$ = β-OH :
$R_3$ = Me : **192**
*i*-Pr : **193**

$R_1$ = $R_2$ = H :
$R_3$ = Me : **196**
*i*-Pr : **197**

In a 250 mL round-bottom flask equipped with magnetic stirring, methyl deoxycholate 162 (8.1 g, 20 mmol) was dissolved in toluene (100 mL) and acetone (50 mL). Aluminum tri-*tert*-butoxide (10.8 g, 44 mmol, 2.2 equiv) was added, and the reaction mixture was refluxed in toluene for 8–12 h. After cooling to room temperature, aqueous $H_2SO_4$ (2 N, 30 mL) was added under stirring, and the mixture was stirred for an additional 15 min. The reaction mixture was washed with aqueous $H_2SO_4$ (2 N, 3 × 50 mL) and then with water (50 mL). The combined organic layers were dried over anhydrous $Na_2SO_4$, filtered, and concentrated under reduced pressure. The crude product was purified by silica gel chromatography (eluent: EtOAc/petroleum ether, 1:1) to afford the desired compound **169** in 62% yield (non-optimized).

**3-oxo-methyl desoxycholate 169** ($C_{25}H_{40}O_4$). **NMR $^{1}$H** (250 MHz, CDCl3) : δ (ppm) = 4.01 (m, 1H), 3.64 (m, 3H), 2.44-0.94 (m, 33H), 0.69 (s, 3H). **NMR $^{13}$C** (63 MHz, CDCl3) : δ (ppm) = 213.40, 174.62, 72.81, 51.44, 48.01, 47.23, 46.47, 44.18, 42.22, 37.01, 36.77, 35.60, 34.98, 34.30, 33.70, 30.95, 30.77, 28.87, 27.35, 26.44, 25.37, 23.48, 22.30, 17.23, 12.66

**3-oxo-isopropyl desoxycholate 170** ($C_{27}H_{44}O_4$). **NMR $^{1}$H** (250 MHz, $CDCl_3$) : δ (ppm) = 5.00 (m, 1H), 4.04 (m, 1H), 2.46-2.12 (m, 4H), 2.06-0.96 (m, 35H), 0.71 (s, 3H). **NMR $^{13}$C** (63 MHz, $CDCl_3$) : δ (ppm) = 212.74, 173.57, 72.98, 67.34, 48.22, 47.59, 46.70, 44.30, 42.33, 37.04, 36.92, 35.87, 35.05, 34.45, 34.11, 31.76, 31.00, 29.06, 27.40, 26.64, 25.55, 23.58, 22.38, 21.81, 17.42, 12.75. Yield : 77%

**3-oxo-ethyl desoxycholate 171** ($C_{26}H_{42}O_4$). **NMR $^{1}$H** (250 MHz, $CDCl_3$) : δ (ppm) = 4.10 (q, 2H), 3.94 (m, 1H), 2.45-0.87 (m, 36H), 0.68 (s, 3H). **NMR $^{13}$C** (63 MHz, $CDCl_3$) : δ (ppm) = 213.52, 174.20, 72.77, 60.14, 47.97, 47.21, 46.43, 44.15, 42.19, 37.00, 36.74, 35.57, 34.97, 34.27, 33.65, 31.21, 30.74, 28.83, 27.34, 26.42, 25.35, 23.47, 22.27, 17.22, 14.15, 12.64. Yield : 51%

**3-oxo-butyl desoxycholate 172** ($C_{28}H_{46}O_4$). **NMR $^{1}$H** (250 MHz, $CDCl_3$) : δ (ppm) = 4.09-3.99 (m, 3H), 2.38-1.06 (m, 31H), 0.96-0.85 (m, 9H), 0.67 (s, 3H). **NMR $^{13}$C** (63 MHz, $CDCl_3$) : δ (ppm) = 213.58, 174.28, 72.74, 64.03, 47.89, 47.12, 46.39, 44.11, 42.14, 36.93, 36.70, 35.52, 34.95, 34.23, 33.58, 31.17, 30.74, 30.52, 28.78, 27.30, 26.38, 25.31, 23.45, 22.22, 18.99, 17.14, 13.58, 12.59. Yield : 80%

**3-oxo-désoxycholate de *tert*-butyle 173** ($C_{28}H_{46}O_4$). **NMR $^{1}$H** (250 MHz, $CDCl_3$) : δ (ppm) = 4.03 (m, 1H), 2.47-0.86 (m, 42H), 0.70 (s, 3H). **NMR $^{13}$C** (63 MHz, $CDCl_3$) : δ (ppm) = 213.45, 173.57, 79.92, 72.89, 48.03, 47.38, 46.49, 44.21, 42.25, 37.05, 36.79, 35.63, 34.91, 34.32, 33.73, 32.48, 30.84, 28.82, 28.04, 27.38, 26.47, 25.39, 23.50, 22.33, 17.31, 12.68. Yield : 40%

**3-oxo-trifluoroethyl desoxycholate174** ($C_{26}H_{39}F_3O_4$). **NMR $^{1}$H** (250 MHz, $CDCl_3$) : δ (ppm) = 4.01 (m, 1H), 3.64 (m, 2H), 2.76-0.87 (m, 33H), 0.70 (s, 3H). **NMR $^{13}$C** (63 MHz, $CDCl_3$) : δ (ppm) = 212.79, 174.46, 72.92, 51.31, 48.17, 47.46, 46.65, 42.29, 37.01, 36.88, 35.82, 35.05, 34.41, 34.05, 31.09, 30.94, 29.05, 27.38, 26.60, 25.50, 23.56, 22.34, 17.37, 12.72. Yield : 42%

**3-oxo-hexafluoroisopropyl desoxycholate 175** ($C_{27}H_{38}F_6O_4$). **NMR $^{1}$H** (250 MHz, $CDCl_3$) : δ (ppm) = 3.99 (m, 1H), 3.62 (m, 2H), 2.74-2.07 (m, 5H), 2.00-0.88 (m, 27H), 0.67 (s, 3H). **NMR $^{13}$C** (63 MHz, $CDCl_3$) : δ (ppm) = 212.93, 174.72, 72.86, 51.25, 48.07, 47.35, 46.58,

44.20, 42.21, 36.93, 36.82, 35.76, 35.01, 34.35, 33.96, 31.03, 30.88, 28.96, 27.32, 26.54, 25.45, 23.52, 22.27, 17.28, 12.66. Yield : 25%

**3-oxo-methyl cholate 179** ($C_{25}H_{40}O_5$). **NMR $^1$H** (250 MHz, $CDCl_3$) : δ (ppm) = 3.92 (m, 1H), 3.52-0.64 (m, 39H). **NMR $^{13}$C** (63 MHz, $CDCl_3$) : δ (ppm) = 213.74, 174.84, 72.96, 68.26, 51.54, 47.21, 46.56, 45.53, 43.04, 41.63, 39.37, 36.75, 36.64, 35.31, 34.94, 34.93, 31.11, 30.62, 28.62, 27.49, 26.89, 23.21, 21.58, 17.30, 12.51. Yield : 54%

**3-oxo-isopropyl cholate 180** ($C_{27}H_{44}O_5$). **NMR $^1$H** (250 MHz, $CDCl_3$) : δ (ppm) = 4.92 (m, 1H), 4.05-3.78 (m, 2H), 3.36-3.18 (m, 4H), 2.26-0.81 (m, 36H), 0.60 (m, 3H). **NMR $^{13}$C** (63 MHz, $CDCl_3$) : δ (ppm) = 173.98, 73.11, 71.88, 68.47, 67.38, 47.06, 46.44, 41.59, 39.50, 35.31, 34.81, 31.76, 30.97, 30.40, 28.15, 2755, 26.30, 25.85, 23.36, 22.47, 21.89, 17.34, 12.48. Yield : 68%

**3-oxo-methyl chenodesoxycholate 185** ($C_{25}H_{40}O_4$). **NMR $^1$H** (250 MHz, $CDCl_3$) : δ (ppm) = 4.00 (m, 1H), 3.75 (s, 3H), 2.26-1.02 (m, 33H), 0.79 (s, 3H). **NMR $^{13}$C** (63 MHz, $CDCl_3$) : δ (ppm) = 213.33, 174.63, 68.18, 55.68, 51.38, 50.17, 45.51, 43.12, 42.57, 39.40, 39.24, 36.83, 36.71, 35.23, 35.19, 33.80, 33.13, 30.85, 28.03, 23.53, 21.82, 20.86, 18.15, 11.67. Yield : 75%

**3-oxo-isopropyl chenodesoxycholate 186** ($C_{27}H_{44}O_4$). **NMR $^1$H** (250 MHz, $CDCl_3$) : δ (ppm) = 4.96 (m, 1H), 3.89 (m, 1H), 2.42-0.67 (m, 39H), 0.67 (s, 3H). **NMR $^{13}$C** (63 MHz, $CDCl_3$) : δ (ppm) = 212.65, 173.55, 68.30, 67.23, 56.02, 50.35, 45.59, 43.18, 42.76, 39.63, 39.53, 36.83, 35.30, 35.25, 34.01, 33.57, 31.66, 31.04, 28.02, 23.63, 21.87, 21.76, 21.02, 18.26, 11.75. Yield : 66%

**3-oxo-ethyl chenodesoxycholate 187** ($C_{26}H_{42}O_4$). **NMR $^1$H** (250 MHz, $CDCl_3$) : δ (ppm) = 4.07 (q, *J* = 7.1 Hz, 2H), 3.85 (m, 1H), 2.38-0.84 (m, 36H), 0.69 (s, 3H). **NMR $^{13}$C** (63 MHz, $CDCl_3$) : δ (ppm) = 212.69, 174.06, 68.42, 60.08, 56.03, 50.40, 45.62, 43.20, 42.81, 39.67, 39.56, 36.86, 35.33, 34.04, 33.61, 31.37, 31.05, 28.06, 23.69, 21.91, 21.05, 18.31, 14.21, 11.80. Yield : 56%

**3-oxo-methyl ursodesoxycholate 192** ($C_{25}H_{40}O_4$). **NMR $^1$H** (250 MHz, $CDCl_3$) : δ (ppm) = 3.62-3.51 (m, 4H), 2.54-1.01 (m, 30H), 0.89 (d, *J* = 6.3 Hz, 3H), 0.67 (s, 3H). **NMR $^{13}$C** (63 MHz, $CDCl_3$) : δ (ppm) = 211.62, 174.15, 69.93, 55.33, 54.49, 51.03, 44.03, 43.22, 42.76, 42.66, 39.56, 38.89, 36.54, 36.11, 35.96, 34.77, 33.94, 30.52, 28.12, 26.37, 22.22, 21.24, 17.96, 11.74. Yield : 37%

**3-oxo-isopropyl ursodesoxycholate 193** ($C_{27}H_{44}O_4$). **NMR $^1$H** (250 MHz, $CDCl_3$) : δ (ppm) = 4.86 (m, 1H), 3.47 (m, 1H), 2.47-0.80 (m, 39H), 0.59 (s, 3H). **NMR $^{13}$C** (63 MHz, $CDCl_3$) : δ (ppm) = 211.89, 173.45, 70.17, 67.05, 55.45, 54.64, 44.13, 43.37, 42.90, 42.78, 39.69, 39.05, 36.67, 36.12, 36.07, 34.86, 34.06, 31.28, 30.67, 28.23, 26.48, 22.32, 21.52, 21.35, 18.07, 11.84. Yield : 43%

**3-oxo-methyl lithocholate 196** ($C_{25}H_{40}O_3$). **NMR $^1$H** (250 MHz, $CDCl_3$) : δ (ppm) = 3.65 (s, 3H), 2.41-1.05 (m, 28H), 1.01 (s, 3H), 0.92 (m, 3H), 0.67 (s, 3H). **NMR $^{13}$C** (63 MHz, $CDCl_3$) : δ (ppm) = 213.39, 174.67, 56.34, 55.87, 51.44, 44.26, 42.69, 42.30, 40.62, 39.96, 37.15, 36.95, 35.44, 35.27, 34.81, 30.96, 30.89, 28.09, 26.54, 25.69, 24.09, 22.59, 21.11, 18.20, 11.99. Yield : 36%

**3-oxo-isopropyl lithocholate 197** ($C_{27}H_{44}O_3$). **NMR $^1$H** (250 MHz, $CDCl_3$) : δ (ppm) = 4.94 (m, 1H), 2.35-0.86 (m, 40H), 0.63 (s, 3H). **NMR $^{13}$C** (63 MHz, $CDCl_3$) : δ (ppm) = 212.41, 173.40, 67.11, 56.44, 56.11, 44.21, 42.76, 42.22, 40.91, 40.08, 37.01, 36.94, 35.57, 35.19, 34.83, 31.61, 30.99, 27.99, 26.62, 25.73, 24.07, 22.52, 21.72, 21.17, 18.21, 11.98. Yield : 65%

## 3. Titatium reductive amination procedure

Claramines (A, B, C, D, and E) were synthesized from the corresponding ketone intermediates in the presence of the appropriate polyamine, following the procedure (synthesis and purification) described for the preparation of claramine A1. All resulting products were obtained as colorless to yellow oils that gradually solidified upon standing.

In a 100 mL round-bottom flask equipped with a magnetic stir bar, 3-oxo-isopropyl deoxycholate **170** (3.5 g, 8.0 mmol) was dissolved in methanol (100 mL). Titanium tetra(isopropoxide) (9.5 mL, 32 mmol, 4 equiv) was added, followed by spermine (4.9 g, 24 mmol, 3 equiv). The reaction mixture was stirred at room temperature for 24 h, then cooled in an ice bath, and $NaBH_4$ (1.2 g, 24 mmol, 4 equiv) was added at 0 °C. Stirring was continued for 12 h, after which water (4.6 mL) was added at room temperature to quench the reaction. The mixture was stirred for an additional 12 h, filtered through Celite, washed with aqueous ammonia and methanol, and concentrated under reduced pressure. The crude product was purified by silica gel column chromatography (eluent: $CH_2Cl_2$/MeOH/32% aqueous $NH_3$, 7:3:1) to afford Claramine A01 as a pale yellow oil in 52% yield.

**3β-spermino-isopropyl desoxycholate A01** ($C_{37}H_{70}N_4O_3$)

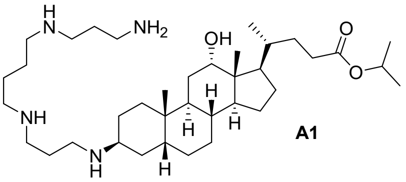

A1

**NMR $^1$H** (250 MHz, $CD_3OD$) : δ (ppm) = 4.96 (m, 1H), 3.94 (m, 1H), 2.88-2.46 (m, 13H), 2.39-2.18 (m, 2H), 2.04-1.12 (m, 40H), 1.01-0.95 (m, 7H), 0.71 (s, 3H). **NMR $^{13}$C** (63 MHz, $CD_3OD$) : δ (ppm) = 175.66, 74.15, 68.95, 59.06, 50.74, 50.70, 49.30, 48.97, 48.42, 48.27, 47.77, 45.83, 44.08, 40.78, 37.58, 37.14, 36.82, 35.97, 35.92, 34.94, 34.59, 33.56, 32.72, 32.45, 30.52, 29.98, 28.84, 28.65, 28.40, 28.29, 27.64, 25.04, 24.10, 22.27, 17.71, 13.37. **MS** ($ESI^+$) : m/z 619.5519 ($[M+H]^+$)

- **Claramines A**

**3β-norspermino-isopropyl desoxycholate A2** ($C_{36}H_{68}N_4O_3$)

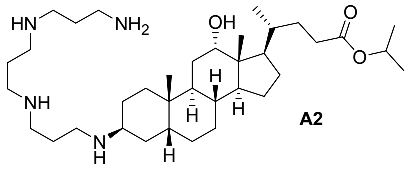

A2

**NMR $^1$H** (250 MHz, $CD_3OD$) : δ (ppm) = 4.96 (m, 1H), 3.95 (m, 1H), 2.79-2.66 (m, 13H), 2.53-0.96 (m, 50H), 0.71 (s, 3H). **NMR $^{13}$C** (63 MHz, $CD_3OD$) : δ (ppm) = 175.76, 74.10, 69.00, 59.10, 48.95, 48.42, 47.86, 45.85, 44.04, 40.75, 37.63, 36.95, 36.79, 36.74, 35.83, 35.10, 32.88, 32.49, 32.29, 30.04, 29.70, 29.27, 28.72, 28.51, 27.76, 27.56, 24.96, 23.90, 22.22, 17.79, 13.35. **MS** ($ESI^+$) : m/z 605.5364 ($[M+H]^+$). Yield : 47%

Mélange 50/50 de **3β-spermidino-isopropyl desoxycholate A3a** et de **3β-*N*-[4'*N*-(3'aminopropyl)aminobutyl]amino-isopropyl desoxycholate A3b** ($C_{34}H_{63}N_3O_3$)

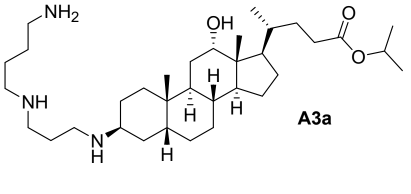

A3a

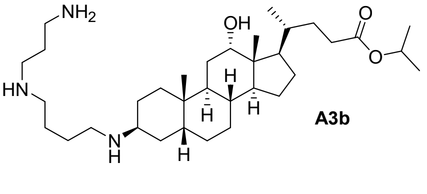

A3b

**NMR $^1$H** (250 MHz, $CD_3OD$) : δ (ppm) = 4.96 (m, 1H), 3.96 (m, 1H), 2.88-2.73 (m, 9H), 2.50-0.93 (m, 49H), 0.71 (s, 3H). **NMR $^{13}$C** (63 MHz, $CD_3OD$) : δ (ppm) = 175.76, 74.18, 74.07, 69.01, 59.10, 49.41, 48.64, 48.38, 46.17, 45.31, 43.87, 41.29, 40.30, 37.59, 37.42, 36.74, 36.62, 65.73, 35.04, 34.17, 32.88, 32.49, 30.01, 28.87, 28.79, 28.73, 28.39, 27.53, 27.21, 27.05, 26.61, 25.05, 24.96, 23.77, 22.22, 18.11, 17.81, 13.42, 13.33. Yield : 32%

**3β-norspermidino-isopropyl desoxycholate A4**

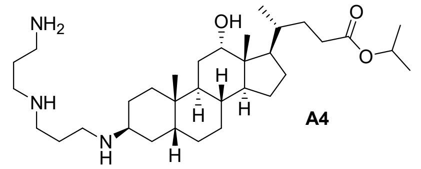


($C_{33}H_{61}N_3O_3$)

**NMR $^{1}$H** (250 MHz, $CD_3OD$) : δ (ppm) = 4.96 (m, 1H), 3.95 (m, 1H), 2.91-2.67 (m, 8H), 2.39-0.96 (m, 48H), 0.71 (s, 3H). **NMR $^{13}$C** (63 MHz, $CD_3OD$) : δ (ppm) = 175.77, 74.10, 69.01, 59.04, 47.86, 45.83, 45.79, 45.72, 43.96, 40.70, 37.60, 36.84, 36.79, 36.74, 35.79, 35.09, 32.88, 32.48, 30.03, 28.72, 28.48, 28.45, 27.53, 24.95, 23.84, 22.21, 17.78, 13.33. **MS** ($ESI^+$) : m/z 548.4786 ($[M+H]^+$). Yield : 37%

**3β-spermino-methyl desoxycholate A5**

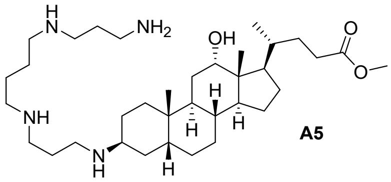


($C_{35}H_{66}N_4O_3$)

**NMR $^{1}$H** (250 MHz, $CD_3OD$) : δ (ppm) = 3.94 (m, 1H), 3.65 (s, 3H), 2.91-3.62 (m, 13H), 2.39-0.96 (m, 46H), 0.71 (s, 3H). **NMR $^{13}$C** (63 MHz, $CD_3OD$) : δ (ppm) = 176.53, 74.09, 59.04, 52.18, 50.57, 49.29, 48.32, 47.74, 45.74, 44.01, 40.69, 37.55, 37.06, 36.85, 35.87, 34.93, 34.33, 33.01, 32.36, 32.00, 30.06, 29.98, 28.81, 28.62, 28.27, 28.05, 27.63, 25.04, 24.08, 17.74, 13.37. **MS** ($ESI^+$) : m/z 591.6 ($[M+H]^+$). Yield : 30%

**3β-norspermino-methyl desoxycholate A6**

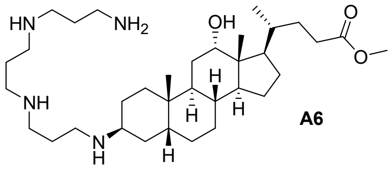


($C_{34}H_{64}N_4O_3$)

**NMR $^{1}$H** (250 MHz, $CD_3OD$) : δ (ppm) = 3.94 (m, 1H), 3.65 (s, 3H), 2.95-2.63 (m, 10H), 2.44-0.92 (m, 47H), 0.71 (s, 3H). **NMR $^{13}$C** (63 MHz, $CD_3OD$) : δ (ppm) = 176.62, 74.08, 59.15, 52.16, 49.36, 48.30, 48.24, 47.75, 43.93, 37.55, 36.87, 35.80, 34.98, 32.38, 32.01, 29.97, 28.80, 28.49, 27.57, 27.48, 24.99, 23.93, 22.23, 17.70, 13.31. **MS** ($ESI^+$) : m/z 577.6 ($[M+H]^+$). Yield : 40%

Mélange 50/50 de **3β-spermidino-methyl desoxycholate A7a** et de **3β-*N*-[4'*N*-(3'aminopropyl)aminobutyl]amino-methyl desoxycholate A7b** ($C_{32}H_{59}N_3O_3$)

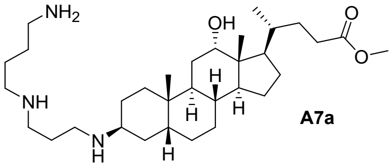
A7a

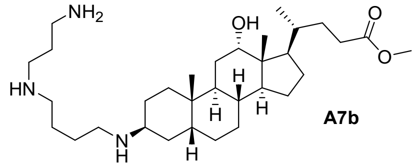
A7b

**NMR $^1$H** (250 MHz, $CD_3OD$) : δ (ppm) = 3.96 (m, 1H), 3.65 (s, 3H), 2.90-2.61 (m, 9H), 2.38-2.14 (m, 3H), 1.84-0.95 (m, 40H), 0.71 (s, 3H). **NMR $^{13}$C** (63 MHz, $CD_3OD$) : δ (ppm) = 176.50, 74.08, 59.04, 59.00, 52.18, 50.53, 49.99, 49.28, 48.31, 47.73, 47.38, 45.77, 44.01, 42.38, 40.71, 37.55, 37.04, 36.84, 35.87, 34.92, 34.33, 33.15, 32.35, 31.99, 31.36, 30.21, 29.98, 28.81, 28.62, 28.43, 28.34, 28.13, 27.88, 27.61, 25.04, 24.09, 17.74, 13.39. Yield : 32%

**3β-norspermidino-methyl desoxycholateA8**
($C_{31}H_{57}N_3O_3$)

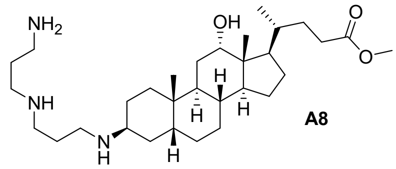
A8

**NMR $^1$H** (250 MHz, $CD_3OD$) : δ (ppm) = 3.94 (m, 1H), 3.65 (s, 3H), 2.95-2.60 (m, 9H), 2.44-0.95 (m, 41H), 0.71 (s, 3H). **NMR $^{13}$C** (63 MHz, $CD_3OD$) : δ (ppm) = 176.59, 74.11, 59.04, 52.16, 49.35, 48.92, 48.31, 47.74, 45.74, 43.99, 40.61, 37.55, 36.86, 35.84, 34.95, 32.64, 32.36, 32.00, 29.97, 28.79, 28.56, 27.59, 25.01, 23.98, 17.71, 13.31. **MS** ($ESI^+$) : m/z 520.5 ($[M+H]^+$). Yield : 55%

**3β-(éthylènediamine)-methyl desoxycholate A9**
($C_{27}H_{48}N_2O_3$)

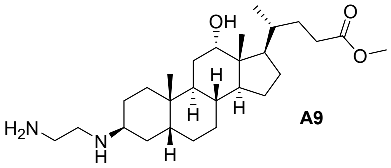
A9

**NMR $^1$H** (250 MHz, $CD_3OD$) : δ (ppm) = 3.95 (m, 1H), 3.65 (s, 3H), 2.97-2.60 (m, 5H), 2.44-0.95 (m, 36H), 0.71 (s, 3H). **NMR $^{13}$C** (63 MHz, $CD_3OD$) : δ (ppm) = 176.54, 74.07, 59.04, 52.18, 49.30, 48.64, 47.73, 43.95, 41.39, 37.55, 37.01, 36.94, 36.85, 35.83, 34.94, 34.32, 32.34, 32.00, 29.98, 28.80, 28.55, 27.61, 25.03, 24.02, 22.27, 17.71, 13.36. **MS** ($ESI^+$) : m/z 449.4 ($[M+H]^+$). Yield : 80%

**3β-(1,3-diaminopropane)-methyl desoxycholate A10**
($C_{28}H_{50}N_2O_3$)

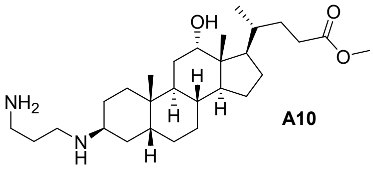
A10

**NMR $^1$H** (250 MHz, $CD_3OD$) : δ (ppm) = 3.95 (m, 1H), 3.65 (s, 3H), 2.92-2.53 (m, 5H), 2.38-0.95 (m, 38H), 0.71 (s, 3H). **NMR $^{13}$C** (63 MHz, $CD_3OD$) : δ (ppm) = 176.55, 74.08 59.06, 52.17, 49.33, 48.18, 45.15, 43.96, 40.57, 37.63, 37.53, 36.95, 36.85, 35.82, 34.94, 32.36, 32.01, 29.98, 28.79, 28.53, 27.67, 27.59, 25.02, 24.00, 22.27, 17.72, 13.35. **MS** (ESI$^+$) : m/z 463.4 ([M+H]$^+$). Yield : 45%

**3β-(1,4-diaminobutane)-methyl desoxycholate A11**

($C_{29}H_{52}N_2O_3$)

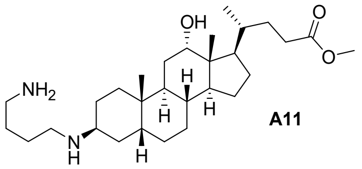

A11

**NMR $^1$H** (250 MHz, $CD_3OD$) : δ (ppm) = 3.93-3.65 (m, 4H), 2.90-2.48 (m, 5H), 2.38-2.21 (m, 2H), 1.93-0.93 (m, 38H), 0.71 (s, 3H). **NMR $^{13}$C** (63 MHz, $CD_3OD$) : δ (ppm) = 176.58, 74.13, 59.00, 52.16, 49.65, 49.32, 48.18, 47.74, 47.31, 46.56, 44.02, 42.34, 36.85, 36.50, 36.37, 35.87, 34.94, 34.30, 32.36, 32.01, 28.81, 28.05, 25.03, 24.04, 22.26, 17.71, 13.35. **MS** (ESI$^+$) : m/z 477.4011 ([M+H]$^+$). Yield : 35%

**3β-(1,5-diaminopentane)-methyl desoxycholate A12**

($C_{30}H_{54}N_2O_3$)

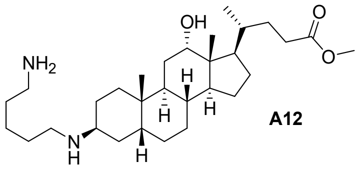

A12

**NMR $^1$H** (250 MHz, $CD_3OD$) : δ (ppm) = 3.94-3.65 (m, 4H), 2.91-2.54 (m, 5H), 2.44-2.19 (m, 2H), 1.97-0.95 (m, 40H), 0.71 (s, 3H). **NMR $^{13}$C** (63 MHz, $CD_3OD$) : δ (ppm) = 176.51, 74.10, 59.09, 52.16, 49.30, 48.17, 47.74, 47.44, 44.02, 42.38, 37.55, 37.05, 36.85, 35.87, 34.94, 33.28, 32.36, 31.99, 30.33, 29.98, 28.80, 28.60, 27.97, 25.82, 25.02, 24.06, 22.27, 17.73, 13.37. **MS** (ESI$^+$) : m/z 491.4 ([M+H]$^+$). Yield : 65%

**3β-(1,6-diaminohexane)-methyl desoxycholate A13**

($C_{31}H_{56}N_2O_3$)

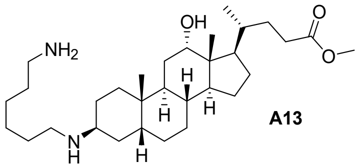

A13

**NMR $^1$H** (250 MHz, $CD_3OD$) : δ (ppm) = 3.95 (m, 1H), 3.65 (s, 3H), 2.92-2.55 (m, 5H), 2.42-2.19 (m, 2H), 1.92-0.95 (m, 42H), 0.71 (s, 3H). **NMR $^{13}$C** (63 MHz, $CD_3OD$) : δ (ppm) = 176.59, 74.14, 59.13, 52.07, 49.39, 48.32, 47.84, 47.38, 44.12, 42.41, 37.63, 37.39, 37.06, 36.79, 35.88, 35.07, 32.42, 32.15, 30.28, 30.03, 28.70, 28.56, 28.29, 27.88, 27.59, 24.98, 23.95, 22.22, 17.79, 13.36. **MS** (ESI$^+$) : m/z 505.4 ([M+H]$^+$). Yield : 41%

**3β-(tétraéthylènepentamine)-methyl desoxycholate A14** ($C_{33}H_{63}N_5O_3$)

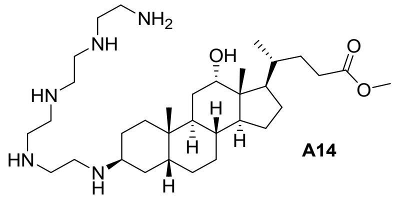


**NMR $^1$H** (250 MHz, $CD_3OD$) : δ (ppm) = 3.94 (m, 1H), 3.65 (s, 3H), 2.99-2.42 (m, 19H), 2.38-2.21(m, 2H), 1.98-0.95 (m, 35H), 0.71 (s, 3H). **NMR $^{13}$C** (63 MHz, $CD_3OD$) : δ (ppm) = 176.56, 74.05, 59.12, 54.25, 54.15, 52.18, 49.68, 49.33, 48.18, 47.74, 46.51, 43.95, 41.59, 37.55, 36.97, 36.85, 35.84, 34.96, 32.36, 32.01, 29.99, 28.79, 28.56, 27.61, 25.02, 24.01, 22.26, 17.71, 13.35. **MS** ($ESI^+$) : m/z 578.4932 ($[M+H]^+$). Yield : 60%

**3β-(pentaethylenehexamine)-methyl desoxycholate A15** ($C_{35}H_{68}N_6O_3$)

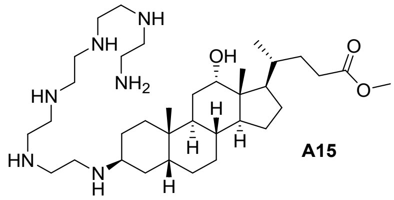


**NMR $^1$H** (250 MHz, $CD_3OD$) : δ (ppm) = 3.95 (m, 1H), 3.65 (s, 3H), 2.84-2.54 (m, 24H), 2.38-2.25 (m, 2H), 1.93-0.96 (m, 35H), 0.71 (s, 3H). **NMR $^{13}$C** (63 MHz, $CD_3OD$) : δ (ppm) = 176.61, 74.06, 59.08, 54.26, 54.15, 52.16, 49.34, 48.19, 47.74, 46.28, 43.95, 41.46, 37.55, 36.85, 35.82, 34.97, 32.36, 32.01, 29.97, 28.78, 28.51, 27.63, 27.59, 25.01, 23.96, 22.23, 17.70, 13.31. **MS** ($ESI^+$) : m/z 621.5385 ($[M+H]^+$). Yield : 25%

**3β-(Bis(3-aminopropyl)methylamine)-désoxycholate de méthyle A16** ($C_{32}H_{59}N_3O_3$)

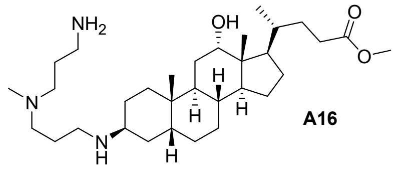


**NMR $^1$H** (250 MHz, $CD_3OD$) : δ (ppm) = 3.93 (m, 1H), 3.65 (s, 3H), 2.88-2.40 (m, 12H), 2.32-2.18 (m, 6H), 2.03-0.95 (m, 34H), 0.71 (s, 3H). **NMR $^{13}$C** (63 MHz, $CD_3OD$) : δ (ppm) = 176.53, 74.13, 59.12, 57.28, 56.74, 52.03, 49.40, 48.35, 47.86, 46.22, 44.19, 42.50, 41.23, 37.67, 37.43, 37.22, 36.76, 35.94, 35.11, 32.42, 32.18, 31.12, 30.06, 28.67, 28.13, 27.61, 24.99, 24.03, 22.24, 17.83, 13.38. **MS** ($ESI^+$) : m/z 534.5 ($[M+H]^+$). Yield : 64%

**3β-(Tris(3-aminopropyl)amine)-désoxycholate de méthyle A17** ($C_{34}H_{64}N_4O_3$)

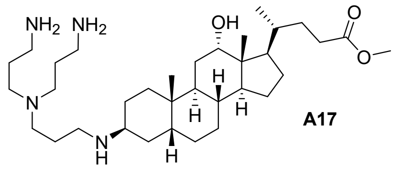

**NMR $^{1}$H** (250 MHz, $CD_3OD$) : δ (ppm) = 3.95 (m, 1H), 3.65 (s, 3H), 2.79-2.54 (m, 13H), 2.38-2.21 (m, 2H), 1.98-0.96 (m, 42H), 0.71 (s, 3H). **NMR $^{13}$C** (63 MHz, $CD_3OD$) : δ (ppm) = 176.59, 74.05, 59.16, 53.54, 53.41, 53.05, 52.92, 52.07, 49.40, 48.35, 47.84, 45.74, 43.91, 43.87, 40.83, 40.76, 37.58, 37.39, 36.79, 35.75, 35.04, 32.40, 32.15, 30.02, 28.69, 28.40, 27.53, 24.96, 23.82, 22.22, 17.79, 13.34. **MS** ($ESI^+$) : m/z 577.5014 ($[M+H]^+$). Yield : 47%

**3β-(1,4-Bis(3-aminopropyl)piperazine)-methyl desoxycholate A18** ($C_{35}H_{64}N_4O_3$)

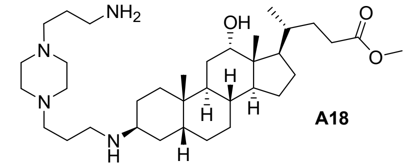


**NMR $^{1}$H** (250 MHz, $CD_3OD$) : δ (ppm) = 3.94 (m 1H), 3.65 (s, 3H), 2.80-0.96 (m, 57H), 0.71 (s, 3H). **NMR $^{13}$C** (63 MHz, $CD_3OD$) : δ (ppm) = 176.50, 73.96, 58.93, 57.79, 57.35, 54.13, 53.96, 52.18, 49.33, 47.73, 46.01, 43.82, 40.86, 37.52, 36.84, 35.76, 34.93, 32.35, 31.99, 30.00, 28.89, 28.86, 28.82, 28.48, 27.57, 27.50, 27.36, 26.20, 25.03, 25.00, 23.95, 22.27, 17.72, 13.35. **MS** ($ESI^+$) : m/z 589.5051 ($[M+H]^+$). Yield : 76%

**3β-(cyclohexylamine)-methyl desoxycholate A19** ($C_{31}H_{53}NO_3$)

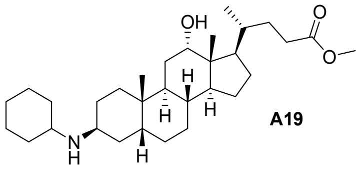


**NMR $^{1}$H** (250 MHz, $CD_3OD$) : δ (ppm) = 3.94 (m 1H), 3.65 (s, 3H), 2.77-2.65 (m, 2H), 2.38-0.95 (m, 44H), 0.71 (s, 3H). **NMR $^{13}$C** (63 MHz, $CD_3OD$) : δ (ppm) = 176.52, 74.08, 54.82, 54.05, 52.18, 50.17, 49.31, 44.08, 37.54, 37.14, 36.86, 35.85, 34.92, 34.52, 33.87, 33.81, 32.35, 32.00, 29.95, 28.81, 28.61, 28.29, 27.62, 27.22, 26.39, 25.05, 24.06, 17.74, 13.40. **MS** ($ESI^+$) : m/z 488.4 ($[M+H]^+$). Yield : 63%

**3β-spermino-ethyl desoxycholate A20** ($C_{36}H_{68}N_4O_3$)

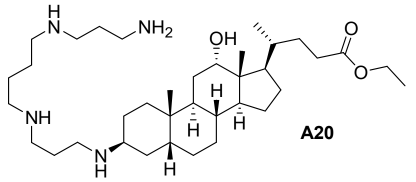


**NMR $^{1}$H** (250 MHz, $CD_3OD$) : δ (ppm) = 4.11 (q, 2H), 3.94 (m, 1H), 2.94-2.65 (m, 15H), 2.40-2.21 (m, 3H), 1.98-1.13 (m, 37H), 1.01-0.95 (m, 7H), 0.71 (s, 3H). **NMR $^{13}$C** (63 MHz, $CD_3OD$) : δ (ppm) = 176.23, 74.15, 61.48, 59.09, 52.07, 50.36, 48.35, 47.86, 45.83, 44.11, 40.81, 37.65, 37.01, 36.79, 35.87, 35.10, 32.61, 32.45, 32.17, 30.04, 29.61, 28.70, 28.54, 28.16, 28.04, 27.58, 24.97, 23.92, 17.78, 14.67, 13.32. **MS** ($ESI^+$) : m/z 605.5 ($[M+H]^+$). Yield : 19%

**3β-spermino-butyl desoxycholate A21**
($C_{38}H_{72}N_4O_3$)

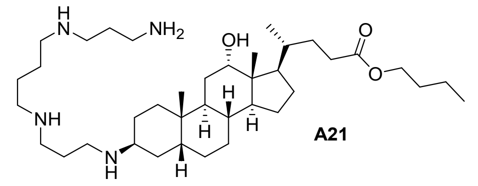


**NMR $^{1}$H** (250 MHz, $CD_3OD$) : δ (ppm) = 4.07 (t, 2H), 3.94 (m, 1H), 2.94-2.65 (m, 15H), 2.36-2.21 (m, 2H), 1.84-0.95 (m, 49H), 0.71 (s, 3H). **NMR $^{13}$C** (63 MHz, $CD_3OD$) : δ (ppm) = 176.56, 74.09, 65.36, 59.06, 52.08, 50.33, 49.36, 48.30, 47.83, 45.80, 44.08, 40.75, 37.62, 37.44, 37.38, 37.07, 36.98, 36.77, 35.87, 35.04, 32.50, 32.41, 32.15, 32.01, 30.22, 30.03, 29.69, 28.71, 28.57, 28.11, 27.59, 24.99, 23.98, 20.29, 17.80, 14.10, 13.37. **MS** ($ESI^+$) : m/z 633.5638 ($[M+H]^+$). Yield : 51%

**3β-spermino-désoxycholate de *t*-butyle A22**
($C_{38}H_{72}N_4O_3$)

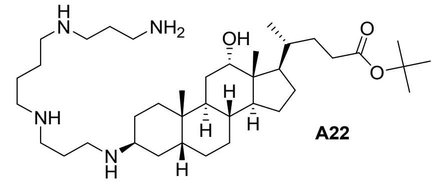


**NMR $^{1}$H** (250 MHz, $CD_3OD$) : δ (ppm) = 3.94 (m, 1H), 2.90-2.53 (m, 13H), 2.34-0.95 (m, 55H), 0.71 (s, 3H). **NMR $^{13}$C** (63 MHz, $CD_3OD$) : δ (ppm) = 175.66, 81.38, 74.14, 59.04, 50.62, 50.57, 49.33, 48.89, 48.35, 48.31, 47.77, 45.77, 44.05, 40.72, 37.58, 37.08, 36.79, 35.89, 34.95, 34.39, 33.72, 33.14, 32.55, 30.16, 29.98, 28.86, 28.54, 28.30, 28.12, 27.63, 25.03, 24.06, 17.69, 13.36. **MS** ($ESI^+$) : m/z 633.7 ($[M+H]^+$). Yield : 39%

**3β-spermino-trifluoroethyl desoxycholate A24**
($C_{36}H_{65}F_3N_4O_3$)

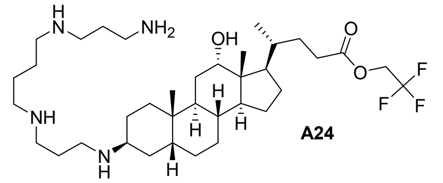


**NMR $^{1}$H** (250 MHz, $CD_3OD$) : δ (ppm) = 3.97 (m, 1H), 3.65-3.56 (m, 2H), 3.18-2.78 (m, 10H), 2.38-0.95 (m, 49H), 0.69 (s, 3H). **NMR $^{13}$C** (63 MHz, $CD_3OD$) : δ (ppm) = 175.74, 72.57, 69.03, 62.84, 58.11, 58.06, 57.83, 57.60, 51.86, 44.88, 44.06, 43.69, 42.03, 41.67, 37.39, 37.33, 36.79, 36.63, 35.84, 33.98, 32.71, 32.45, 31.35, 29.49, 29.39, 28.51, 25.40, 24.09, 22.22, 19.13, 18.87, 12.62. **MS** ($ESI^+$) : m/z 659.5040 ($[M+H]^+$). Yield : 15%

**3β-spermino-hexafluoroisopropyl desoxycholate A25** ($C_{37}H_{64}F_6N_4O_3$)

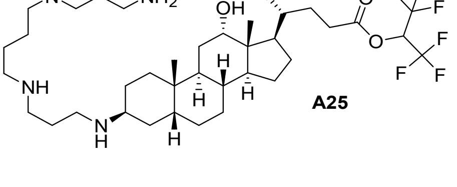

**NMR $^1$H** (250 MHz, $CD_3OD$) : δ (ppm) = 3.95 (m, 1H), 3.65 (m, 1H), 2.96-2.59 (m, 11H), 2.45-2.21 (m, 3H), 2.13-0.95 (m, 45H), 0.71 (s, 3H). **NMR $^{13}$C** (63 MHz, $CD_3OD$) : δ (ppm) = 176.64, 74.13, 59.06, 52.15, 50.46, 50.39, 49.38, 48.24, 47.76, 45.68, 44.01, 40.62, 37.58, 36.87, 35.85, 35.00, 34.40, 33.61, 32.37, 32.03, 30.23, .30.00, 28.81, 28.13, 27.62, 25.01, 23.98, 17.70, 13.32. **MS** ($ESI^+$) : m/z 727.4915 ($[M+H]^+$). Yield : 30%

**3β-(1,4-Bis(3-aminopropyl)piperazine)-isopropyl desoxycholate A26** ($C_{37}H_{68}N_4O_3$)

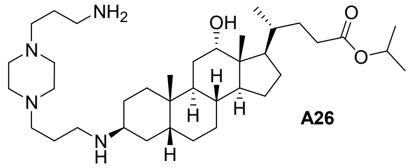

A26

**NMR $^1$H** (250 MHz, $CD_3OD$) : δ (ppm) = 4.96 (m, 1H), 3.93 (m, 1H), 2.84-2.39 (m, 17H), 2.33-0.95 (m, 46H), 0.71 (s, 3H). **NMR $^{13}$C** (63 MHz, $CD_3OD$) : δ (ppm) = 175.61, 74.08, 68.92, 59.04, 58.02, 57.48, 54.10, 54.02, 49.30, 48.24, 47.76, 46.28, 44.03, 41.15, 37.58, 37.13, 36.82, 35.91, 34.92, 34.64, 34.30, 32.71, 32.44, 30.52, 30.00, 28.85, 28.65, 28.32, 27.65, 27.39, 25.05, 24.13, 22.30, 17.72, 13.40. **MS** ($ESI^+$) : m/z 617.5 ($[M+H]^+$). Yield : 58%

**3β-(pentaethylenehexamine)-isopropyl desoxycholate A27** ($C_{37}H_{72}N_6O_3$)

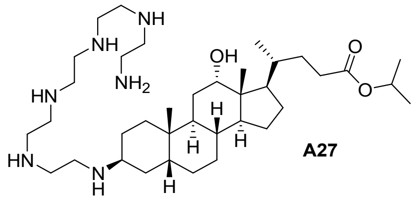

A27

**NMR $^1$H** (250 MHz, $CD_3OD$) : δ (ppm) = 4.96 (m, 1H), 3.94 (m, 1H), 2.84-2.47 (m, 22H), 2.33-2.21 (m, 2H), 1.98-0.88 (m, 43H), 0.71 (s, 3H). **NMR $^{13}$C** (63 MHz, $CD_3OD$) : δ (ppm) = 175.74, 74.12, 68.98, 59.13, 58.59, 55.32, 55.14, 54.50, 54.29, 54.05, 49.63, 49.34, 48.29, 47.77, 44.04, 37.58, 37.03, 36.81, 35.88, 34.97, 32.79, 32.74, 32.45, 29.99, 28.82, 28.59, 28.22, 27.62, 25.01, 24.86, 24.03, 22.24, 17.68, 13.34. **MS** ($ESI^+$) : m/z 649.5601 ($[M+H]^+$). Yield : 24%

**3β-(Tris(3-aminopropyl)amine)-isopropyl desoxycholate A28** ($C_{36}H_{68}N_4O_3$)

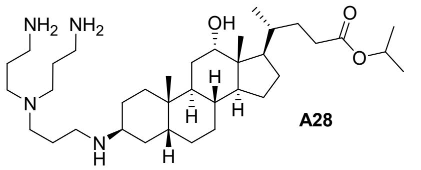

A28

**NMR $^1$H** (250 MHz, $CD_3OD$) : δ (ppm) = 4.96 (m, 1H), 3.95 (m, 1H), 2.90-2.50 (m, 13H), 2.37-2.18 (m, 3H), 2.04-0.96 (m, 47H), 0.71 (s, 3H). **NMR $^{13}$C** (63 MHz, $CD_3OD$) : δ (ppm) = 175.74, 74.08, 68.99, 59.14, 53.30, 52.86, 52.77, 49.38, 48.28, 47.75, 45.94, 43.90, 40.85,

37.53, 36.81, 35.80, 34.97, 32.73, 32.44, 29.98, 29.55, 29.46, 28.81, 28.48, 27.58, 26.89, 24.99, 24.48, 23.94, 22.24, 17.68, 13.33. **MS** (ESI+) : m/z 605.5 ([M+H]+). Yield : 26%

**3β-(Tris(2-aminoéthyl)amine)-isopropyl desoxycholate A29** ($C_{33}H_{62}N_4O_3$)

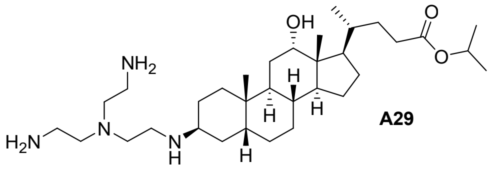


**NMR $^{1}$H** (250 MHz, $CD_3OD$) : δ (ppm) = 4.96 (m, 1H), 3.95 (m, 1H), 2.85-2.51 (m, 13H), 2.37-0.90 (m, 44H), 0.71 (s, 3H). **NMR $^{13}$C** (63 MHz, $CD_3OD$) : δ (ppm) = 175.77, 74.12, 69.00, 59.17, 57.74, 54.93, 48.28, 47.76, 45.19, 44.03, 40.22, 37.58, 37.51, 37.04, 36.82, 35.87, 34.99, 34.46, 32.73, 32.45, 29.98, 28.82, 27.64, 25.01, 24.01, 22.24, 17.68, 13.33. **MS** (ESI+) : m/z 563.4856 ([M+H]+). Yield : 24%

**3β-(Bis(3-aminopropyl)methylamine)-isopropyl desoxycholate A30** ($C_{34}H_{63}N_3O_3$)

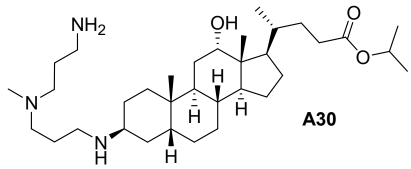


**NMR $^{1}$H** (250 MHz, $CD_3OD$) : δ (ppm) = 4.96 (m, 1H), 3.94 (m, 1H), 2.90-2.37 (m, 12H), 2.33-2.16 (m, 6H), 2.04-0.95 (m, 40H), 0.71 (s, 3H). **NMR $^{13}$C** (63 MHz, $CD_3OD$) : δ (ppm) = 175.74, 74.17, 68.98, 59.10, 57.12, 56.62, 49.34, 48.28, 47.77, 46.12, 44.06, 42.47, 42.42, 41.10, 37.58, 37.10, 36.81, 35.91, 34.96, 34.53, 32.72, 32.45, 30.84, 29.98, 28.82, 28.62, 28.24, 27.78, 27.63, 25.02, 24.26, 22.24, 17.68, 13.34. **MS** (ESI+) : m/z 562.5 ([M+H]+). Yield : 24%

- **Claramines B**

**3β-norspermidino-methyl cholate B2** ($C_{31}H_{57}N_3O_4$)

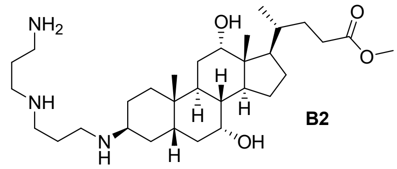


**NMR $^{1}$H** (250 MHz, $CD_3OD$) : δ (ppm) = 4.94 (m, 1H), 3.88 (m, 1H), 3.82 (m, 1H), 3.13-0.72 (m, 54H). **NMR $^{13}$C** (63 MHz, $CD_3OD$) : δ (ppm) = 173.10, 73.30, 68.41, 62.84, 51.90, 54.04, 47.42, 47.36, 43.37, 41.22, 41.12, 36.59, 36.31, 36.02, 35.80, 32.90, 32.50, 28.74, 28.23, 28.12, 26.01, 24.28, 19.10, 13.52, 12.64. **MS** (ESI+) : m/z 536.4381 ([M+H]+). Yield : 39%

**3β-(Bis(3-aminopropyl)methylamine)-methyl cholate B3**

($C_{32}H_{59}N_3O_4$)

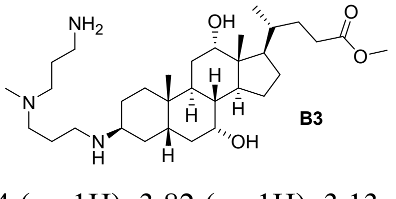
B3

**NMR $^1$H** (250 MHz, $CD_3OD$) : δ (ppm) = 4.92 (m, 1H), 3.84 (m, 1H), 3.82 (m, 1H), 3.13-0.78 (m, 56H). **NMR $^{13}$C** (63 MHz, $CD_3OD$) : δ (ppm) = 173.14, 73.28, 68.37, 62.91, 51.90, 54.04, 47.42, 47.36, 47.21, 43.37, 41.22, 41.12, 36.59, 36.29, 36.02, 35.48, 32.65, 31.50, 28.34, 28.13, 28.01, 26.11, 24.48, 19.0, 13.42, 12.64. **MS** (ESI$^+$) : m/z 550.4536 ($[M+H]^+$). Yield : 48%

**3β-spermino-ethyl cholateB11**

($C_{36}H_{68}N_4O_4$)

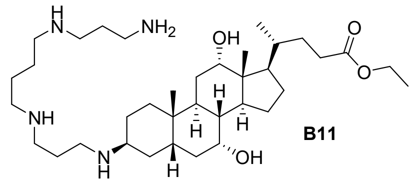
B11

**NMR $^1$H** (250 MHz, $CD_3OD$) : δ (ppm) = 4.11 (q, *J* = 7.1 Hz, 1H), 3.94 (m, 1H), 3.80 (m, 1H), 2.83-2.56 (m, 11H), 2.40-0.93 (m, 51H), 0.71 (s, 3H). **NMR $^{13}$C** (63 MHz, $CD_3OD$) : δ (ppm) = 176.22, 74.09, 69.14, 61.53, 59.43, 49.43, 48.39, 48.16, 47.66, 45.70, 43.75, 43.11, 41.20, 40.77, 37.72, 37.18, 36.89, 36.48, 36.03, 33.47, 32.42, 32.35, 32.02, 30.24, 29.69, 28.81, 28.36, 28.26, 28.03, 24.36, 23.50, 22.24, 18.53, 17.76, 14.73, 13.13. **MS** (ESI$^+$) : m/z 621.8 ($[M+H]^+$). Yield : 38%

**3β-spermino-isopropyl cholate B12**

($C_{37}H_{70}N_4O_4$)

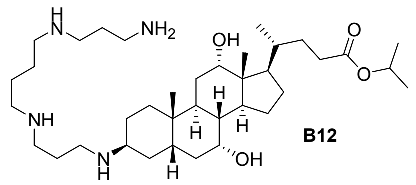
B12

**NMR $^1$H** (250 MHz, $CD_3OD$) : δ (ppm) = 4.96 (m, 1H), 3.96 (m, 1H), 3.80 (m, 1H), 2.95-2.69 (m, 15H), 2.34-0.95 (m, 49H), 0.71 (s, 3H). **NMR $^{13}$C** (63 MHz, $CD_3OD$) : δ (ppm) = 175.69, 73.89, 69.84, 68.96, 59.31, 56.08, 53.74, 49.91, 48.19, 47.64, 45.61, 45.43, 43.40, 43.17, 41.17, 40.37, 36.87, 36.63, 36.27, 35.96, 35.84, 32.74, 32.46, 30.78, 29.73, 28.87, 28.07, 27.80, 27.67, 26.91, 24.32, 23.32, 22.24, 17.73. 13.14. **MS** (ESI$^+$) : m/z 635.6 ($[M+H]^+$). Yield : 34%

**3β-norspermino-isopropyl cholate B13**

($C_{36}H_{68}N_4O_4$)

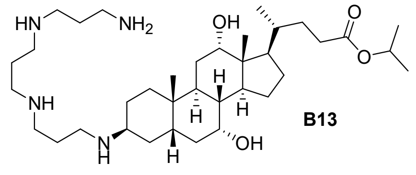
B13

**NMR $^1$H** (250 MHz, $CD_3OD$) : δ (ppm) = 4.96 (m, 1H), 3.98 (m, 1H), 3.82 (m, 1H), 3.13-2.81 (m, 13H), 2.59-0.92 (m, 49H), 0.72 (s, 3H). **NMR $^{13}$C** (63 MHz, $CD_3OD$) : δ (ppm) = 175.80, 73.96, 69.03, 68.96, 59.44, 54.04, 48.38, 47.75, 47.73, 43.37, 41.25, 41.22, 36.79, 36.21, 35.85, 35.80, 32.90, 32.51, 39.78, 28.75, 28.23, 28.19, 26.16, 24.28, 23.16, 22.20, 17.83, 13.13. **MS** ($ESI^+$) : m/z 621.6 ($[M+H]^+$). Yield : 30%

Mélange 50/50 de **3β-spermidino-isopropyl cholate B14a** et de **3β-*N*-[4'*N*-(3'aminopropyl)aminobutyl]amino-isopropyl cholate B14b** ($C_{34}H_{63}N_3O_4$)

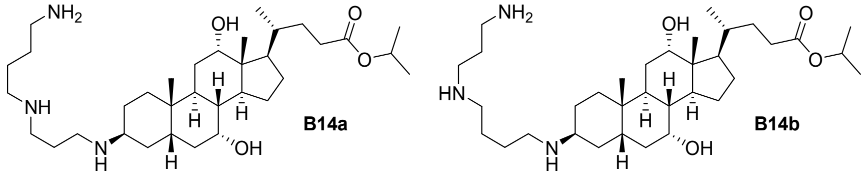


**NMR $^1$H** (250 MHz, $CD_3OD$) : δ (ppm) = 4.96 (m, 1H), 3.95 (m, 1H), 3.80 (m, 1H), 2.92-2.50 (m, 9H), 2.32-0.94 (m, 48H), 0.71 (s, 3H). **NMR $^{13}$C** (63 MHz, $CD_3OD$) : δ (ppm) = 175.74, 74.00, 69.03, 68.98, 59.37, 48.20, 47.67, 43.60, 43.14, 41.20, 36.92, 36.87, 36.38, 35.97, 35.95, 35.88, 32.73, 32.47, 29.72, 28.85, 28.06, 24.33, 23.44, 23.41, 22.24, 17.73, 13.14. Yield : 43%

**3β-norspermidino-isopropyl cholate B15**
($C_{33}H_{61}N_3O_4$)

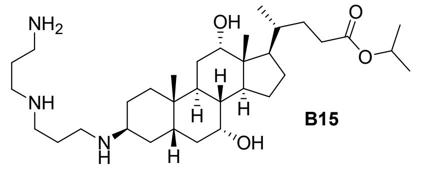


**NMR $^1$H** (250 MHz, $CD_3OD$) : δ (ppm) = 4.98 (m, 1H), 3.98 (m, 1H), 3.81 (m, 1H), 3.07-2.68 (m, 9H), 2.38-2.18 (m, 4H), 2.01-0.95 (m, 42H), 0.71 (s, 3H). **NMR $^{13}$C** (63 MHz, $CD_3OD$) : δ (ppm) = 175.82, 73.99, 69.05, 48.30, 48.24, 48.19, 47.70, 43.30, 43.25, 43.21, 43.16, 41.16, 36.71, 36.17, 32.85, 32.45, 32.14, 29.70, 28.68, 28.12, 24.25, 23.18, 22.20, 17.82, 13.13. **MS** ($ESI^+$) : m/z 564.6 ($[M+H]^+$). Yield : 45%

**3β-(1,4-Bis(3-aminopropyl)piperazine)-isopropyl cholate**
**B16** ($C_{37}H_{68}N_4O_4$)

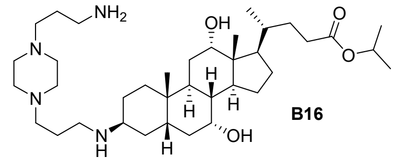


**NMR $^1$H** (250 MHz, $CD_3OD$) : δ (ppm) = 4.96 (m 1H), 3.94 (m, 1H), 3.79 (m, 1H), 2.84-2.40 (m, 19H), 2.34-0.94 (m, 43H), 0.71 (s, 3H). **NMR $^{13}$C** (63 MHz, $CD_3OD$) : δ (ppm) = 175.74, 74.06, 69.15, 68.98, 59.45, 58.06, 57.47, 54.21, 54.13, 48.33, 47.78, 46.23, 43.83, 43.19, 41.26, 37.23, 36.79, 36.48, 36.12, 32.87, 32.52, 30.57, 29.83, 28.75, 27.16, 24.34, 23.49, 22.23, 17.84, 13.18. **MS** ($ESI^+$) : m/z 633.5 ($[M+H]^+$). Yield : 28%

**3β-(pentaethylenehexamine)-isopropyl cholate B17** ($C_{37}H_{72}N_6O_4$)

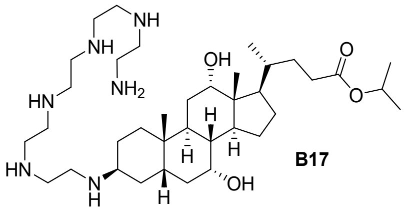


**NMR $^1$H** (250 MHz, $CD_3OD$) : δ (ppm) = 4.96 (m, 1H), 3.94 (m, 1H), 3.80 (m, 1H), 3.01-2.54 (m, 21H), 2.38-0.93 (m, 45H), 0.71 (s, 3H). **NMR $^{13}$C** (63 MHz, $CD_3OD$) : δ (ppm) = 175.69, 74.05, 69.10, 68.95, 59.50, 58.61, 55.36, 55.19, 54.26, 54.23, 48.16, 47.66, 46.21, 43.74, 43.09, 41.21, 39.09, 37.87, 37.84, 37.18, 36.84, 36.48, 36.06, 32.72, 32.46, 29.71, 28.84, 28.40, 28.36, 28.02, 24.37, 23.54, 22.27, 17.76, 13.18. **MS** ($ESI^+$) : m/z 665.5 ($[M+H]^+$). Yield : 45%

**3β-(Tris(3-aminopropyl)amine)-isopropyl cholate B18** ($C_{36}H_{68}N_4O_4$)

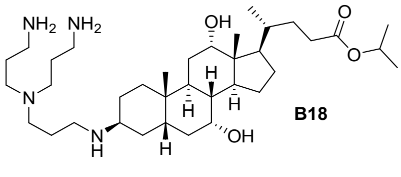


**NMR $^1$H** (250 MHz, $CD_3OD$) : δ (ppm) = 4.96 (m, 1H), 3.95 (m, 1H), 3.80 (m, 1H), 2.84-2.50 (m, 13H), 2.38-0.94 (m, 49H), 0.71 (s, 3H). **NMR $^{13}$C** (63 MHz, $CD_3OD$) : δ (ppm) = 175.69, 74.02, 69.08, 68.95, 59.42, 53.72, 52.86, 48.17, 47.65, 46.12, 43.71, 43.11, 41.20, 41.12, 37.54, 37.14, 36.84, 36.45, 36.05, 32.72, 32.46, 30.57, 29.71, 28.84, 28.17, 28.03, 27.30, 24.36, 23.52, 22.27, 17.76, 13.17. **MS** ($ESI^+$) : m/z 311.3 ($[M+2H]^{2+}$). Yield : 40%

**3β-(Tris(2-aminoéthyl)amine)-isopropyl cholate**
**B19** ($C_{33}H_{62}N_4O_4$)

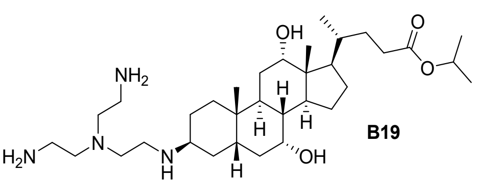

**NMR $^{1}$H** (250 MHz, $CD_3OD$) : δ (ppm) = 4.96 (m, 1H), 3.95 (m, 1H), 3.80 (m, 1H), 2.86-2.53 (m, 13H), 2.46-0.94 (m, 43H), 0.71 (s, 3H). **NMR $^{13}$C** (63 MHz, $CD_3OD$) : δ (ppm) = 175.71, 74.02, 69.11, 68.95, 59.45, 57.91, 55.17, 54.98, 51.72, 47.67, 46.35, 44.95, 43.72, 43.11, 41.24, 40.28, 37.75, 37.17, 36.68, 36.46, 36.00, 32.72, 32.46, 29.71, 28.85, 28.05, 24.36, 23.52, 22.25, 17.74, 13.16. **MS** ($ESI^+$) : m/z 579.5 ($[M+H]^+$). Yield : 35%

**3β-(Bis(3-aminopropyl)methylamine)-isopropyl cholate B20** ($C_{34}H_{63}N_3O_4$)

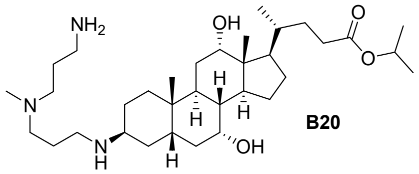


B20

**NMR $^{1}$H** (250 MHz, $CD_3OD$) : δ (ppm) = 4.96 (m, 1H), 3.96 (m, 1H), 3.80 (m, 1H), 2.81-2.41 (m, 12H), 2.34-0.94 (m, 45H), 0.71 (s, 3H). **NMR $^{13}$C** (63 MHz, $CD_3OD$) : δ (ppm) = 175.77, 74.00, 69.00, 59.37, 57.27, 56.59, 52.31, 47.65, 43.57, 43.22, 43.13, 42.36, 41.03, 40.97, 40.91, 36.86, 36.37, 35.92, 32.72, 32.46, 30.24, 28.86, 28.06, 24.32, 23.41, 22.24, 17.72, 13.14. **MS** ($ESI^+$) : m/z 578.4821 ($[M+H]^+$). Yield : 28%

- ## Claramines C

**3β-spermino-methyl chenodesoxycholate C1** ($C_{35}H_{66}N_4O_3$)

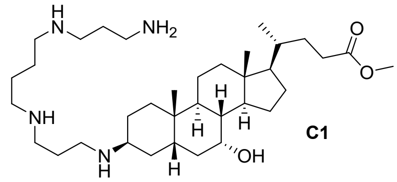


C1

**NMR $^{1}$H** (250 MHz, $CD_3OD$) : δ (ppm) = 3.84 (m, 1H), 3.66 (s, 3H), 2.89-2.59 (m, 13H), 2.38-2.22 (m, 3H), 2.04-1.09 (m, 37H), 0.97 (m, 6H), 0.71 (s, 3H). **NMR $^{13}$C** (63 MHz, $CD_3OD$) : δ (ppm) = 176.53, 69.01, 59.43, 57.66, 52.06, 51.80, 48.28, 45.83, 45.72, 43.90, 43.64, 41.28, 41.07, 40.70, 40.64, 36.82, 36.72, 36.02, 34.35, 32.46, 32.15, 31.70, 29.27, 27.92, 24.73, 23.56, 21.96, 18.98, 12.35. **MS** ($ESI^+$) : m/z 591.5 ($[M+H]^+$). Yield : 35%

**3β-norspermino-methyl chenodesoxycholate C2**

($C_{34}H_{64}N_4O_3$)

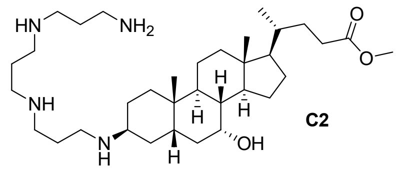

C2

**NMR $^{1}$H** (250 MHz, $CD_3OD$) : δ (ppm) = 3.79 (m, 1H), 3.65 (s, 3H), 2.78-0.95 (m, 57H), 0.70 (s, 3H). **NMR $^{13}$C** (63 MHz, $CD_3OD$) : δ (ppm) = 176.53, 69.12, 59.55, 57.58, 52.07, 51.74, 49.60, 48.95, 43.91, 43.81, 41.26, 41.09, 41.02, 40.85, 36.83, 34.34, 34.29, 33.31, 32.45, 32.14, 30.33, 29.34, 29.30, 24.75, 23.69, 22.22, 21.98, 18.99, 12.39. **MS** ($ESI^+$) : m/z 577.5 ($[M+H]^+$). Yield : 59%

Mélange 50/50 de **3β-spermidino-methyl chenodesoxycholate C3a** et de **3β-*N*-[4'*N*-(3'aminopropyl)aminobutyl]amino-methyl chenodesoxycholate C3b** ($C_{32}H_{59}N_3O_3$)

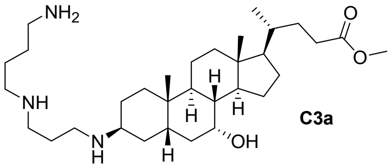

C3a

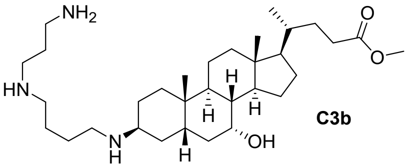

C3b

**NMR $^{1}$H** (250 MHz, $CD_3OD$) : δ (ppm) = 3.80 (m, 1H), 3.65 (m, 3H), 2.94-2.68 (m, 9H), 2.37-0.94 (m, 43H), 0.70 (s, 3H). **NMR $^{13}$C** (63 MHz, $CD_3OD$) : δ (ppm) = 176.56, 68.94, 59.45, 57.63, 53.69, 52.08, 51.82, 49.59, 45.61, 43.89, 41.25, 41.03, 36.81, 36.61, 35.89, 34.39, 32.44, 32.15, 29.40, 29.26, 24.74, 24.70, 23.42, 23.38, 22.17, 21.92, 19.27, 18.97, 12.36. Yield : 27%

**3β-norspermidino-methyl chenodesoxycholate C4**

($C_{31}H_{57}N_3O_3$)

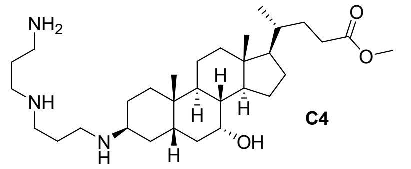

C4

**NMR $^{1}$H** (250 MHz, $CD_3OD$) : δ (ppm) = 3.81 (m, 1H), 3.65 (s, 3H), 3.03-2.66 (m, 9H), 2.49-0.94 (m, 41H), 0.70 (s, 3H). **NMR $^{13}$C** (63 MHz, $CD_3OD$) : δ (ppm) = 176.58, 69.02, 59.42, 57.65, 52.07, 51.82, 45.70, 45.69, 43.44, 43.90, 43.41, 41.26, 41.24, 41.04, 36.82, 36.60, 35.89, 35.84, 34.43, 34.37, 32.44, 32.14, 29.26, 24.70, 21.93, 18.96, 12.34. **MS** ($ESI^+$) : m/z 520.5 ($[M+H]^+$). Yield : 27%

**3β-(1,4-Bis(3-aminopropyl)piperazine)-methyl chenodesoxycholate C5** ($C_{35}H_{64}N_4O_3$)

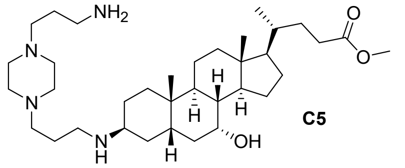

C5

**NMR $^1$H** (250 MHz, $CD_3OD$) : δ (ppm) = 3.82 (m 1H), 3.68 (s, 3H), 2.87-2.19 (m, 23H), 2.06-0.98 (m, 34H), 0.73 (s, 3H). **NMR $^{13}$C** (63 MHz, $CD_3OD$) : δ (ppm) = 176.47, 69.08, 59.41, 58.12, 57.51, 54.25, 54.12, 52.07, 51.77, 46.30, 43.91, 43.80, 41.26, 41.11, 37.81, 37.25, 36.82, 36.37, 34.37, 32.46, 32.15, 30.54, 29.30, 28.50, 27.04, 24.77, 23.69, 21.97, 19.02, 12.42. **MS** ($ESI^+$) : m/z 589.5 ($[M+H]^+$). Yield : 35%

**3β-(éthylènediamine)-methyl chenodesoxycholate C6** ($C_{27}H_{48}N_2O_3$)

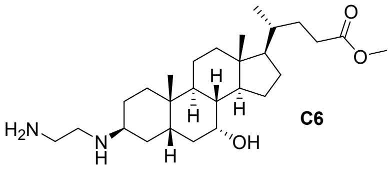

C6

**NMR $^1$H** (250 MHz, $CD_3OD$) : δ (ppm) = 3.80 (m, 1H), 3.65 (s, 3H), 2.97-2.68 (m, 5H), 2.44-2.18 (m, 4H), 2.03, 0.96 (m, 32H), 0.71 (s, 3H). **NMR $^{13}$C** (63 MHz, $CD_3OD$) : δ (ppm) = 176.53, 69.14, 59.56, 57.58, 52.07, 51.74, 43.90, 42.03, 41.97, 41.24, 41.08, 37.85, 37.28, 36.82, 36.14, 34.32, 32.45, 32.12, 29.28, 28.58, 24.75, 23.69, 22.21, 21.97, 18.99, 12.39. **MS** ($ESI^+$) : m/z 449.3695 ($[M+H]^+$). Yield : 42%

**3β-(1,3-diaminopropane)-methyl chenodesoxycholate C7** ($C_{28}H_{50}N_2O_3$)

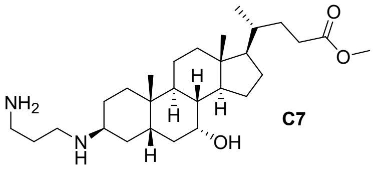

C7

**NMR $^1$H** (250 MHz, $CD_3OD$) : δ (ppm) = 3.80 (m, 1H), 3.65 (s, 3H), 2.86-2.62 (m, 4H), 2.44-0.94 (m, 39H), 0.69 (s, 3H). **NMR $^{13}$C** (63 MHz, $CD_3OD$) : δ (ppm) = 176.98, 69.06, 59.46, 57.48, 51.67, 45.09, 43.82, 43.62, 41.18, 40.94, 40.65, 39.75, 37.66, 37.09, 36.76, 36.05, 34.18, 33.54, 33.35, 29.46, 28.02, 24.79, 23.73, 21.94, 19.08, 12.39. **MS** ($ESI^+$) : m/z 463.3 ($[M+H]^+$). Yield : 57%

**3β-(1,4-diaminobutane)-methyl chenodesoxycholate C8** ($C_{29}H_{52}N_2O_3$)

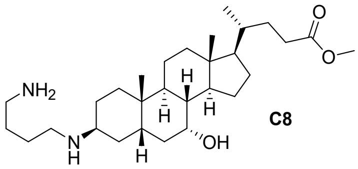

C8

**NMR $^1$H** (250 MHz, $CD_3OD$) : δ (ppm) = 3.80 (m, 1H), 3.65 (m, 3H), 2.90-2.44 (m, 5H), 2.44-2.18 (m, 3H), 2.03-0.94 (m, 37H), 0.69 (s, 3H). **NMR $^{13}$C** (63 MHz, $CD_3OD$) : δ (ppm)

= 176.56, 69.12, 59.48, 57.62, 52.07, 51.75, 46.97, 43.90, 43.73, 42.30, 42.18, 41.24, 41.08, 37.14, 36.82, 36.08, 34.35, 32.45, 32.16, 31.18, 29.27, 27.82, 24.74, 23.64, 21.96, 18.99, 12.38. **MS** ($ESI^+$) : m/z 477.4 ($[M+H]^+$). Yield : 31%

**3β-(1,5-diaminopentane)-methyl chenodesoxycholate C9**
($C_{30}H_{54}N_2O_3$)

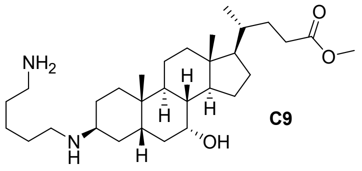
C9

**NMR $^1$H** (250 MHz, $CD_3OD$) : δ (ppm) = 3.80 (m, 1H), 3.65 (s, 3H), 2.86-2.62 (m, 5H), 2.44-2.20 (m, 3H), 2.03-0.94 (m, 39H), 0.71 (s, 3H). **NMR $^{13}$C** (63 MHz, $CD_3OD$) : δ (ppm) = 176.58, 69.17, 59.58, 57.68, 52.06, 51.73, 49.60, 43.91, 42.43, 41.26, 41.08, 36.83, 34.34, 3336, 32.45, 32.15, 29.27, 25.82, 25.72, 24.74, 23.63, 22.59, 22.19, 21.95, 18.96, 12.35. **MS** ($ESI^+$) : m/z 491.4 ($[M+H]^+$). Yield : 31%

**3β-(1,6-diaminohexane)-methyl chenodesoxycholate C10**
($C_{31}H_{56}N_2O_3$)

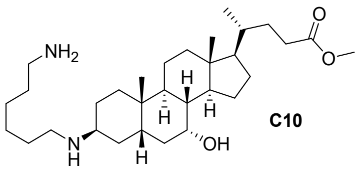
C10

**NMR $^1$H** (250 MHz, $CD_3OD$) : δ (ppm) = 3.80 (m, 1H), 3.65 (s, 3H), 2.88-0.95 (m, 49H), 0.71 (s, 3H). **NMR $^{13}$C** (63 MHz, $CD_3OD$) : δ (ppm) = 176.53, 69.11, 59.60, 57.62, 52.05, 51.75, 47.15, 43.91, 43.73, 42.42, 41.26, 41.09, 37.12, 36.82, 36.08, 34.35, 33.26, 32.46, 32.14, 30.01, 29.98, 29.27, 28.18, 27.94, 27.81, 24.74, 23.62, 21.97, 18.99, 12.37. **MS** ($ESI^+$) : m/z 505.4 ($[M+H]^+$). Yield : 25%

**3β-(pentaethylenehexamine)-methyl chenodesoxycholate C11** ($C_{35}H_{68}N_6O_3$)

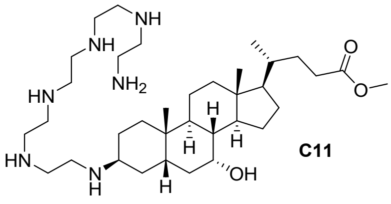
C11

**NMR $^1$H** (250 MHz, $CD_3OD$) : δ (ppm) = 3.79 (m, 1H), 3.65 (s, 3H), 2.98-2.44 (m, 21H), 2.38-0.95 (m, 40H), 0.70 (s, 3H). **NMR $^{13}$C** (63 MHz, $CD_3OD$) : δ (ppm) = 176.54, 69.14, 59.66, 57.60, 57.53, 54.56, 54.34, 54.18, 52.07, 51.74, 50.02, 49.91, 46.40, 43.90, 41.27, 41.10, 37.33, 36.84, 36.21, 34.34, 32.45, 32.14, 29.29, 24.76, 23.72, 21.97, 18.99, 12.38. **MS** ($ESI^+$) : m/z 621.5386 ($[M+H]^+$). Yield : 46%

**3β-(Bis(3-aminopropyl)methylamine)- methyl chenodesoxycholate C12** ($C_{32}H_{59}N_3O_3$)

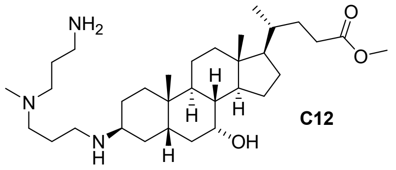
C12

**NMR $^1$H** (250 MHz, $CD_3OD$) : δ (ppm) = 3.79 (m, 1H), 3.64 (s, 3H), 2.89-2.39 (m, 12H), 2.29-0.93 (m, 40H), 0.68 (s, 3H). **NMR $^{13}$C** (63 MHz, $CD_3OD$) : δ (ppm) = 176.53, 89.36, 69.08, 57.59, 57.33, 56.99, 56.74, 52.06, 51.75, 51.71, 43.90, 43.66, 42.38, 41.22, 41.10, 37.03, 36.82, 36.06, 34.33, 34.28, 32.43, 32.14, 30.39, 29.23, 24.72, 23.60, 22.00, 21.95, 18.99, 12.39. **MS** ($ESI^+$) : m/z 534.8588 ($[M+H]^+$). Yield : 40%

**3β-(Tris(3-aminopropyl)amine)- methyl chenodesoxycholate C13** ($C_{34}H_{64}N_4O_3$)

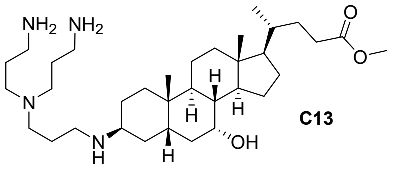
C13

**NMR $^1$H** (250 MHz, $CD_3OD$) : δ (ppm) = 3.80 (m, 1H), 3.65 (s, 3H), 2.80-0.94 (m, 57H), 0.70 (s, 3H). **NMR $^{13}$C** (63 MHz, $CD_3OD$) : δ (ppm) = 176.82, 69.08, 59.57, 57.48, 54.24, 53.87, 53.56, 52.91, 52.85, 51.71, 45.69, 43.83, 41.15, 38.93, 37.61, 37.10, 36.82, 34.22, 33.62, 32.39, 31.97, 30.58, 29.52, 27.81, 27.10, 24.82, 23.73, 21.94, 19.20, 18.94, 12.35. **MS** ($ESI^+$) : m/z 577.5011 ($[M+H]^+$). Yield : 25%

**3β-(cyclohexylamine)-methyl chenodesoxycholate C14** ($C_{31}H_{53}NO_3$)

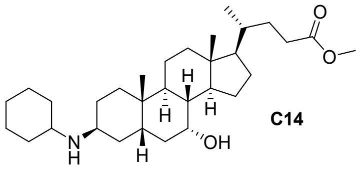
C14

**NMR $^{13}$C** (63 MHz, $CD_3OD$) : δ (ppm) = 176.47, 69.15, 57.55, 52.05, 51.70, 47.86, 43.89, 41.22, 41.03, 36.79, 36.52, 35.74, 34.28, 32.99, 32.43, 32.13, 30.76, 29.26, 26.24, 25.84, 25.70, 24.75, 23.55, 23.44, 23.32, 22.22, 21.96, 19.01, 12.39. **MS** ($ESI^+$) : m/z 488.4 ($[M+H]^+$). Yield : 45%

**3β-(1-(3-aminopropyl)imidazole)-methyl chenodesoxycholate C15** ($C_{31}H_{51}NO_3$)

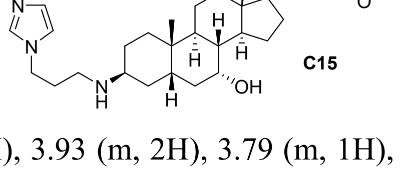
C15

**NMR $^1$H** (250 MHz, $CD_3OD$) : δ (ppm) = 7.62-6.89 (m, 3H), 3.93 (m, 2H), 3.79 (m, 1H), 2.86-2.67 (m, 2H), 2.34-2.11 (m, 6H), 1.63-0.87 (m, 33H), 0.68 (s, 3H). **NMR $^{13}$C** (63 MHz, $CD_3OD$) : δ (ppm) = 176.77, 138.91, 129.66, 121.25, 69.02, 59.67, 57.65, 51.81, 47.59,

46.21, 46.12, 44.50, 43.89, 41.32, 41.13, 37.71, 37.39, 37.02, 36.87, 36.33, 34.37, 33.44, 32.45, 32.20, 29.41, 28.92, 24.82, 23.83, 22.02, 19.26, 12.55. **MS** ($ESI^+$) : m/z 514.4 ($[M+H]^+$). Yield : 46%

**3β-spermino-isopropyl chenodesoxycholate C16**
($C_{37}H_{70}N_4O_3$)

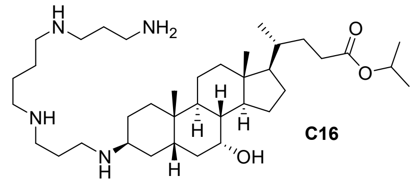


**NMR $^1$H** (250 MHz, $CD_3OD$) : δ (ppm) = 4.96 (m, 1H), 3.80 (m, 1H), 3.03-2.68 (m, 13H), 2.32-2.20 (m, 4H), 2.03-0.94 (m, 48H), 0.70 (s, 3H). **NMR $^{13}$C** (63 MHz, $CD_3OD$) : δ (ppm) = 175.63, 69.00, 59.38, 57.69, 51.78, 51.75, 49.90, 45.74, 43.90, 43.51, 41.24, 41.21, 41.05, 41.01, 36.73, 36.66, 35.98, 35.92, 34.35, 34.30, 32.83, 32.50, 29.27, 24.75, 24.71, 23.52, 22.21, 21.94, 18.99, 12.39. **MS** ($ESI^+$) : m/z 619.6 ($[M+H]^+$). Yield : 38%

**3β-norspermino-isopropyl chenodesoxycholate C17**
($C_{36}H_{68}N_4O_3$)

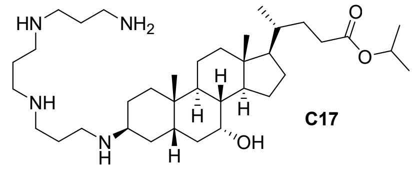


**NMR $^1$H** (250 MHz, $CD_3OD$) : δ (ppm) = 4.96 (m, 1H), 3.79 (m, 1H), 2.80-2.59 (m, 13H), 2.42-2.17 (m, 5H), 2.02-0.92 (m, 45H), 0.69 (s, 3H). **NMR $^{13}$C** (63 MHz, $CD_3OD$) : δ (ppm) = 175.62, 69.16, 68.98, 59.58, 57.61, 51.74, 48.54, 45.93, 43.92, 41.27, 41.13, 40.94, 38.24, 37.43, 36.88, 36.77, 36.24, 34.32, 33.91, 32.85, 32.53, 30.82, 30.56, 29.32, 28.91, 24.77, 23.78, 22.25, 21.98, 19.03, 12.43. **MS** ($ESI^+$) : m/z 605.5364 ($[M+H]^+$). Yield : 40%

Mélange 50/50 de **3β-spermidino-isopropyl chenodesoxycholate C18a** et de **3β-*N*-[4'*N*-(3'aminopropyl)aminobutyl]amino-isopropyl chenodesoxycholate C18b** ($C_{34}H_{63}N_3O_3$)

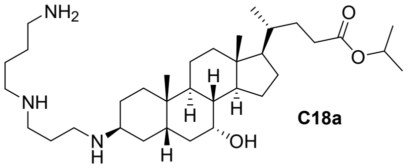
C18a

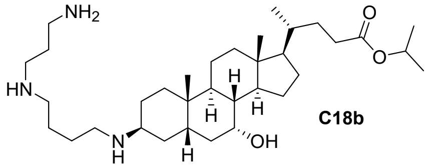
C18b

**NMR $^{1}$H** (250 MHz, $CD_3OD$) : δ (ppm) = 4.98 (m, 1H), 3.80 (m, 1H), 2.86-2.48 (m, 9H), 2.42-2.14 (m, 4H), 2.02-0.95 (m, 45H), 0.69 (s, 3H). **NMR $^{13}$C** (63 MHz, $CD_3OD$) : δ (ppm) = 175.59, 69.05, 68.97, 59.44, 59.39, 57.50, 51.67, 50.48, 48.90, 47.15, 45.71, 43.82, 43.66, 43.62, 42.38, 41.20, 40.94, 40.72, 37.17, 37.10, 36.83, 36.79, 36.04, 34.18, 32.67, 32.45, 31.36, 29.42, 28.35, 28.27, 27.89, 24.76, 23.76, 23.73, 22.27, 21.92, 18.97, 12.39. Yield : 49%

**3β-norspermidino-isopropyl chenodesoxycholate C19** ($C_{33}H_{61}N_3O_3$)

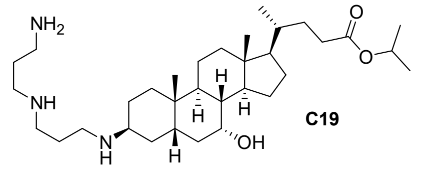
C19

**NMR $^{1}$H** (250 MHz, $CD_3OD$) : δ (ppm) = 4.96 (m, 1H), 3.79 (m, 1H), 2.82-2.60 (m, 9H), 2.42-2.13 (m, 4H), 2.02-0.95 (m, 43H), 0.69 (s, 3H). **NMR $^{13}$C** (63 MHz, $CD_3OD$) : δ (ppm) = 175.63, 69.10, 68.99, 59.47, 57.50, 51.67, 49.05, 48.39, 45.76, 43.83, 43.72, 41.19, 40.96, 40.76, 37.74, 37.25, 36.82, 36.08, 34.19, 33.45, 32.69, 32.46, 30.24, 29.41, 28.43, 24.77, 23.78, 22.27, 21.94, 18.97, 12.39. **MS** ($ESI^+$) : m/z 548.4786 ($[M+H]^+$). Yield : 45%

**3β-(1,4-Bis(3-aminopropyl)piperazine)-isopropyl chenodesoxycholate C20** ($C_{37}H_{68}N_4O_3$)

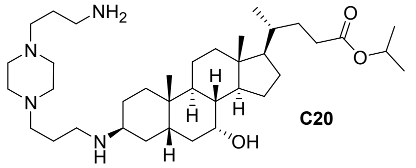
C20

**NMR $^{1}$H** (250 MHz, $CD_3OD$) : δ (ppm) = 4.96 (m, 1H), 3.79 (m, 1H), 2.84-2.40 (m, 17H), 2.33-0.95 (m, 46H), 0.71 (s, 3H). **NMR $^{13}$C** (63 MHz, $CD_3OD$) : δ (ppm) = 175.53, 69.04, 68.95, 59.42, 58.10, 57.65, 57.50, 54.24, 54.11, 51.75, 46.30, 43.90, 43.78, 41.26, 41.08, 37.27, 36.81, 36.76, 36.19, 34.34, 32.83, 32.50, 30.48, 29.32, 28.52, 27.10, 24.75, 23.74, 22.27, 21.98, 19.06, 12.47. **MS** ($ESI^+$) : m/z 617.5 ($[M+H]^+$). Yield : 44%

**3β-spermino-ethyl chenodesoxycholate C21** ($C_{36}H_{68}N_4O_3$)

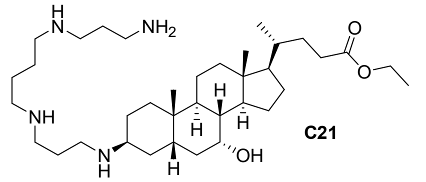
C21

**NMR $^{1}$H** (250 MHz, $CD_3OD$) : δ (ppm) = 4.09 (q, *J* = 7.1 Hz, 2H), 3.79 (m, 1H), 2.90-2.63 (m, 12H), 2.39-2.18 (m, 4H), 2.02-0.93 (m, 46H), 0.68 (s, 3H). **NMR $^{13}$C** (63 MHz, $CD_3OD$)

: δ (ppm) = 176.14, 69.11, 61.49, 59.48, 57.66, 53.38, 51.77, 50.41, 45.83, 43.91, 43.73, 41.27, 41.07, 40.79, 36.80, 36.06, 34.55, 32.77, 29.28, 28.21, 24.74, 23.60, 21.96, 18.98, 14.67, 12.36. **MS** ($ESI^+$) : m/z 605.5 ($[M+H]^+$). Yield : 26%

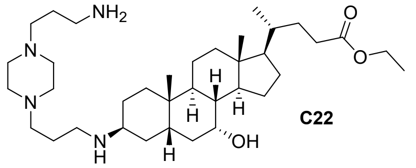

C22

**3β-(1,4-Bis(3-aminopropyl)piperazine)-ethyl chenodesoxycholate C22** ($C_{36}H_{66}N_4O_3$)

**NMR $^1$H** (250 MHz, $CD_3OD$) : δ (ppm) = 4.11 (q, $J$ = 7.1 Hz, 2H), 3.80 (m, 1H), 2.84-2.30 (m, 21H), 2.03-1.66 (m, 15H), 1.57-1.08 (m, 18H), 0.96 (m, 6H), 0.7 (s, 3H). **NMR $^{13}$C** (63 MHz, $CD_3OD$) : δ (ppm) = 176.02, 69.03, 61.46, 59.38, 57.66, 57.48, 54.28, 54.15, 51.79, 46.29, 43.92, 43.72, 41.30, 41.22, 41.10, 37.41, 37.14, 36.79, 36.14, 34.37, 32.47, 30.24, 29.30, 28.26, 26.62, 24.75, 23.65, 22.49, 21.97, 19.04, 14.71, 12.43. **MS** ($ESI^+$) : m/z 603.5 ($[M+H]^+$). Yield : 66%

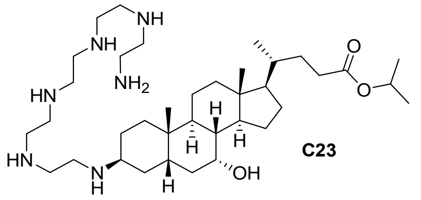

C23

**3β-(pentaethylenehexamine)-isopropyl chenodesoxycholate C23** ($C_{37}H_{72}N_6O_3$)

**NMR $^1$H** (250 MHz, $CD_3OD$) : δ (ppm) = 4.96 (m, 1H), 3.79 (m, 1H), 2.97-2.45 (m, 20H), 2.39-2.17 (m, 4H), 2.02-0.88 (m, 43H), 0.71 (s, 3H). **NMR $^{13}$C** (63 MHz, $CD_3OD$) : δ (ppm) = 175.60, 69.06, 68.96, 59.59, 58.62, 57.50, 55.39, 55.20, 54.29, 51.68, 43.84, 43.76, 41.21, 40.98, 38.02, 37.28, 36.84, 36.12, 34.21, 32.69, 32.47, 29.42, 28.70, 24.77, 23.80, 22.27, 21.94, 18.98, 12.40. **MS** ($ESI^+$) : m/z 649.5698 ($[M+H]^+$). Yield : 30%

**3β-(Tris(3-aminopropyl)amine)-isopropyl chenodesoxycholate C24** ($C_{36}H_{68}N_4O_3$)

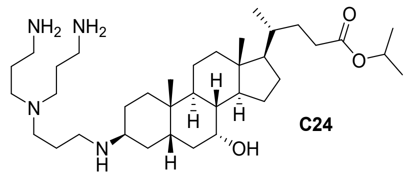

**NMR $^1$H** (250 MHz, $CD_3OD$) : δ (ppm) = 4.96 (m, 1H), 3.80 (m, 1H), 2.70-2.47 (m, 13H), 2.39-2.15 (m, 4H), 2.03-1.10 (m, 40H), 0.97-0.95 (m, 6H), 0.69 (s, 3H). **NMR $^{13}$C** (63 MHz, $CD_3OD$) : δ (ppm) = 175.63, 69.08, 59.57, 57.51, 53.57, 52.86, 51.70, 46.23, 43.84, 43.71, 41.22, 41.14, 40.97, 37.68, 37.21, 36.82, 36.08, 34.22, 32.69, 32.47, 30.64, 29.41, 28.41, 27.40, 24.77, 23.76, 22.27, 21.94, 18.96, 12.39. **MS** ($ESI^+$) : m/z 605.5364 ($[M+H]^+$). Yield : 37%

**3β-(Tris(2-aminoéthyl)amine)-isopropyl chenodesoxycholateC25** ($C_{33}H_{62}N_4O_3$)

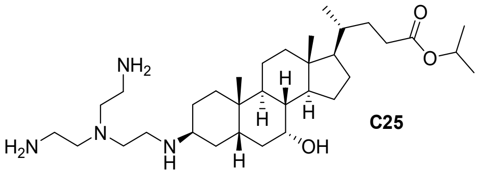

**NMR $^1$H** (250 MHz, $CD_3OD$) : δ (ppm) = 4.96 (m, 1H), 3.80 (m, 1H), 2.84-2.54 (m, 13H), 2.46-0.95 (m, 44H), 0.69 (s, 3H). **NMR $^{13}$C** (63 MHz, $CD_3OD$) : δ (ppm) = 175.61, 69.08, 68.98, 59.48, 57.76, 57.50, 54.86, 51.68, 44.98, 43.82, 43.66, 41.19, 40.97, 40.21, 37.66, 37.20, 36.82, 36.79, 36.04, 34.21, 32.69, 32.45, 29.41, 24.76, 23.76, 22.27, 21.92, 18.97, 12.41. **MS** ($ESI^+$) : m/z 563.5 ($[M+H]^+$). Yield : 40%

**3β-(Bis(3-aminopropyl)methylamine)-isopropyl chenodesoxycholate C26** ($C_{34}H_{63}N_3O_3$)

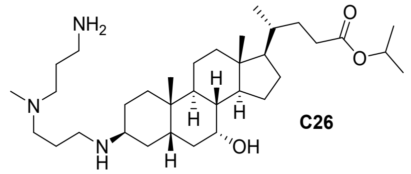

**NMR $^1$H** (250 MHz, $CD_3OD$) : δ (ppm) = 4.96 (m, 1H), 3.79 (m, 1H), 2.74-2.29 (m, 12H), 2.24-0.95 (m, 46H), 0.69 (s, 3H). **NMR $^{13}$C** (63 MHz, $CD_3OD$) : δ (ppm) = 175.56, 69.08, 68.95, 59.53, 57.48, 57.17, 56.58, 51.66, 46.11, 43.63, 42.46, 41.21, 41.12, 40.97, 37.87, 37.30, 36.83, 36.11, 34.18, 32.68, 32.46, 30.99, 29.42, 28.56, 27.78, 24.78, 23.81, 22.27, 21.94, 18.98, 12.41. **MS** ($ESI^+$) : m/z 562.5 ($[M+H]^+$). Yield : 47%

**Synthesis of Claramine C27**

In a three-necked round-bottom flask equipped with magnetic stirring, 3-oxo-isopropyl chenodeoxycholate **186** (85 mg, 0.20 mmol) was dissolved in methanol (5 mL). Titanium tetra(isopropoxide) (180 µL, 0.60 mmol, 3 equiv) was added under stirring, followed by $N^1$,$N^5$-bis-Boc-spermidine 188 (140 mg, 0.60 mmol, 2 equiv), and the reaction mixture was stirred for 24 h. The flask was cooled in an ice bath, and $NaBH_4$ (30 mg, 0.80 mmol, 4 equiv)

was added at 0 °C. After stirring for 2 h, water (300 µL) was added at room temperature, and the mixture was stirred for an additional 1 h. The reaction mixture was filtered through Celite, washed with aqueous ammonia and methanol, and concentrated under reduced pressure. The residue was dissolved in $CH_2Cl_2$ (5 mL), cooled in an ice bath, and treated with trifluoroacetic acid (750 µL). Stirring was continued for 36 h. The solvent was evaporated under reduced pressure, and the residue was co-evaporated three times with $CH_2Cl_2$ (5 mL) to remove excess trifluoroacetic acid. The crude material was dissolved in MeOH (3 mL), followed by addition of isopropylamine (1 mL) under stirring. After concentration and purification by silica gel chromatography (eluent: $CH_2Cl_2$/MeOH/32% aqueous $NH_3$, 7:3:1), claramine C27 was obtained as a yellow oil in 24% yield.

**3β-spermidino-isopropyl chenodesoxycholate C27** ($C_{34}H_{63}N_3O_3$).

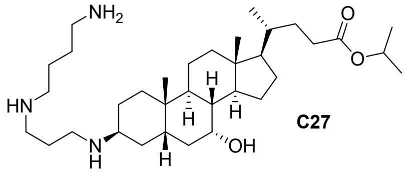

C27

**NMR ¹H** (250 MHz, $CD_3OD$) : δ (ppm) = 4.98 (m, 1H), 3.80 (m, 1H), 2.87-2.58 (m, 11H), 2.42-0.95 (m, 47H), 0.69 (s, 3H). **NMR ¹³C** (63 MHz, $CD_3OD$) : δ (ppm) = 175.74, 69.12, 69.04, 59.45, 57.51, 51.69, 50.46, 47.86, 45.69, 43.83, 43.65, 42.36, 41.18, 40.94, 40.69, 37.46, 37.12, 36.84, 36.01, 34.20, 32.69, 32.46, 31.31, 29.79, 29.68, 29.41, 28.17, 27.89, 24.75, 23.71, 22.24, 21.92, 18.93, 12.34. **MS** ($ESI^+$) : m/z 562.5 ($[M+H]^+$)

- ## Claramines D

**3β-spermino-methyl ursodesoxycholate D1** ($C_{35}H_{66}N_4O_3$)

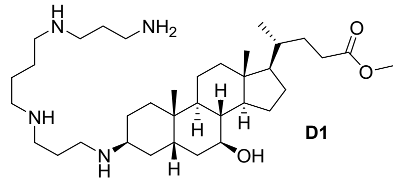

D1

**NMR ¹H** (250 MHz, $CD_3OD$) : δ (ppm) = 3.65 (s, 3H), 3.47 (m, 1H), 2.87-2.52 (m, 13H), 2.41-2.20 (m, 3H), 2.06-0.94 (m, 43H), 0.71 (s, 3H). **NMR ¹³C** (63 MHz, $CD_3OD$) : δ (ppm) = 176.49, 72.01, 58.87, 57.64, 57.60, 56.68, 52.18, 50.46, 48.27, 46.73, 45.78, 44.91, 44.88, 44.65, 41.68, 40.78, 40.62, 38.84, 38.82, 36.82, 36.73, 35.75, 35.13, 32.56, 32.42, 32.01, 29.92, 29.79, 28.15, 28.02, 24.28, 22.54, 19.09, 12.81. **MS** ($ESI^+$) : m/z 591.52 ($[M+H]^+$). Yield : 48%

**3β-norspermino-methyl ursodesoxycholate D2** ($C_{34}H_{64}N_4O_3$)

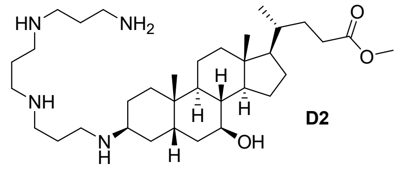

D2

**NMR $^{1}$H** (250 MHz, $CD_3OD$) : δ (ppm) = 3.64 (s, 3H), 3.46 (m, 1H), 2.84-2.48 (m, 13H), 2.36-2.00 (m, 3H), 1.88-0.93 (m, 41H), 0.70 (s, 3H). **NMR $^{13}$C** (63 MHz, $CD_3OD$) : δ (ppm) = 176.53, 72.06, 58.91, 57.62, 56.69, 52.16, 48.95, 45.90, 44.93, 44.68, 44.51, 41.70, 40.80, 40.72, 38.87, 36.82, 35.78, 35.35, 35.30, 33.18, 32.43, 33.65, 33.63, 32.02, 30.36, 30.19, 29.79, 28.18, 28.08, 24.28, 22.54, 19.07, 12.80. **MS** ($ESI^+$) : m/z 577.5 ($[M+H]^+$). Yield : 31%

Mélange 50/50 de **3β-spermidino-methyl ursodesoxycholate D3a** et de **3β-*N*-[4'*N*-(3'aminopropyl)aminobutyl]amino-methyl ursodesoxycholate D3b** ($C_{32}H_{59}N_3O_3$)

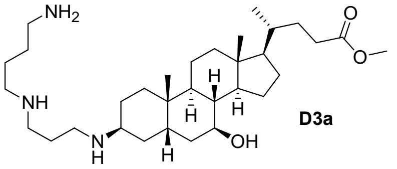

D3a

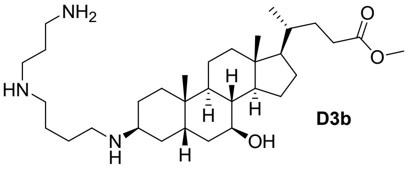

D3b

**NMR $^{1}$H** (250 MHz, $CD_3OD$) : δ (ppm) = 3.66 (m, 3H), 3.48 (m, 1H), 2.92-2.64 (m, 9H), 2.41-2.21 (m, 2H), 2.05-0.93 (m, 41H), 0.71 (s, 3H). **NMR $^{13}$C** (63 MHz, $CD_3OD$) : δ (ppm) = 176.60, 72.09, 58.91, 58.86, 57.64, 56.74, 52.15, 50.45, 49.28, 48.98, 44.95, 44.91, 44.72, 44.54, 44.40, 42.33, 41.73, 40.85, 40.74, 38.86, 38.84, 36.80, 35.78, 32.45, 32.07, 30.33, 30.17, 29.87, 29.74, 28.40, 28.19, 28.07, 27.88, 24.25, 22.54, 19.06, 12.79. Yield : 50%

**3β-norspermidino-methyl ursodesoxycholate D4**
($C_{31}H_{57}N_3O_3$)

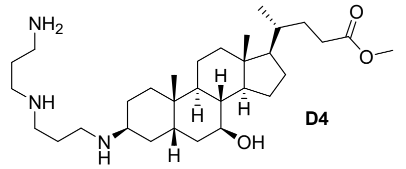

D4

**NMR $^{1}$H** (250 MHz, $CD_3OD$) : δ (ppm) = 3.64 (s, 3H), 3.47 (m, 1H), 2.79-2.63 (m, 7H), 2.40-2.20 (m, 3H), 2.06-0.94 (m, 40H), 0.71 (s, 3H). **NMR $^{13}$C** (63 MHz, $CD_3OD$) : δ (ppm) = 176.51, 72.05, 58.94, 57.68, 56.82, 52.07, 48.89, 48.30, 45.79, 45.00, 44.85, 44.51, 41.75, 41.69, 40.87, 40.64, 38.74, 36.73, 35.75, 32.47, 32.17, 30.24, 29.79, 29.65, 28.03, 24.16, 22.57, 19.12, 12.82. **MS** ($ESI^+$) : m/z 520.4 ($[M+H]^+$). Yield : 41%

**3β-(1,4-Bis(3-aminopropyl)piperazine)-methyl ursodesoxycholate D5** ($C_{35}H_{64}N_4O_3$)

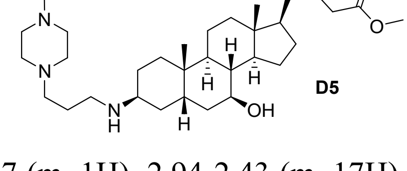

D5

**NMR $^{1}$H** (250 MHz, $CD_3OD$) : δ (ppm) = 3.65 (s, 3H), 3.47 (m, 1H), 2.94-2.43 (m, 17H), 2.37-0.94 (m, 40H), 0.72 (s, 3H). **NMR $^{13}$C** (63 MHz, $CD_3OD$) : δ (ppm) = 176.51, 71.93,

58.72, 57.80, 57.64, 57.34, 56.71, 54.13, 53.97, 52.20, 46.08, 44.92, 44.88, 44.62, 44.29, 41.69, 40.80, 40.71, 38.69, 36.82, 36.45, 25.66, 34.40, 32.42, 32.01, 29.79, 28.24, 28.07, 27.40, 26.22, 24.17, 22.52, 19.08, 12.80. **MS** ($ESI^+$) : m/z 589.5 ($[M+H]^+$). Yield : 41%

**3β-(1,5-diaminopentane)-methyl ursodesoxycholate D6** ($C_{30}H_{54}N_2O_3$)

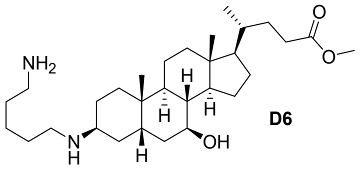

D6

**NMR $^1$H** (250 MHz, $CD_3OD$) : δ (ppm) = 3.65 (m, 3H), 3.47 (m, 1H), 2.84-2.57 (m, 4H), 2.52-2.16 (m, 4H), 2.05-1.09 (m, 33H), 1.01-0.94 (m, 6H), 0.71 (s, 3H). **NMR $^{13}$C** (63 MHz, $CD_3OD$) : δ (ppm) = 176.53, 72.05, 59.80, 58.95, 57.66, 57.60, 56.68, 56.65, 52.17, 47.59, 44.92, 44.89, 44.66, 44.47, 42.32, 41.68, 40.79, 36.84, 35.76, 33.12, 32.42, 32.01, 30.37, 29.79, 28.08, 25.82, 24.26, 22.54, 19.06, 12.79. **MS** ($ESI^+$) : m/z 491.4165 ($[M+H]^+$). Yield : 21%

**3β-norspermidino-isopropyl ursodesoxycholate D7** ($C_{33}H_{61}N_3O_3$)

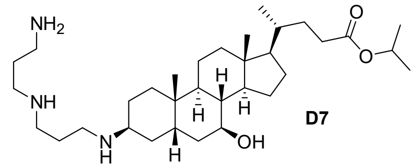

D7

**NMR $^1$H** (250 MHz, $CD_3OD$) : δ (ppm) = 4.96 (m, 1H), 3.39 (m, 1H), 2.79-2.66 (m, 9H), 2.41-2.17 (m, 5H), 2.05-0.87 (m, 42H), 0.71 (s, 3H). **NMR $^{13}$C** (63 MHz, $CD_3OD$) : δ (ppm) = 175.72, 72.04, 69.03, 57.84, 57.71, 57.60, 56.71, 53.81, 48.29, 45.67, 44.93, 44.82, 44.67, 41.70, 40.51, 36.76, 36.73, 35.72, 32.72, 32.49, 31.92, 30.92, 30.63, 29.79, 28.70, 28.06, 22.22, 19.03, 12.77. **MS** ($ESI^+$) : m/z 548.4751 ($[M+H]^+$).Yield : 15%

**3β-(Tris(3-aminopropyl)amine)-isopropyl ursodesoxycholate D8** ($C_{36}H_{68}N_4O_3$)

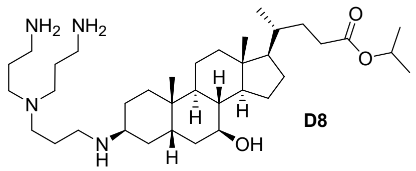

D8

**NMR $^1$H** (250 MHz, $CD_3OD$) : δ (ppm) = 4.96 (m, 1H), 3.38 (m, 1H), 2.89-2.47 (m, 13H), 2.35-0.87 (m, 50H), 0.71 (s, 3H). **NMR $^{13}$C** (63 MHz, $CD_3OD$) : δ (ppm) = 175.64, 72.07, 68.99, 59.02, 57.73, 57.63, 56.74, 56.72, 53.51, 52.96, 44.94, 44.84, 44.68, 41.23, 36.82, 36.76, 35.80, 32.72, 32.49, 30.90, 30.82, 29.79, 28.08, 25.44, 24.31, 22.25, 19.08, 12.82. **MS** ($ESI^+$) : m/z 605.5335 ($[M+H]^+$).Yield : 26%

**3β-(pentaethylenehexamine)-isopropyl ursodesoxycholate D9** ($C_{37}H_{72}N_6O_3$)

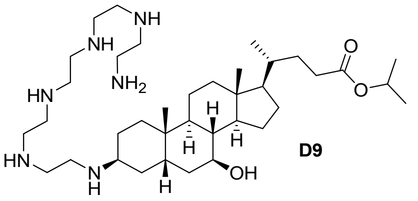

D9

**NMR $^{1}$H** (250 MHz, $CD_3OD$) : δ (ppm) = 4.96 (m, 1H), 3.47 (m, 1H), 2.98-2.45 (m, 21H), 2.32-2.17 (m, 3H), 2.06-1.05 (m, 36H), 0.99-0.94 (m, 5H), 0.71 (s, 3H). **NMR $^{13}$C** (63 MHz, $CD_3OD$) : δ (ppm) = 175.55, 72.01, 68.95, 61.54, 58.88, 58.63, 57.63, 56.73, 55.22, 54.27, 47.10, 46.92, 46.38, 46.23, 44.94, 44.66, 44.50, 42.20, 41.95, 41.71, 40.81, 39.13, 38.90, 37.76, 35.79, 35.57, 32.71, 32.50, 29.83, 28.40, 28.09, 24.34, 22.54, 22.28, 19.10, 12.85. **MS** ($ESI^+$) : m/z 649.5 ($[M+H]^+$).Yield : 34%

**3β-(Tris(2-aminoéthyl)amine)-isopropyl ursodesoxycholate D10** ($C_{33}H_{62}N_4O_3$)

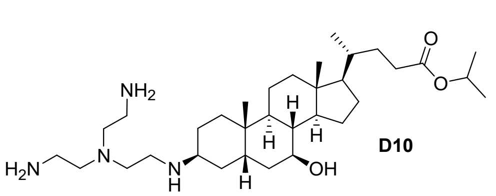

D10

**NMR $^{1}$H** (250 MHz, $CD_3OD$) : δ (ppm) = 4.96 (m, 1H), 3.45 (m, 1H), 2.82-2.50 (m, 13H), 2.36-2.14 (m, 3H), 2.06-1.05 (m, 34H), 0.99-0.94 (m, 7H), 0.71 (s, 3H). **NMR $^{13}$C** (63 MHz, $CD_3OD$) : δ (ppm) = 175.63, 72.05, 68.98, 59.04, 58.08, 57.66, 56.74, 55.19, 45.37, 44.94, 44.68, 44.50, 41.72, 40.83, 40.40, 40.30, 38.87, 36.76, 35.79, 35.56, 32.72, 32.50, 29.81, 28.39, 28.08, 24.30, 22.54, 22.25, 19.07, 12.82. **MS** ($ESI^+$) : m/z 563.5 ($[M+H]^+$). Yield : 34%

**3β-(Bis(3-aminopropyl)methylamine)-isopropyl ursodesoxycholate D11** ($C_{34}H_{63}N_3O_3$)

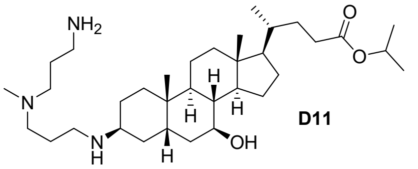

D11

**NMR $^{1}$H** (250 MHz, $CD_3OD$) : δ (ppm) = 4.96 (m, 1H), 3.46 (m, 1H), 2.91-2.20 (m, 14H), 2.07-0.88 (m, 44H), 0.72 (s, 3H). **NMR $^{13}$C** (63 MHz, $CD_3OD$) : δ (ppm) = 175.71, 71.88, 69.03, 58.96, 57.56, 56.74, 56.61, 53.38, 44.93, 44.63, 44.16, 42.08, 41.63, 40.74, 40.38, 38.50, 36.75, 35.57, 32.72, 32.48, 29.78, 28.94, 28.05, 27.17, 26.39, 26.00, 23.99, 22.51, 22.22, 19.02, 12.76. **MS** ($ESI^+$) : m/z 562.4 ($[M+H]^+$).Yield : 12%

**3β-(1,3-diaminopropane)-methyl ursodesoxycholate D12** ($C_{28}H_{50}N_2O_3$)

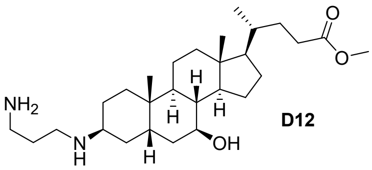

D12

**NMR $^{1}$H** (250 MHz, $CD_3OD$) : δ (ppm) = 3.62 (s, 3H), 3.45 (m, 1H), 2.82-2.55 (m, 5H), 2.45-2.14 (m, 3H), 2.03-0.92 (m, 35H), 0.69 (m, 3H). **NMR $^{13}$C** (63 MHz, $CD_3OD$) : δ (ppm) = 176.43, 72.01, 58.95, 57.60, 56.66, 52.18, 45.42, 44.92, 44.65, 44.52, 41.70, 40.82, 38.91, 36.84, 35.80, 35.45, 33.91, 32.42, 32.01, 29.81, 28.29, 28.10, 24.35, 22.55, 19.11, 12.83. **MS** ($ESI^+$) : m/z 463.4 ($[M+H]^+$).Yield : 68%

**3β-(Tris(3-aminopropyl)amine)-methyl ursodesoxycholate D13** ($C_{34}H_{64}N_4O_3$)

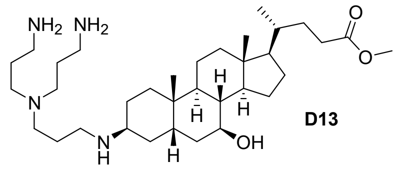

D13

**NMR $^{1}$H** (250 MHz, $CD_3OD$) : δ (ppm) = 3.65 (m, 3H), 3.46 (m, 1H), 2.86-2.48 (m, 13H), 2.37-0.94 (m, 44H), 0.71 (s, 3H). **NMR $^{13}$C** (63 MHz, $CD_3OD$) : δ (ppm) = 176.44, 72.00, 59.00, 57.65, 56.68, 53.39, 52.87, 52.18, 46.33, 44.92, 44.64, 44.44, 41.70, 41.06, 40.80, 38.86, 37.33, 37.06, 36.83, 35.78, 35.21, 32.43, 32.00, 30.22, 29.81, 28.10, 27.50, 24.32, 22.55, 19.10, 12.83. **MS** ($ESI^+$) : m/z 577.5 ($[M+H]^+$).Yield : 43%

**3β-(pentaethylenehexamine)-methyl ursodesoxycholate D14** ($C_{35}H_{68}N_6O_3$)

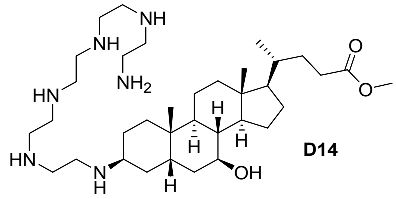

D14

**NMR $^{1}$H** (250 MHz, $CD_3OD$) : δ (ppm) = 3.65 (s, 3H), 3.46 (m, 1H), 2.85-2.54 (m, 21H), 2.37-2.20 (m, 3H), 2.06-0.94 (m, 37H), 0.72 (s, 3H). **NMR $^{13}$C** (63 MHz, $CD_3OD$) : δ (ppm) = 176.47, 72.02, 58.88, 58.58, 57.64, 56.68, 54.29, 54.23, 52.17, 44.92, 44.65, 44.46, 41.80, 41.73, 40.81, 38.88, 36.83, 36.73, 35.77, 35.43, 32.42, 32.00, 29.81, 28.31, 28.10, 24.30, 22.54, 19.08, 12.80. **MS** ($ESI^+$) : m/z 621.5 ($[M+H]^+$).Yield : 33%

**3β-(Bis(3-aminopropyl)methylamine)-methyl ursodesoxycholate D15** ($C_{32}H_{59}N_3O_3$)

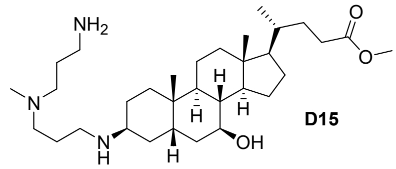

D15

**NMR $^{1}$H** (250 MHz, $CD_3OD$) : δ (ppm) = 3.65 (s, 3H), 3.47 (m, 1H), 2.84-2.30 (m, 12H), 2.24-0.94 (m, 40H), 0.71 (s, 3H). **NMR $^{13}$C** (63 MHz, $CD_3OD$) : δ (ppm) = 176.34, 71.98, 58.93, 57.66, 57.59, 57.04, 56.57, 52.18, 46.21, 44.90, 44.63, 44.47, 42.48, 41.69, 41.08, 40.77, 38.90, 36.82, 35.78, 35.37, 32.42, 31.99, 30.82, 29.82, 28.10, 27.85, 24.36, 22.54, 19.13, 12.86. **MS** ($ESI^+$) : m/z 534.4591 ($[M+H]^+$).Yield : 57%

**3β-(1,4-diaminobutane)-methyl ursodesoxycholate D16**

($C_{29}H_{52}N_2O_3$)

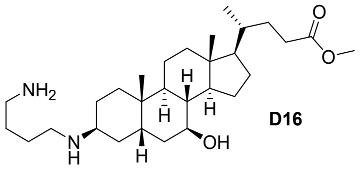

D16

**NMR $^1$H** (250 MHz, $CD_3OD$) : δ (ppm) = 3.65 (s, 3H), 3.48 (m, 1H), 2.84-2.41 (m, 5H), 2.37-2.18 (m, 2H), 2.06-0.94 (m, 38H), 0.71 (s, 3H). **NMR $^{13}$C** (63 MHz, $CD_3OD$) : δ (ppm) = 176.50, 72.05, 58.87, 57.60, 56.67, 52.17, 47.60, 44.92, 44.65, 44.50, 42.51, 41.70, 40.80, 38.88, 36.83, 35.79, 35.40, 32.42, 32.00, 31.71, 29.80, 28.20, 28.09, 24.44, 24.30, 22.54, 19.07, 12.80. **MS** ($ESI^+$) : m/z 477.4 ($[M+H]^+$).Yield : 30%

**3β-(1,6-diaminohexane)-methyl ursodesoxycholate D17**

($C_{31}H_{56}N_2O_3$)

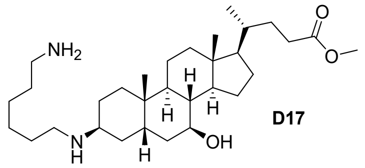

D17

**NMR $^1$H** (250 MHz, $CD_3OD$) : δ (ppm) = 3.65 (s, 3H), 3.48 (m, 1H), 2.84-2.41 (m, 5H), 2.37-0.94 (m, 44H), 0.71 (s, 3H). **NMR $^{13}$C** (63 MHz, $CD_3OD$) : δ (ppm) = 176.40, 72.01, 58.94, 57.69, 57.58, 56.65, 52.18, 47.78, 44.91, 44.64, 44.50, 42.55, 41.70, 40.77, 37.33, 36.83, 35.79, 35.33, 33.68, 32.42, 32.00, 30.76, 29.82, 28.53, 28.08, 24.36, 22.57, 19.13, 12.85. **MS** ($ESI^+$) : m/z 505.4 ($[M+H]^+$).Yield : 47%

**3β-spermino-isopropyl ursodesoxycholate D18**

($C_{37}H_{70}N_4O_3$)

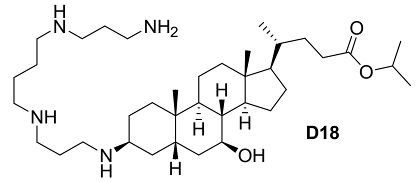

D18

**NMR $^1$H** (250 MHz, $CD_3OD$) : δ (ppm) = 4.96 (m, 1H), 3.47 (m, 1H), 2.85-2.49 (m, 13H), 2.38-2.14 (m, 3H), 2.06-0.94 (m, 49H), 0.71 (s, 3H). **NMR $^{13}$C** (63 MHz, $CD_3OD$) : δ (ppm) = 175.55, 72.00, 68.95, 58.89, 57.61, 56.73, 50.60, 48.34, 45.85, 44.93, 44.65, 44.50, 41.70, 40.78, 40.70, 38.88, 36.77, 35.78, 35.29, 33.05, 32.70, 32.50, 30.25, 29.84, 28.28, 28.15, 28.10, 24.33, 22.56, 22.29, 19.10, 12.84. **MS** ($ESI^+$) : m/z 619.5480 ($[M+H]^+$).Yield : 37%

**3β-norspermino-isopropyl ursodesoxycholate D19**

($C_{36}H_{68}N_4O_3$)

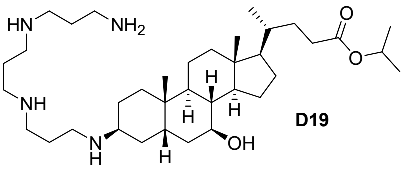

D19

**NMR $^{1}$H** (250 MHz, $CD_3OD$) : δ (ppm) = 4.96 (m, 1H), 3.47 (m, 1H), 2.87-2.51 (m, 14H), 2.36-2.14 (m, 2H), 2.06-0.94 (m, 47H), 0.71 (s, 3H). **NMR $^{13}$C** (63 MHz, $CD_3OD$) : δ (ppm) = 175.61, 72.02, 68.98, 58.88, 57.62, 56.73, 48.92, 48.40, 45.85, 44.93, 44.65, 44.48, 41.70, 40.79, 40.67, 38.85, 36.77, 35.86, 35.76, 35.22, 35.17, 32.83, 32.71, 32.50, 30.13, 30.00, 29.83, 28.08, 24.29, 22.54, 22.26, 19.07, 12.81. **MS** ($ESI^+$) : m/z 605.5 ($[M+H]^+$).Yield : 32%

**3β-(1,4-Bis(3-aminopropyl)piperazine)-isopropyl ursodesoxycholate D20** ($C_{37}H_{68}N_4O_3$)

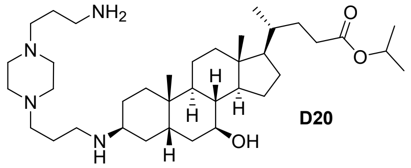


D20

**NMR $^{1}$H** (250 MHz, $CD_3OD$) : δ (ppm) = 4.96 (m, 1H), 3.48 (m, 1H), 2.84-2.38 (m, 17H), 2.32-0.94 (m, 46H), 0.71 (s, 3H). **NMR $^{13}$C** (63 MHz, $CD_3OD$) : δ (ppm) = 175.46, 71.97, 68.91, 58.85, 58.07, 57.63, 57.52, 56.71, 54.13, 54.03, 46.40, 44.93, 44.63, 44.48, 41.71, 41.16, 40.80, 38.93, 36.76, 35.78, 35.51, 32.69, 32.48, 30.60, 29.85, 28.37, 28.08, 27.40, 24.55, 24.38, 22.55, 22.31, 19.14, 12.87. **MS** ($ESI^+$) : m/z 617.5325 ($[M+H]^+$).Yield : 77%

- ## Claramines E

**3β-spermino-isopropyl lithocholate E1** ($C_{37}H_{70}N_4O_2$)

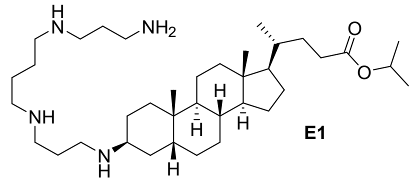


E1

**NMR $^{1}$H** (250 MHz, $CD_3OD$) : δ (ppm) = 4.96 (m, 1H), 2.89-2.48 (m, 13H), 2.36-2.14 (m, 3H), 2.03-0.93 (m, 50H), 0.70 (s, 3H). **NMR $^{13}$C** (63 MHz, $CD_3OD$) : δ (ppm) = 175.55, 68.97, 59.13, 58.09, 57.77, 50.63, 49.03, 46.05, 44.15, 42.11, 41.73, 40.90, 38.40, 37.48, 37.28, 36.72, 36.35, 34.83, 34.73, 33.53, 32.85, 32.50, 30.57, 29.32, 28.64, 28.45, 27.85, 25.42, 24.32, 22.27, 22.17, 19.01, 12.75. **MS** ($ESI^+$) : m/z 603.6 ($[M+H]^+$). Yield : 41%

**3β-norspermino-isopropyl lithocholate E2**
($C_{36}H_{68}N_4O_2$)

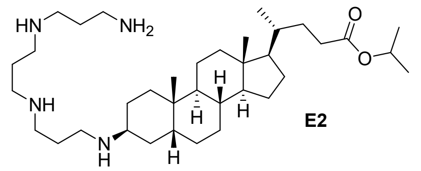


**NMR $^{1}$H** (250 MHz, $CD_3OD$) : δ (ppm) = 4.98 (m, 1H), 2.95-2.63 (m, 13H), 2.44-2.17 (m, 3H), 2.03-0.90 (m, 48H), 0.69 (s, 3H). **NMR $^{13}$C** (63 MHz, $CD_3OD$) : δ (ppm) = 175.58, 68.98, 59.04, 58.01, 57.62, 52.18, 48.88, 48.37, 44.06, 43.90, 41.94, 41.65, 40.63, 37.36, 37.03, 36.84, 36.79, 36.35, 36.25, 32.68, 32.63, 32.43, 32.36, 31.98, 29.92, 29.42, 28.54, 27.80, 25.43, 24.29, 22.67, 22.27, 22.11, 18.94, 12.69. **MS** ($ESI^+$) : m/z 589.5 ($[M+H]^+$). Yield : 36%

Mélange 50/50 de **3β-spermidino-isopropyl lithocholate E3a** et de **3β-*N*-[4'*N*-(3'aminopropyl)aminobutyl]amino-isopropyl lithocholate E3b** ($C_{34}H_{63}N_3O_2$)

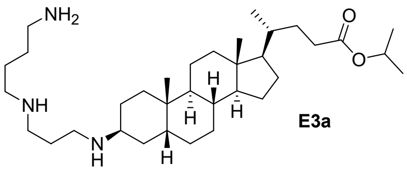


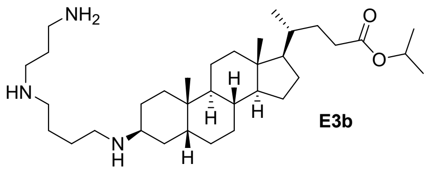


**NMR $^{1}$H** (250 MHz, $CD_3OD$) : δ (ppm) = 4.97 (m, 1H), 2.90-2.61 (m, 8H), 2.36-2.20 (m, 3H), 2.03-0.96 (m, 48H), 0.69 (s, 3H). **NMR $^{13}$C** (63 MHz, $CD_3OD$) : δ (ppm) = 175.60, 68.98, 59.04, 58.03, 57.65, 52.18, 49.25, 44.08, 43.93, 41.97, 431.67, 42.35, 41.97, 41.67, 37.36, 37.23, 37.11, 36.79, 36.28, 32.68, 32.45, 32.37, 31.98, 29.44, 29.38, 28.62, 28.59, 27.89, 27.83, 25.49, 25.43, 24.38, 24.32, 22.28, 22.12, 18.93, 12.71. Yield : 38%

**3β-norspermidino-isopropyl lithocholate E4**
($C_{33}H_{61}N_3O_2$)

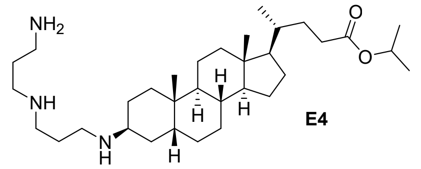


**NMR $^{1}$H** (250 MHz, $CD_3OD$) : δ (ppm) = 4.96 (m, 1H), 2.97-2.55 (m, 8H), 2.36-2.13 (m, 3H), 2.04-0.93 (m, 46H), 0.69 (s, 3H). **NMR $^{13}$C** (63 MHz, $CD_3OD$) : δ (ppm) = 175.58, 68.98, 59.08, 58.03, 57.63, 52.16, 48.96, 47.96, 44.07, 43.94, 41.96, 41.67, 40.71, 38.20, 37.38, 37.22, 37.03, 36.78, 36.28, 34.37, 32.69, 32.45, 29.41, 28.57, 28.53, 25.44, 24.32, 22.27, 22.13, 18.96, 12.72. **MS** ($ESI^+$) : m/z 532.4 ($[M+H]^+$). Yield : 41%

**3β-(1,4-Bis(3-aminopropyl)piperazine)-isopropyl lithocholateE5** ($C_{37}H_{68}N_4O_2$)

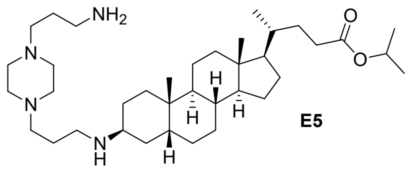


**NMR $^1$H** (250 MHz, $CD_3OD$) : δ (ppm) = 4.96 (m, 1H), 2.88-2.37 (m, 18H), 2.32-0.93 (m, 46H), 0.70 (s, 3H). **NMR $^{13}$C** (63 MHz, $CD_3OD$) : δ (ppm) = 175.58, 68.98, 59.04, 58.12, 57.79, 57.50, 54.27, 54.16, 52.06, 46.43, 44.14, 44.03, 42.11, 41.73, 41.22, 37.44, 37.19, 36.76, 36.30, 32.83, 32.42, 30.34, 30.25, 29.29, 28.59, 27.80, 27.11, 25.40, 22.24, 22.14, 18.99, 12.79. **MS** ($ESI^+$) : m/z 601.6 ($[M+H]^+$). Yield : 43%

**3β-spermino-methyl lithocholate E6** ($C_{35}H_{66}N_4O_2$)

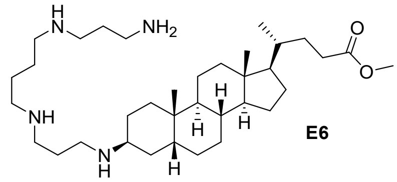


**NMR $^1$H** (250 MHz, $CD_3OD$) : δ (ppm) = 3.65 (s, 3H), 2.94-2.64 (m, 13H), 2.41-2.20 (m, 3H), 2.03-0.87 (m, 44H), 0.71 (s, 3H). **NMR $^{13}$C** (63 MHz, $CD_3OD$) : δ (ppm) = 176.46, 59.11, 58.12, 57.75, 52.06, 50.45, 50.42, 46.01, 44.13, 42.13, 41.74, 40.86, 38.40, 37.47, 37.17, 36.96, 36.77, 34.49, 33.52, 32.86, 32.44, 32.15, 31.71, 30.35, 29.27, 28.57, 28.28, 25.39, 24.22, 22.22, 18.96, 12.69. **MS** ($ESI^+$) : m/z 575.5215 ($[M+H]^+$).Yield : 30%

**3β-norspermino-methyl lithocholate E7** ($C_{34}H_{64}N_4O_2$)

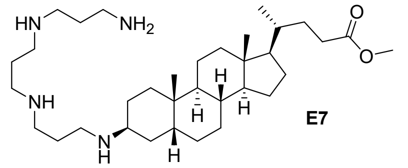


**NMR $^1$H** (250 MHz, $CD_3OD$) : δ (ppm) = 3.65 (s, 3H), 2.93-2.57 (m, 13H), 2.43-2.17 (m, 3H), 2.03-0.92 (m, 42H), 0.69 (s, 3H). **NMR $^{13}$C** (63 MHz, $CD_3OD$) : δ (ppm) = 176.86, 59.05, 58.05, 57.67, 54.32, 52.17, 48.97, 44.09, 41.97, 41.68, 40.72, 38.19, 37.39, 36.85, 36.30, 34.41, 33.57, 33.24, 32.37, 31.99, 31.49, 30.38, 29.41, 28.62, 28.27, 27.86, 25.48, 24.36, 22.34, 22.13, 19.09, 12.77. **MS** ($ESI^+$) : m/z 561.5 ($[M+H]^+$).Yield : 36%

Mélange 50/50 de **3β-spermidino-methyl lithocholate E8a** et de **3β-*N*-[4'*N*-(3'aminopropyl)aminobutyl]amino-methyl lithocholate E8b** ($C_{32}H_{59}N_3O_2$)

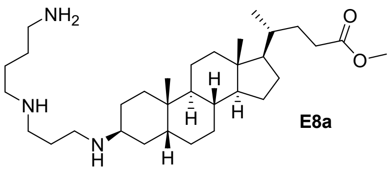
E8a

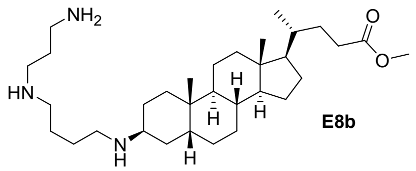
E8b

**NMR $^{1}$H** (250 MHz, $CD_3OD$) : δ (ppm) = 3.65 (m, 3H), 2.91-2.64 (m, 8H), 2.43-2.17 (m, 3H), 2.03-0.93 (m, 42H), 0.69 (s, 3H). **NMR $^{13}$C** (63 MHz, $CD_3OD$) : δ (ppm) = 176.37, 59.03, 58.01, 57.56, 56.25, 53.84, 52.18, 50.46, 45.67, 44.06, 43.93, 42.25, 41.95, 41.65, 41.48, 38.12, 37.37, 37.21, 37.11, 36.84, 36.28, 34.32, 32.36, 31.98, 31.43, 31.32, 31.06, 29.42, 28.60, 28.24, 27.83, 27.05, 25.46, 24.35, 22.35, 22.13, 18.98, 12.74. Yield : 51%

**3β-norspermidino-methyl lithocholate E9**
($C_{31}H_{57}N_3O_2$)

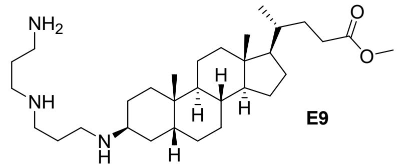
E9

**NMR $^{1}$H** (250 MHz, $CD_3OD$) : δ (ppm) = 3.65 (s, 3H), 2.71-2.56 (m, 9H), 2.23-2.17 (m, 2H), 0.93 (m, 40H), 0.69 (s, 3H). **NMR $^{13}$C** (63 MHz, $CD_3OD$) : δ (ppm) = 176.45, 59.07, 58.02, 57.58, 52.17, 49.04, 45.93, 44.06, 41.97, 41.67, 40.79, 38.23, 37.40, 37.24, 37.19, 36.84, 36.31, 34.59, 33.56, 32.37, 32.01, 30.54, 29.41, 28.62, 28.43, 27.84, 25.45, 24.36, 22.13, 18.95. **MS** ($ESI^+$) : m/z 504.4 ($[M+H]^+$). Yield : 28%

**3β-(1,4-Bis(3-aminopropyl)piperazine)-methyl lithocholate E10** ($C_{35}H_{64}N_4O_2$)

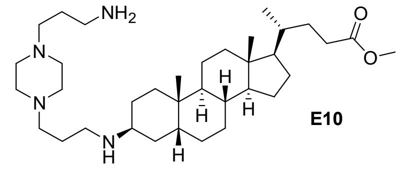
E10

**NMR $^{1}$H** (250 MHz, $CD_3OD$) : δ (ppm) = 3.65 (s, 3H), 2.97-2.17 (m, 19H), 2.03-0.93 (m, 39H), 0.69 (s, 3H). **NMR $^{13}$C** (63 MHz, $CD_3OD$) : δ (ppm) = 176.43, 59.03, 58.12, 57.70, 57.33, 54.25, 54.15, 52.08, 46.46, 44.14, 44.05, 42.13, 41.75, 41.24, 37.47, 37.22, 36.77, 36.32, 32.43, 32.15, 30.45, 29.30, 28.61, 27.82, 27.62, 27.37, 25.41, 24.27, 22.26, 18.99, 12.72. **MS** ($ESI^+$) : m/z 573.5 ($[M+H]^+$).Yield : 61%

**3β-(éthylènediamine)-methyl lithocholate E11**
($C_{27}H_{48}N_2O_2$)

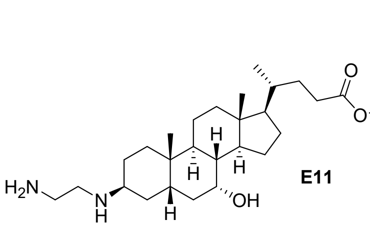
E11

**NMR $^{1}$H** (250 MHz, $CD_3OD$) : δ (ppm) = 3.65 (s, 3H), 2.87-2.62 (m, 3H), 2.50-2.17 (m, 3H), 2.03, 0.93 (m, 36H), 0.70 (s, 3H). **NMR $^{13}$C** (63 MHz, $CD_3OD$) : δ (ppm) = 176.49, 59.02, 58.74, 58.02, 57.55, 54.35, 52.20, 50.09, 44.06, 42.25, 41.97, 41.65, 37.38, 37.22,

36.84, 36.30, 32.36, 31.99, 29.41, 28.61, 27.83, 25.44, 24.36, 22.12, 18.97, 12.72. **MS** ($ESI^+$) : m/z 449.4 ($[M+H]^+$).Yield : 57%

**3β-(1,3-diaminopropane)-methyl lithocholate E12** ($C_{28}H_{50}N_2O_2$)

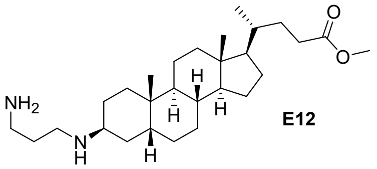

E12

**NMR $^1$H** (250 MHz, $CD_3OD$) : δ (ppm) = 3.65 (s, 3H), 2.88-2.46 (m, 5H), 2.37-2.17 (m, 2H), 2.03-0.93 (m, 37H), 0.69 (s, 3H). **NMR $^{13}$C** (63 MHz, $CD_3OD$) : δ (ppm) = 176.34, 59.10, 57.99, 57.55, 52.18, 45.44, 44.06, 41.94, 41.65, 40.84, 37.39, 37.21, 36.85, 36.32, 34.59, 33.89, 33.66, 32.36, 31.98, 29.43, 28.85, 28.44, 27.86, 24.47, 24.22, 22.15, 18.98, 12.76. **MS** ($ESI^+$) : m/z 447.4 ($[M+H]^+$).Yield : 56%

**3β-(1,4-diaminobutane)-methyl lithocholate E13** ($C_{29}H_{52}N_2O_2$)

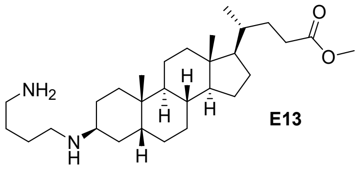

E13

**NMR $^1$H** (250 MHz, $CD_3OD$) : δ (ppm) = 3.65 (m, 3H), 2.90-2.53 (m, 4H), 2.43-2.17 (m, 3H), 2.01-0.87 (m, 39H), 0.69 (s, 3H). **NMR $^{13}$C** (63 MHz, $CD_3OD$) : δ (ppm) = 176.46, 58.98, 58.01, 57.58, 52.18, 47.32, 44.04, 42.22, 42.13, 41.95, 41.63, 37.36, 37.19, 37.05, 36.83, 36.35, 36.26, 32.36, 31.98, 29.38, 27.94, 27.83, 27.80, 25.43, 24.44, 24.30, 22.11, 18.95, 12.70. **MS** ($ESI^+$) : m/z 461.4165 ($[M+H]^+$).Yield : 35%

**3β-(1,5-diaminopentane)-methyl lithocholate E14** ($C_{30}H_{54}N_2O_2$)

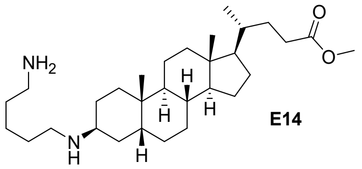

E14

**NMR $^1$H** (250 MHz, $CD_3OD$) : δ (ppm) = 3.65 (s, 3H), 2.89-2.48 (m, 5H), 2.37-0.94 (m, 43H), 0.69 (s, 3H). **NMR $^{13}$C** (63 MHz, $CD_3OD$) : δ (ppm) = 176.77, 58.96, 58.09, 57.64, 52.17, 44.06, 42.91, 42.43, 40.40, 37.86, 37.22, 37.00, 36.83, 36.38, 34.35, 33.59, 33.39, 33.32, 33.23, 32.38, 32.00, 30.43, 29.42, 25.80, 25.42, 24.44, 22.65, 19.09, 12.72. **MS** ($ESI^+$) : m/z 475.4215 ($[M+H]^+$).Yield : 33%

**3β-(1,6-diaminohexane)-methyl lithocholate E15** ($C_{31}H_{56}N_2O_2$)

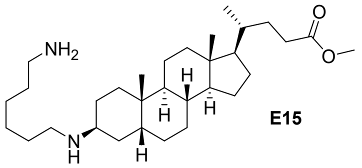

E15

**NMR $^1$H** (250 MHz, $CD_3OD$) : δ (ppm) = 3.65 (s, 3H), 2.83-2.67 (m, 3H), 2.41-0.93 (m, 47H), 0.69 (s, 3H). **NMR $^{13}$C** (63 MHz, $CD_3OD$) : δ (ppm) = 176.49, 59.10, 58.04, 57.60, 52.18, 49.39, 47.02, 44.06, 43.75, 41.92, 41.75, 41.64, 41.49, 37.33, 37.02, 36.84, 36.17, 34.33, 34.24, 33.59, 32.35, 31.99, 29.51, 29.41, 27.72, 25.45, 24.47, 22.11, 19.07, 12.68. **MS** ($ESI^+$) : m/z 489.4325 ($[M+H]^+$).Yield : 59%

**3β-(pentaethylenehexamine)-methyl lithocholate E16** ($C_{35}H_{68}N_6O_2$)

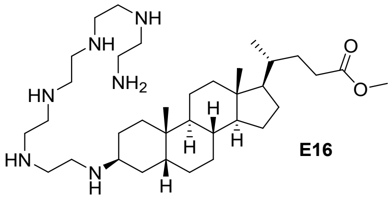

E16

**NMR $^1$H** (250 MHz, $CD_3OD$) : δ (ppm) = 3.65 (m, 3H), 2.84-2.47 (m, 23H), 2.37-2.20 (3H), 2.03-0.93 (s, 36H), 0.69 (s, 3H). **NMR $^{13}$C** (63 MHz, $CD_3OD$) : δ (ppm) = 176.42, 59.03, 58.05, 57.57, 55.35, 55.18, 54.30, 54.07, 52.17, 44.06, 43.93, 41.93, 41.67, 39.05, 37.37, 37.11, 36.84, 36.29, 32.36, 31.98, 29.41, 28.59, 27.83, 25.44, 24.33, 22.33, 22.11, 18.93, 12.69. **MS** ($ESI^+$) : m/z 605.5 ($[M+H]^+$).Yield : 32%

**3β-(Bis(3-aminopropyl)methylamine)-lithocholate de méthyle E17** ($C_{32}H_{59}N_3O_2$)

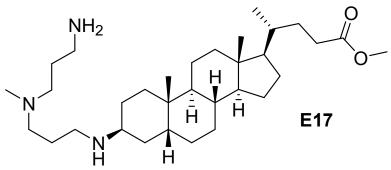

E17

**NMR $^1$H** (250 MHz, $CD_3OD$) : δ (ppm) = 3.65 (m, 3H), 2.70-2.18 (m, 15H), 2.03-0.93 (m, 38H), 0.70 (s, 3H). **NMR $^{13}$C** (63 MHz, $CD_3OD$) : δ (ppm) = 176.31, 59.10, 58.00, 57.55, 57.09, 56.59, 52.18, 44.05, 43.93, 42.62, 42.48, 41.94, 41.64, 41.08, 37.37, 37.21, 36.84, 36.30, 32.36, 31.98, 30.79, 29.43, 28.62, 28.35, 27.84, 25.47, 24.39, 22.13, 19.01, 12.75. **MS** ($ESI^+$) : m/z 518.4 ($[M+H]^+$).Yield : 68%

**3β-(Tris(3-aminopropyl)amine)-lithocholate de méthyle E18** ($C_{34}H_{64}N_4O_2$)

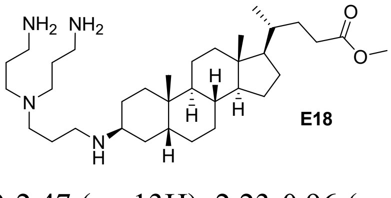

E18

**NMR $^1$H** (250 MHz, $CD_3OD$) : δ (ppm) = 3.65 (m, 3H), 2.70-2.47 (m, 13H), 2.23-0.96 (m, 45H), 0.69 (s, 3H). **NMR $^{13}$C** (63 MHz, $CD_3OD$) : δ (ppm) = 176.51, 59.18, 58.09, 57.71,

52.96, 52.16, 44.08, 42.00, 41.69, 41.16, 38.90, 37.39, 37.05, 36.84, 36.31, 34.40, 33.59, 32.39, 32.00, 30.61, 29.44, 27.90, 25.47, 24.47, 22.16, 19.15, 12.77. **MS** ($ESI^+$) : m/z 561.5063 ($[M+H]^+$).Yield : 41%

**3β-(pentaethylenehexamine)-isopropyl lithocholate E20** ($C_{37}H_{72}N_6O_2$)

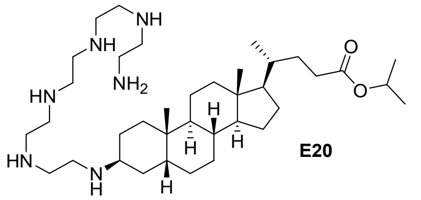


**NMR $^1$H** (250 MHz, $CD_3OD$) : δ (ppm) = 4.96 (m, 1H), 2.90-2.45 (m, 25H), 2.30-2.14 (m, 2H), 2.04-0.93 (m, 41H), 0.69 (s, 3H). **NMR $^{13}$C** (63 MHz, $CD_3OD$) : δ (ppm) = 175.59, 68.98, 59.10, 58.64, 58.07, 57.64, 55.38, 55.22, 54.50, 54.09, 46.38, 46.23, 44.07, 43.95, 42.00, 41.95, 41.67, 39.11, 37.39, 37.24, 37.13, 36.78, 36.30, 34.68, 32.69, 32.44, 29.41, 28.59, 27.83, 25.42, 24.32, 22.26, 22.11, 18.92, 12.69. **MS** ($ESI^+$) : m/z 633.5760 ($[M+H]^+$).Yield : 24%

**3β-(Tris(3-aminopropyl)amine)-isopropyl lithocholate E21** ($C_{36}H_{68}N_4O_2$)

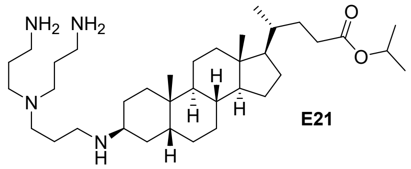


**NMR $^1$H** (250 MHz, $CD_3OD$) : δ (ppm) = 4.96 (m, 1H), 2.89-2.47 (m, 13H), 2.38-2.15 (m, 3H), 2.03-0.93 (m, 48H), 0.69 (s, 3H). **NMR $^{13}$C** (63 MHz, $CD_3OD$) : δ (ppm) = 175.44, 68.91, 59.16, 58.02, 57.60, 54.37, 53.50, 52.93, 46.41, 44.07, 43.92, 41.97, 41.65, 41.15, 37.38, 37.22, 36.77, 36.36, 36.30, 34.51, 32.69, 30.60, 29.43, 28.62, 28.37, 27.84, 27.65, 25.45, 24.55, 24.40, 22.32, 22.14, 18.98, 12.78. **MS** ($ESI^+$) : m/z 589.5 ($[M+H]^+$).Yield : 55%

**3β-(Tris(2-aminoéthyl)amine)-isopropyl lithocholateE22** ($C_{33}H_{62}N_4O_2$)

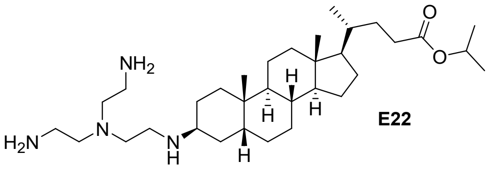


**NMR $^1$H** (250 MHz, $CD_3OD$) : δ (ppm) = 4.96 (m, 1H), 2.83-2.56 (m, 12H), 2.38-2.17 (m, 3H), 2.04-0.90 (m, 43H), 0.69 (s, 3H). **NMR $^{13}$C** (63 MHz, $CD_3OD$) : δ (ppm) = 175.55, 68.95, 59.12, 58.05, 57.63, 57.25, 54.67, 45.17, 44.07, 43.88, 42.11, 41.99, 41.66, 40.07, 37.36, 37.20, 37.03, 36.76, 36.36, 36.25, 34.20, 32.69, 32.43, 29.41, 27.82, 25.42, 24.28, 22.28, 22.11, 18.93, 12.71. **MS** ($ESI^+$) : m/z 547.5 ($[M+H]^+$). Yield : 38%

**3β-(Bis(3-aminopropyl)methylamine)-isopropyl lithocholateE23** ($C_{34}H_{63}N_3O_2$)

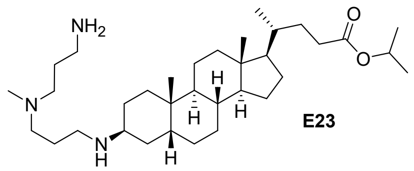


**NMR $^1$H** (250 MHz, $CD_3OD$) : δ (ppm) = 4.96 (m, 1H), 2.73-2.12 (m, 14H), 2.03-0.93 (m, 45H), 0.69 (s, 3H). **NMR $^{13}$C** (63 MHz, $CD_3OD$) : δ (ppm) = 175.56, 68.98, 59.10, 58.02, 57.62, 57.03, 56.57, 46.17, 44.07, 43.91, 42.42, 41.97, 41.65, 41.00, 37.37, 37.09, 36.77, 36.27, 34.30, 32.69, 32.43, 30.34, 29.42, 28.57, 28.17, 27.81, 27.61, 25.44, 24.33, 22.29, 22.13, 18.96, 12.73. **MS** ($ESI^+$) : m/z 532.4796 ($[M+H]^+$).Yield : 43%

## $^{13}$C NMR spectra of Claramine derivatives A-E

**A.** $^1$H NMR spectrum for the claramine **A1** in $CD_3OD$ (250.13 MHz)

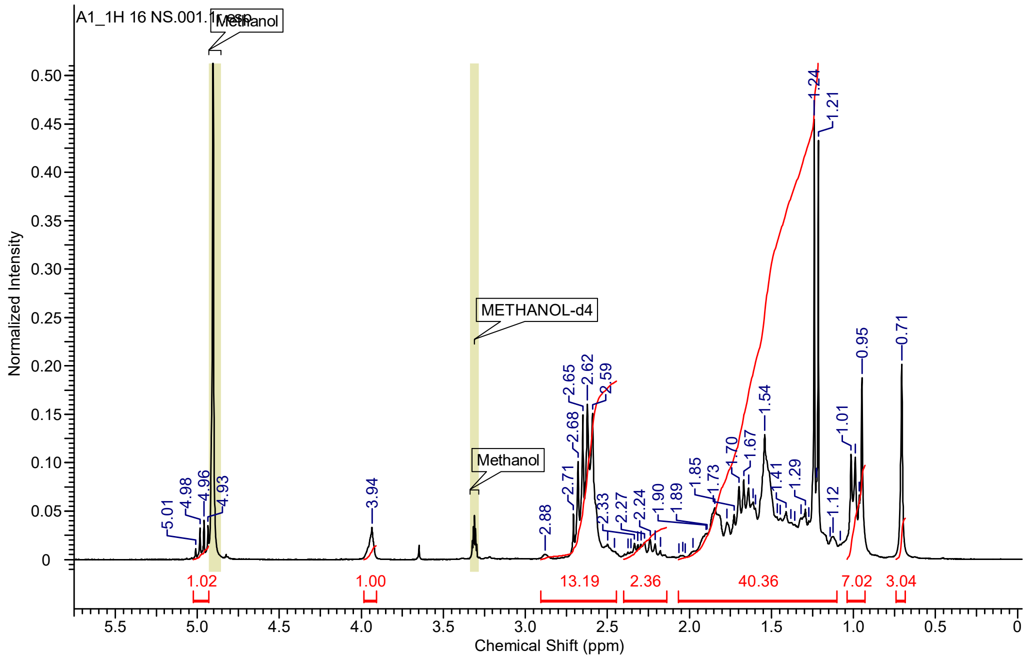


**B.** $^{13}$C NMR spectrum for the claramine **A1** in $CD_3OD$ (62.90 MHz)

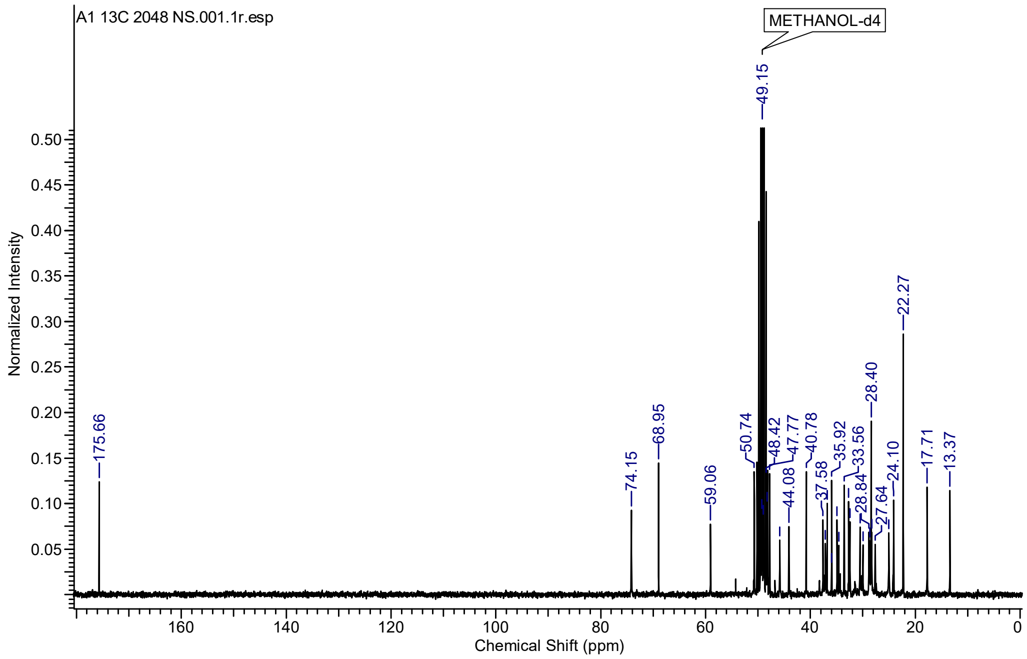


**C.** COSY spectrum for the claramine **A1** in $CD_3OD$ ($^1H$-$^1H$ ; 250.13, 250.13 MHz)

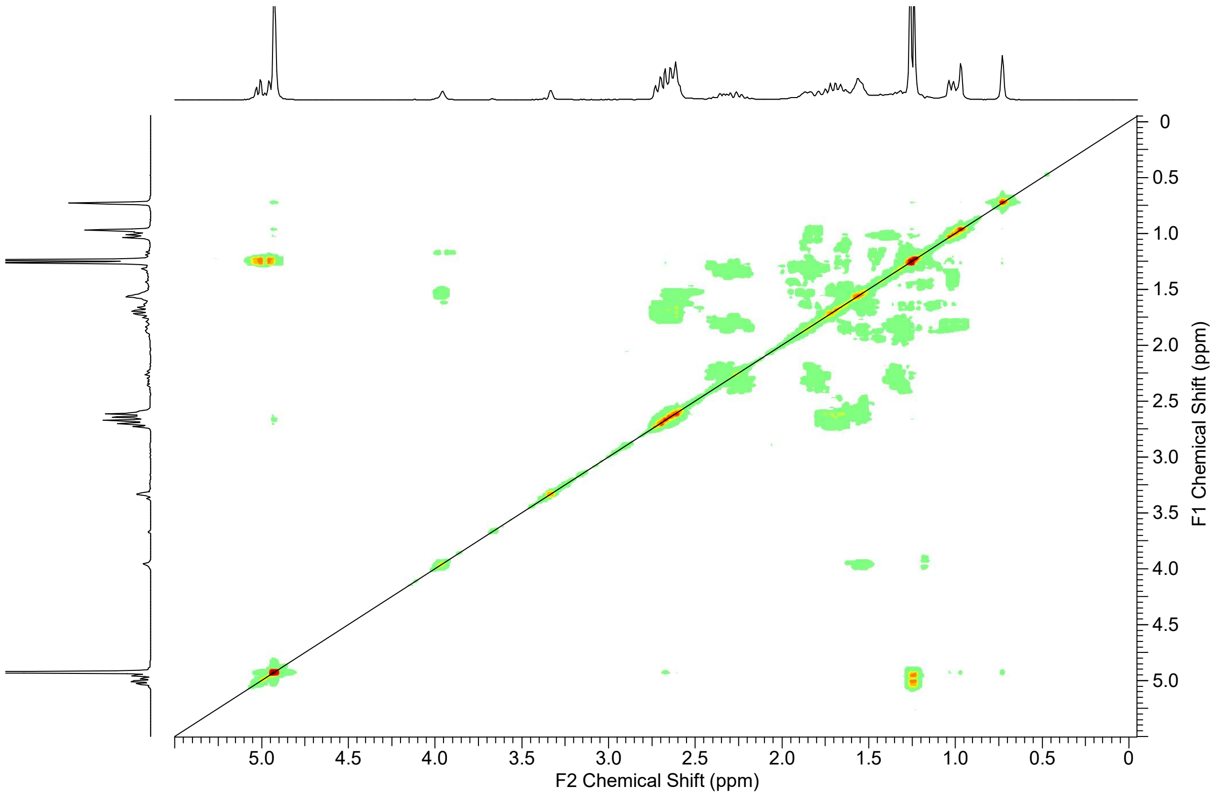


**D.** HMQC spectrum for the claramine **A1** in $CD_3OD$ (0-80 ppm ; 250.13, 62.90 MHz)

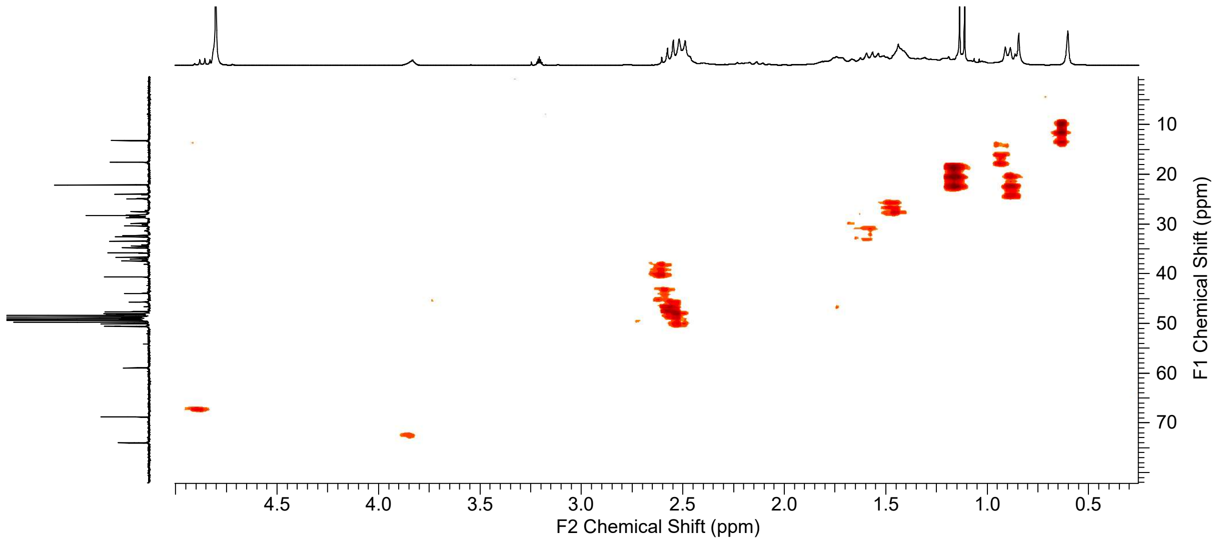


**E.** HMQC spectrum for the claramine **A1** in $CD_3OD$ (0-220 ppm ; 250.13, 62.90 MHz)

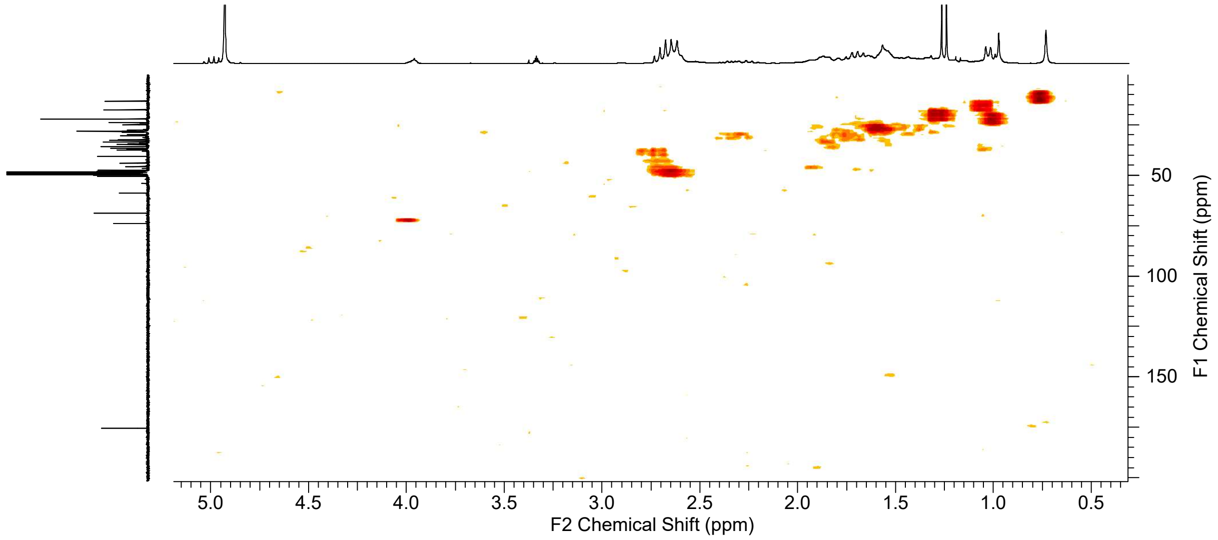


**F.** HMBC spectrum for the claramine **A1** in $CD_3OD$ (250.13, 62.90 MHz)

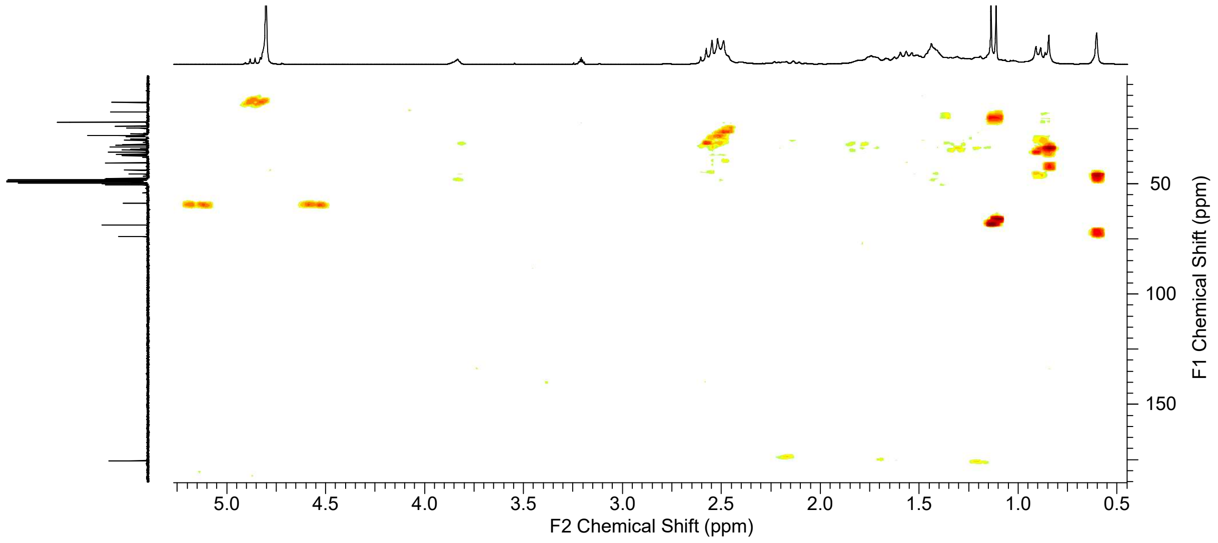

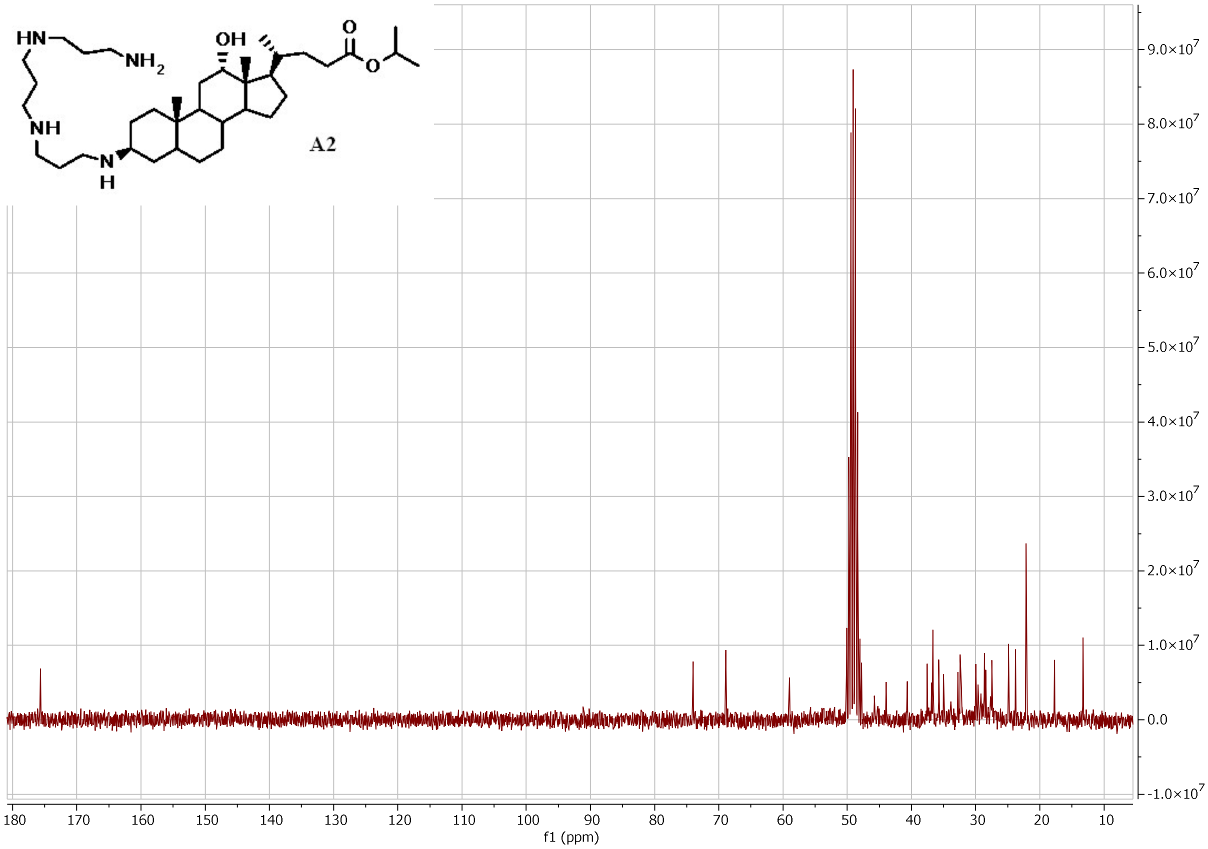
HN
NH2
OH
O
O
NH
N
H
A2
f1 (ppm)

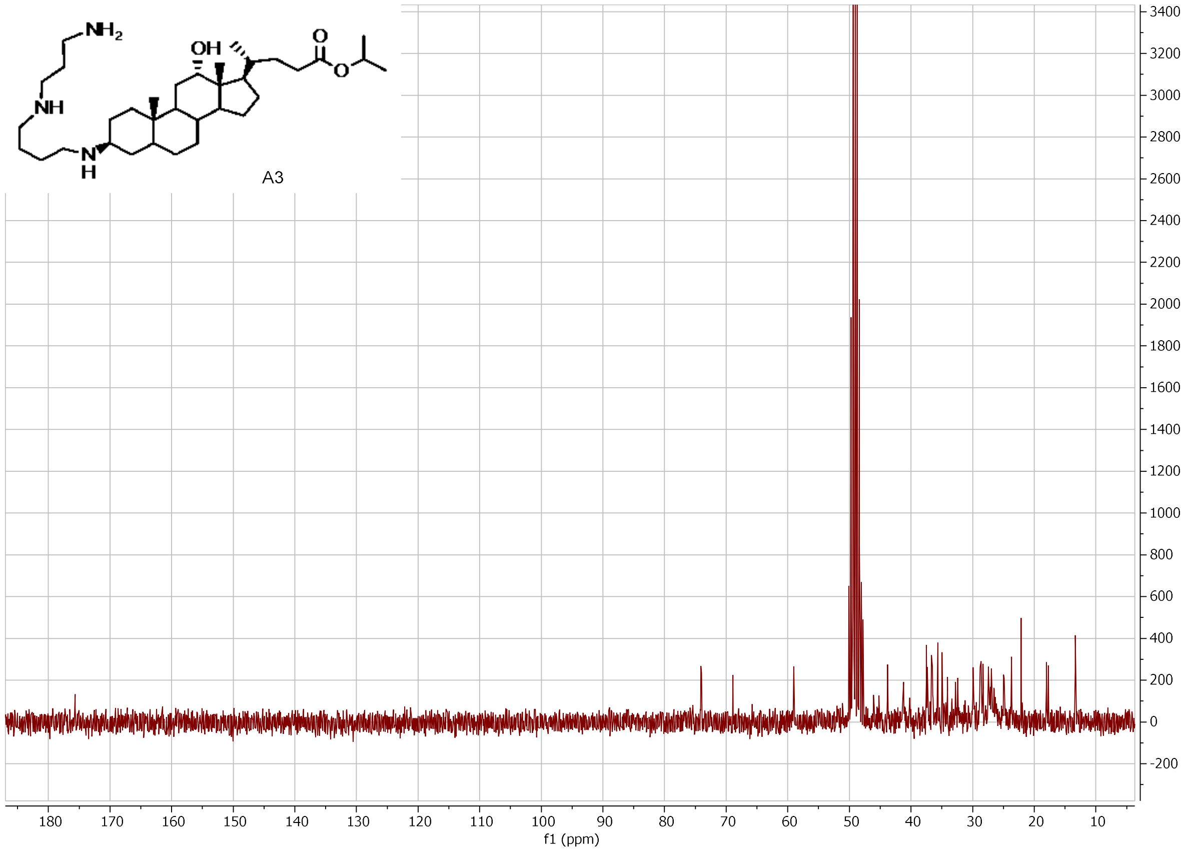
NH2
OH
O
O
NH
N
H
A3
f1 (ppm)

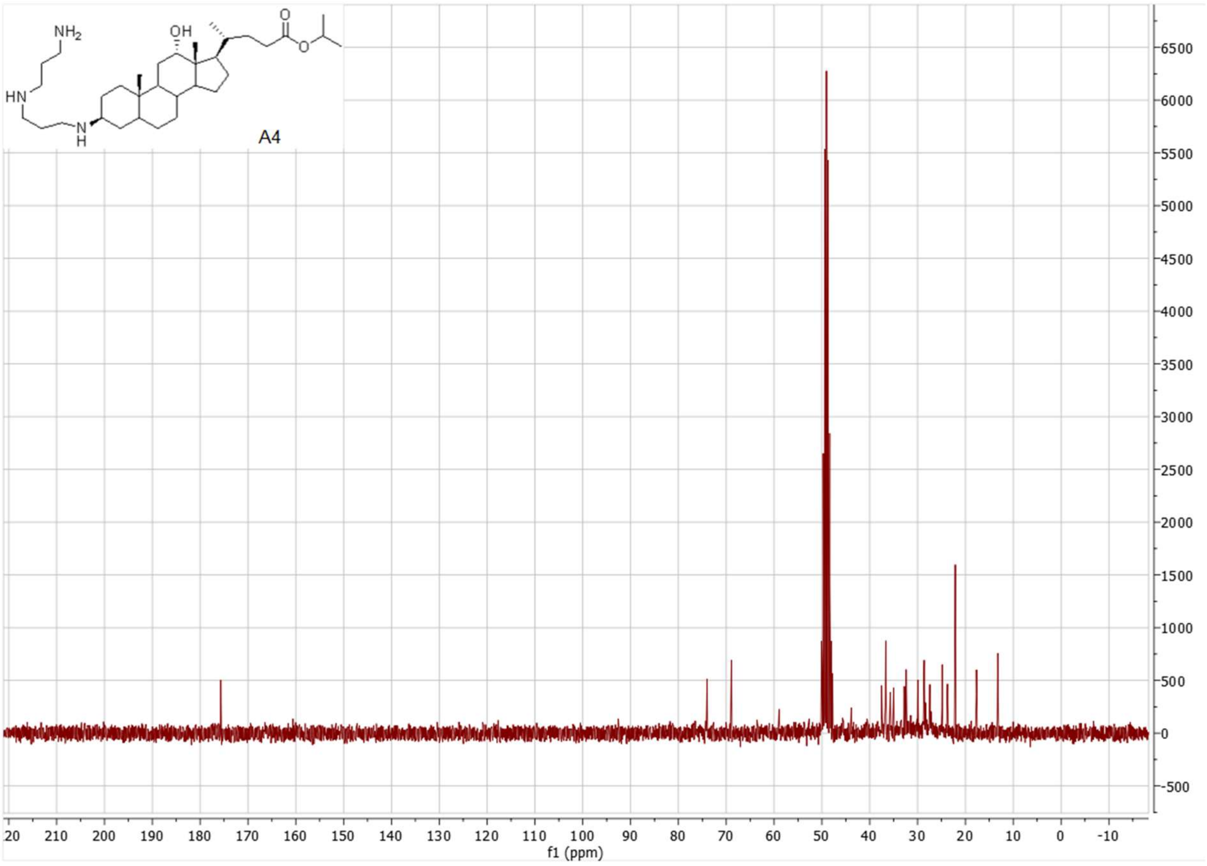
NH2
OH
O
HN
N
H
A4
f1 (ppm)

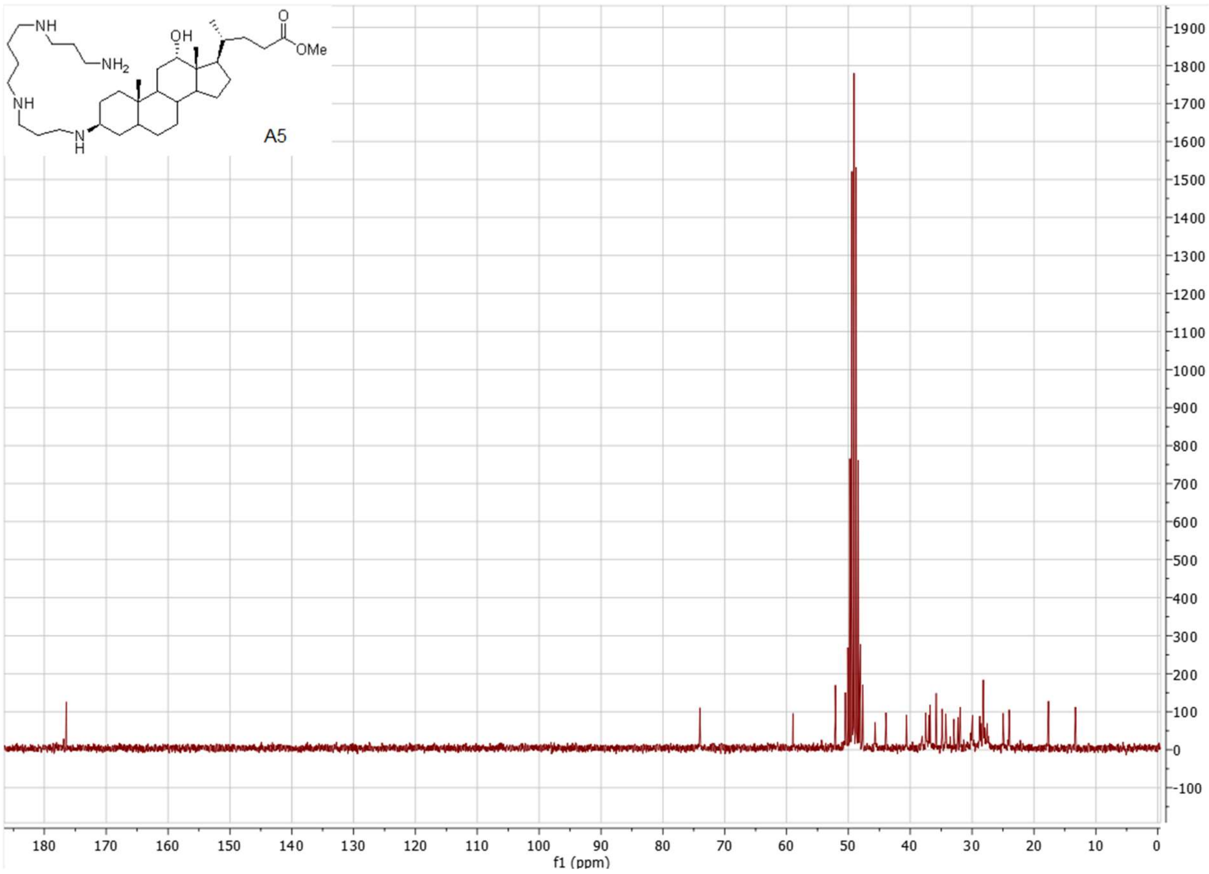
NH
OH
O
NH2
OMe
NH
N
H
A5
f1 (ppm)

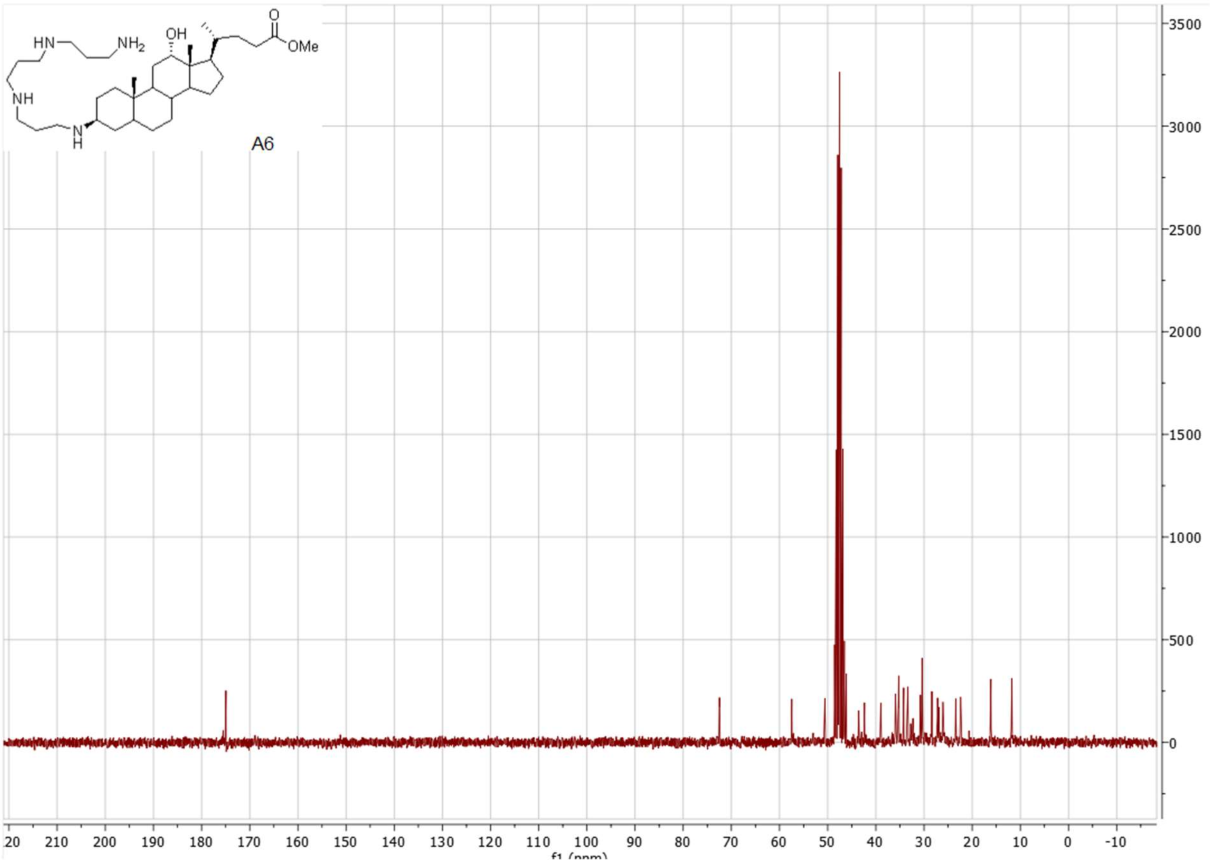
HN
NH2
OH
O
OMe
NH
N
H
A6
f1 (ppm)

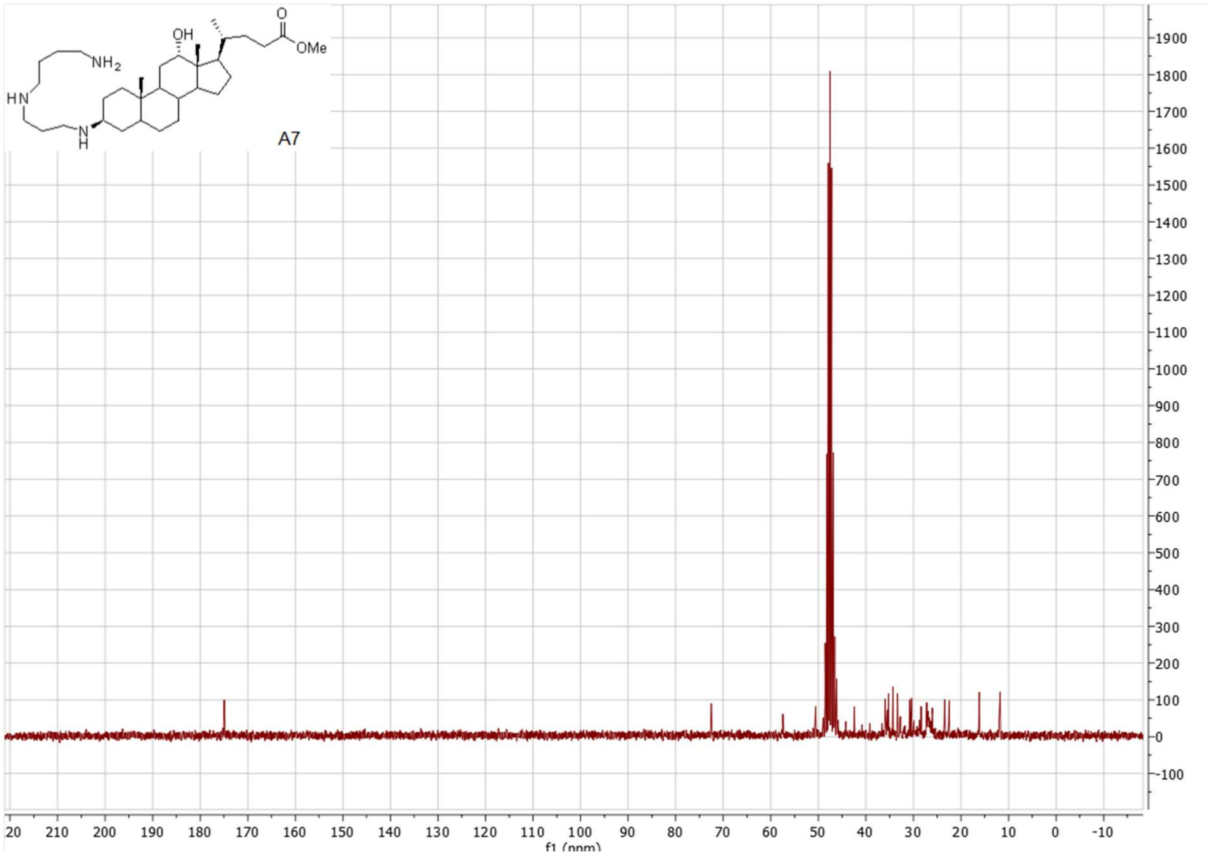
OH
O
OMe
NH2
HN
N
H
A7
f1 (ppm)

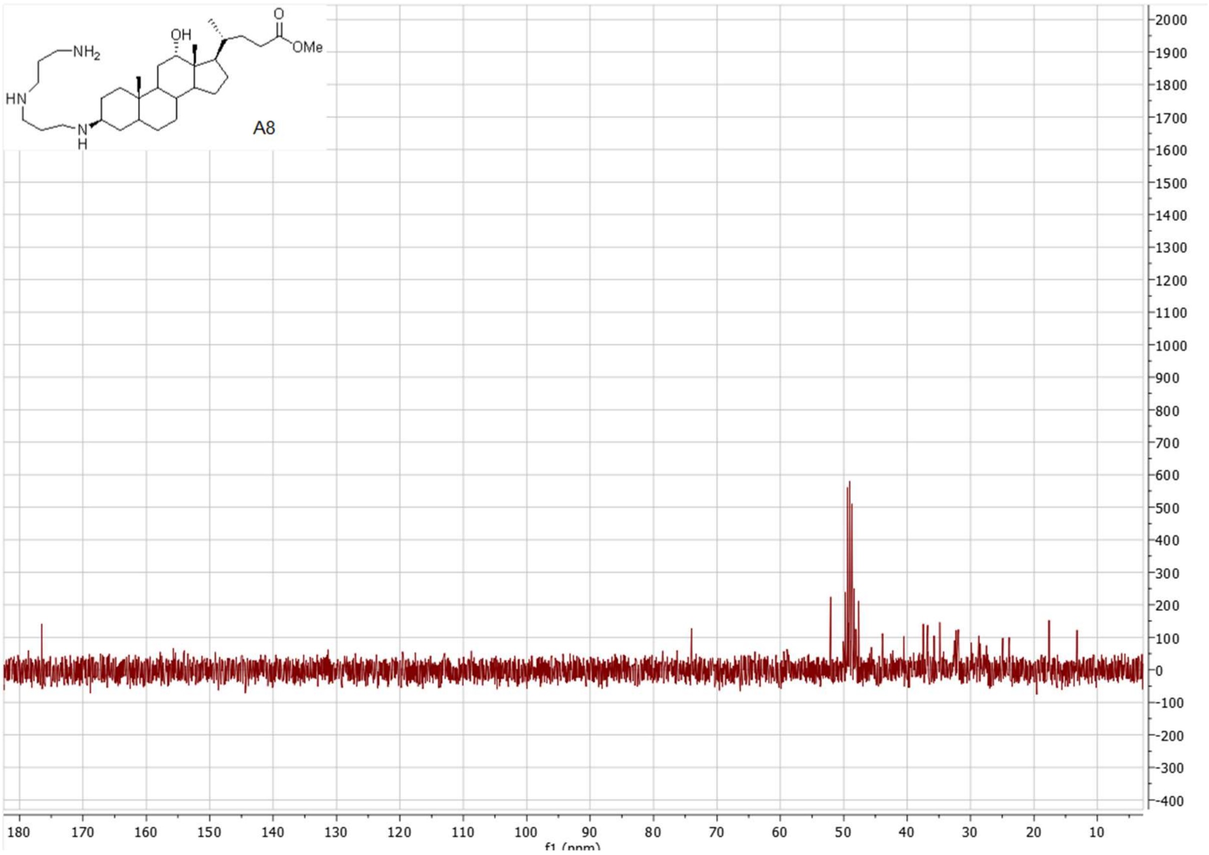
A8
OH
OMe
NH2
HN
N
H
f1 (ppm)

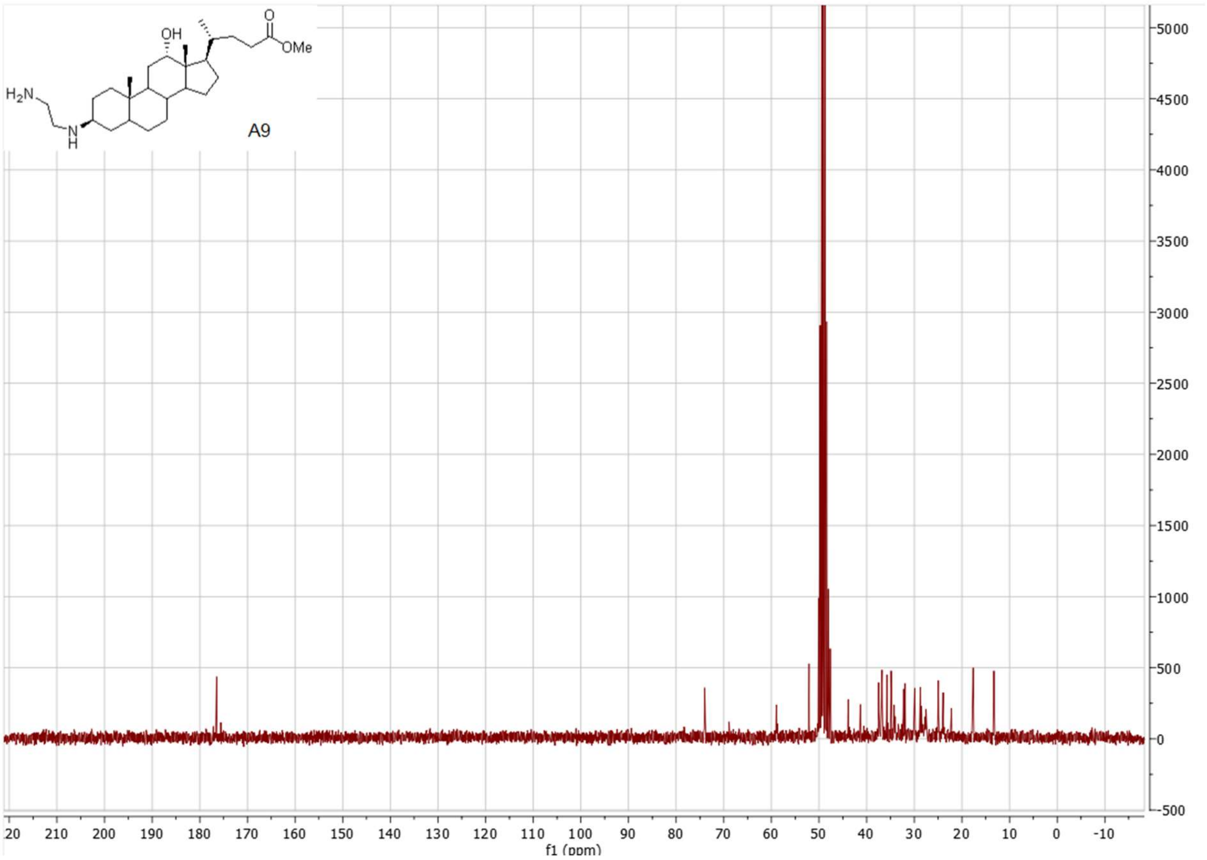
A9
OH
OMe
H2N
N
H
f1 (ppm)

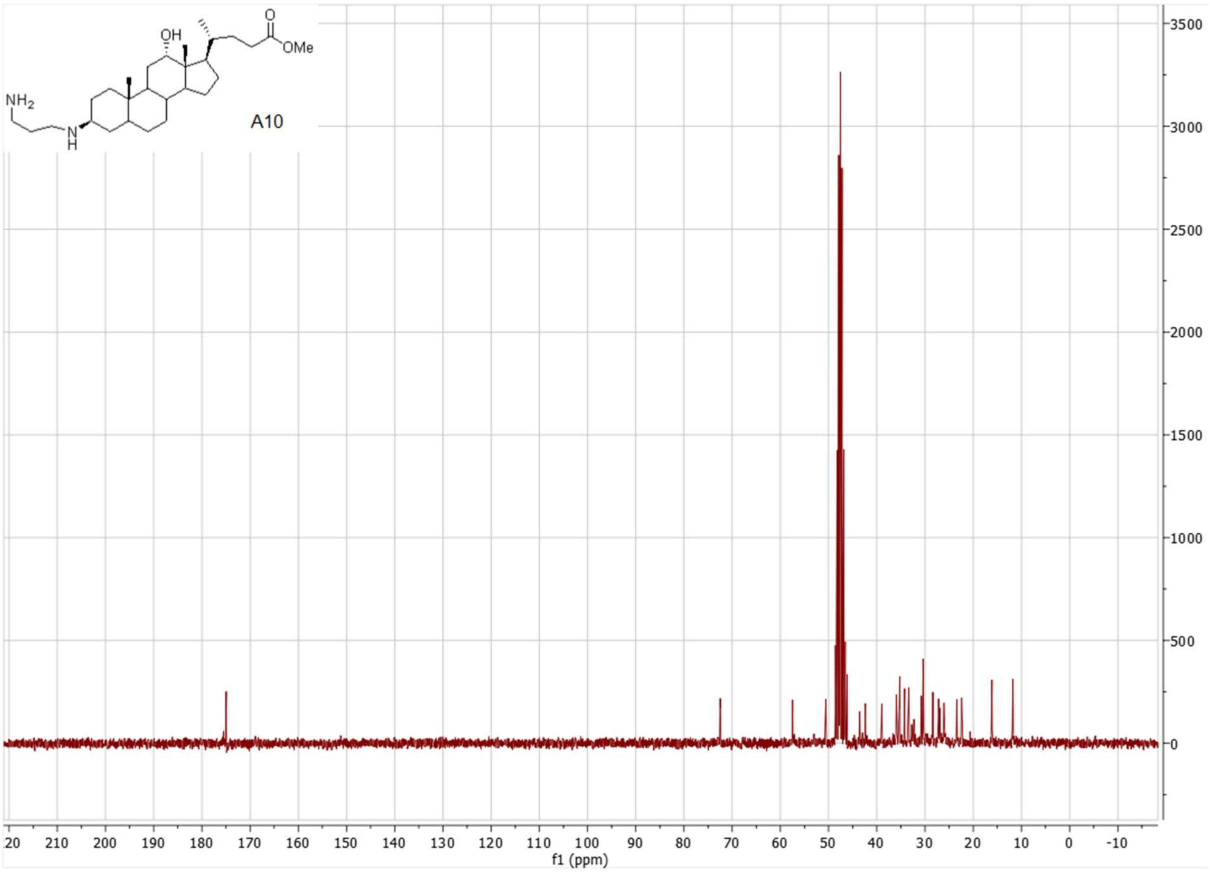
OH
O
OMe
NH2
N
H
A10
f1 (ppm)

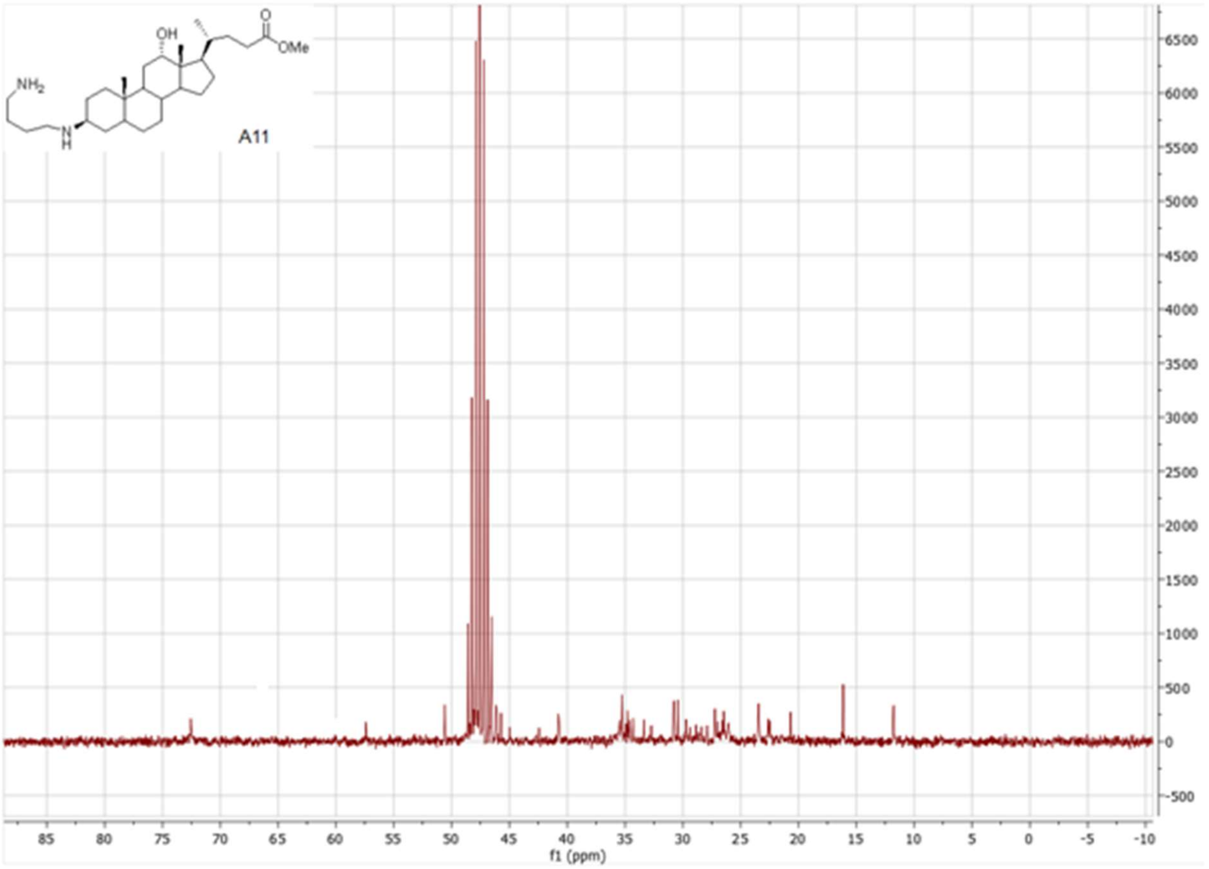
OH
O
OMe
NH2
N
H
A11
f1 (ppm)

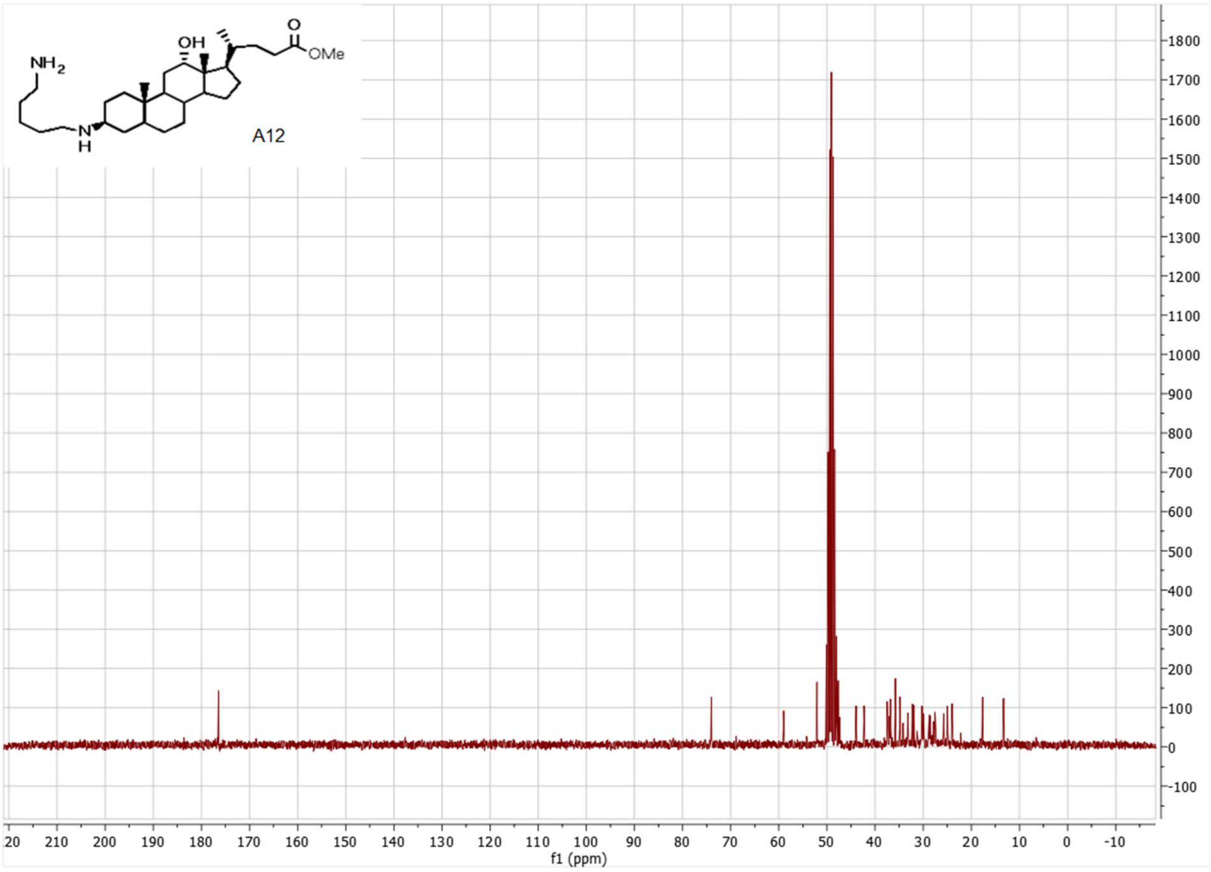

A12
NH2
OH
OMe
H
N
f1 (ppm)


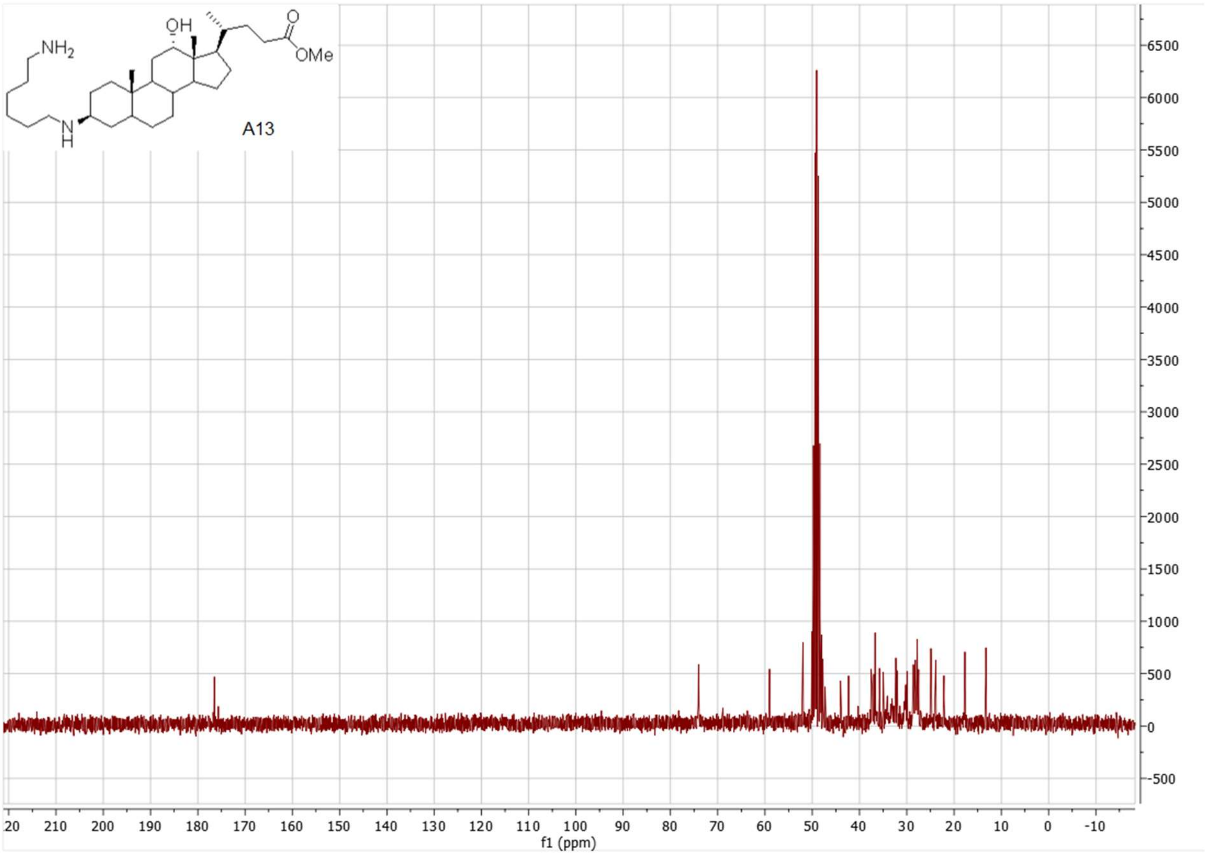

A13
NH2
OH
OMe
N
H
f1 (ppm)

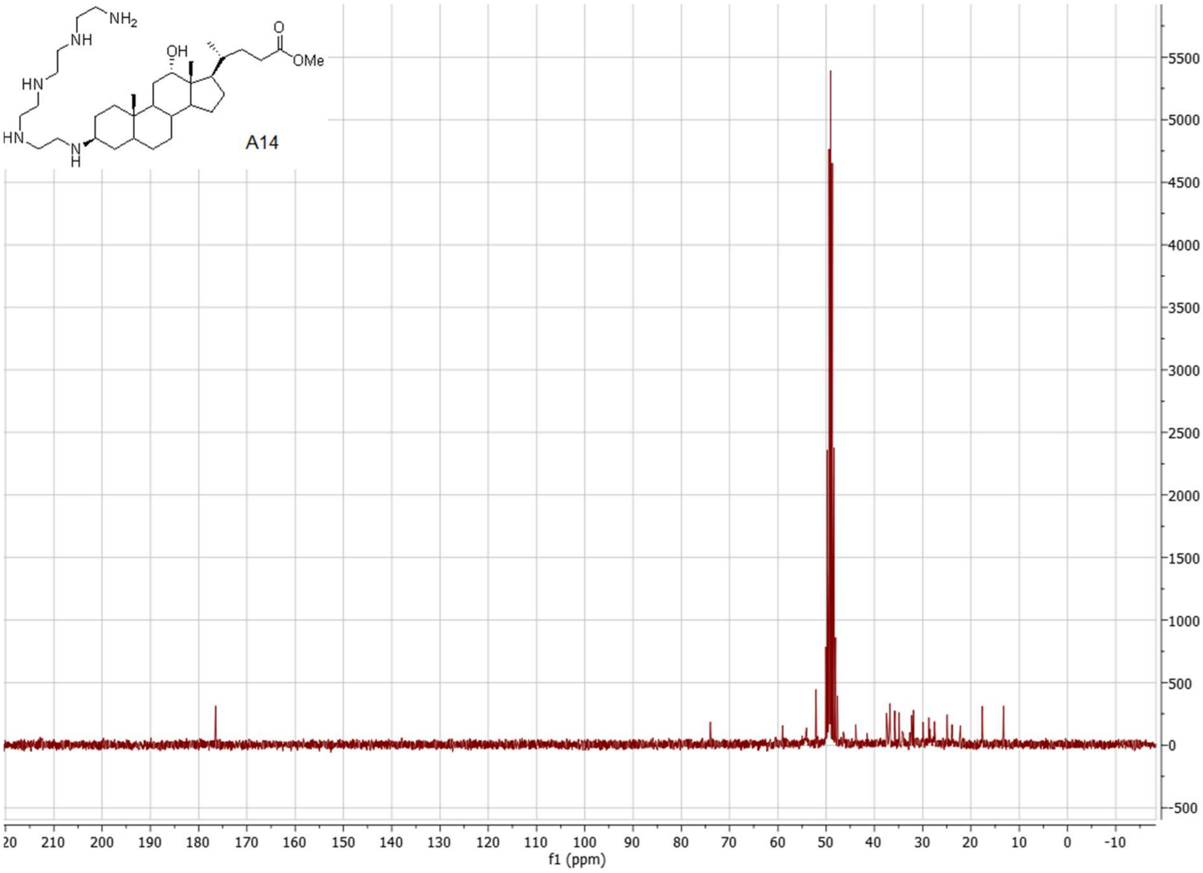
A14
OH
OMe
NH2
NH
HN
HN
N H
f1 (ppm)

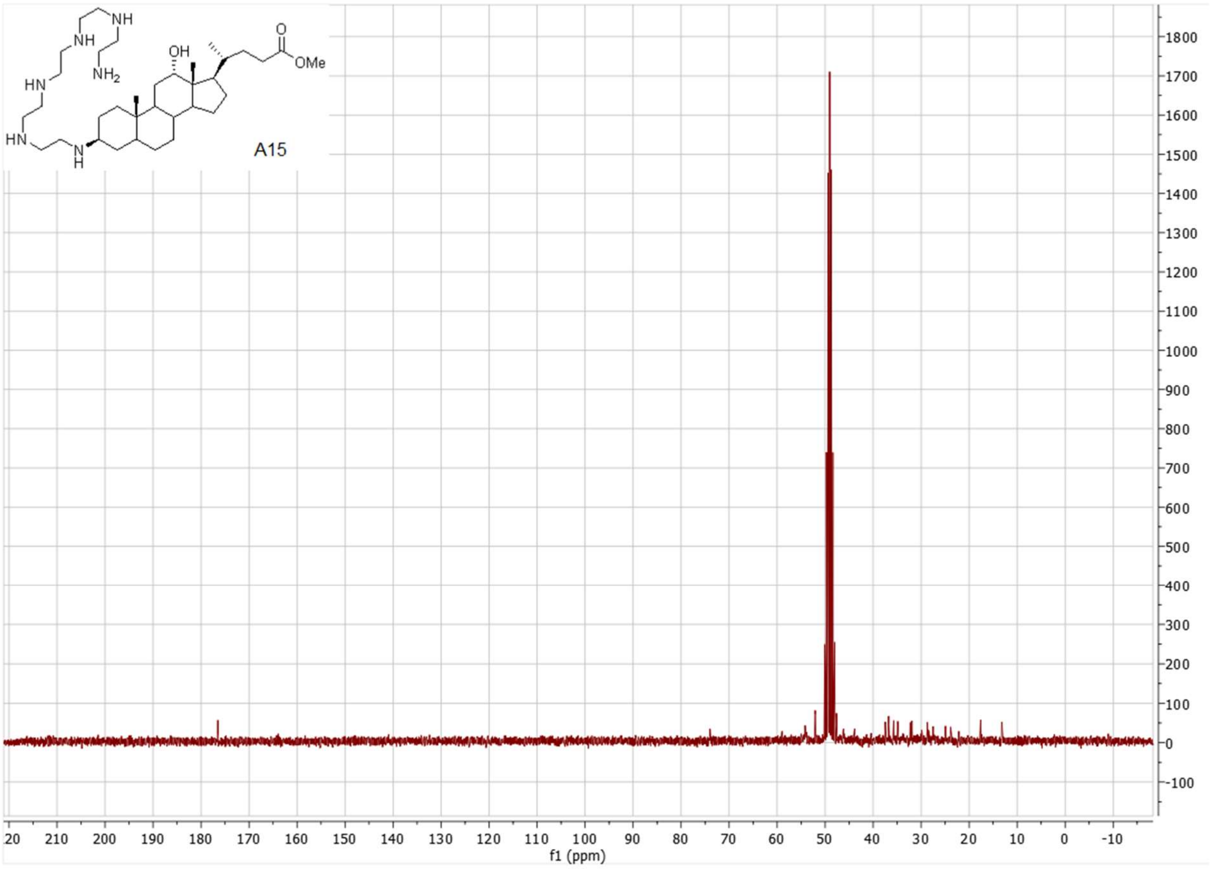
A15
OH
OMe
NH
NH2
HN
HN
N H
f1 (ppm)

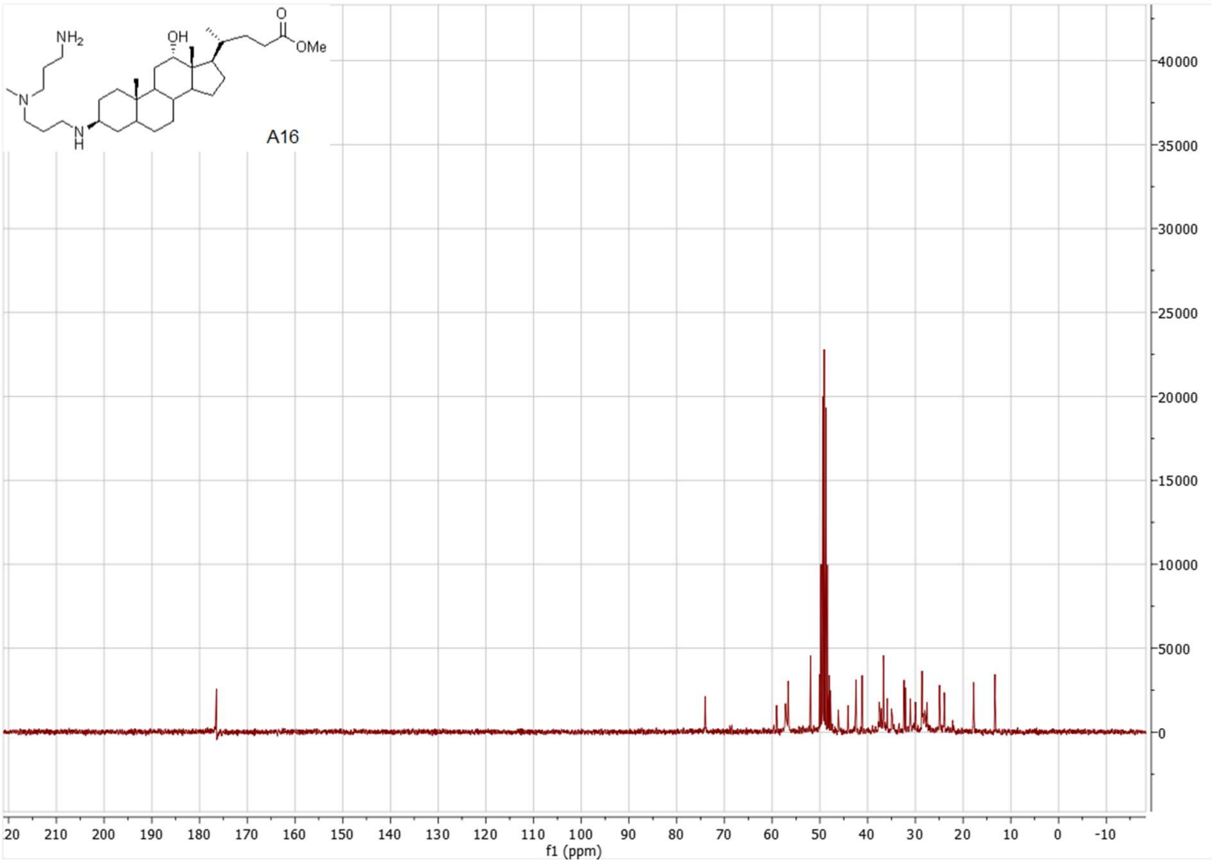
NH2
OH
O
OMe
N
N
H
A16
f1 (ppm)

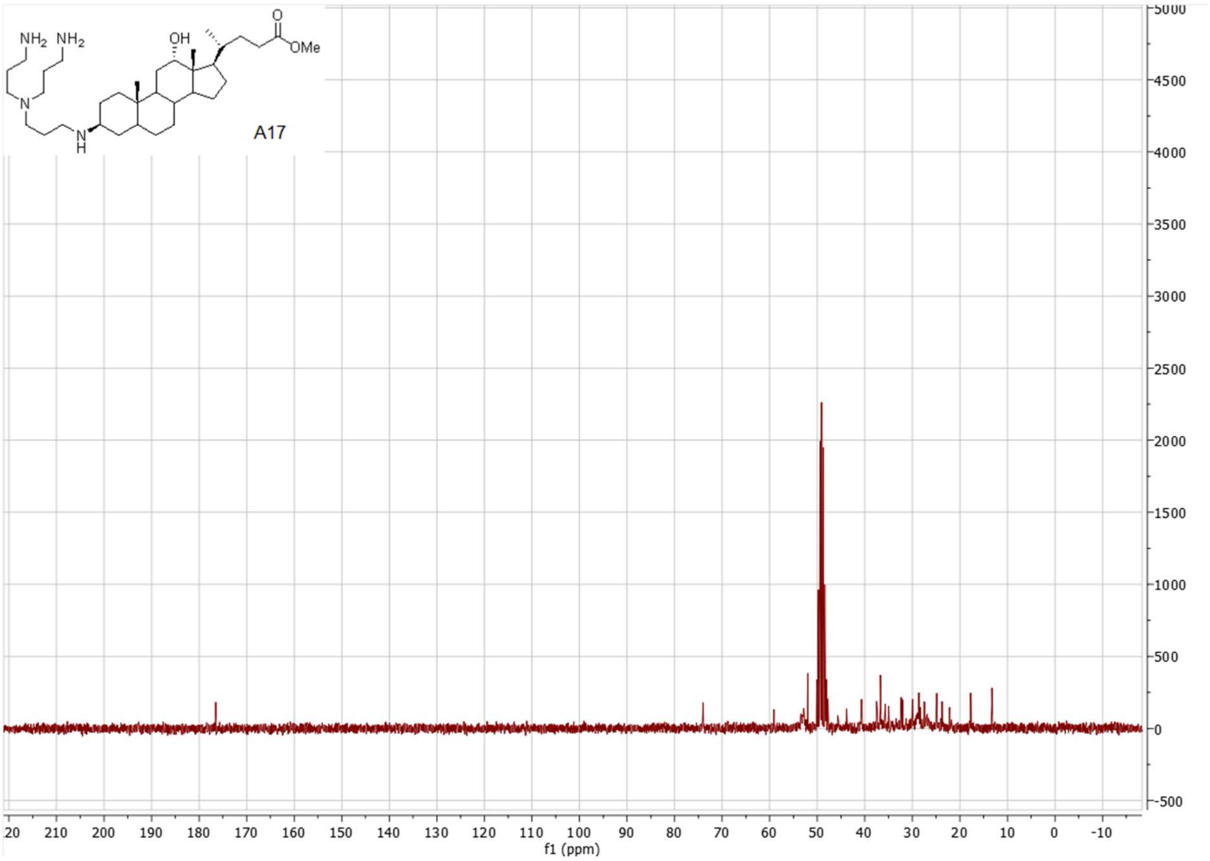
NH2 NH2
OH
O
OMe
N
N
H
A17
f1 (ppm)

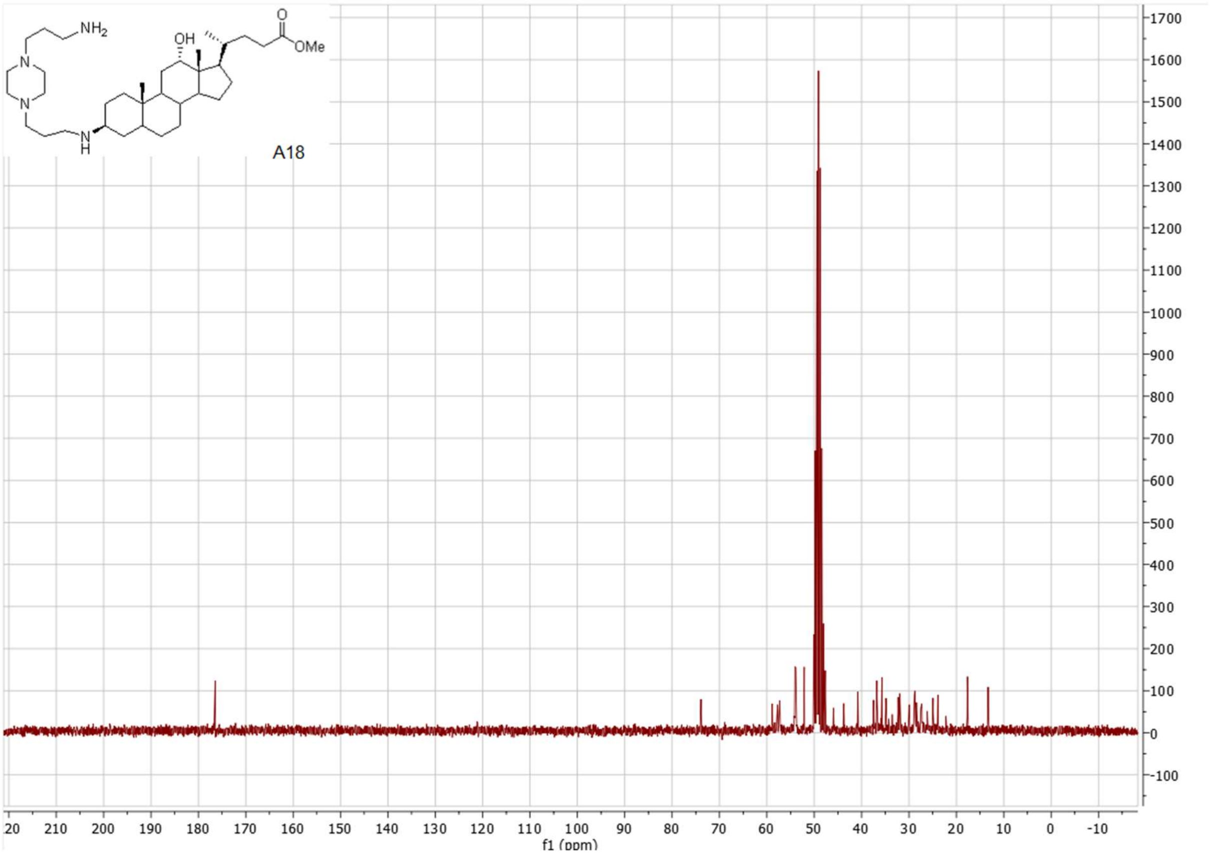
NH2
OH
OMe
A18
f1 (ppm)

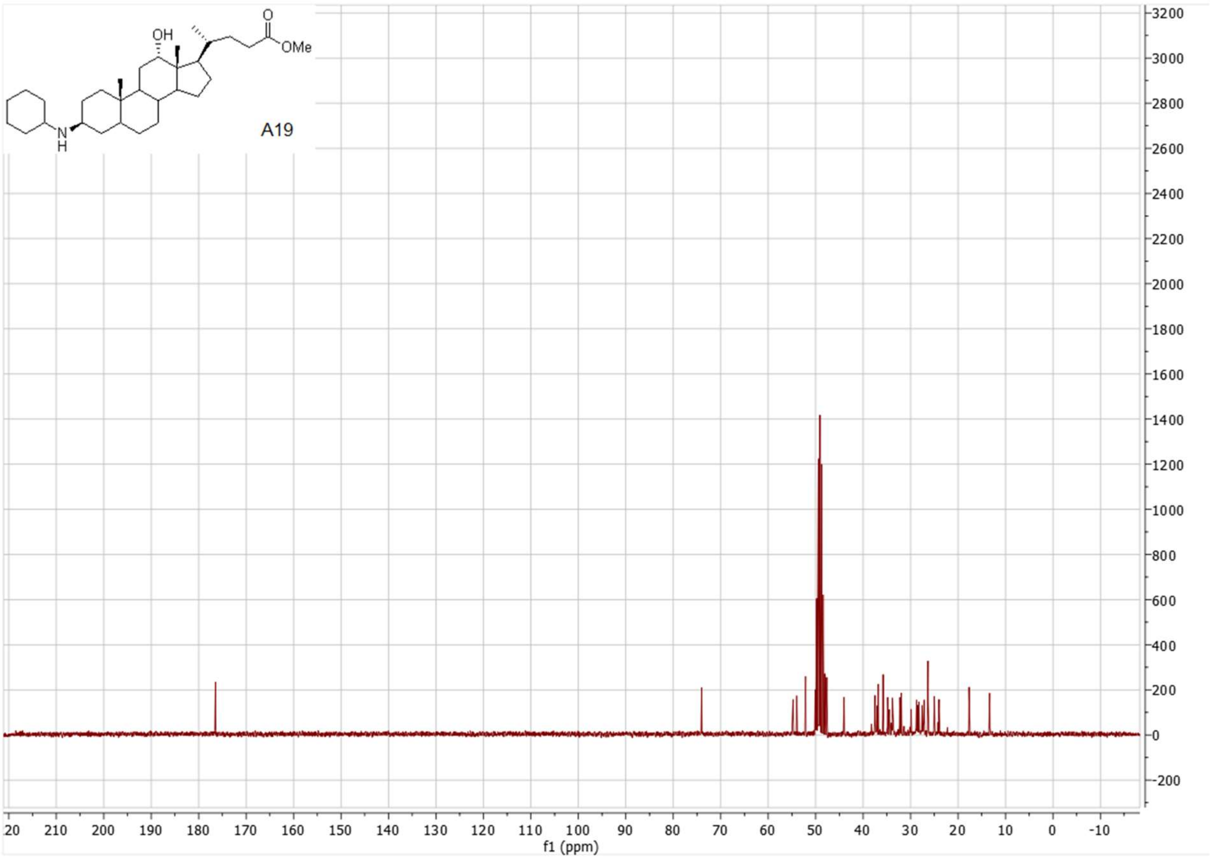
OH
OMe
A19
f1 (ppm)

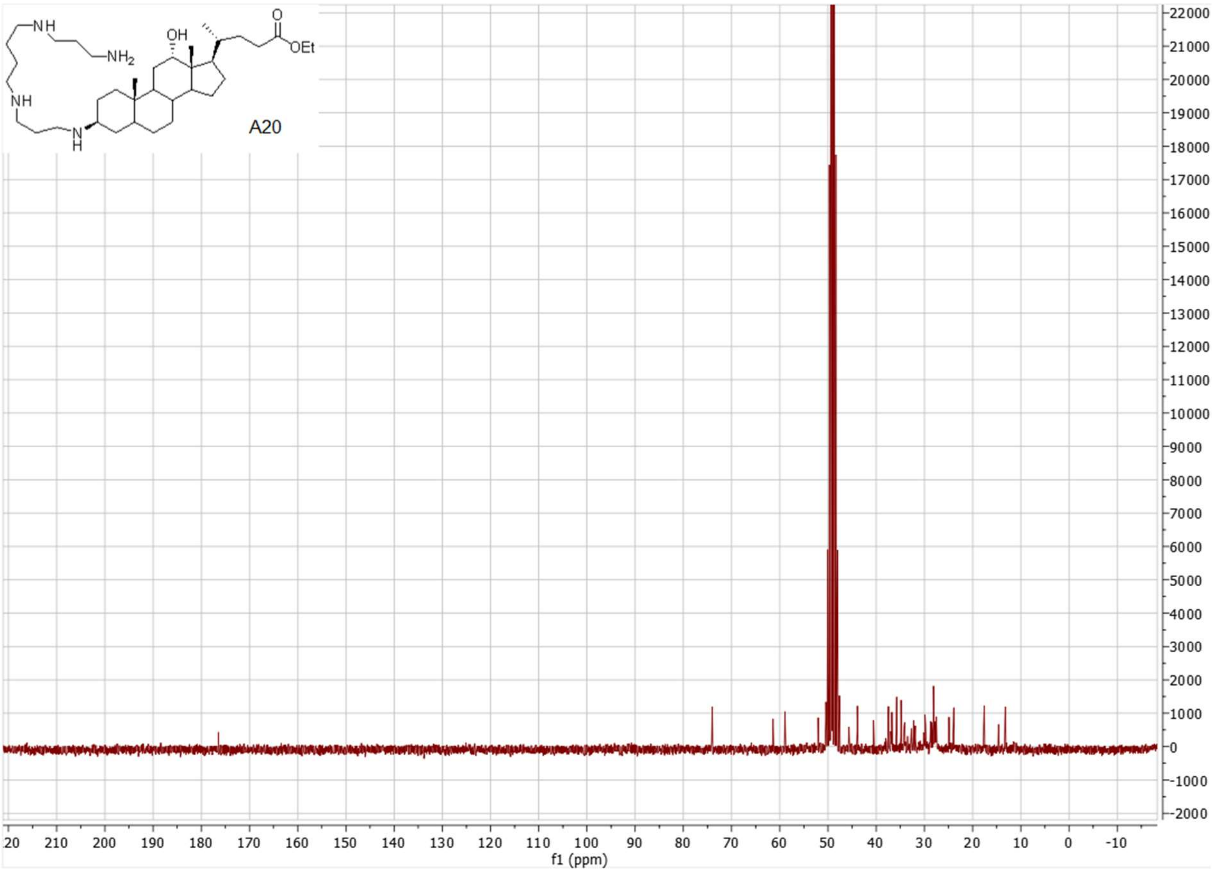
A20
OEt
OH
NH
NH2
H
f1 (ppm)

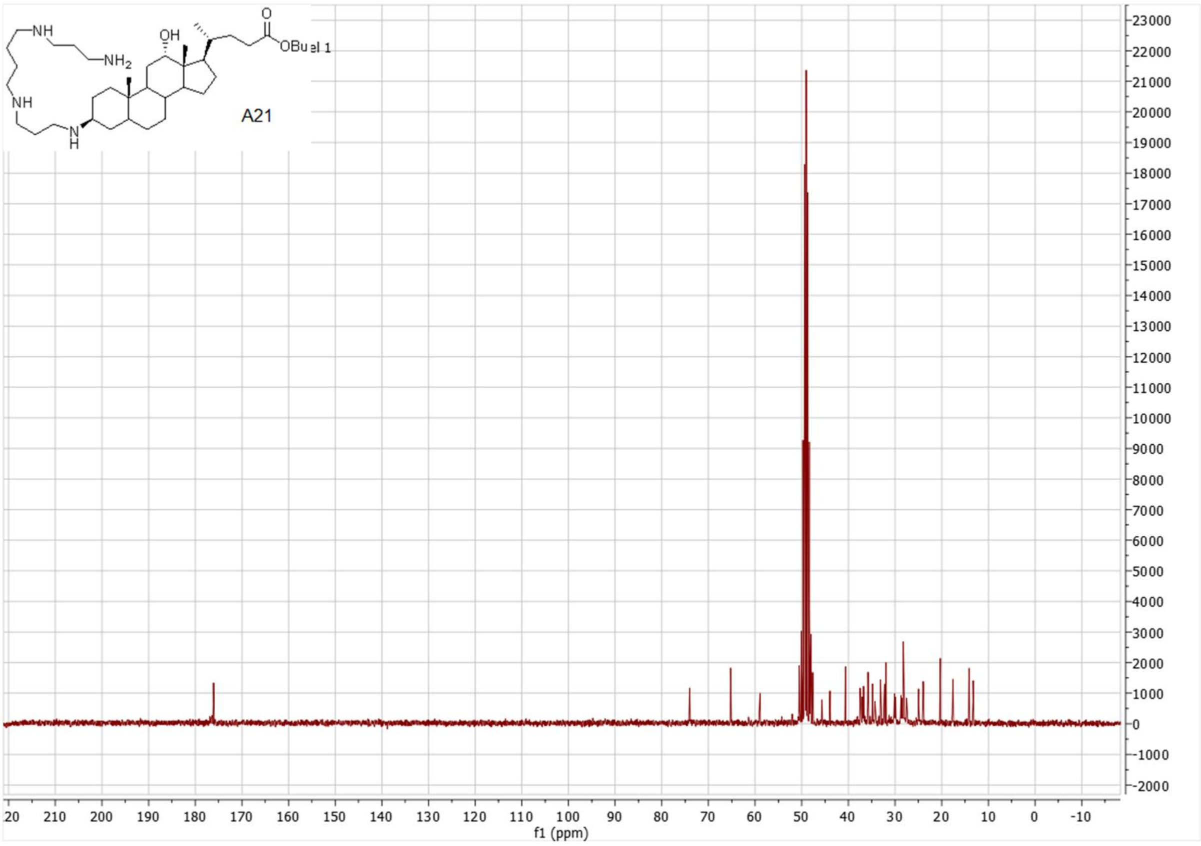
A21
OBu
OH
NH
NH2
H
f1 (ppm)

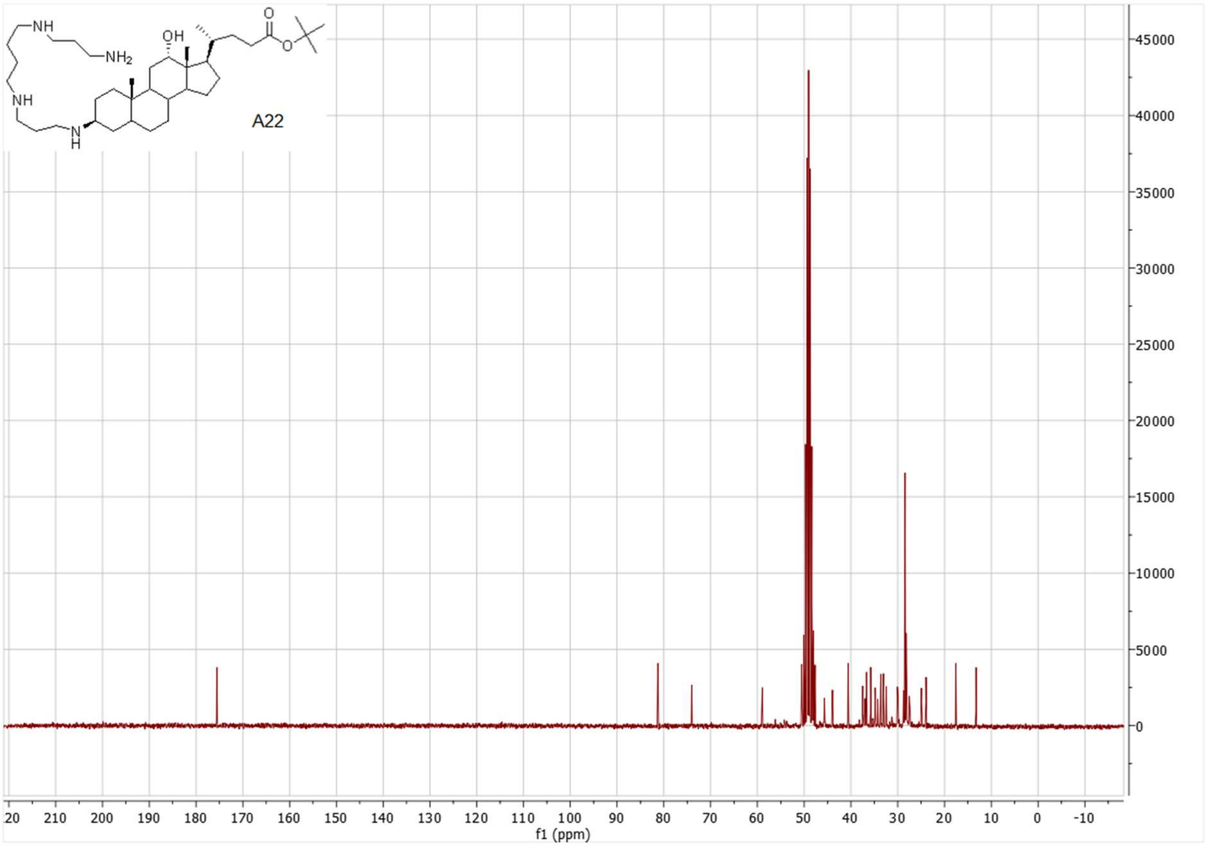
NH
NH2
OH
O
O
NH
N
H
A22
45000
40000
35000
30000
25000
20000
15000
10000
5000
0
220 210 200 190 180 170 160 150 140 130 120 110 100 90 80 70 60 50 40 30 20 10 0 -10
f1 (ppm)

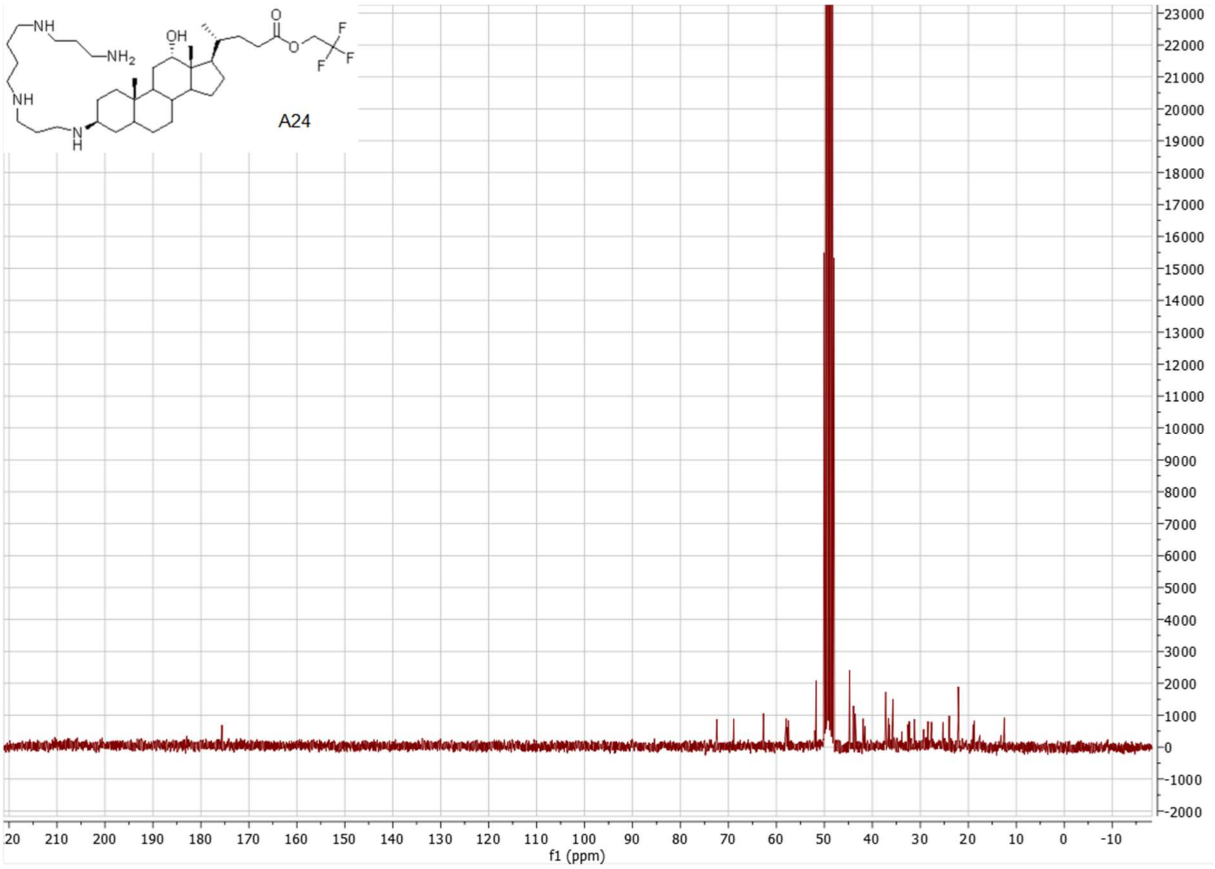
NH
NH2
OH
O
O
F
F
F
NH
N
H
A24
23000
22000
21000
20000
19000
18000
17000
16000
15000
14000
13000
12000
11000
10000
9000
8000
7000
6000
5000
4000
3000
2000
1000
0
-1000
-2000
220 210 200 190 180 170 160 150 140 130 120 110 100 90 80 70 60 50 40 30 20 10 0 -10
f1 (ppm)

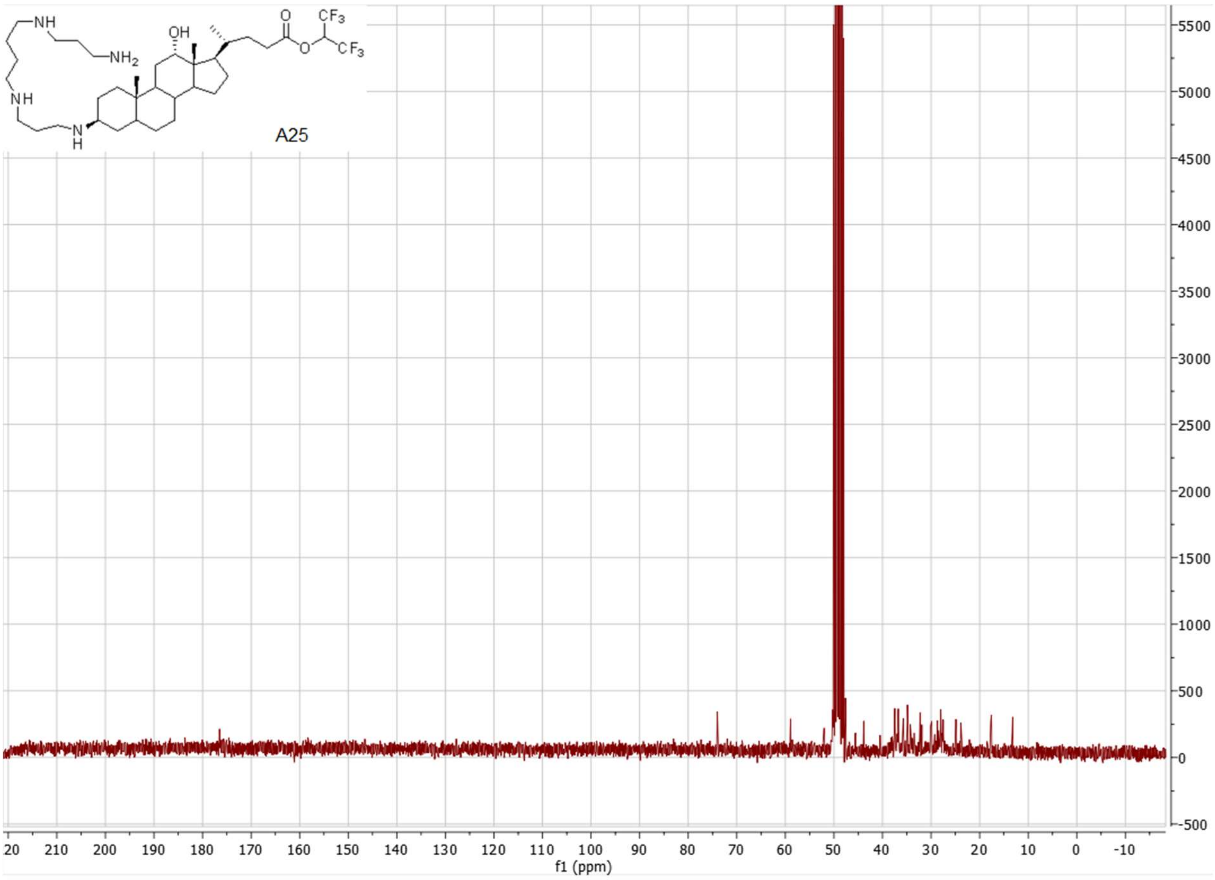
A25
NH
NH2
NH
N
H
OH
O
O
CF3
CF3
f1 (ppm)
20 210 200 190 180 170 160 150 140 130 120 110 100 90 80 70 60 50 40 30 20 10 0 -10
5500 5000 4500 4000 3500 3000 2500 2000 1500 1000 500 0 -500

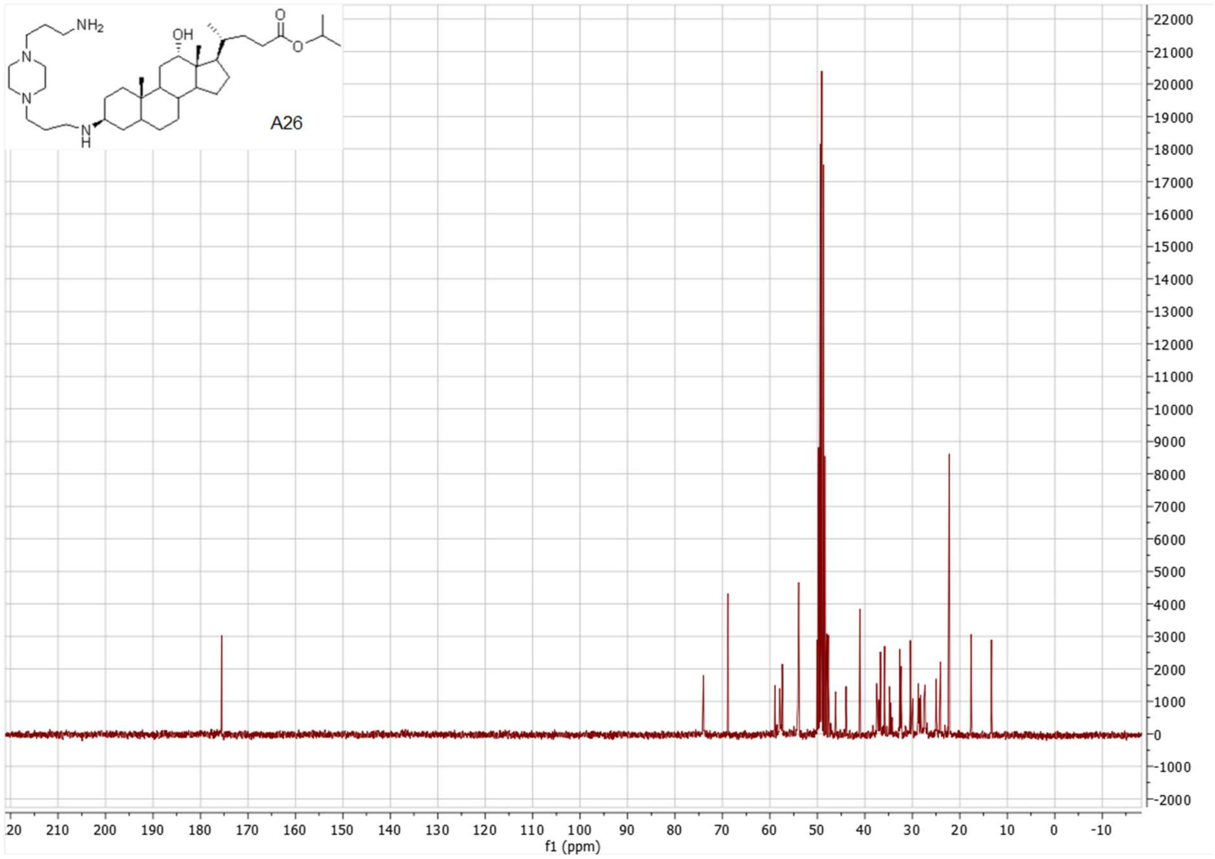
A26
NH2
N
N
N
H
OH
O
O
f1 (ppm)
20 210 200 190 180 170 160 150 140 130 120 110 100 90 80 70 60 50 40 30 20 10 0 -10
22000 21000 20000 19000 18000 17000 16000 15000 14000 13000 12000 11000 10000 9000 8000 7000 6000 5000 4000 3000 2000 1000 0 -1000 -2000

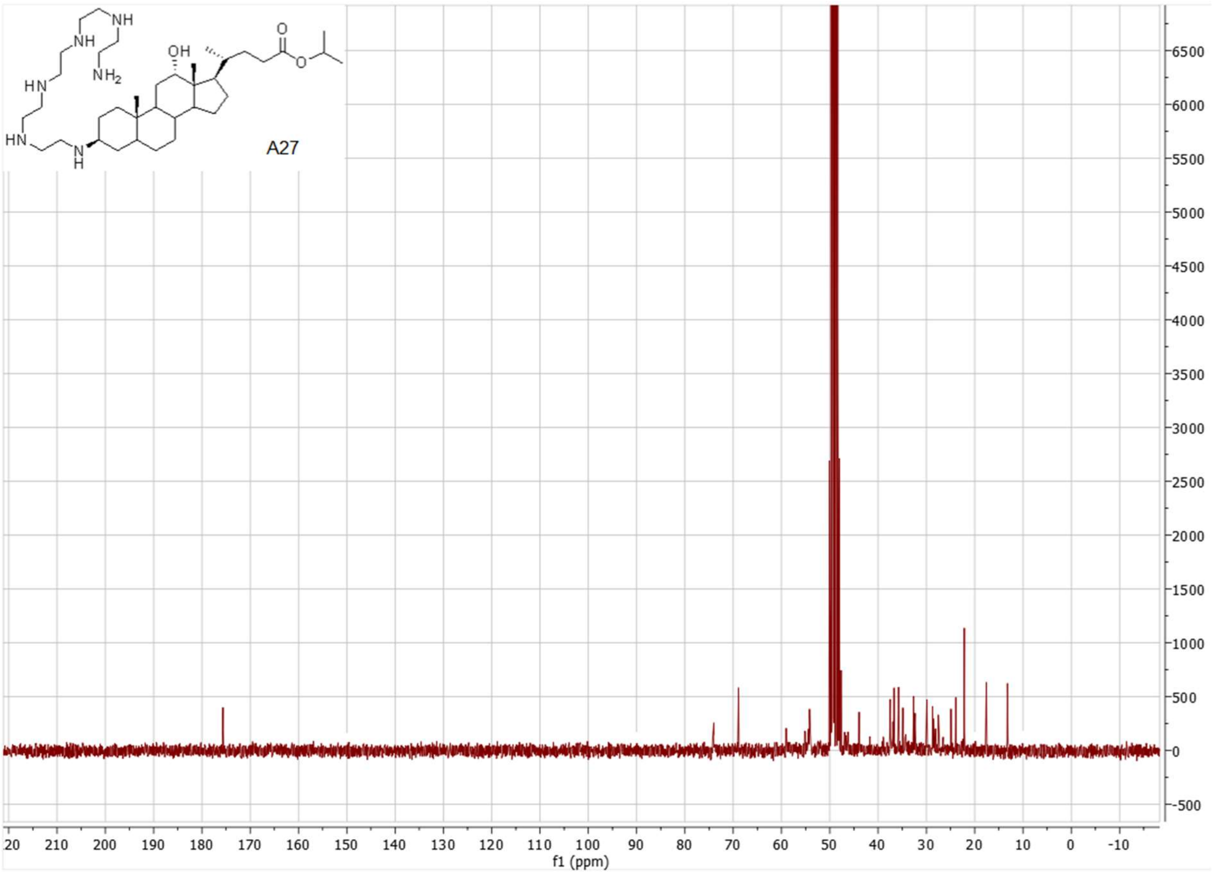
A27
f1 (ppm)

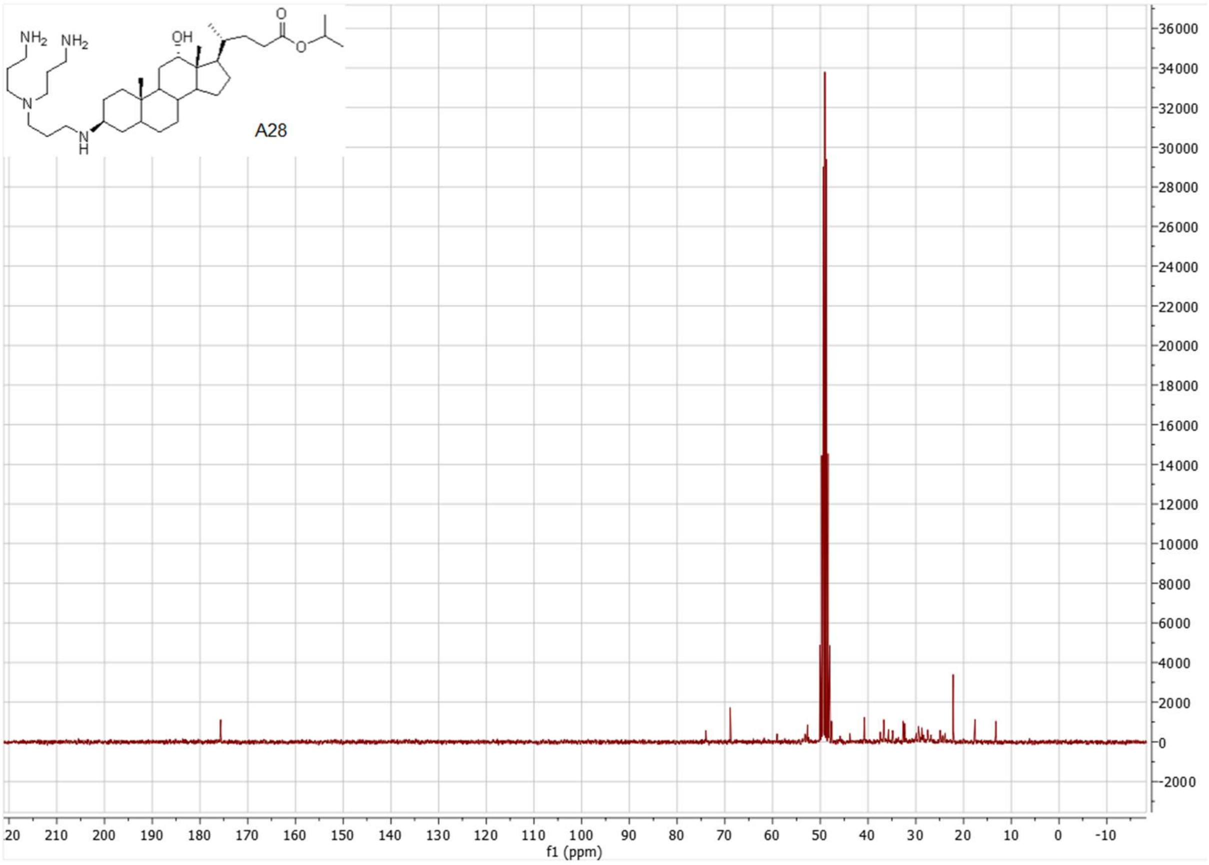
A28
f1 (ppm)

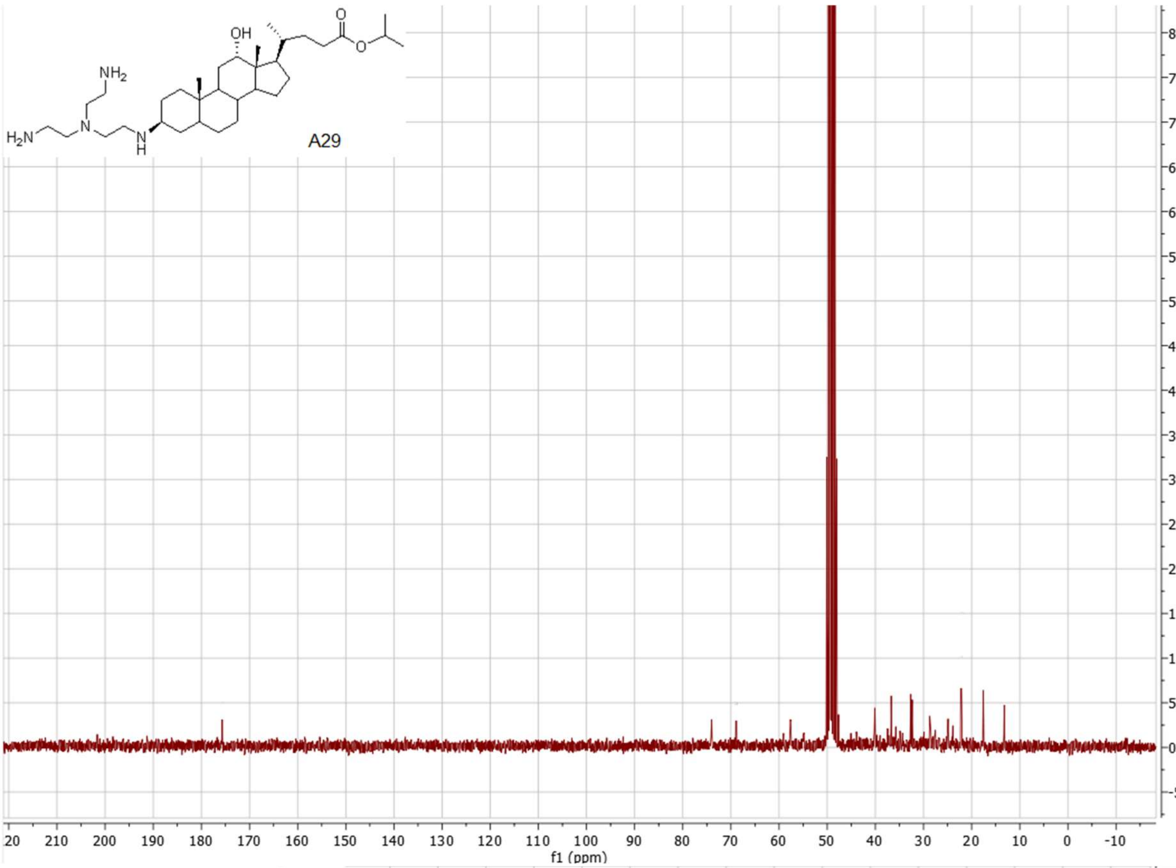

OH
NH2
H2N
N
H
A29
f1 (ppm)


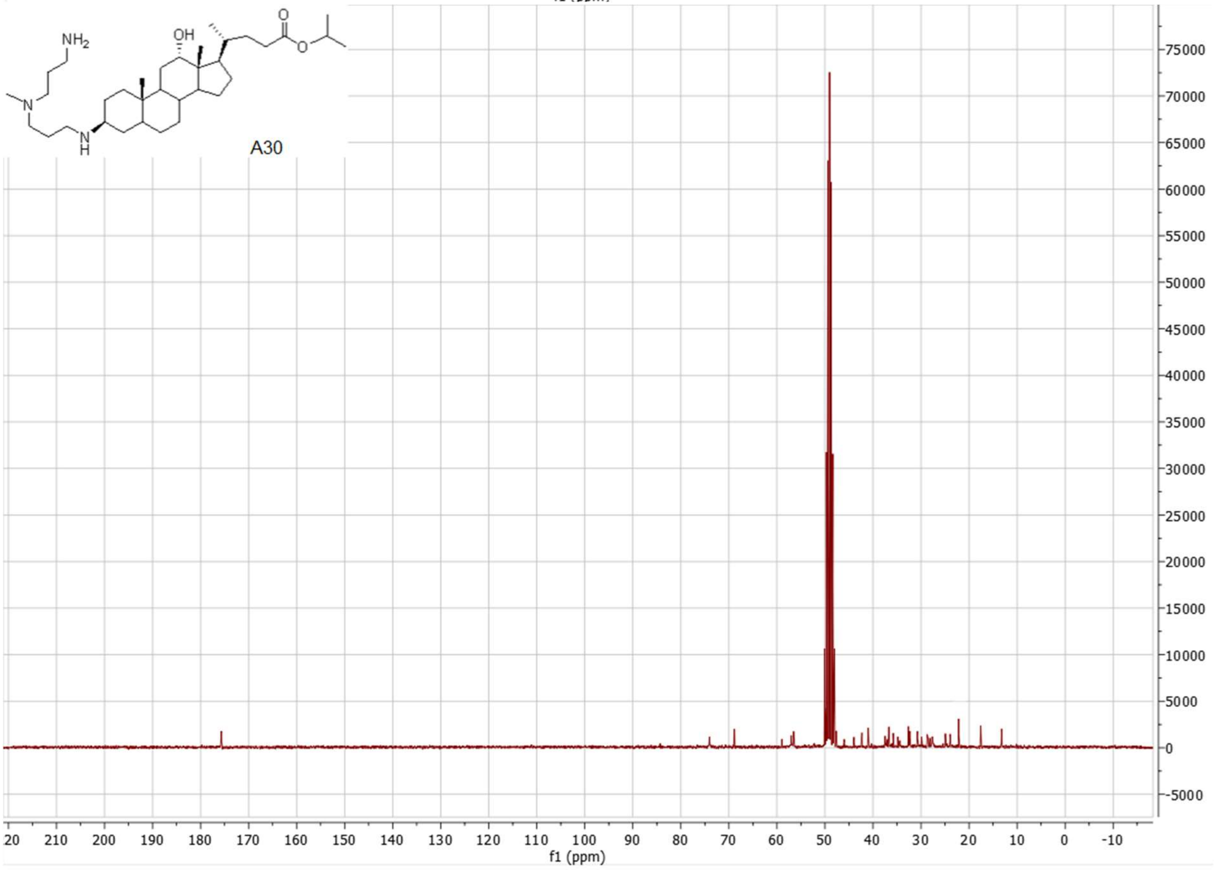

OH
NH2
N
H
A30
f1 (ppm)

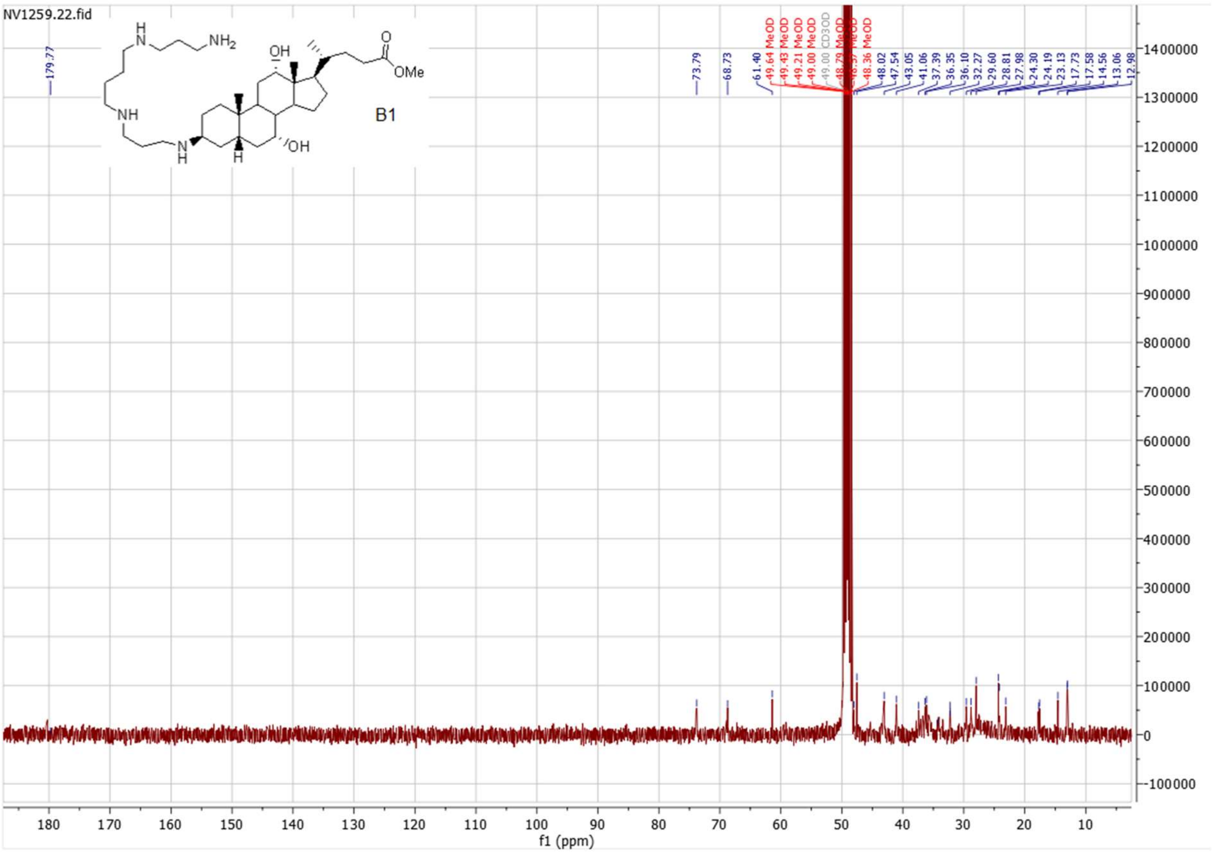
NV1259.22.fid
B1
179.77
73.79
68.73
61.40
49.64 MeOD
49.43 MeOD
49.21 MeOD
49.00 MeOD
49.00 CD3OD
48.79 MeOD
48.57 MeOD
48.36 MeOD
48.02
47.54
43.05
41.06
37.39
36.35
36.10
32.27
29.60
28.81
27.98
24.30
24.19
23.13
17.73
17.58
14.56
13.06
12.98
f1 (ppm)

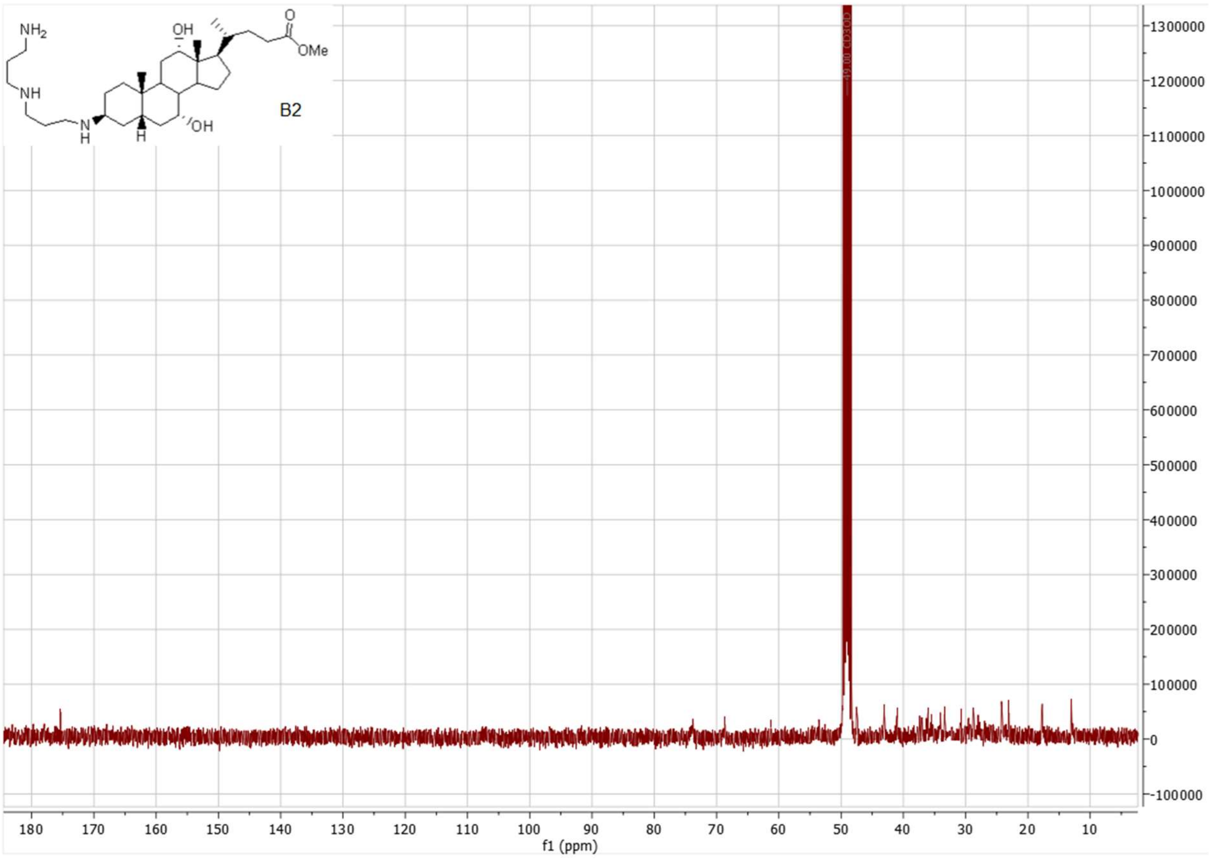
B2
49.00 CD3OD
f1 (ppm)

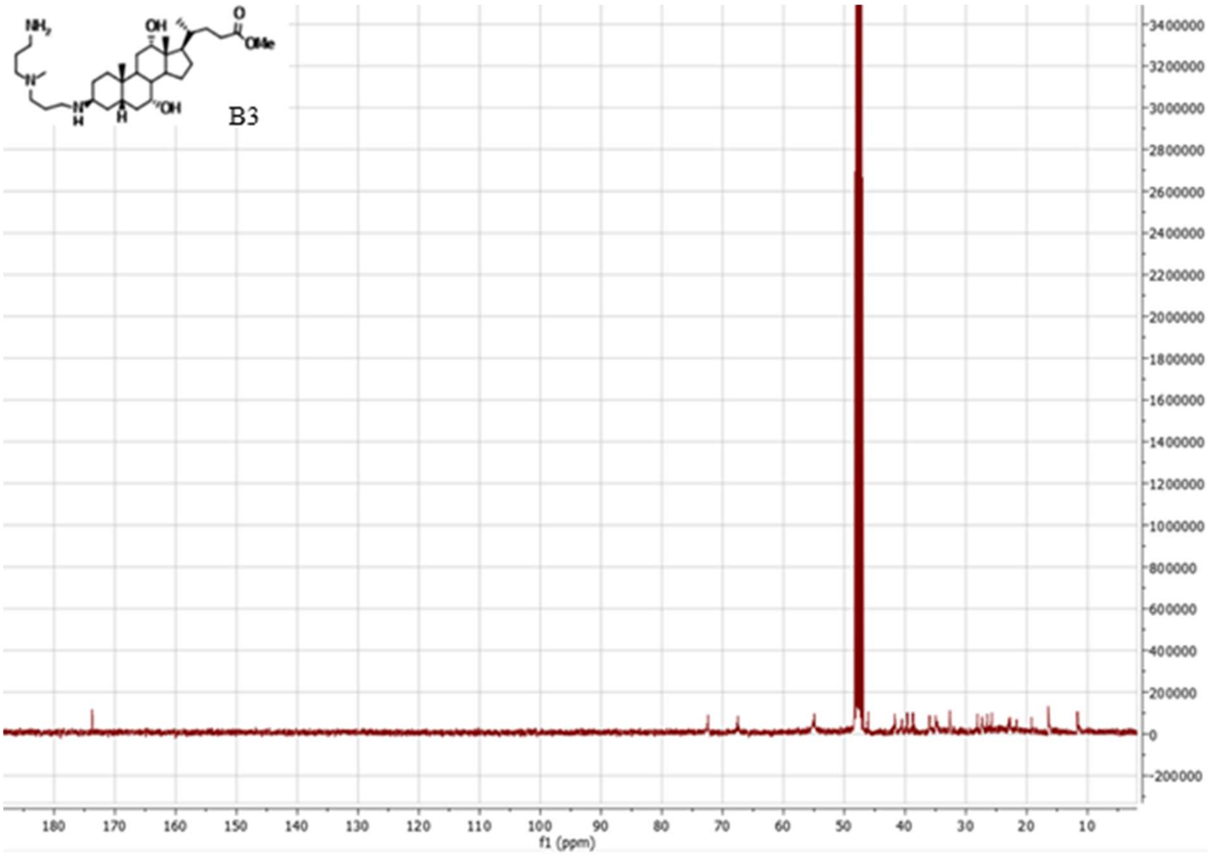
B3
f1 (ppm)

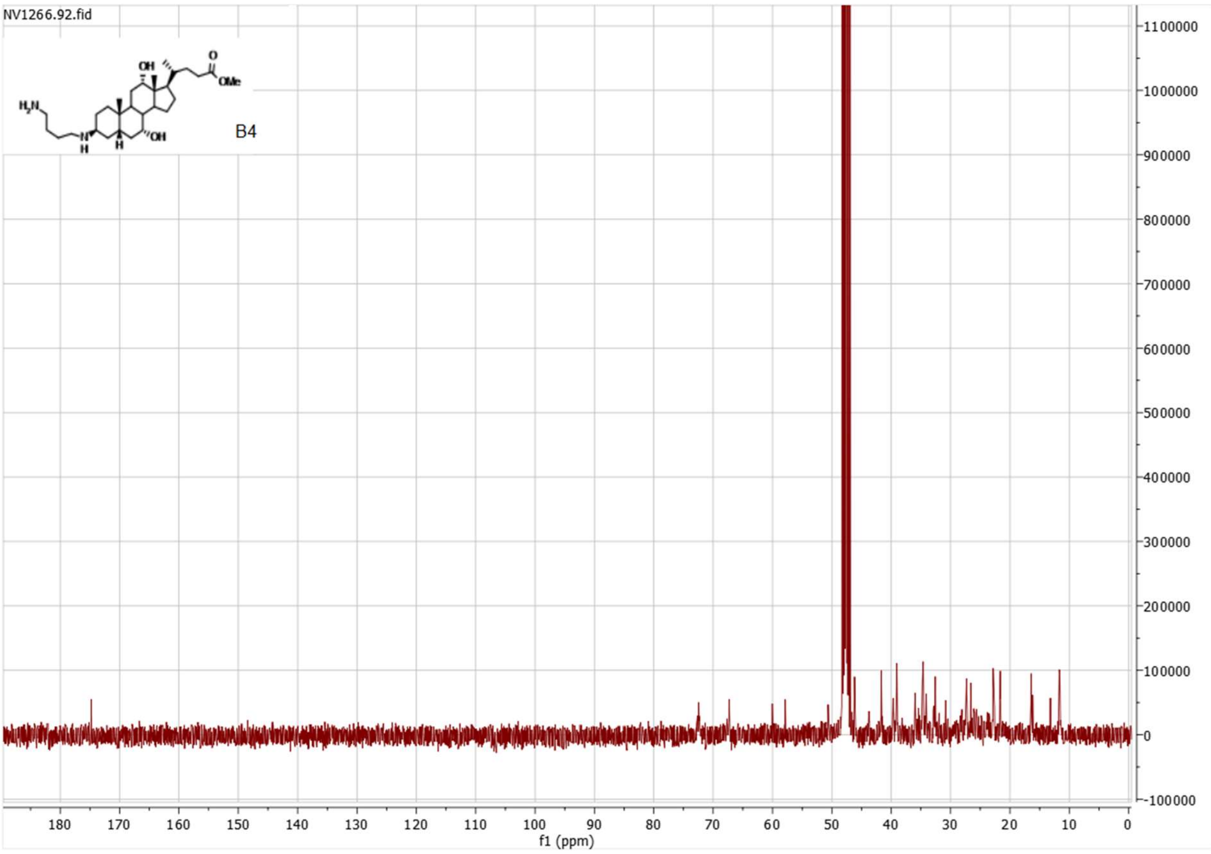
NV1266.92.fid
B4
f1 (ppm)

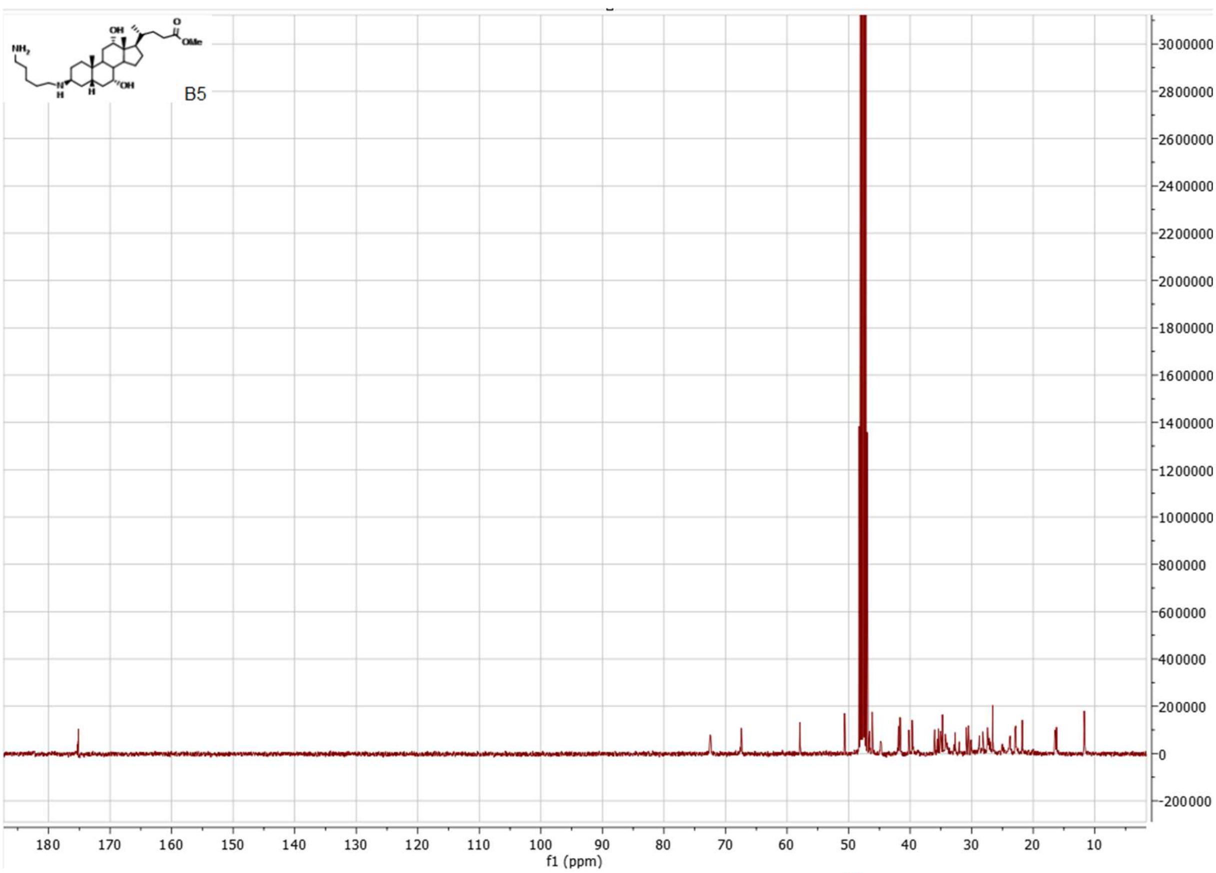

B5
f1 (ppm)


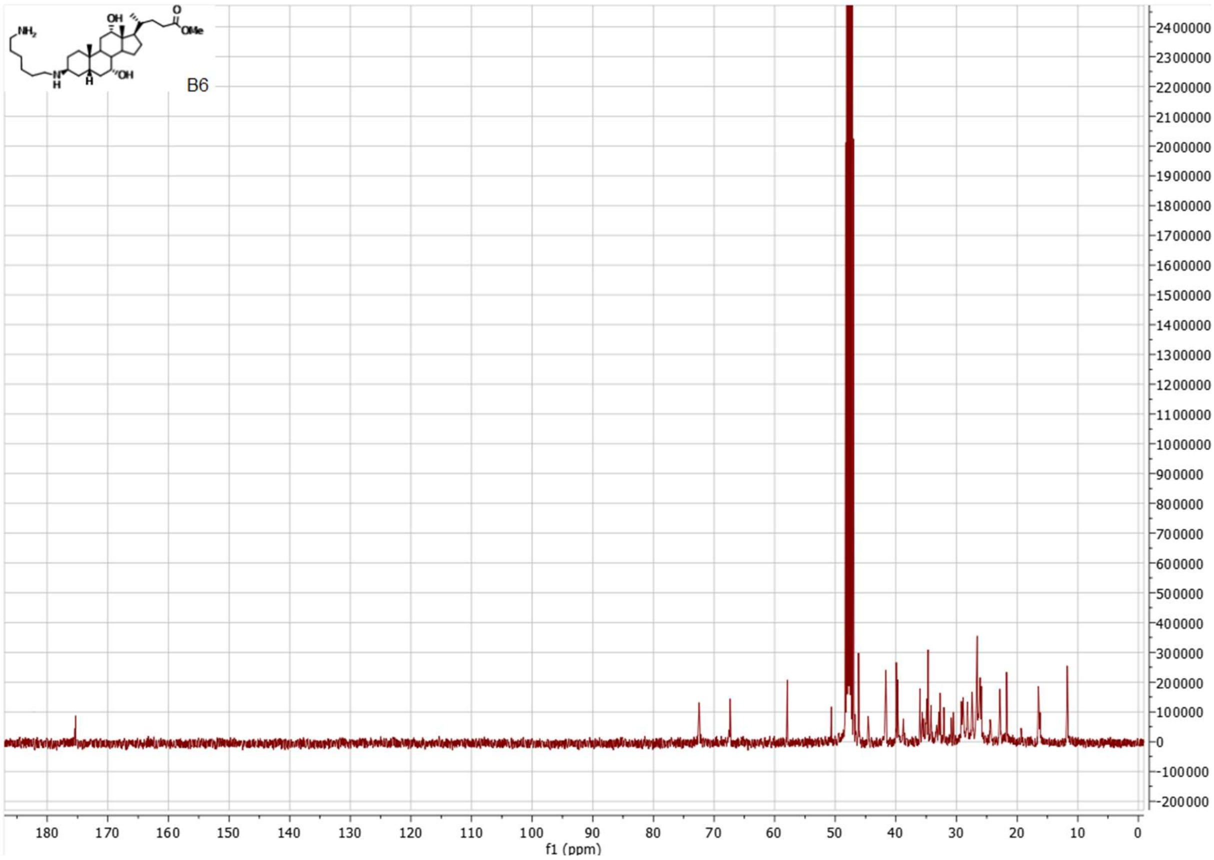

B6
f1 (ppm)

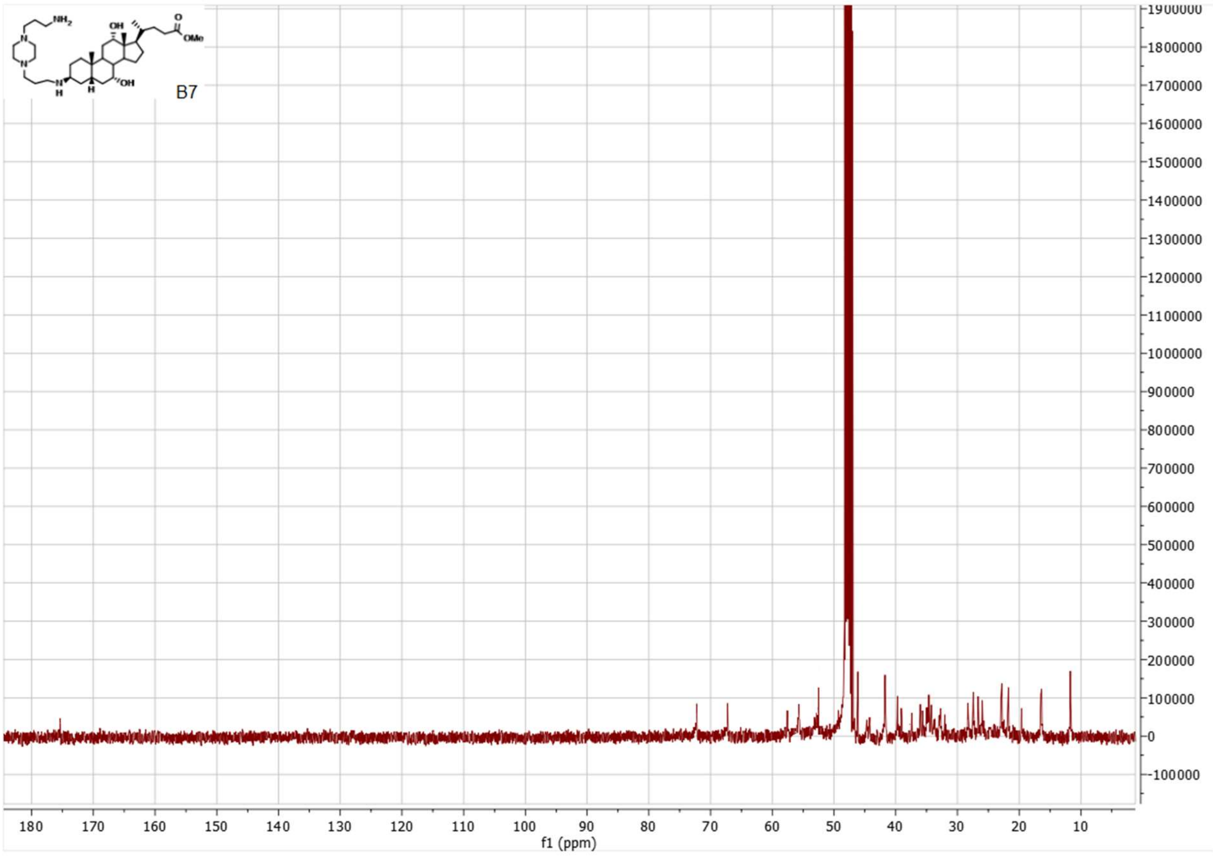

NH2
OH
OMe
N
H
OH
180 170 160 150 140 130 120 110 100 90 80 70 60 50 40 30 20 10
f1 (ppm)
1900000 1800000 1700000 1600000 1500000 1400000 1300000 1200000 1100000 1000000 900000 800000 700000 600000 500000 400000 300000 200000 100000 0 -100000


B7

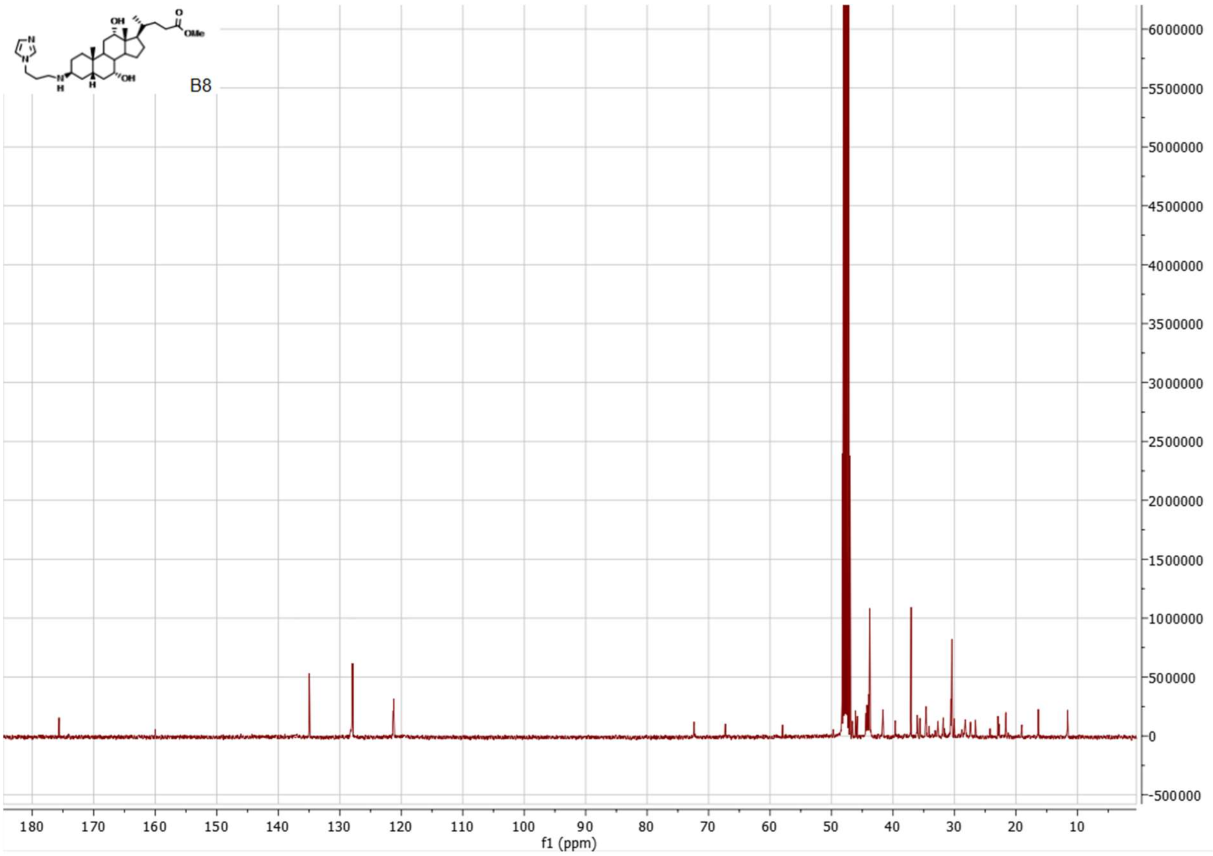

N
OH
OMe
H
OH
180 170 160 150 140 130 120 110 100 90 80 70 60 50 40 30 20 10
f1 (ppm)
6000000 5500000 5000000 4500000 4000000 3500000 3000000 2500000 2000000 1500000 1000000 500000 0 -500000


B8

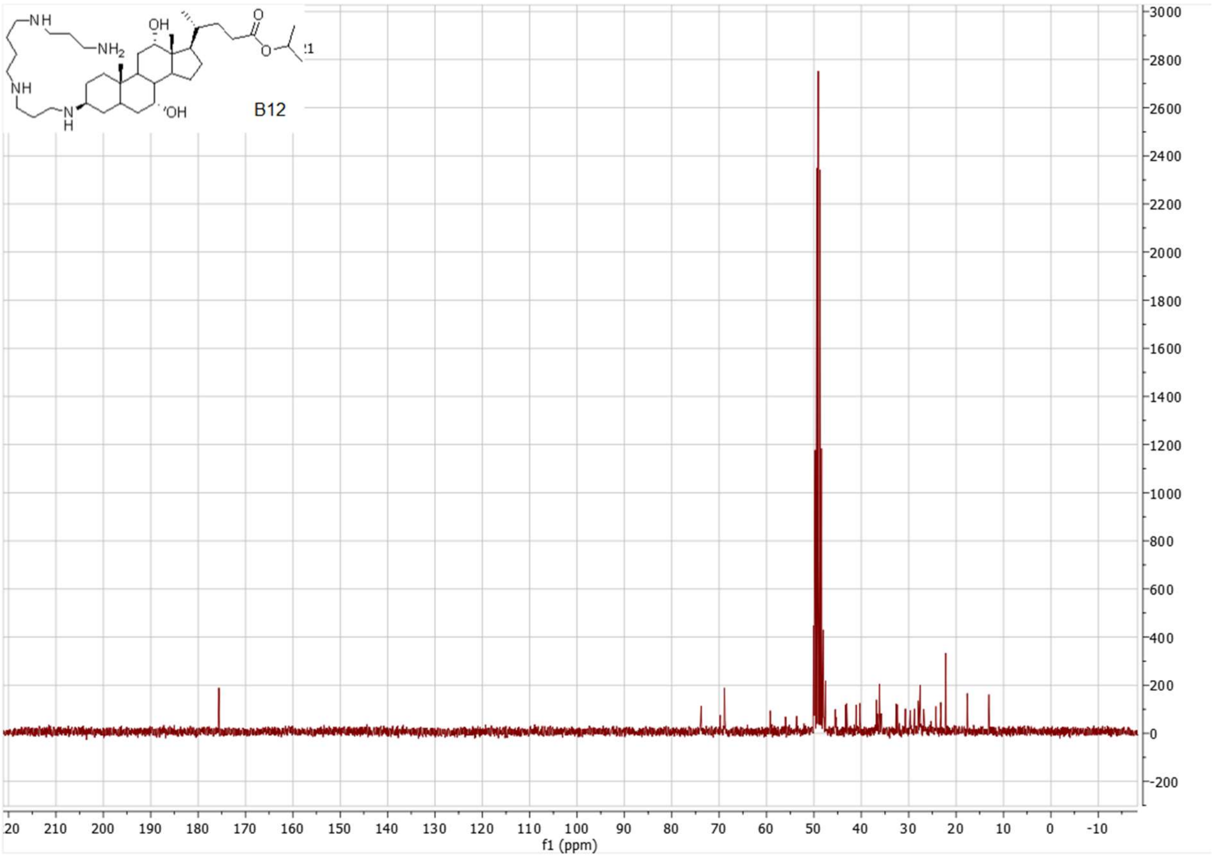
B12
f1 (ppm)

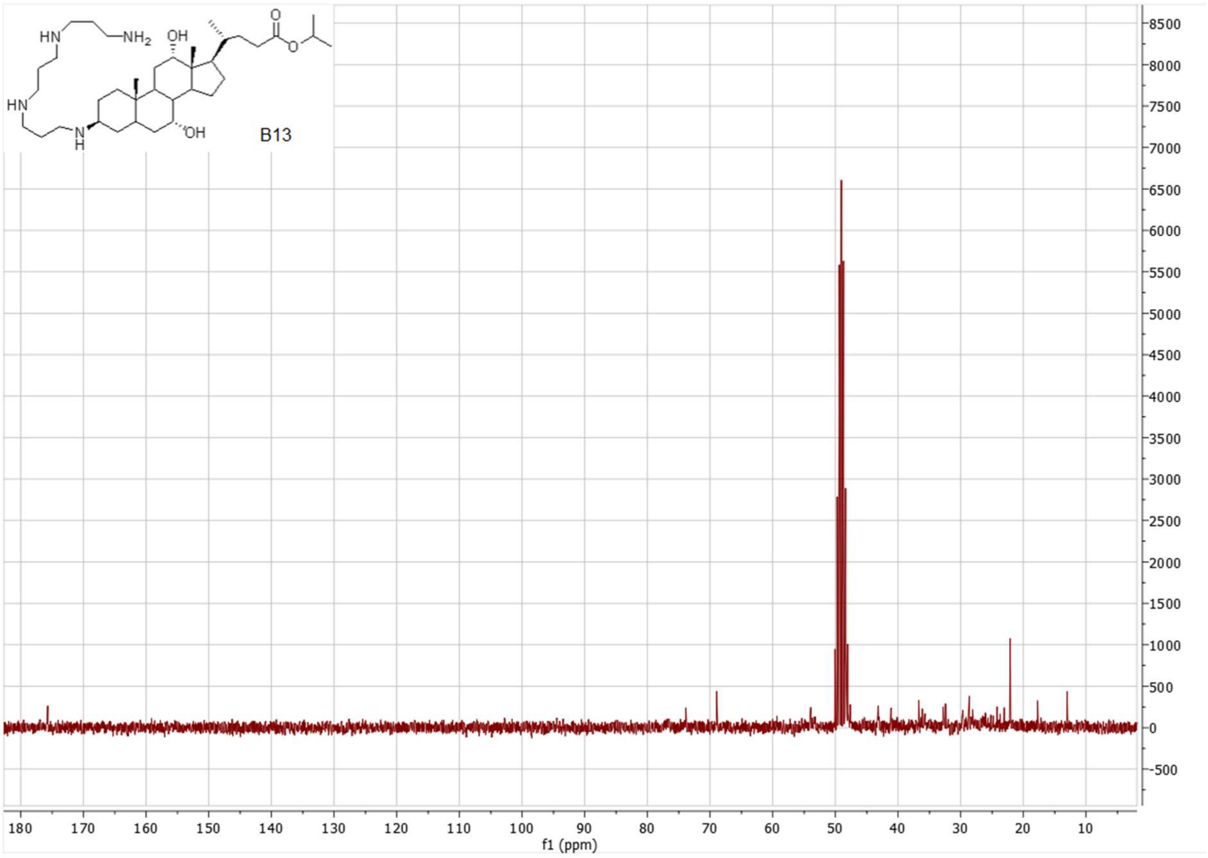
B13
f1 (ppm)

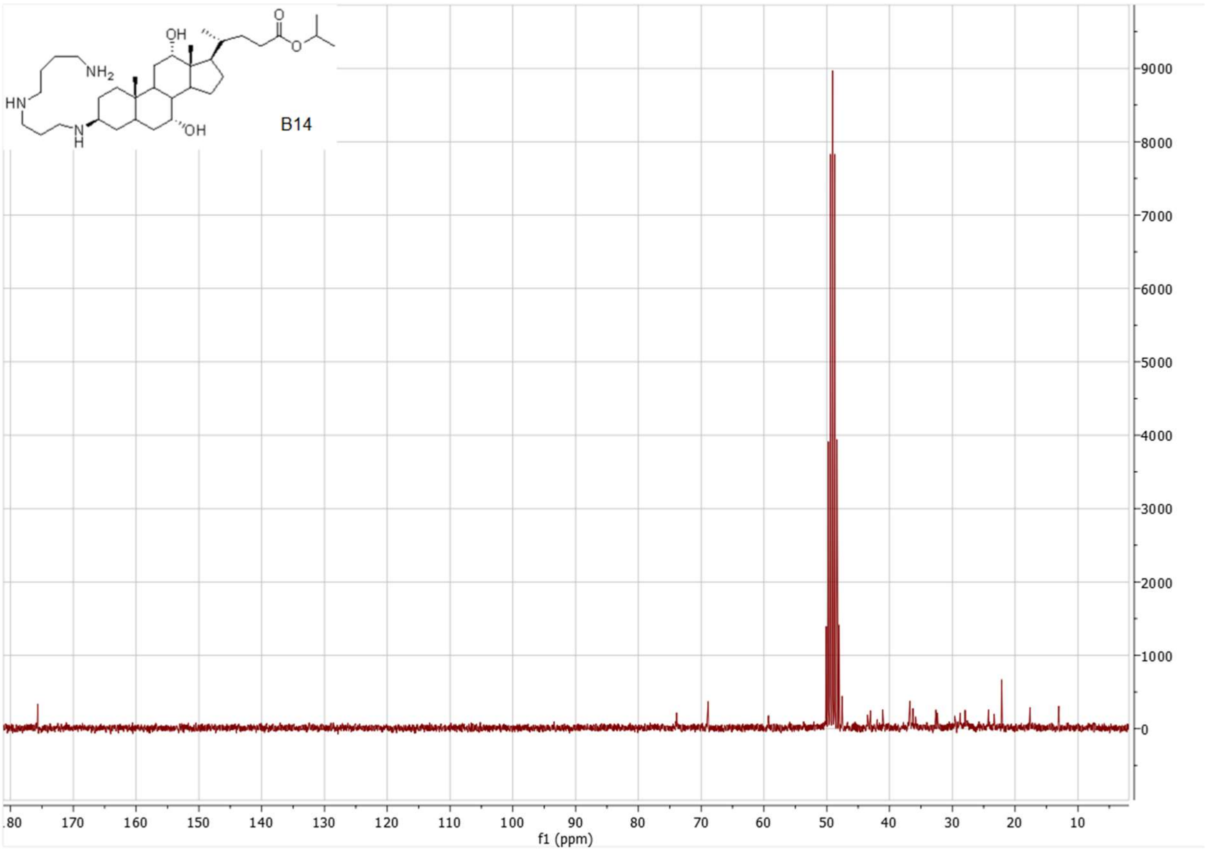
B14
f1 (ppm)

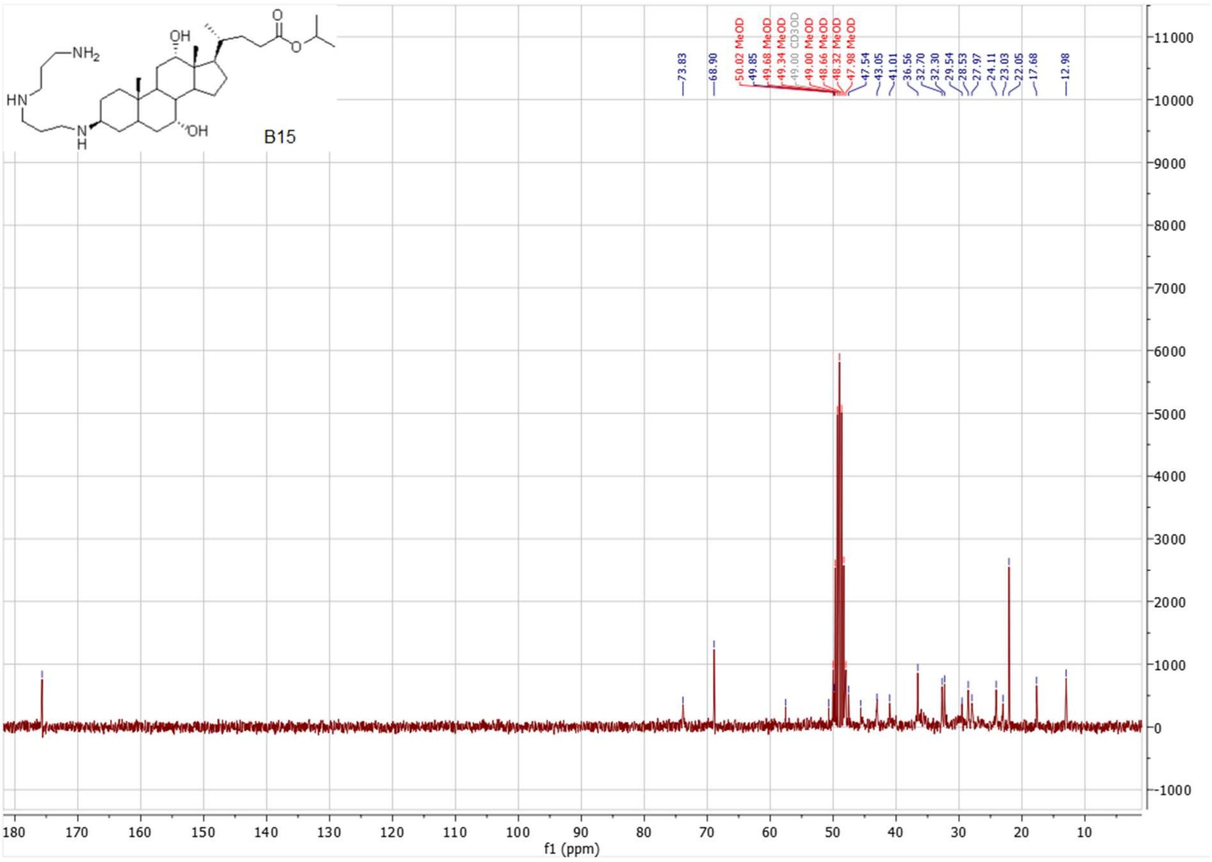
B15
73.83
68.90
50.02 MeOD
49.85
49.68 MeOD
49.34 MeOD
49.00 CD3OD
49.00 MeOD
48.66 MeOD
48.32 MeOD
47.98 MeOD
47.54
43.05
41.01
36.56
32.70
32.30
29.54
28.53
27.97
24.11
23.03
22.05
17.68
12.98
f1 (ppm)

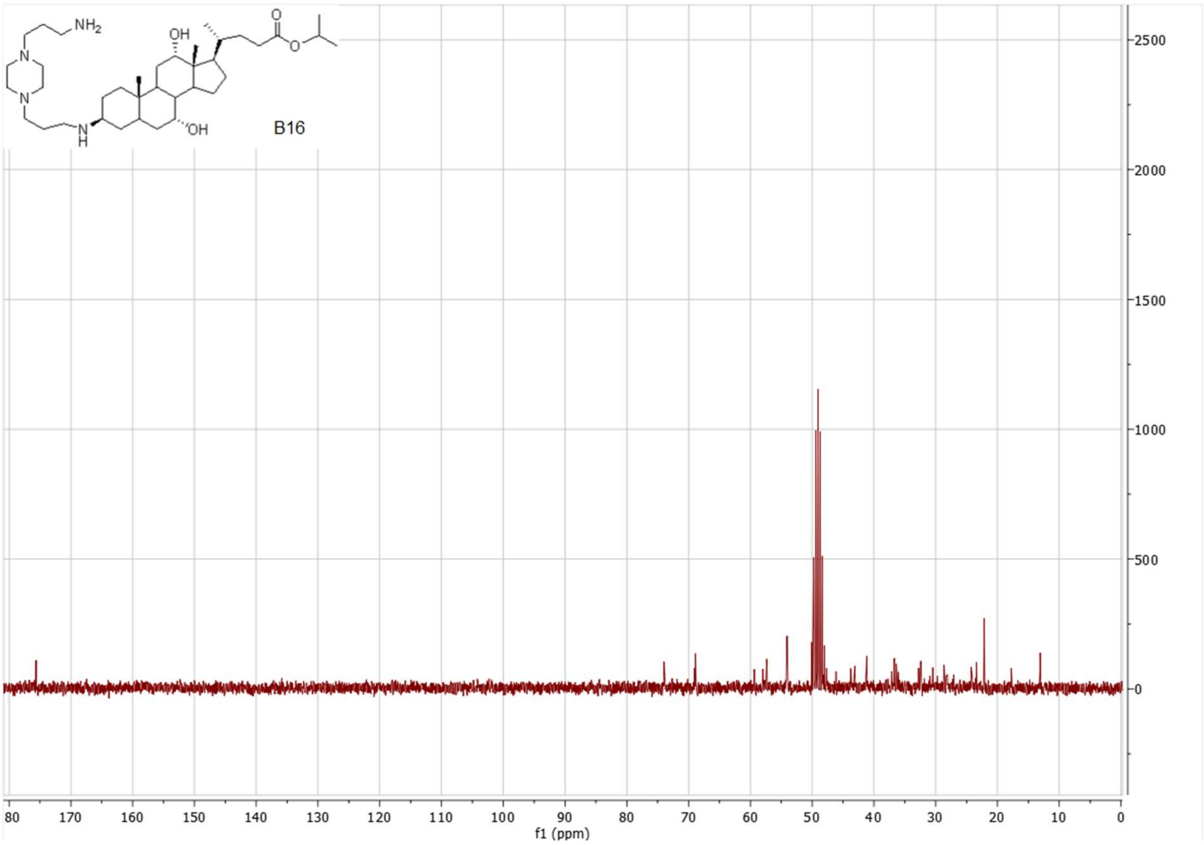
B16
f1 (ppm)

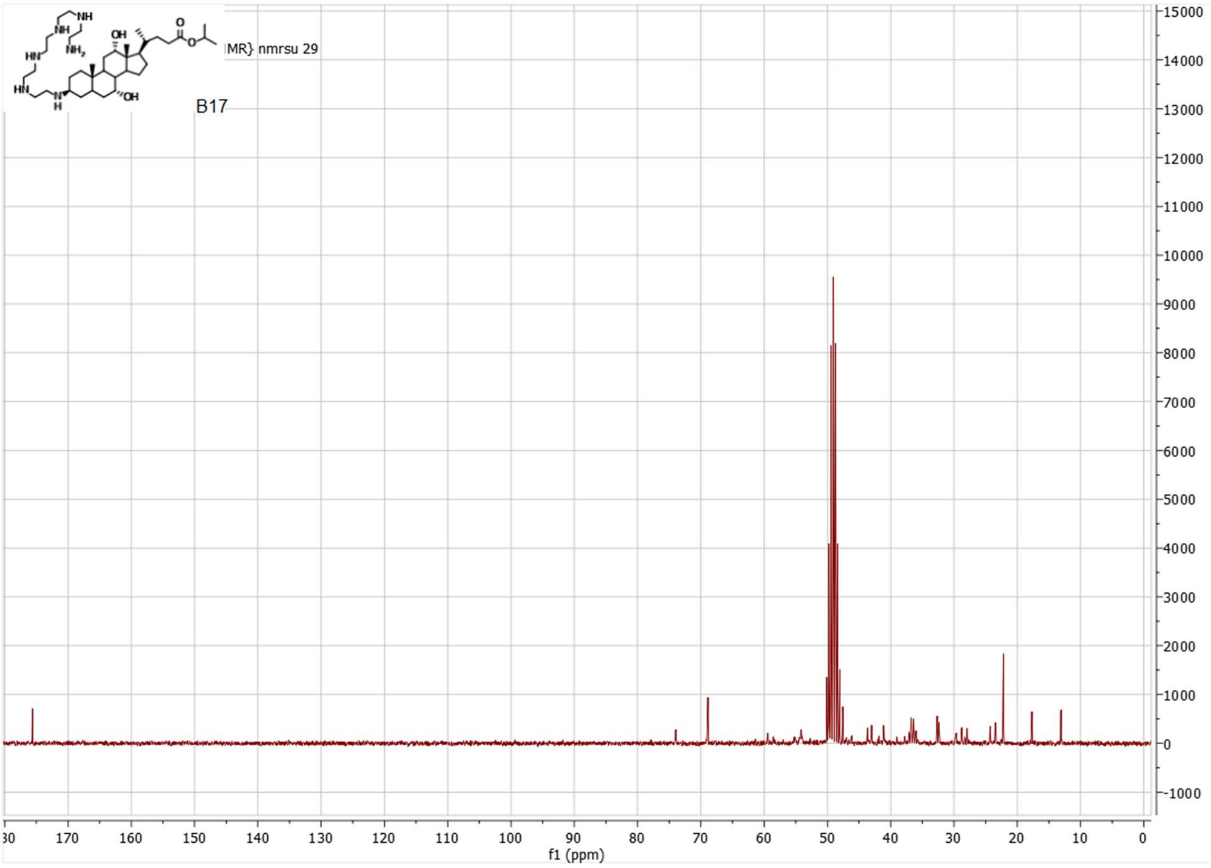
MR} nmrsu 29
B17
f1 (ppm)

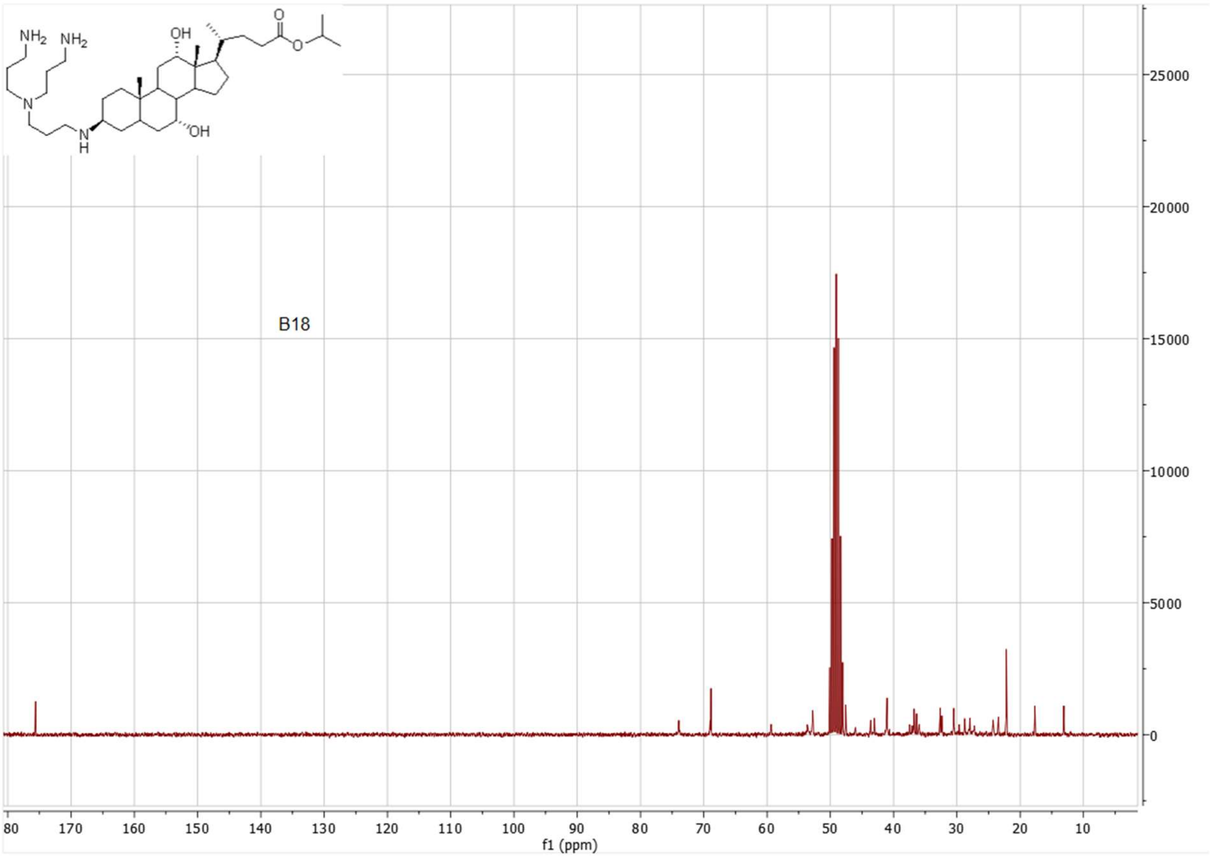
B18
f1 (ppm)

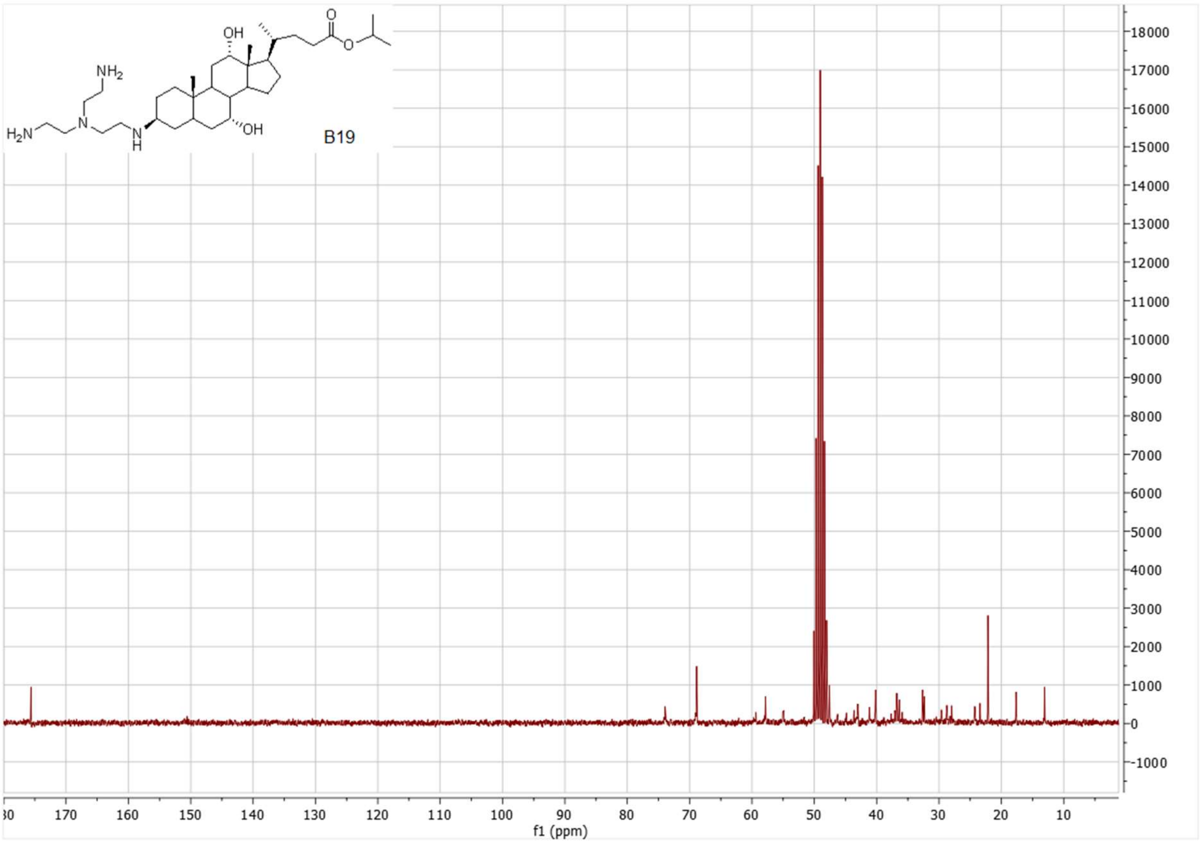
B19
f1 (ppm)

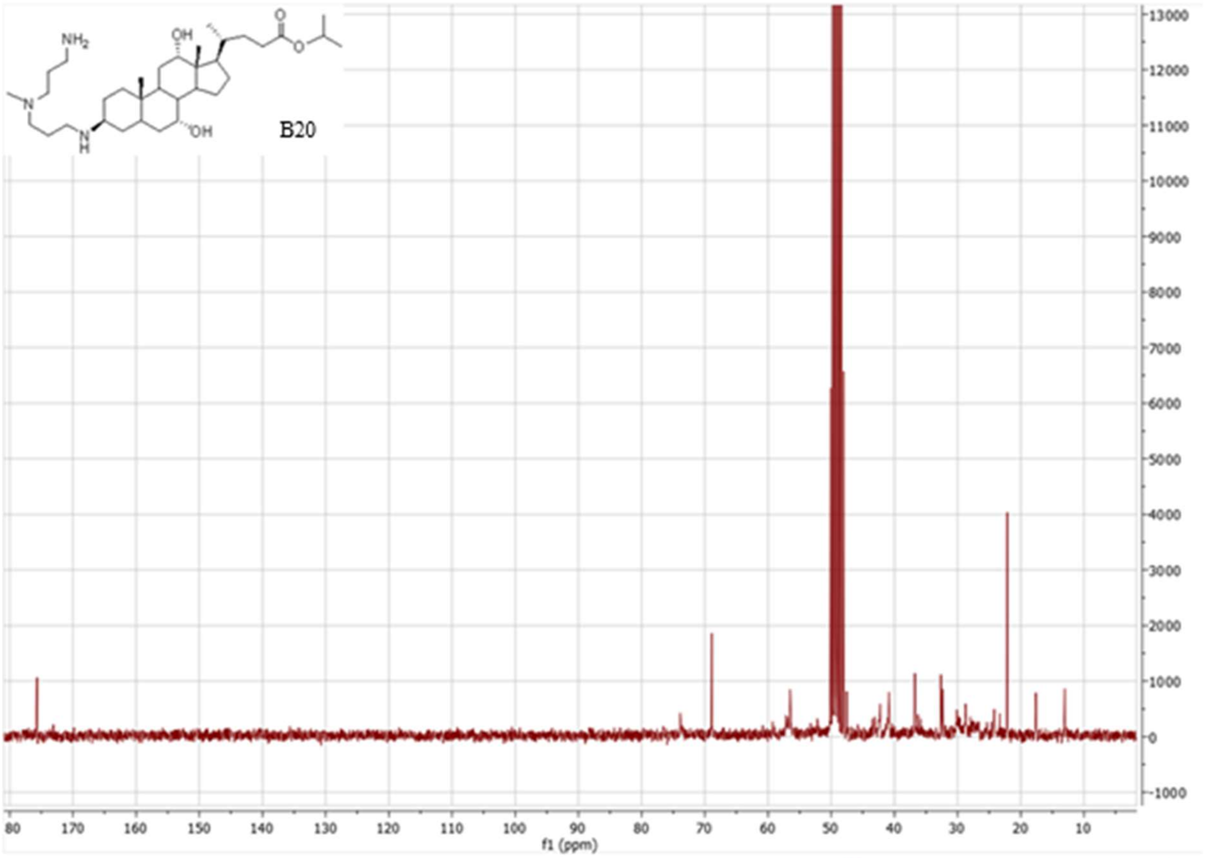

B20
f1 (ppm)


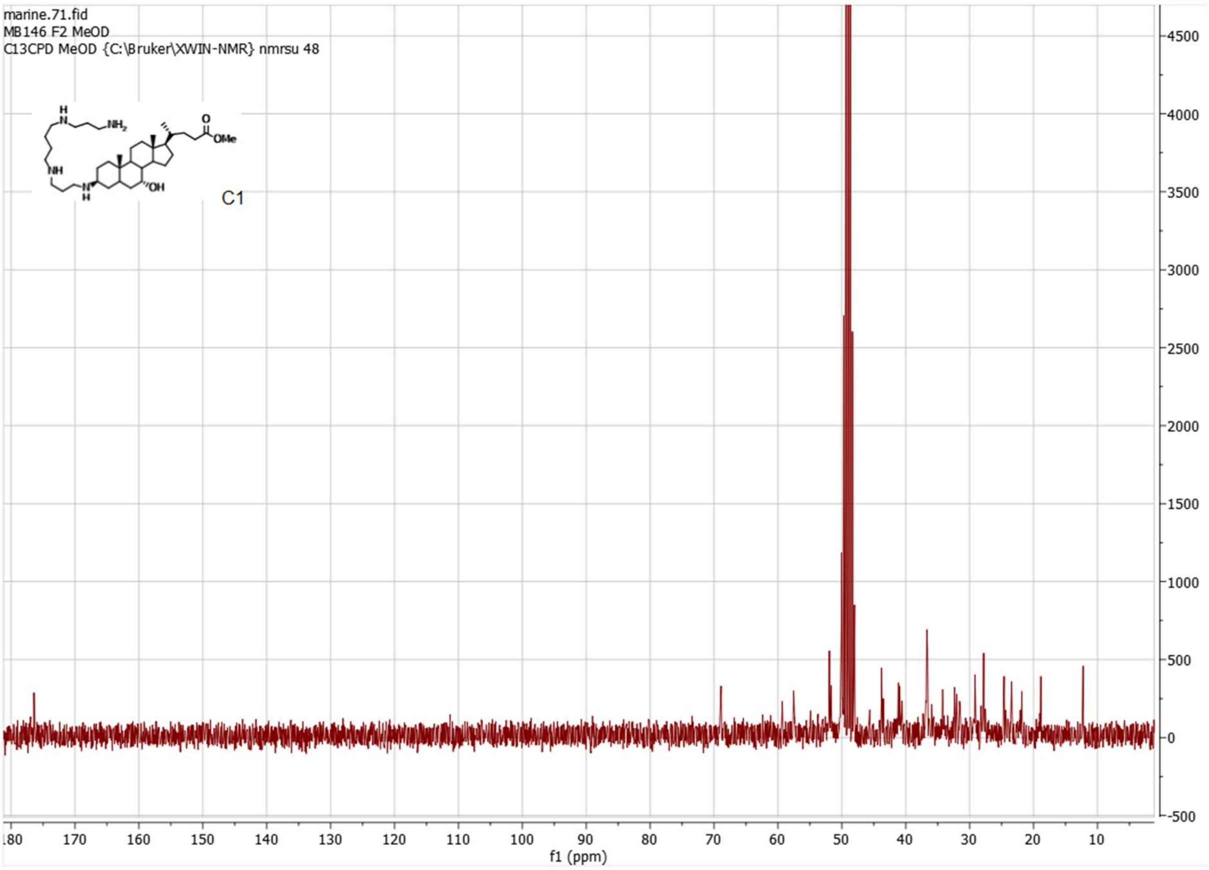

marine.71.fid
MB146 F2 MeOD
C13CPD MeOD {C:\Bruker\XWIN-NMR} nmrsu 48
C1
f1 (ppm)

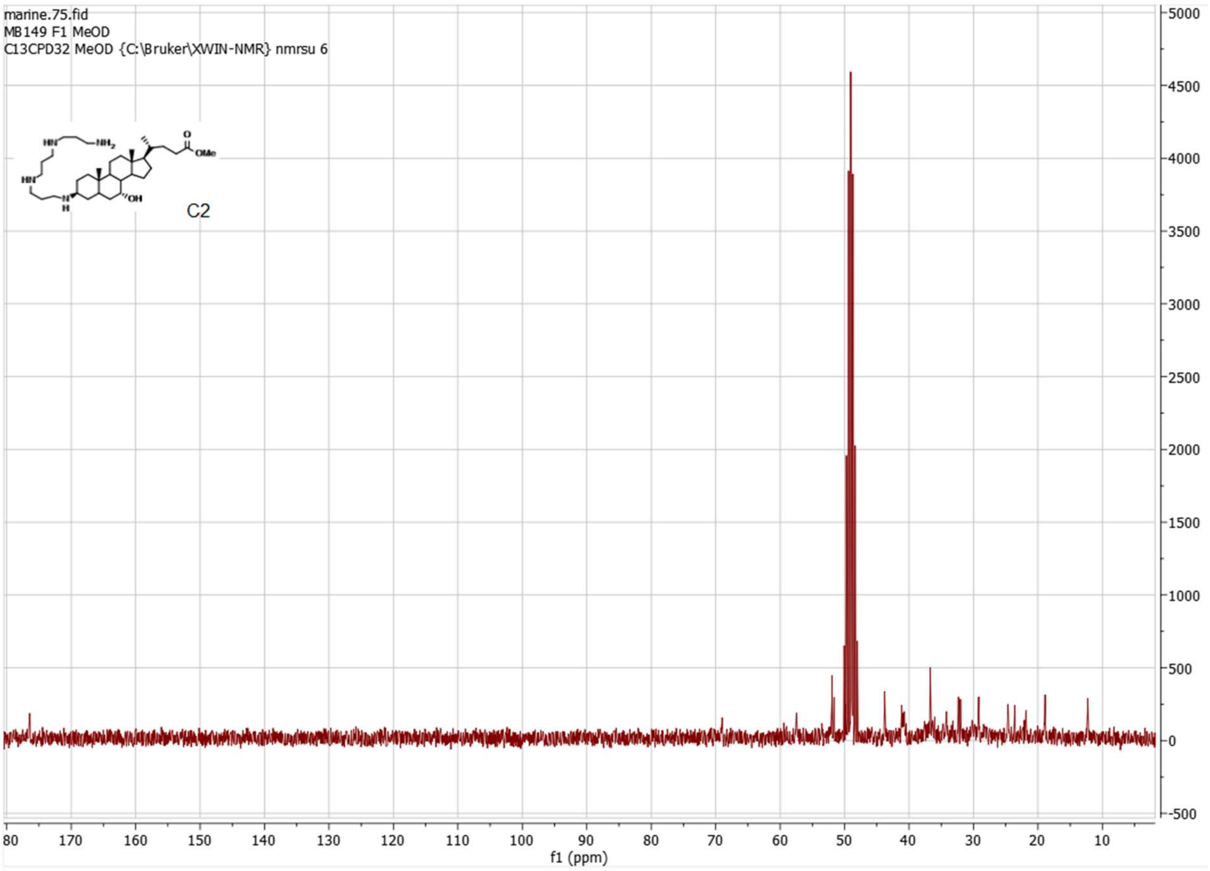
marine.75.fid
MB149 F1 MeOD
C13CPD32 MeOD {C:\Bruker\XWIN-NMR} nmrsu 6
C2
f1 (ppm)

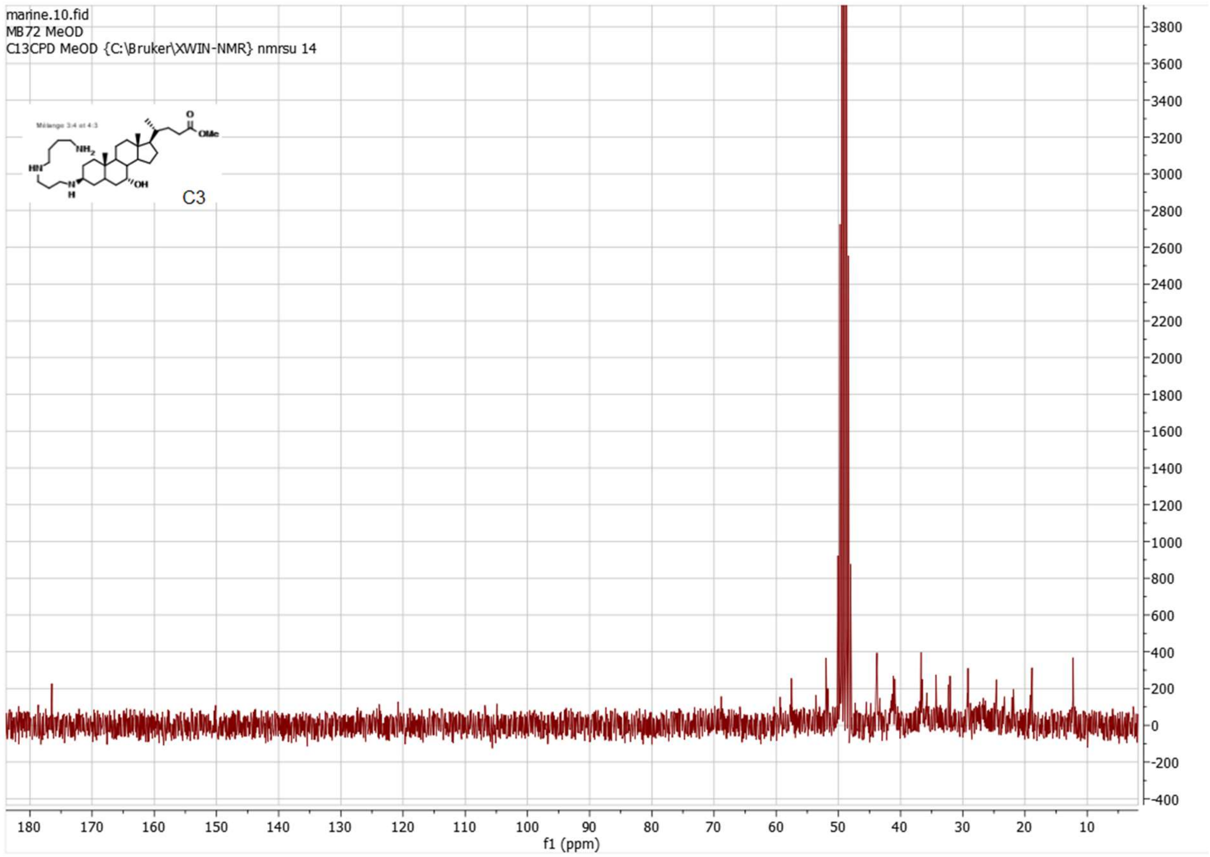
marine.10.fid
MB72 MeOD
C13CPD MeOD {C:\Bruker\XWIN-NMR} nmrsu 14
C3
f1 (ppm)

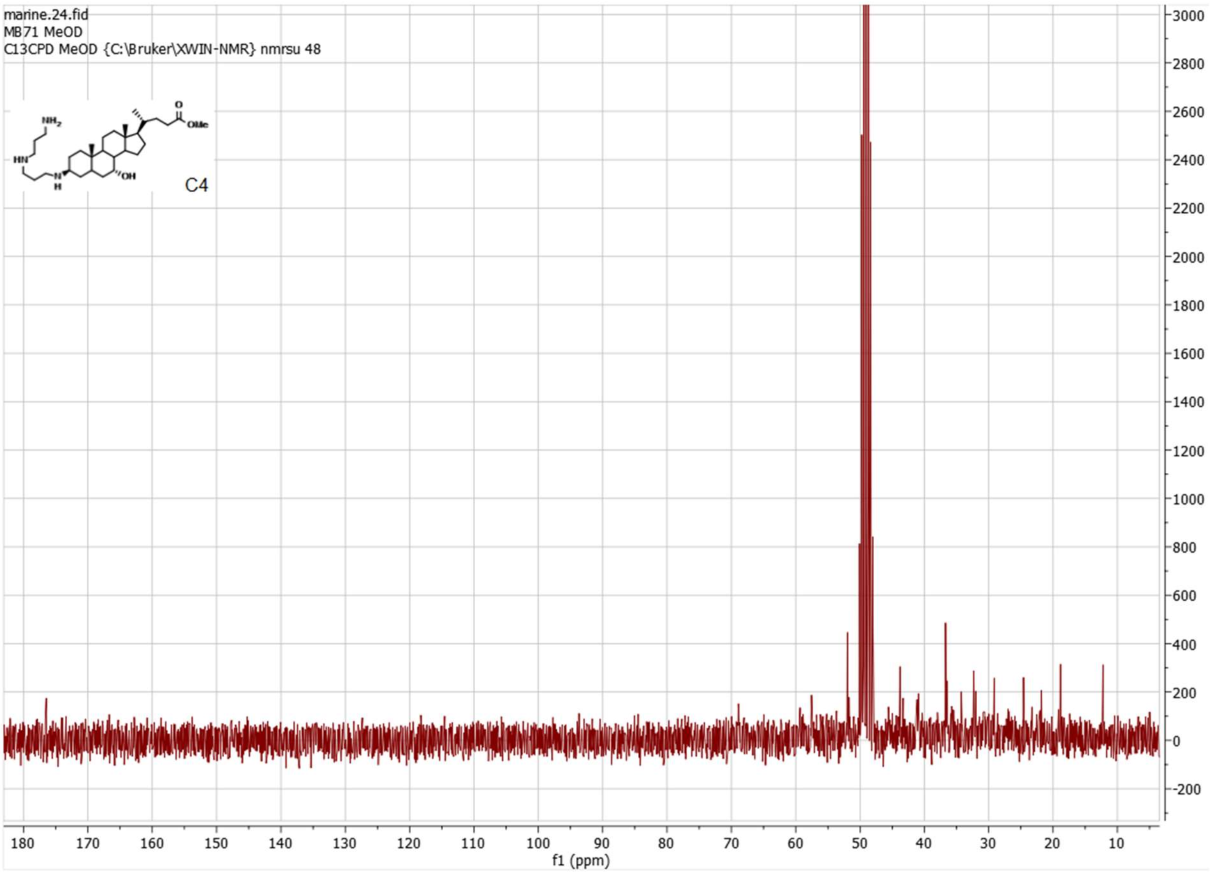
marine.24.fid
MB71 MeOD
C13CPD MeOD {C:\Bruker\XWIN-NMR} nmrsu 48
C4
f1 (ppm)

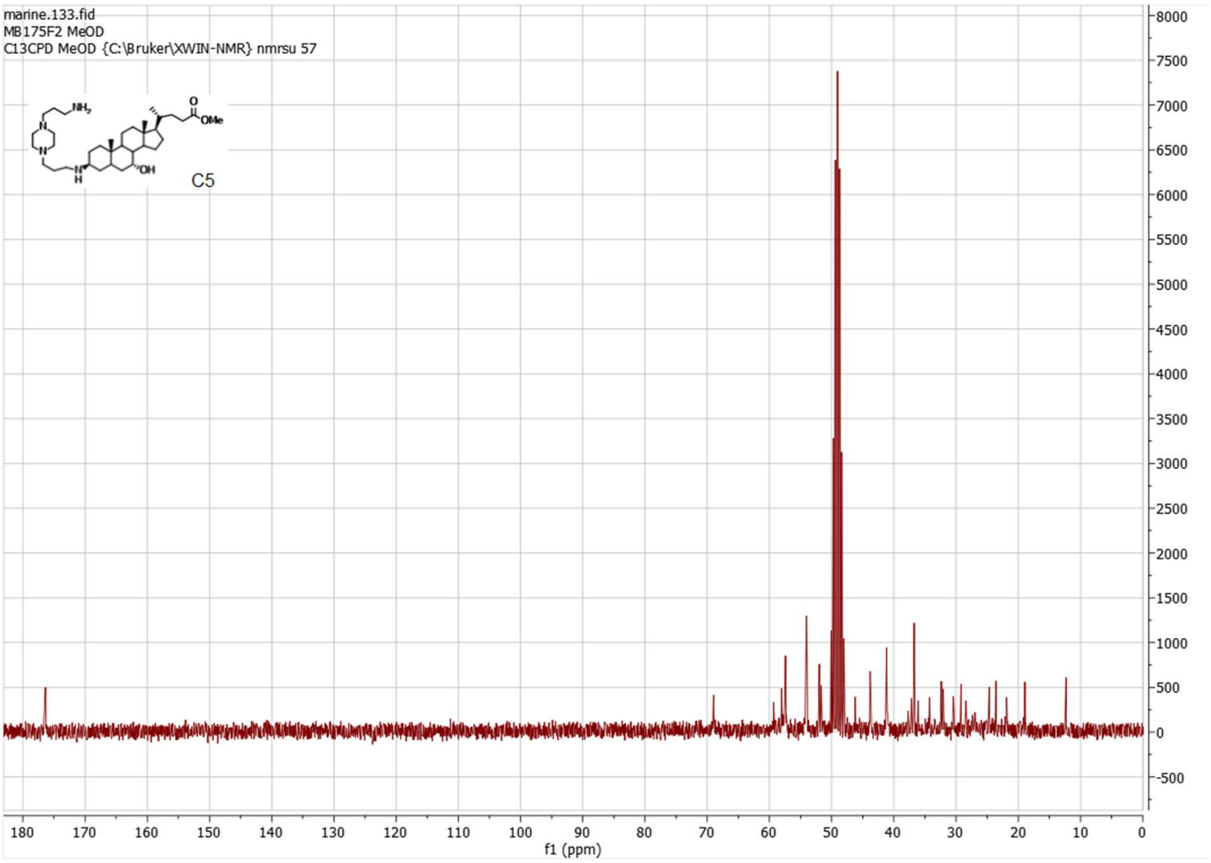
marine.133.fid
MB175F2 MeOD
C13CPD MeOD {C:\Bruker\XWIN-NMR} nmrsu 57
C5
f1 (ppm)

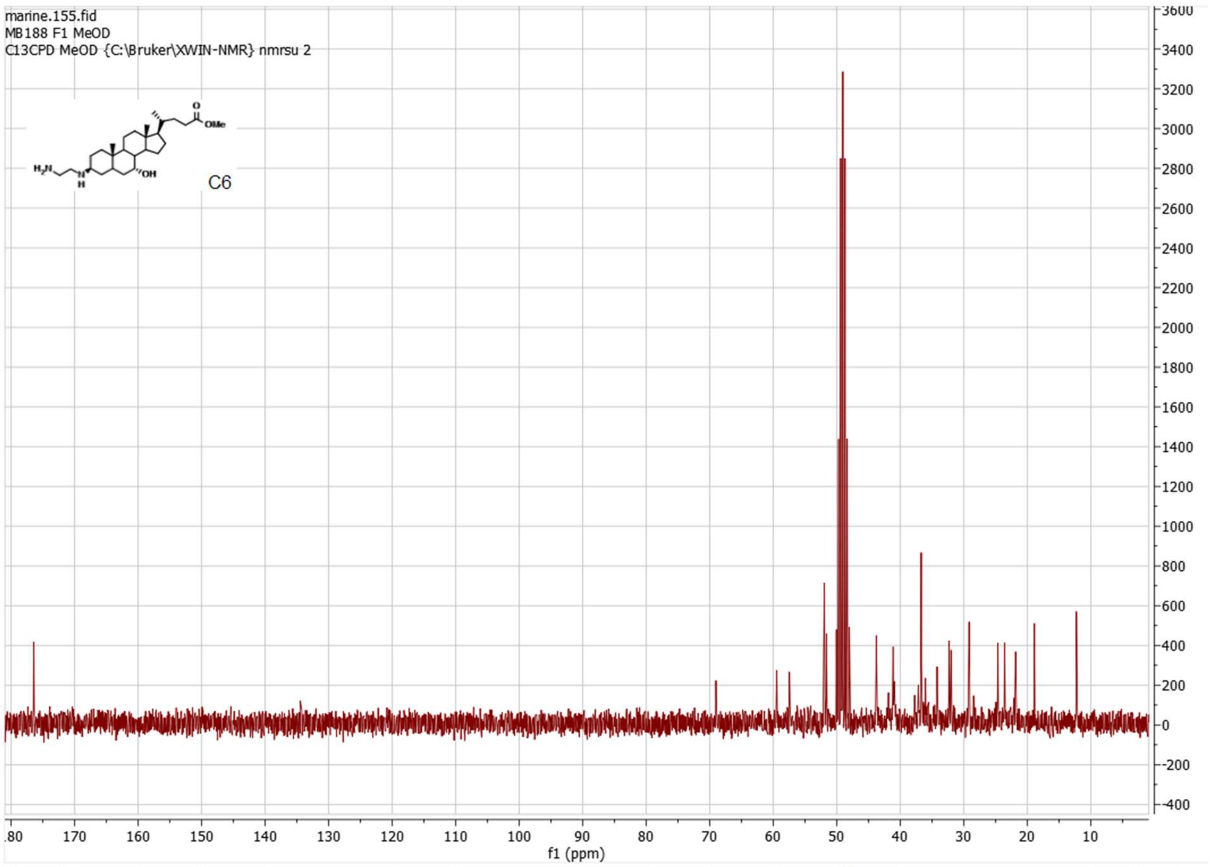
marine.155.fid
MB188 F1 MeOD
C13CPD MeOD {C:\Bruker\XWIN-NMR} nmrsu 2
C6
f1 (ppm)

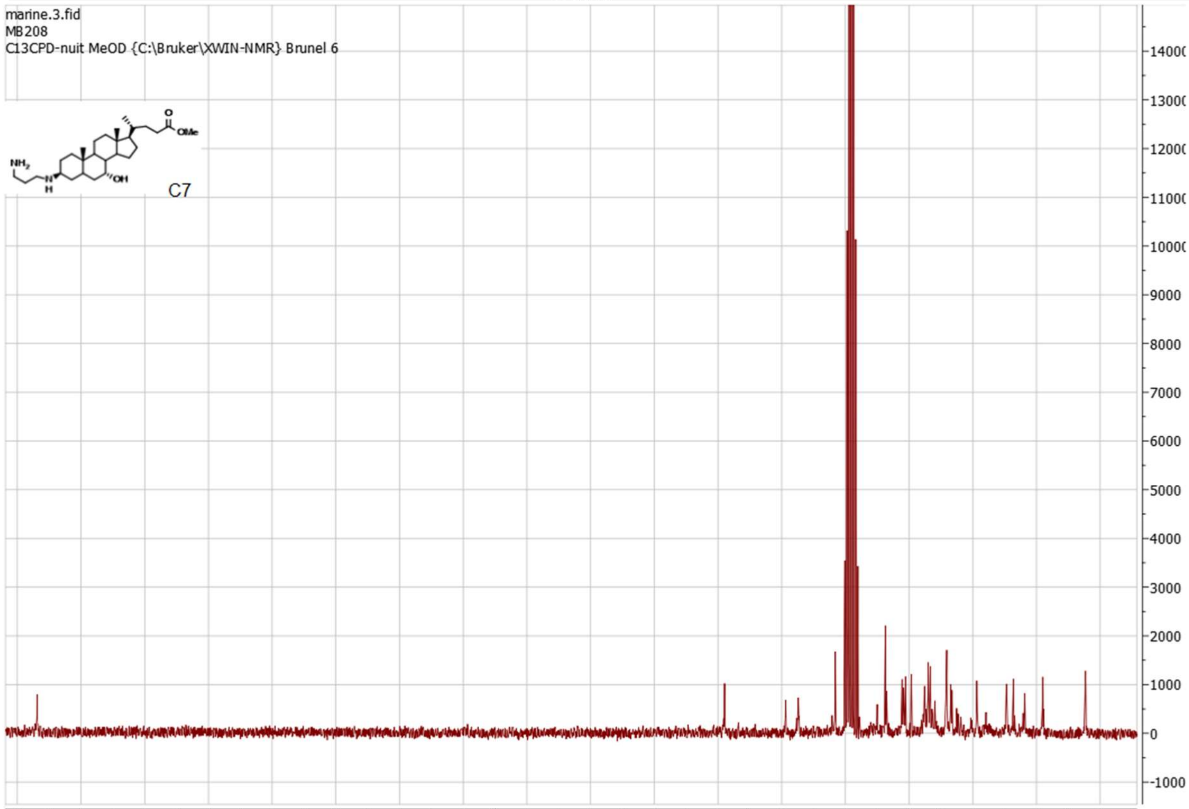
marine.3.fid
MB208
C13CPD-nuit MeOD {C:\Bruker\XWIN-NMR} Brunel 6
C7
f1 (ppm)

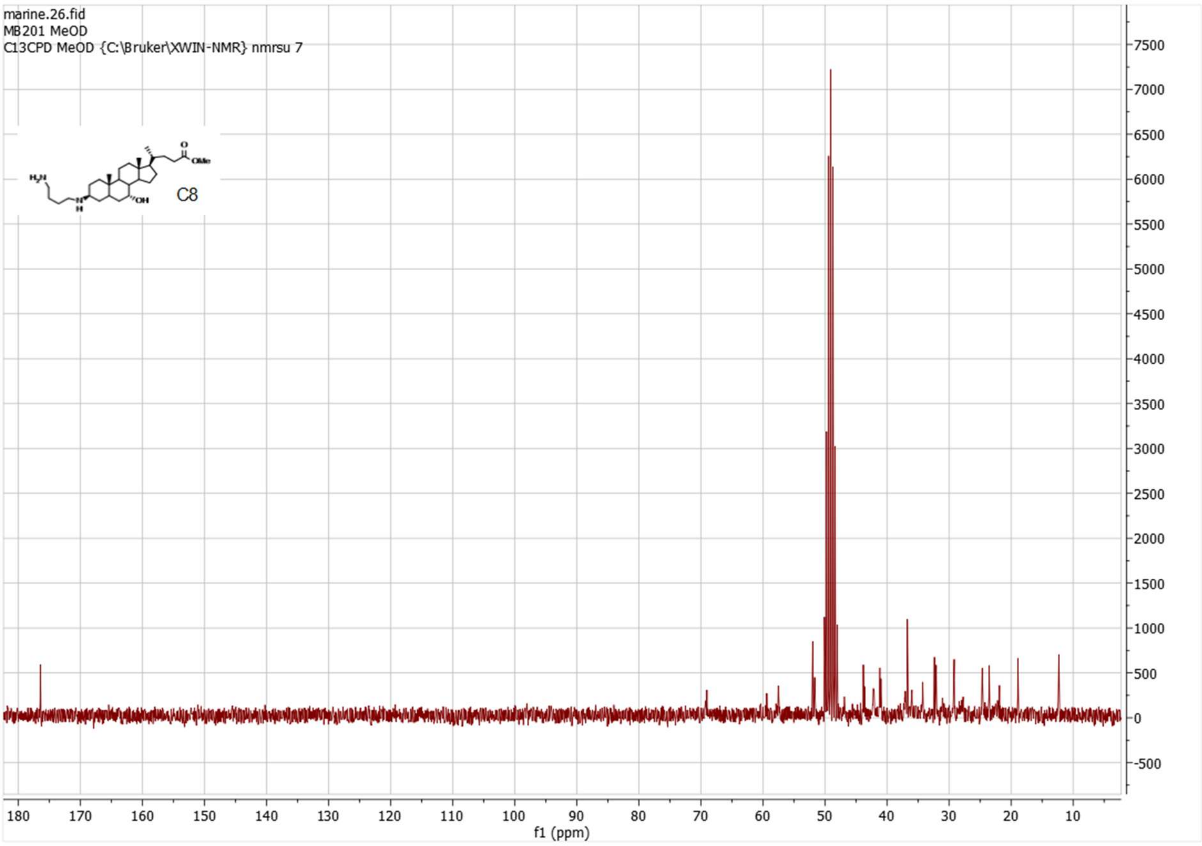
marine.26.fid
MB201 MeOD
C13CPD MeOD {C:\Bruker\XWIN-NMR} nmrsu 7
C8
f1 (ppm)

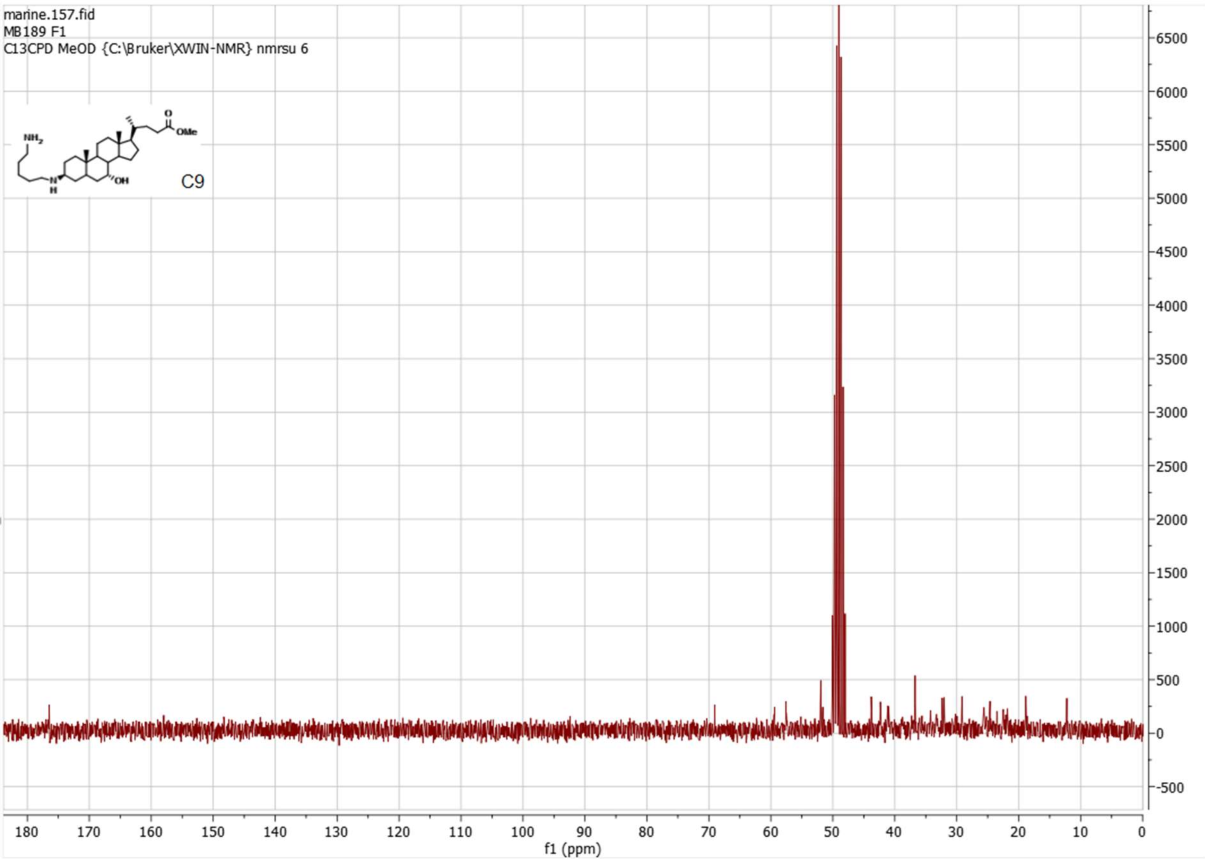
marine.157.fid
MB189 F1
C13CPD MeOD {C:\Bruker\XWIN-NMR} nmrsu 6
C9
f1 (ppm)

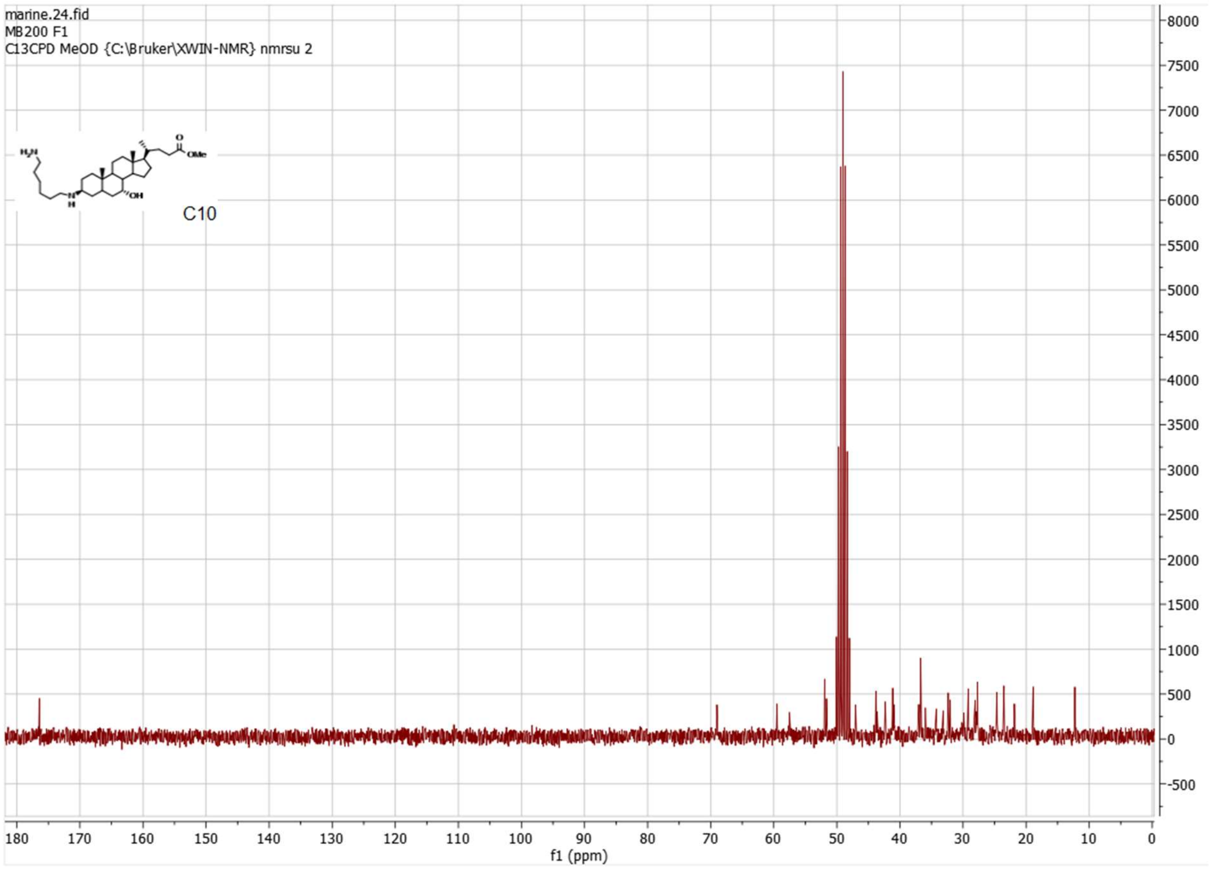
marine.24.fid
MB200 F1
C13CPD MeOD {C:\Bruker\XWIN-NMR} nmrsu 2
C10
f1 (ppm)

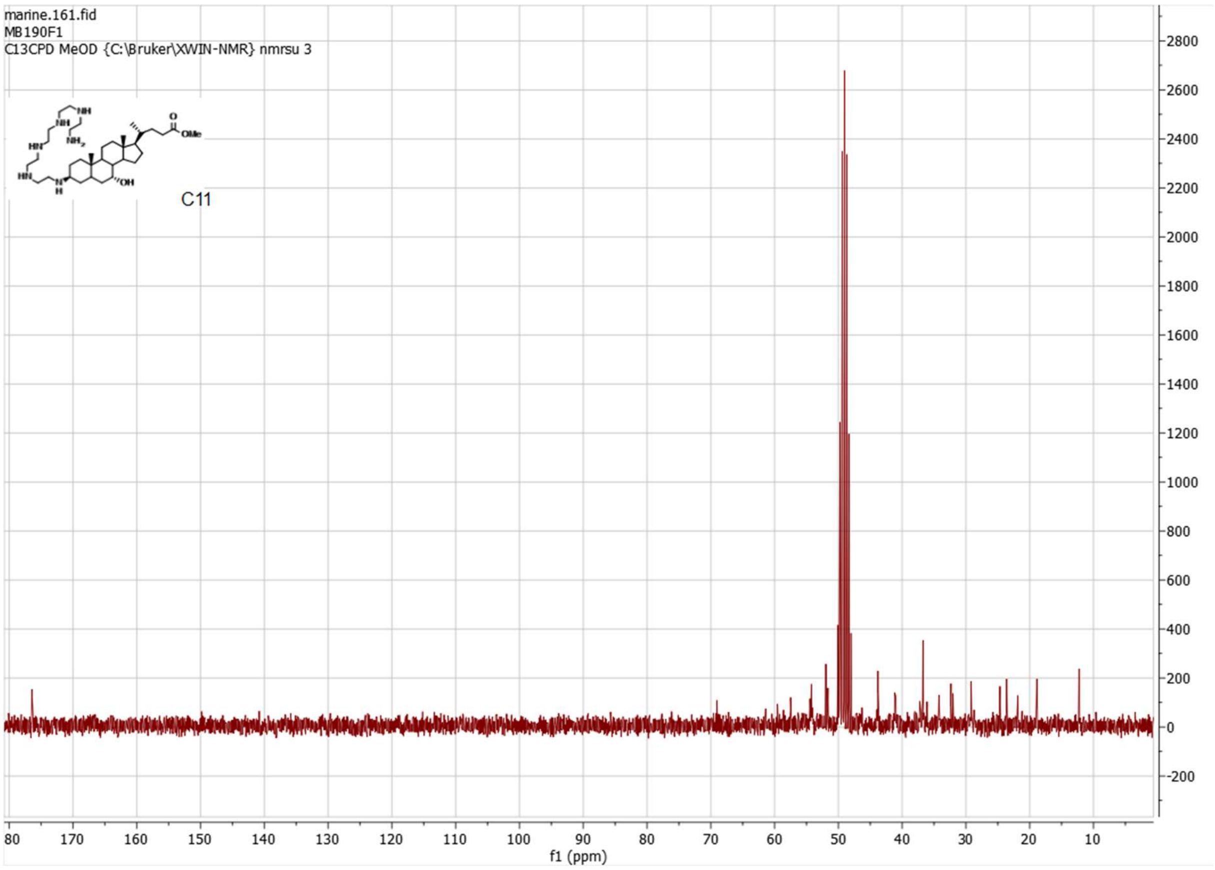
marine.161.fid
MB190F1
C13CPD MeOD {C:\Bruker\XWIN-NMR} nmrsu 3
C11
f1 (ppm)

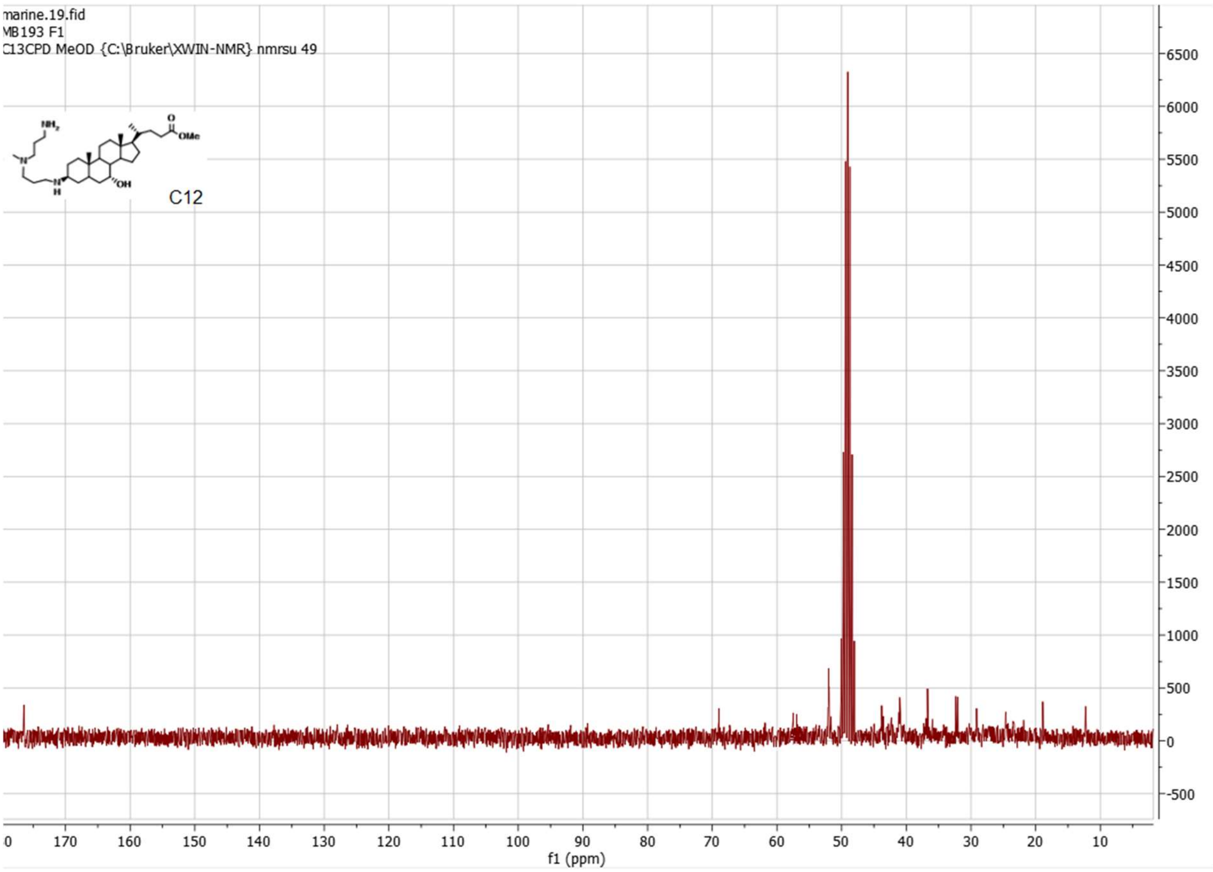
marine.19.fid
MB193 F1
C13CPD MeOD {C:\Bruker\XWIN-NMR} nmrsu 49
C12
f1 (ppm)

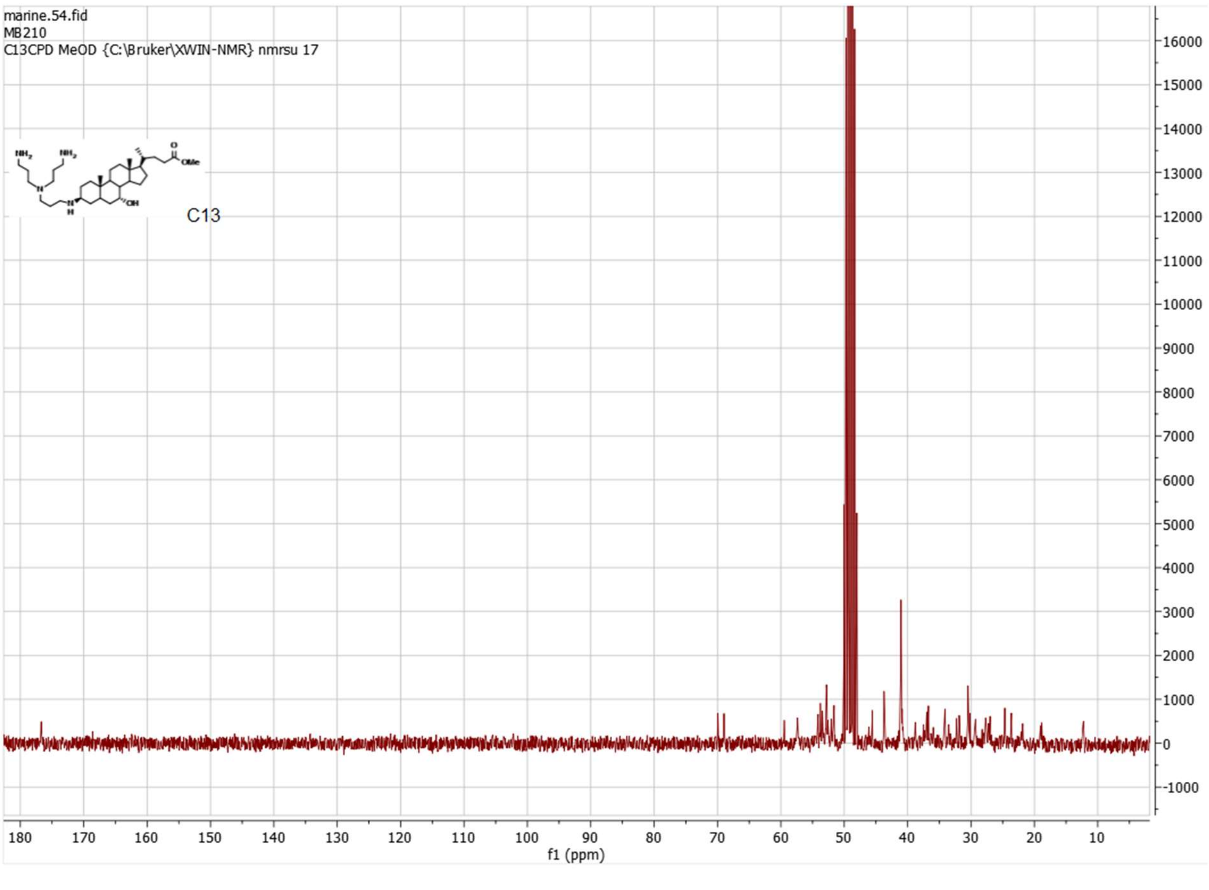
marine.54.fid
MB210
C13CPD MeOD {C:\Bruker\XWIN-NMR} nmrsu 17
C13
f1 (ppm)

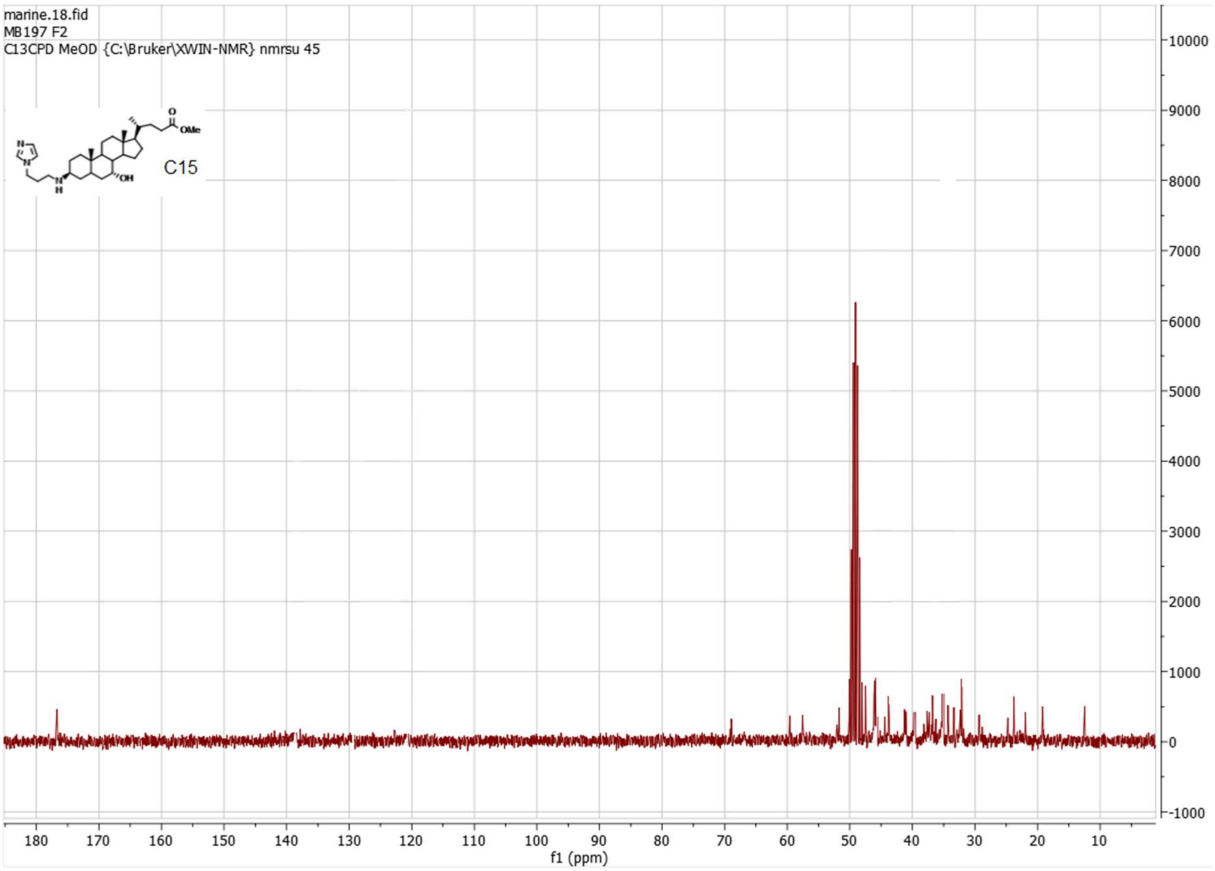
marine.18.fid
MB197 F2
C13CPD MeOD {C:\Bruker\XWIN-NMR} nmrsu 45
C15
f1 (ppm)

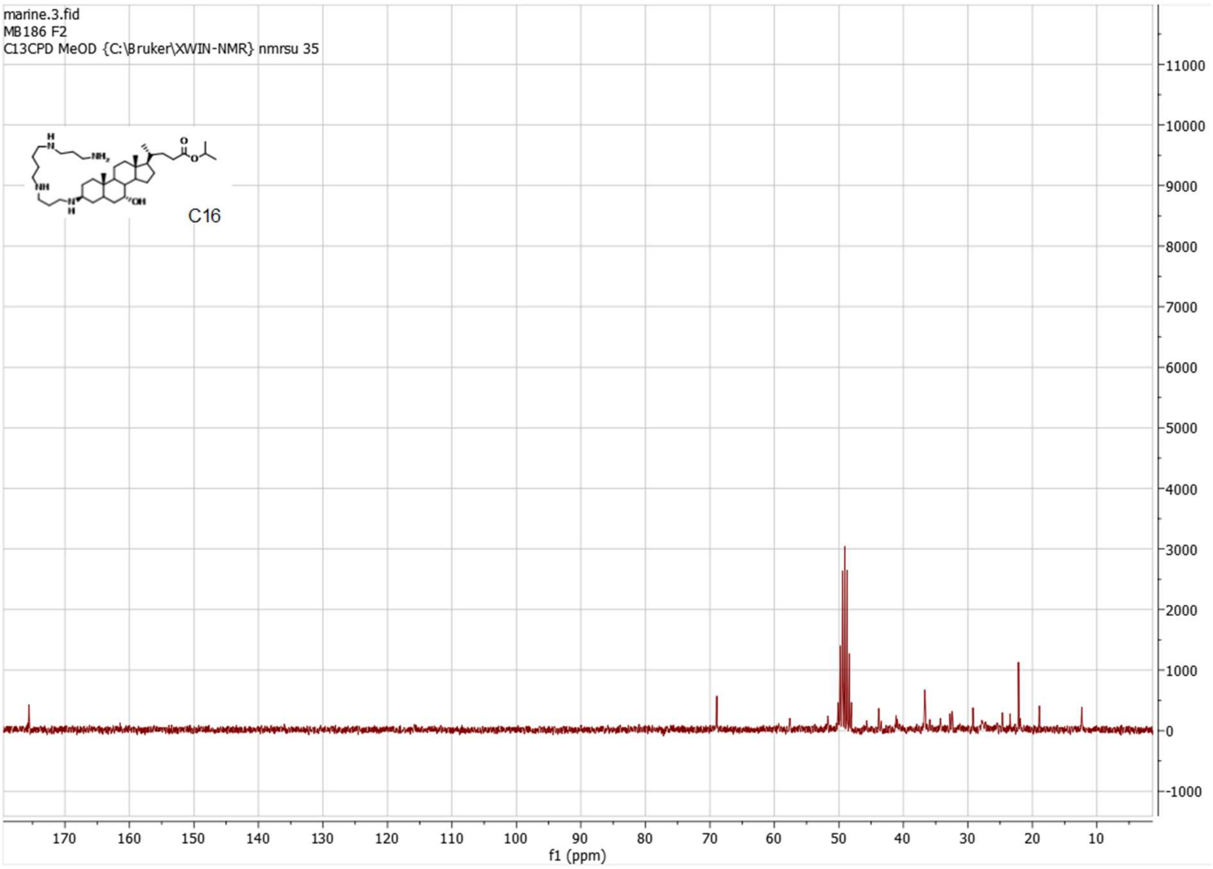
marine.3.fid
MB186 F2
C13CPD MeOD {C:\Bruker\XWIN-NMR} nmrsu 35
C16
f1 (ppm)

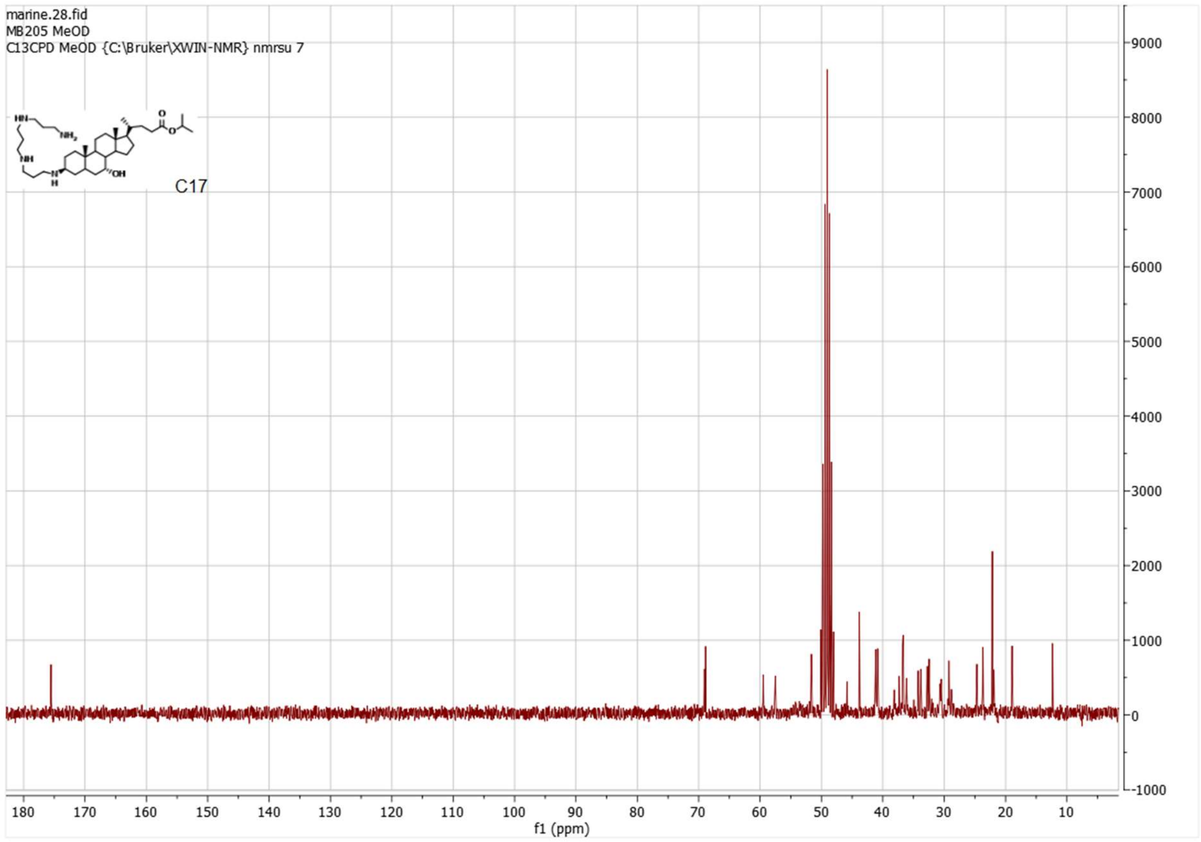
marine.28.fid
MB205 MeOD
C13CPD MeOD {C:\Bruker\XWIN-NMR} nmrsu 7
C17
f1 (ppm)

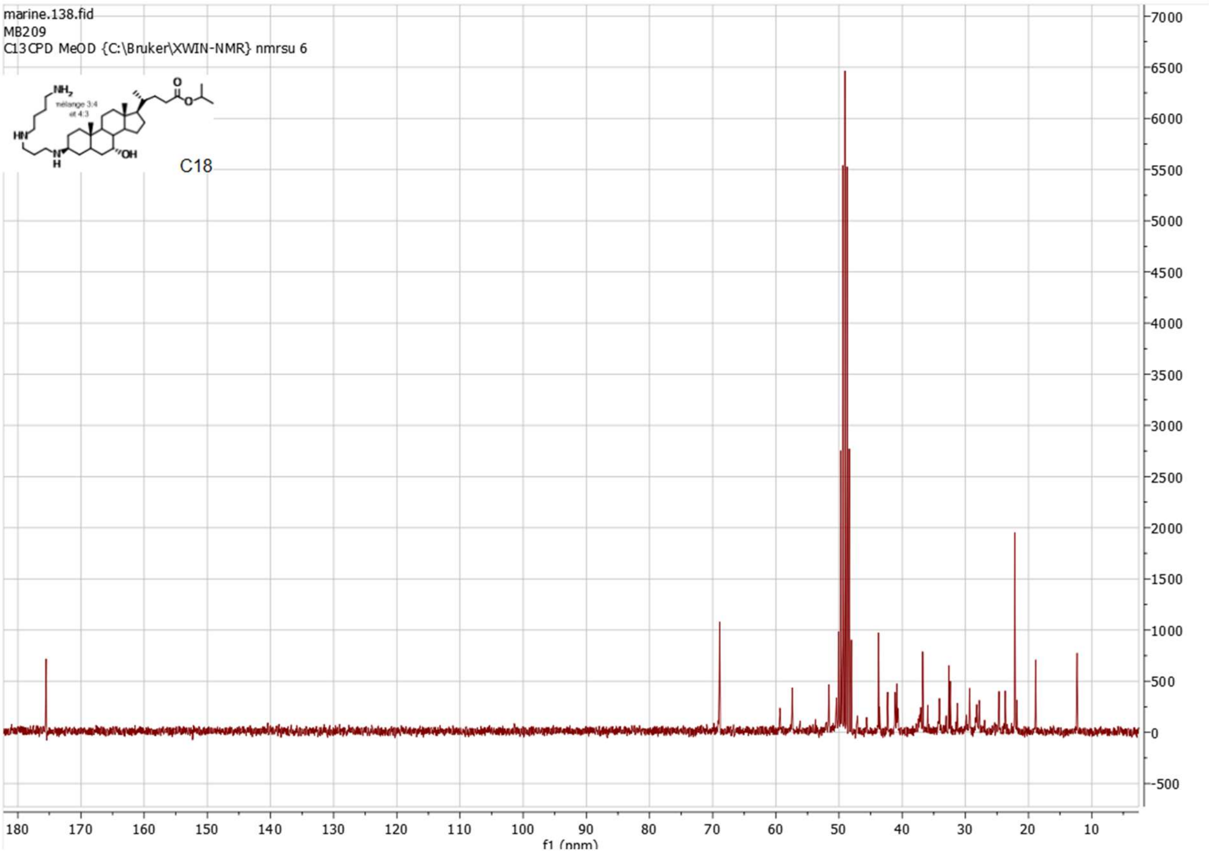
marine.138.fid
MB209
C13CPD MeOD {C:\Bruker\XWIN-NMR} nmrsu 6
mélange 3:4 et 4:3
C18

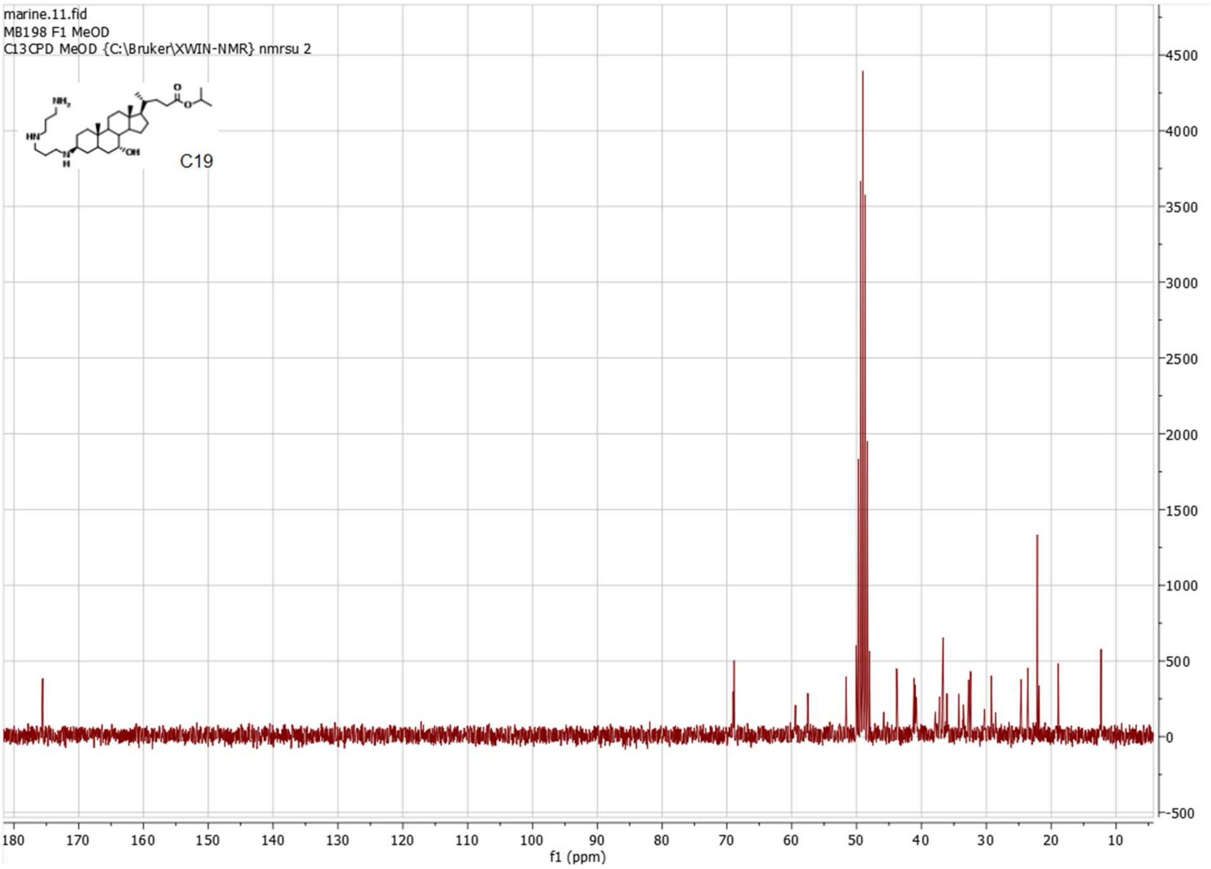
marine.11.fid
MB198 F1 MeOD
C13CPD MeOD {C:\Bruker\XWIN-NMR} nmrsu 2
C19
f1 (ppm)

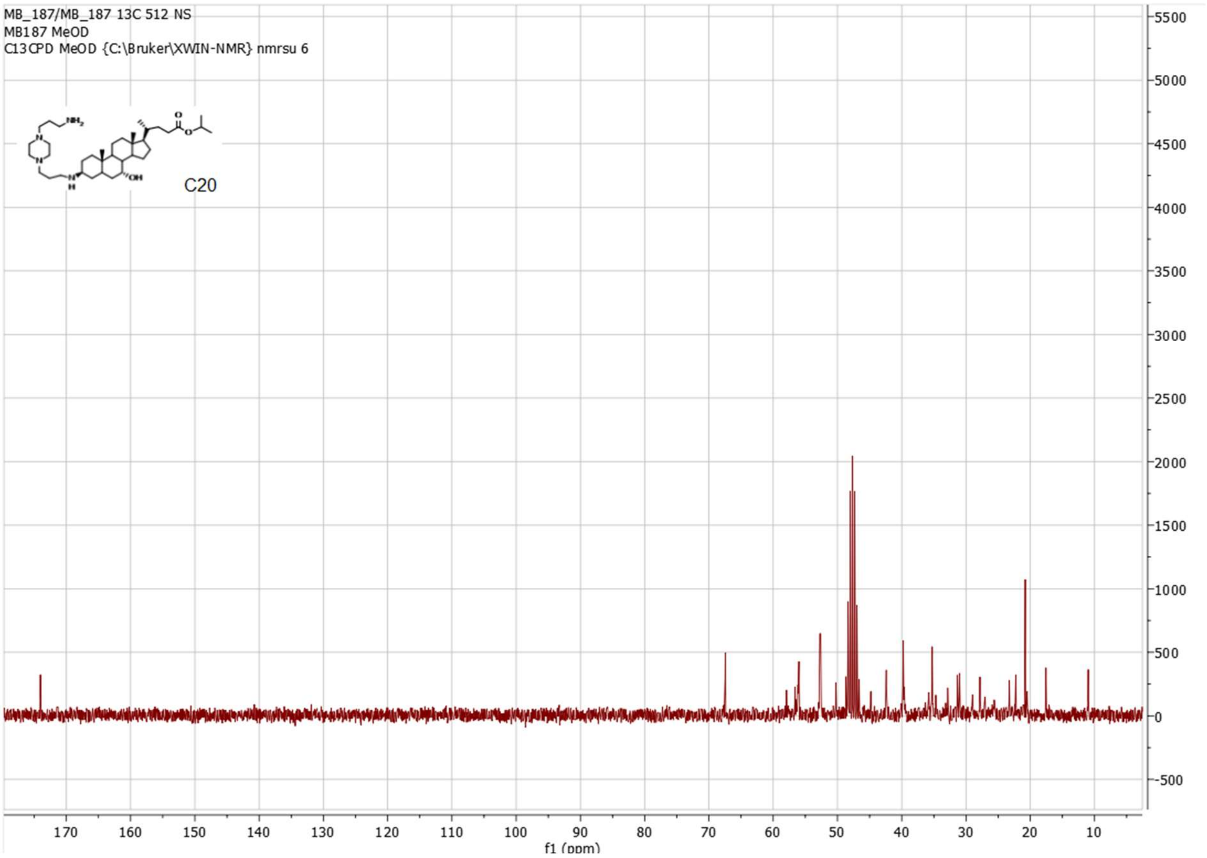
MB_187/MB_187 13C 512 NS
MB187 MeOD
C13CPD MeOD {C:\Bruker\XWIN-NMR} nmrsu 6
C20
f1 (ppm)

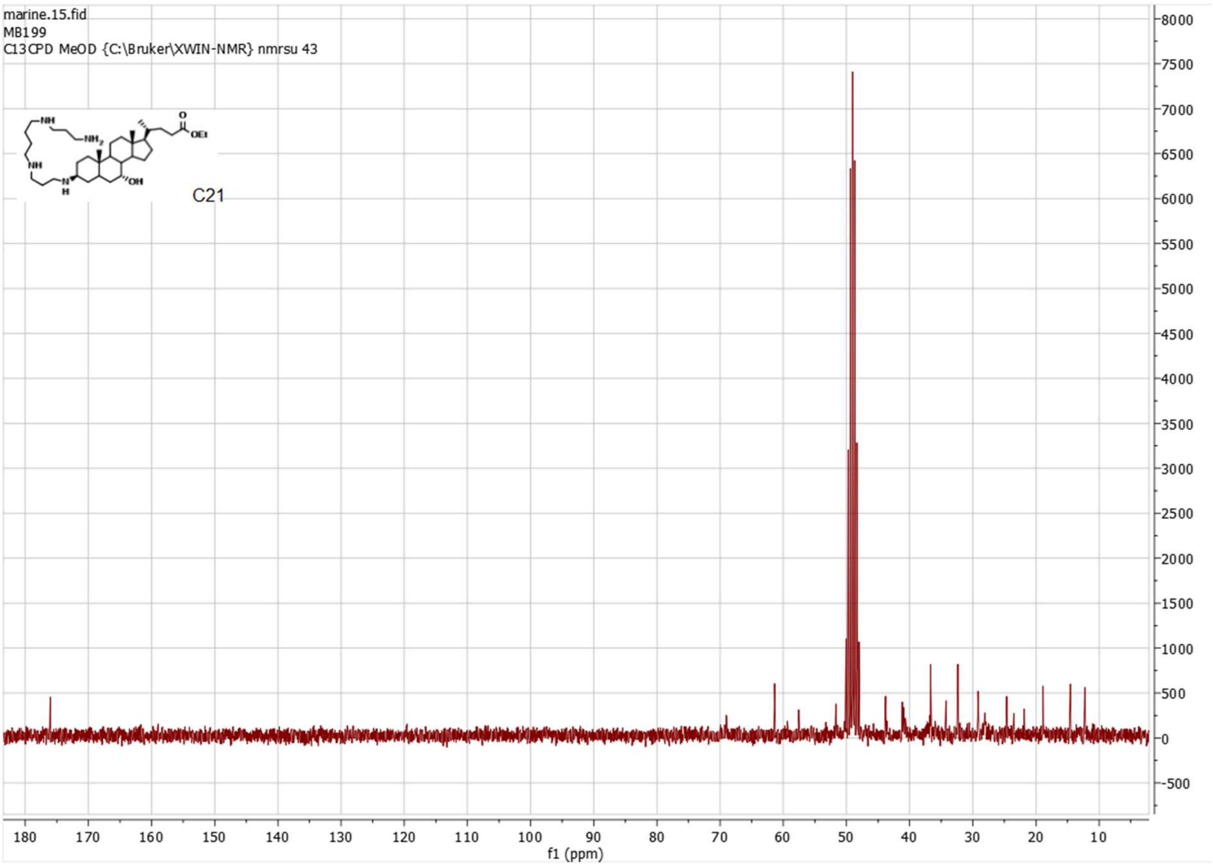
marine.15.fid
MB199
C13CPD MeOD {C:\Bruker\XWIN-NMR} nmrsu 43
C21
f1 (ppm)

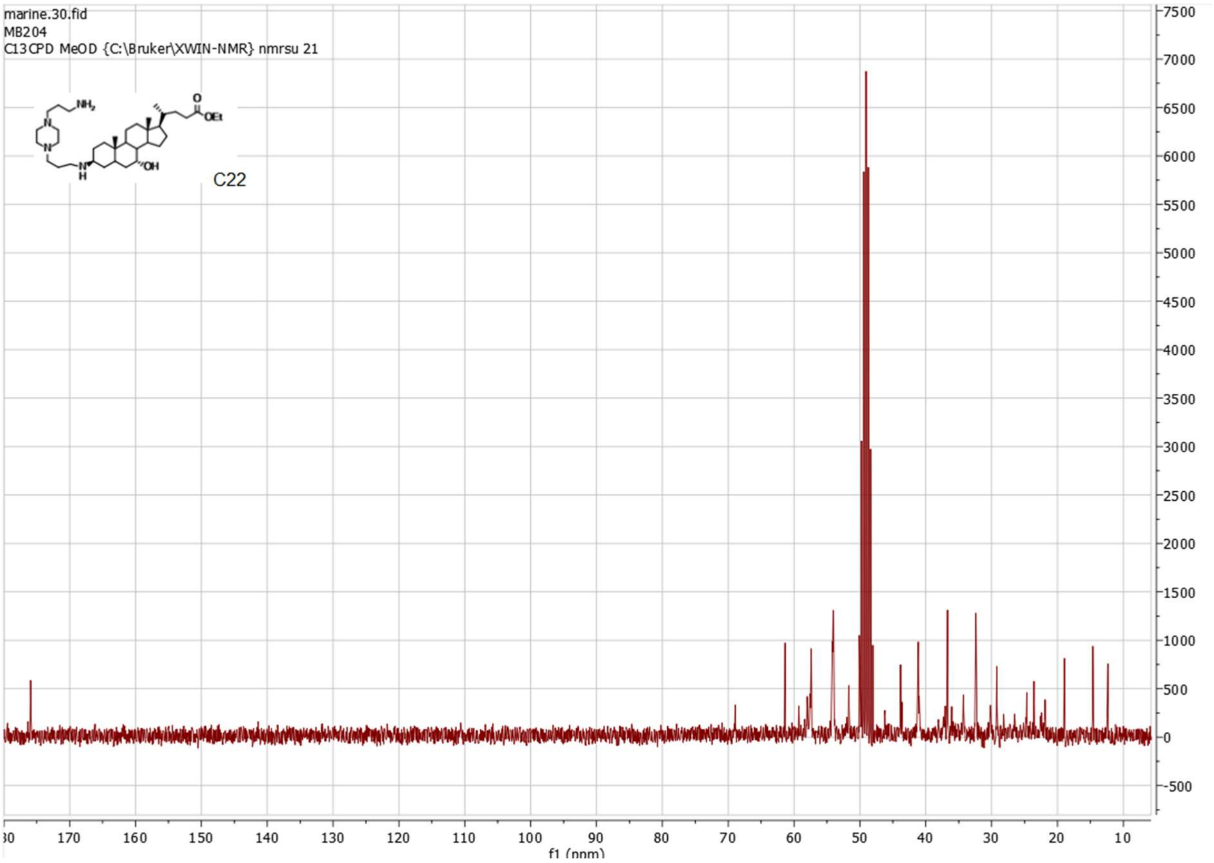
marine.30.fid
MB204
C13CPD MeOD {C:\Bruker\XWIN-NMR} nmrsu 21
C22
f1 (ppm)

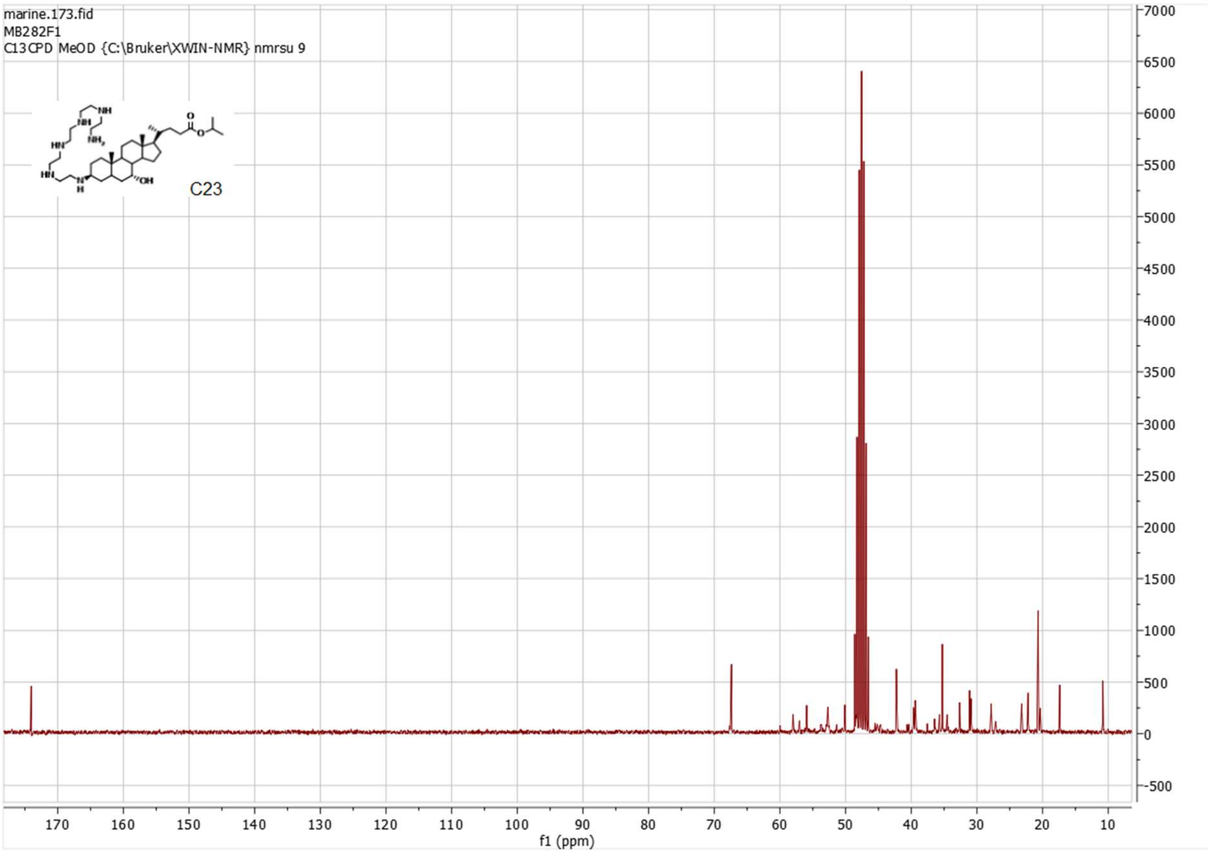
marine.173.fid
MB282F1
C13CPD MeOD {C:\Bruker\XWIN-NMR} nmrsu 9
C23
f1 (ppm)

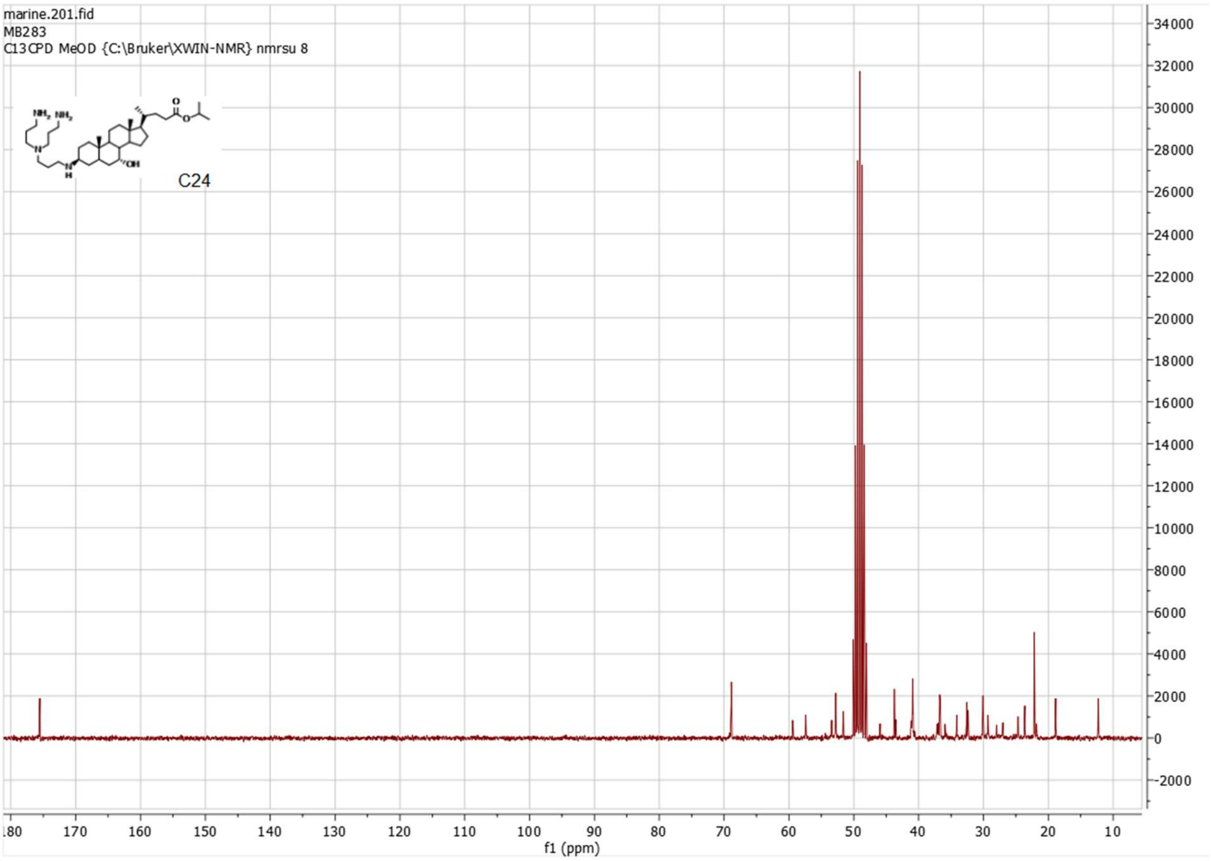
marine.201.fid
MB283
C13CPD MeOD {C:\Bruker\XWIN-NMR} nmrsu 8
C24
f1 (ppm)

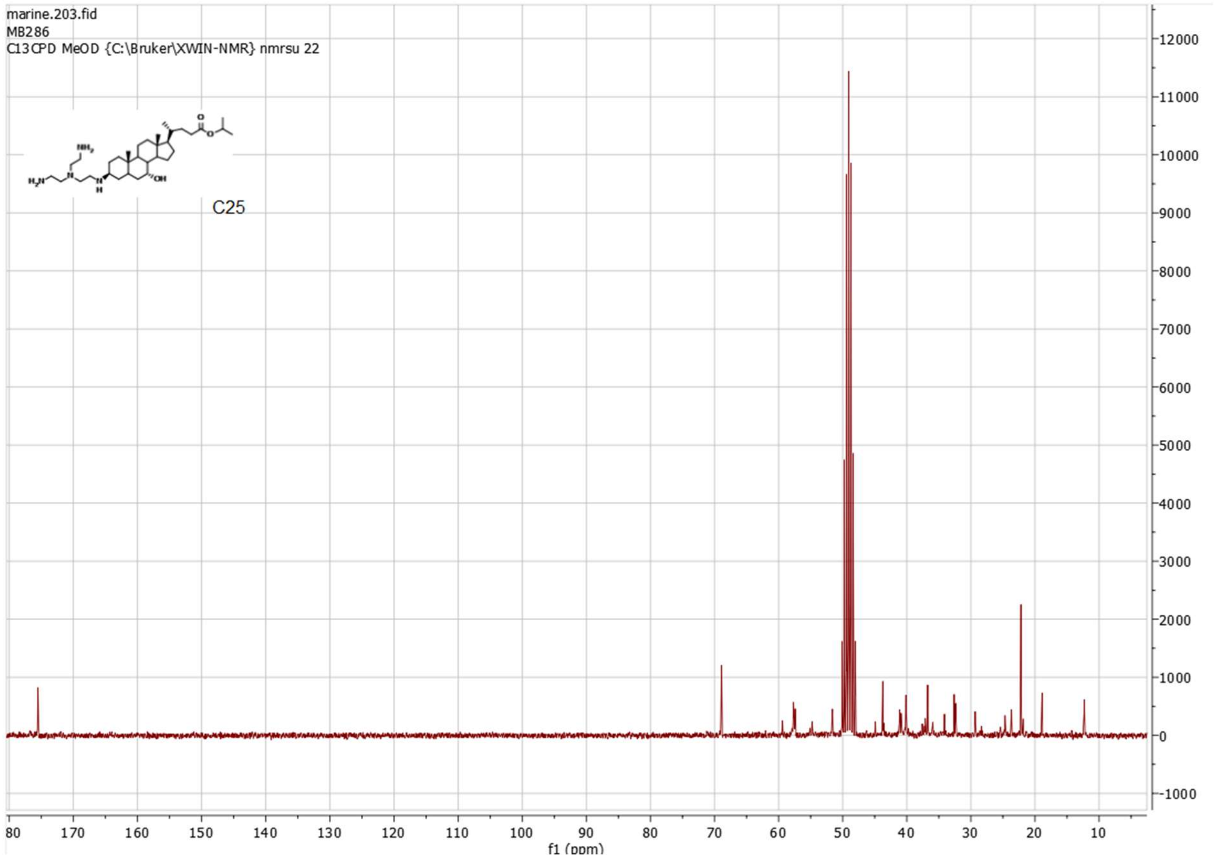
marine.203.fid
MB286
C13CPD MeOD {C:\Bruker\XWIN-NMR} nmrsu 22
C25
f1 (ppm)

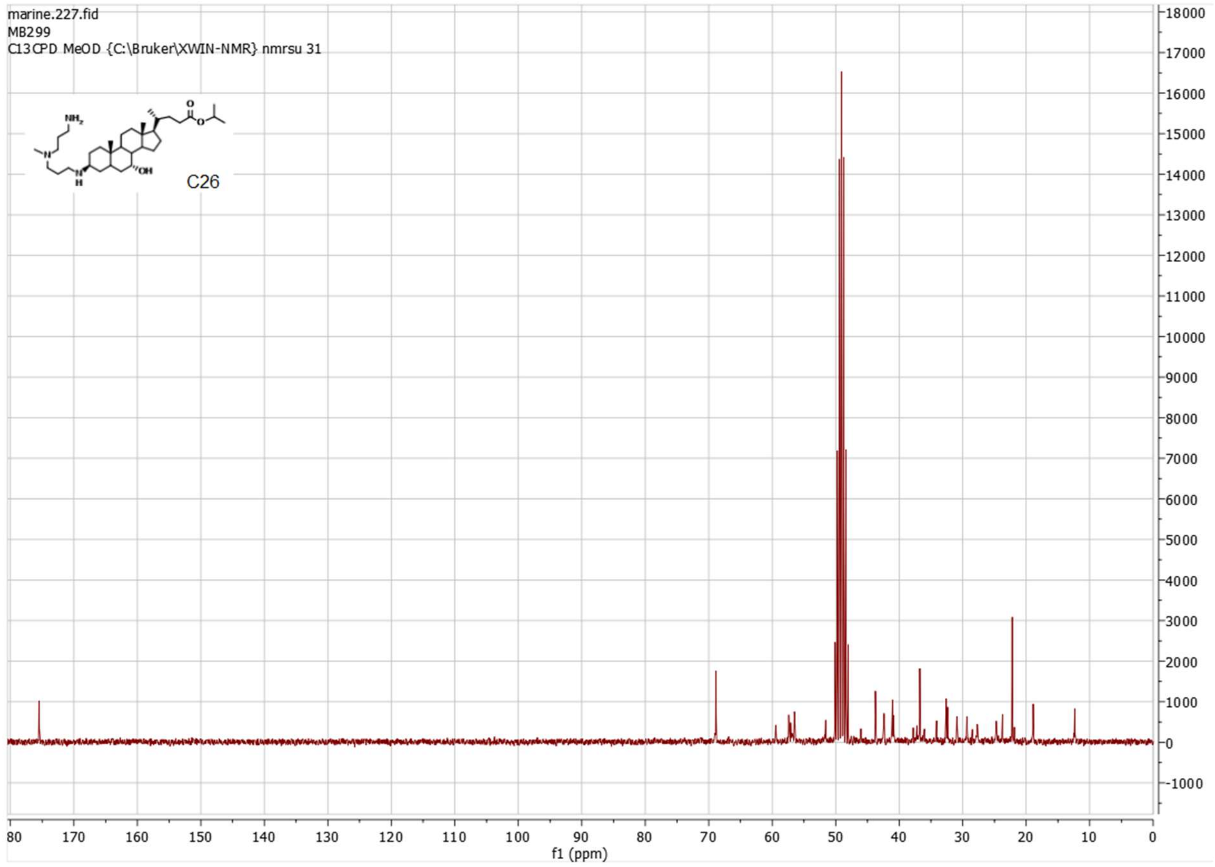
marine.227.fid
MB299
C13CPD MeOD {C:\Bruker\XWIN-NMR} nmrsu 31
C26
f1 (ppm)

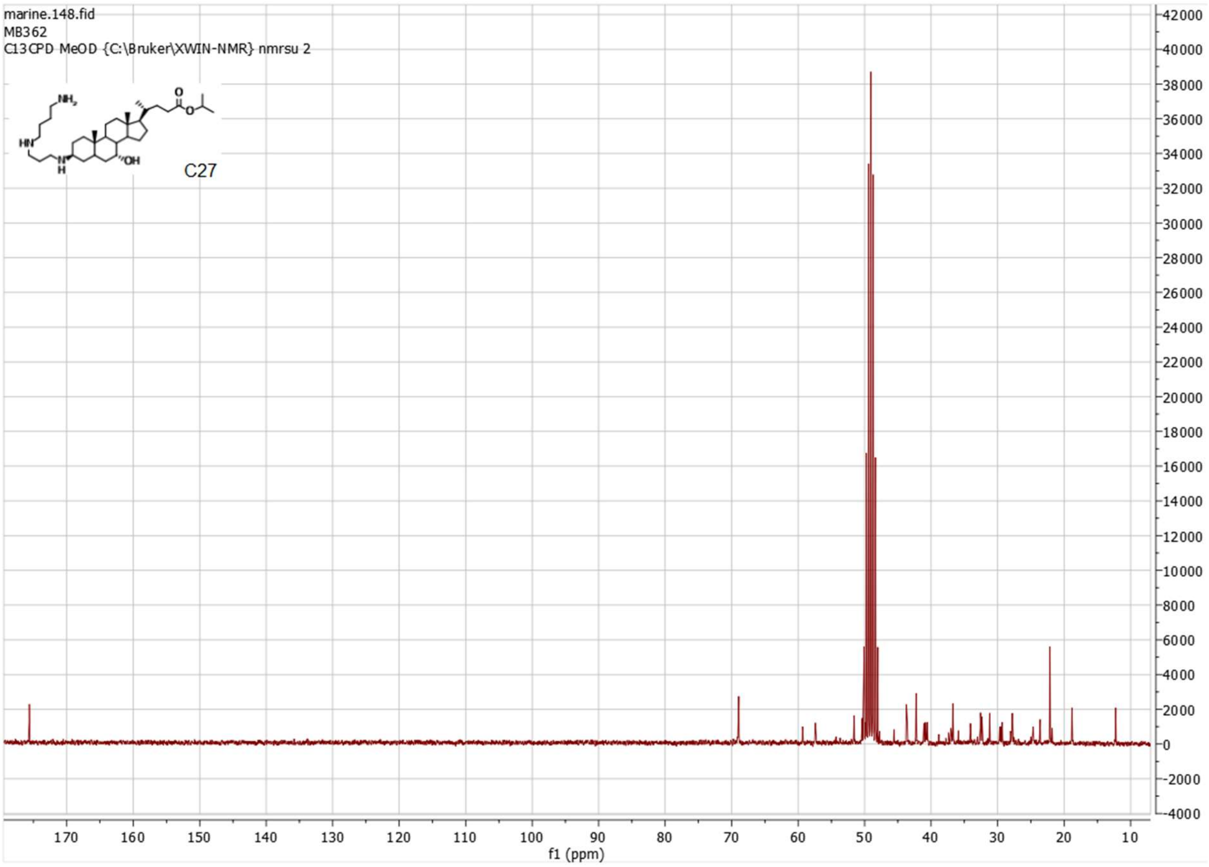
marine.148.fid
MB362
C13CPD MeOD {C:\Bruker\XWIN-NMR} nmrsu 2
C27
f1 (ppm)

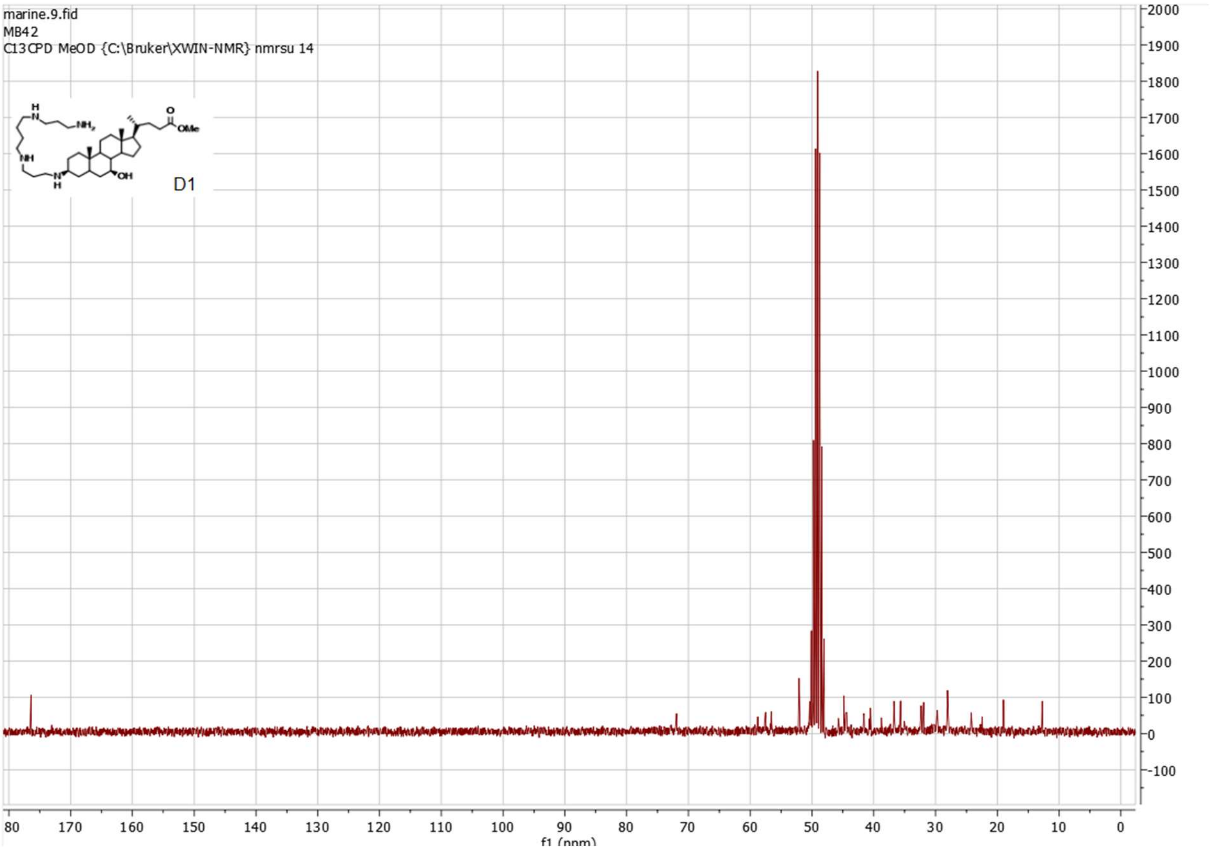
marine.9.fid
MB42
C13CPD MeOD {C:\Bruker\XWIN-NMR} nmrsu 14
D1
f1 (ppm)

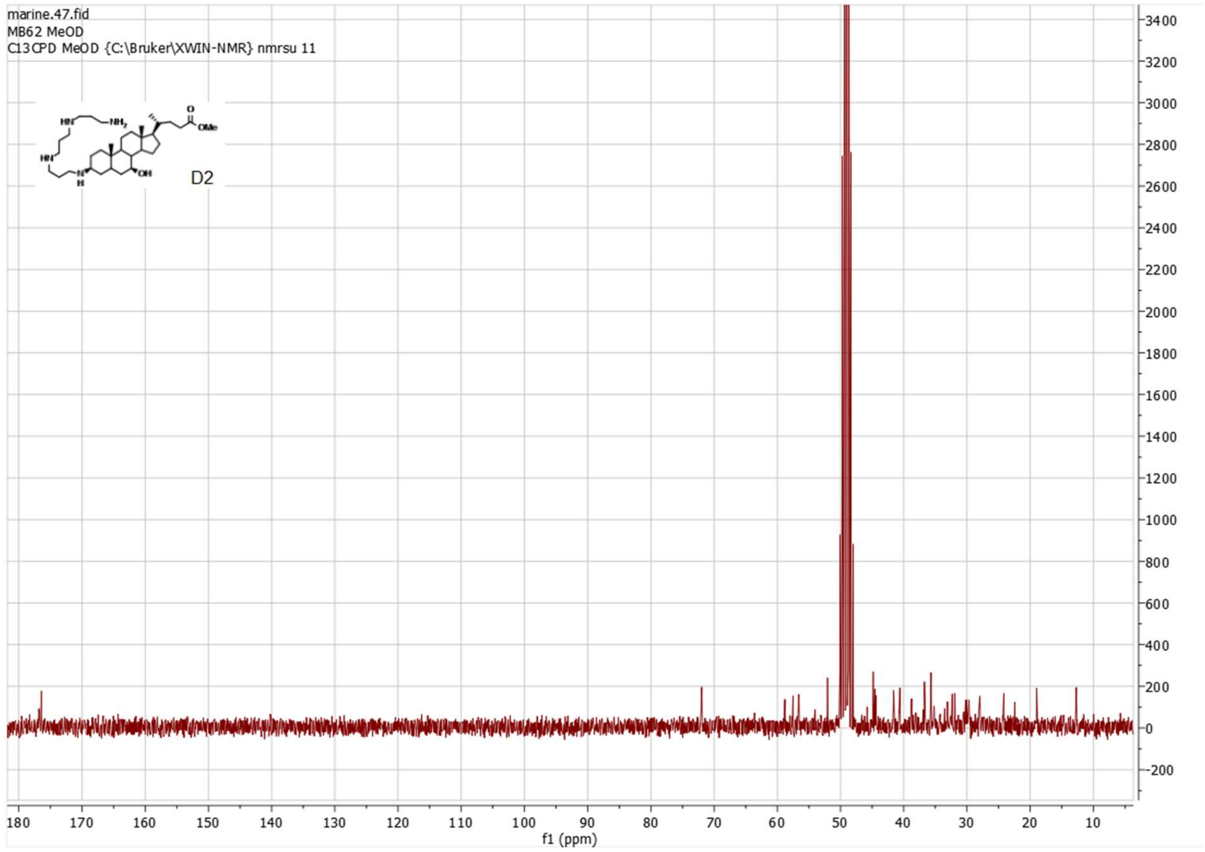
marine.47.fid
MB62 MeOD
C13CPD MeOD {C:\Bruker\XWIN-NMR} nmrsu 11
D2
f1 (ppm)

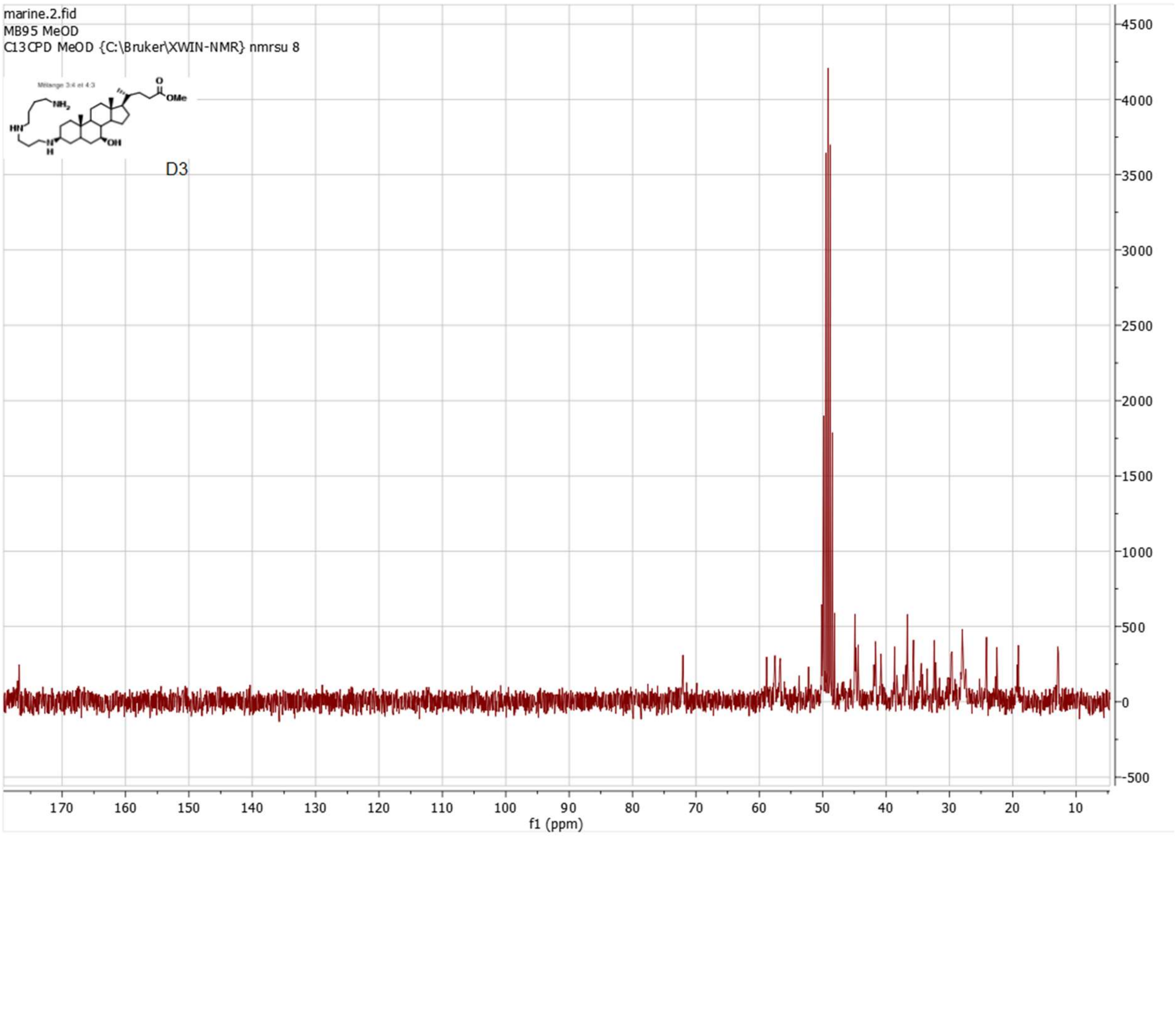
marine.2.fid
MB95 MeOD
C13CPD MeOD {C:\Bruker\XWIN-NMR} nmrsu 8
D3
f1 (ppm)

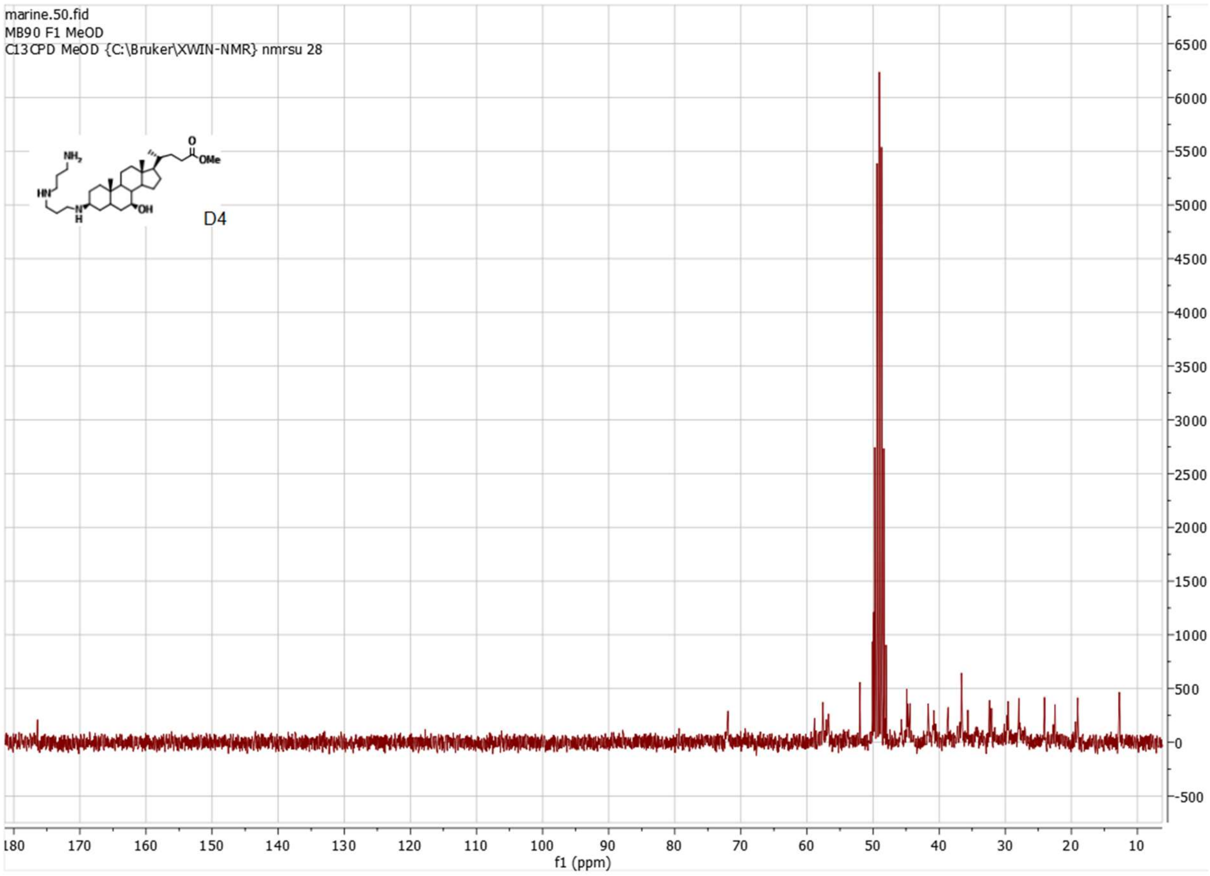
marine.50.fid
MB90 F1 MeOD
C13CPD MeOD {C:\Bruker\XWIN-NMR} nmrsu 28
D4
f1 (ppm)

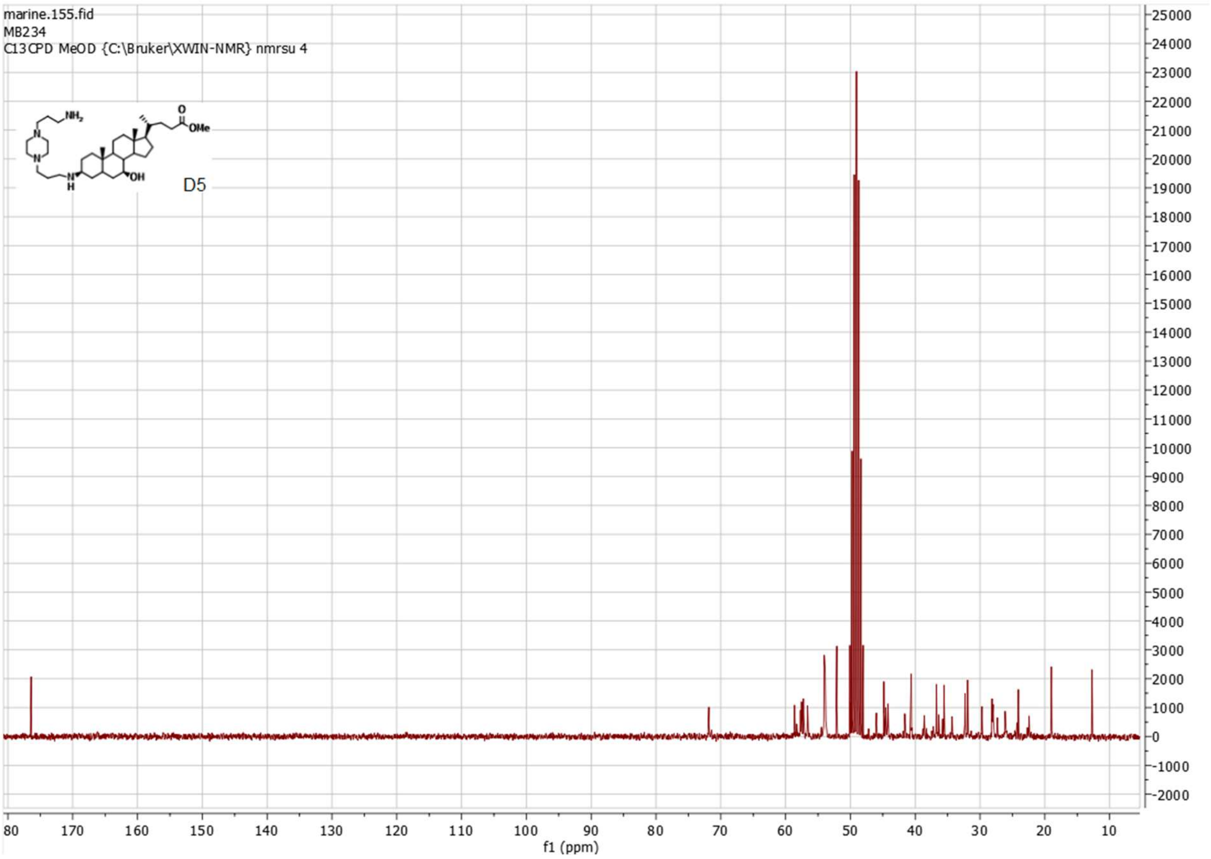
marine.155.fid
MB234
C13CPD MeOD {C:\Bruker\XWIN-NMR} nmrsu 4
D5
f1 (ppm)

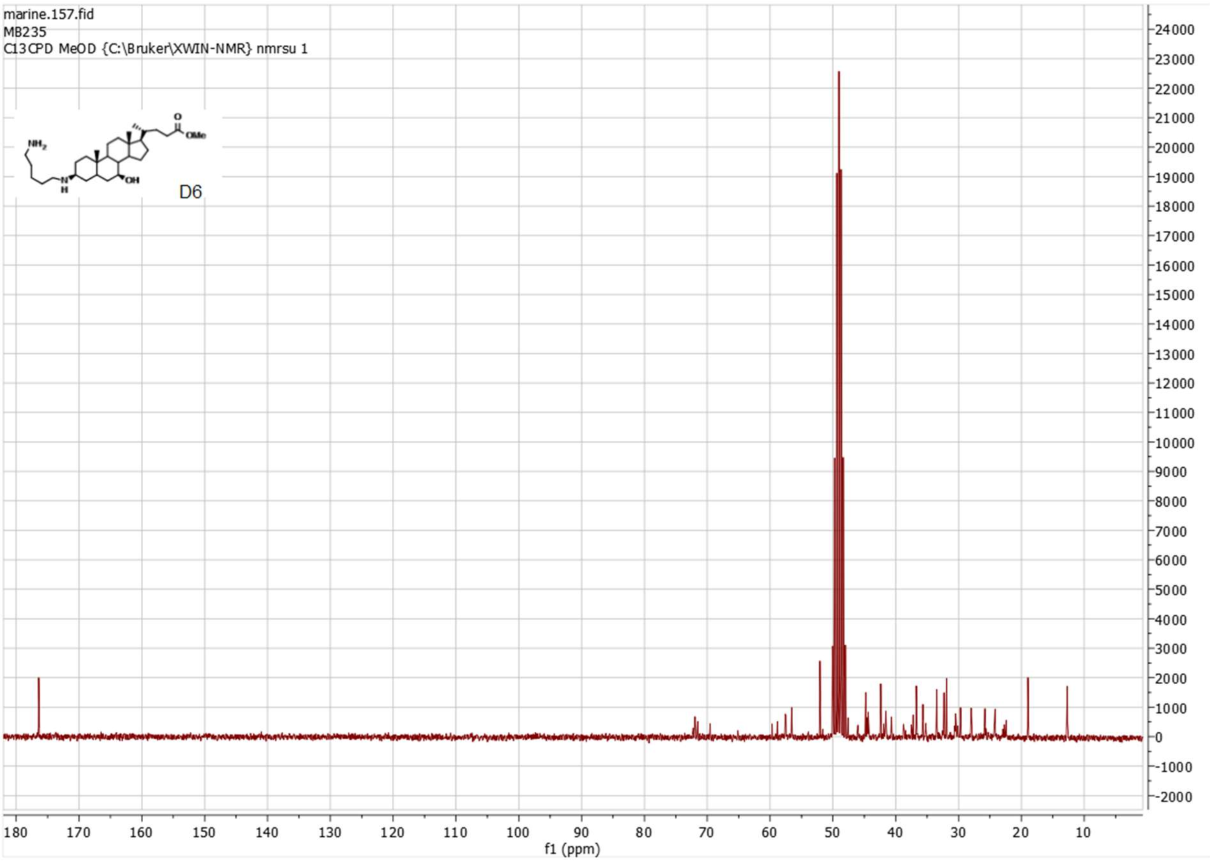
marine.157.fid
MB235
C13CPD MeOD {C:\Bruker\XWIN-NMR} nmrsu 1
D6
f1 (ppm)

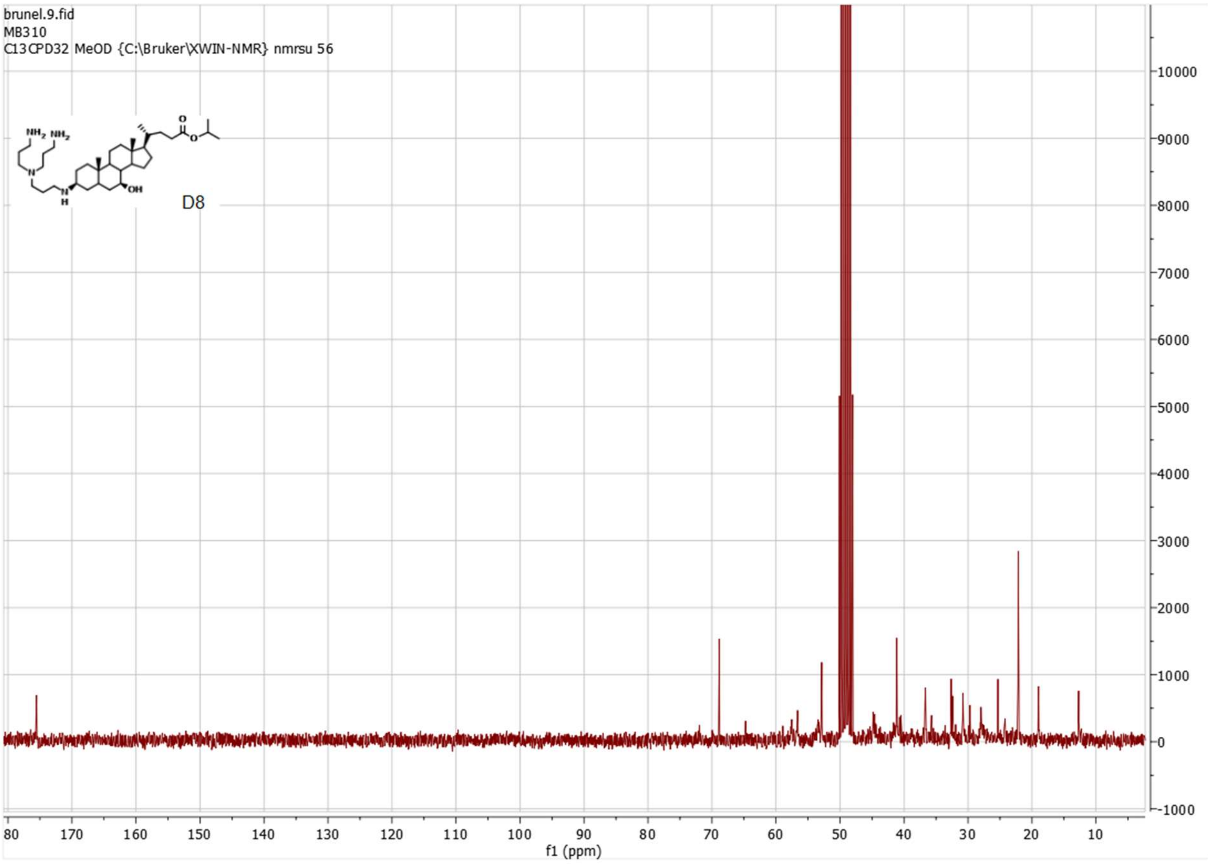
brunel.9.fid
MB310
C13CPD32 MeOD {C:\Bruker\XWIN-NMR} nmrsu 56
D8
f1 (ppm)

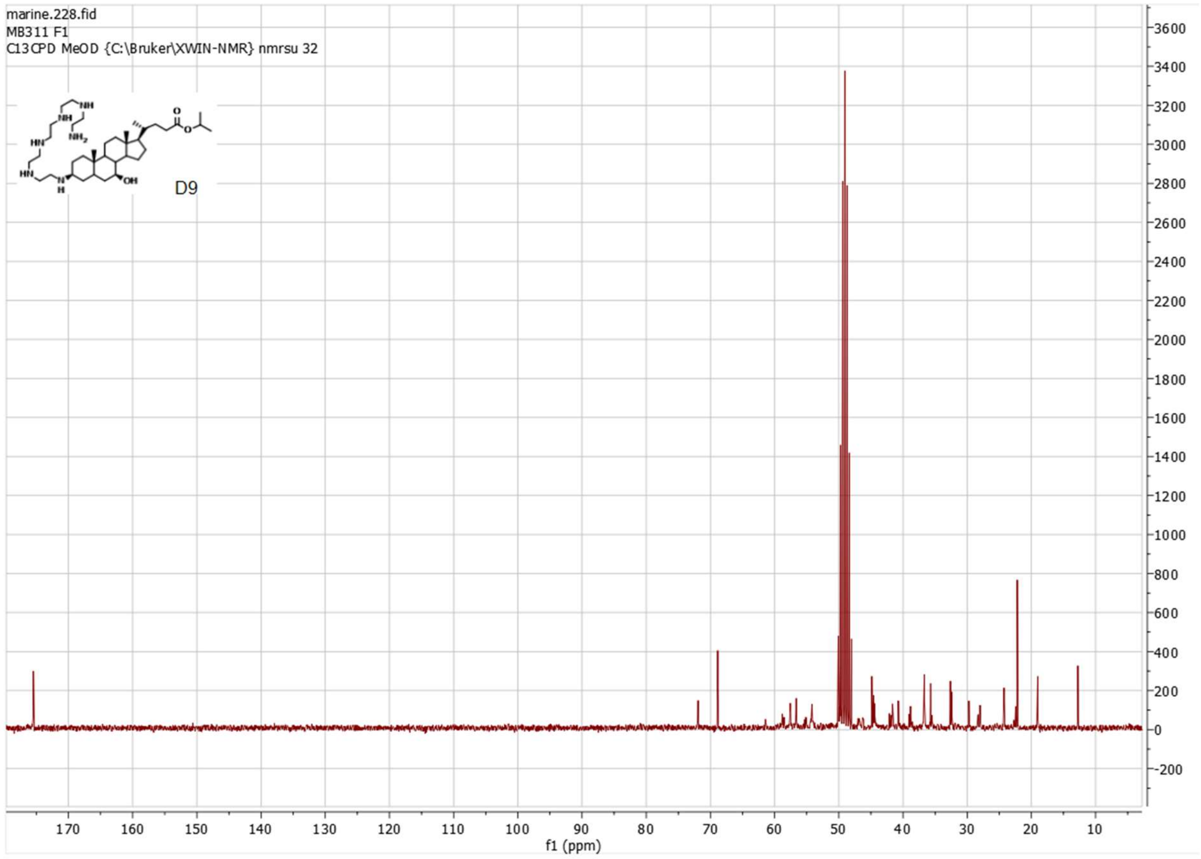
marine.228.fid
MB311 F1
C13CPD MeOD {C:\Bruker\XWIN-NMR} nmrsu 32
D9
f1 (ppm)

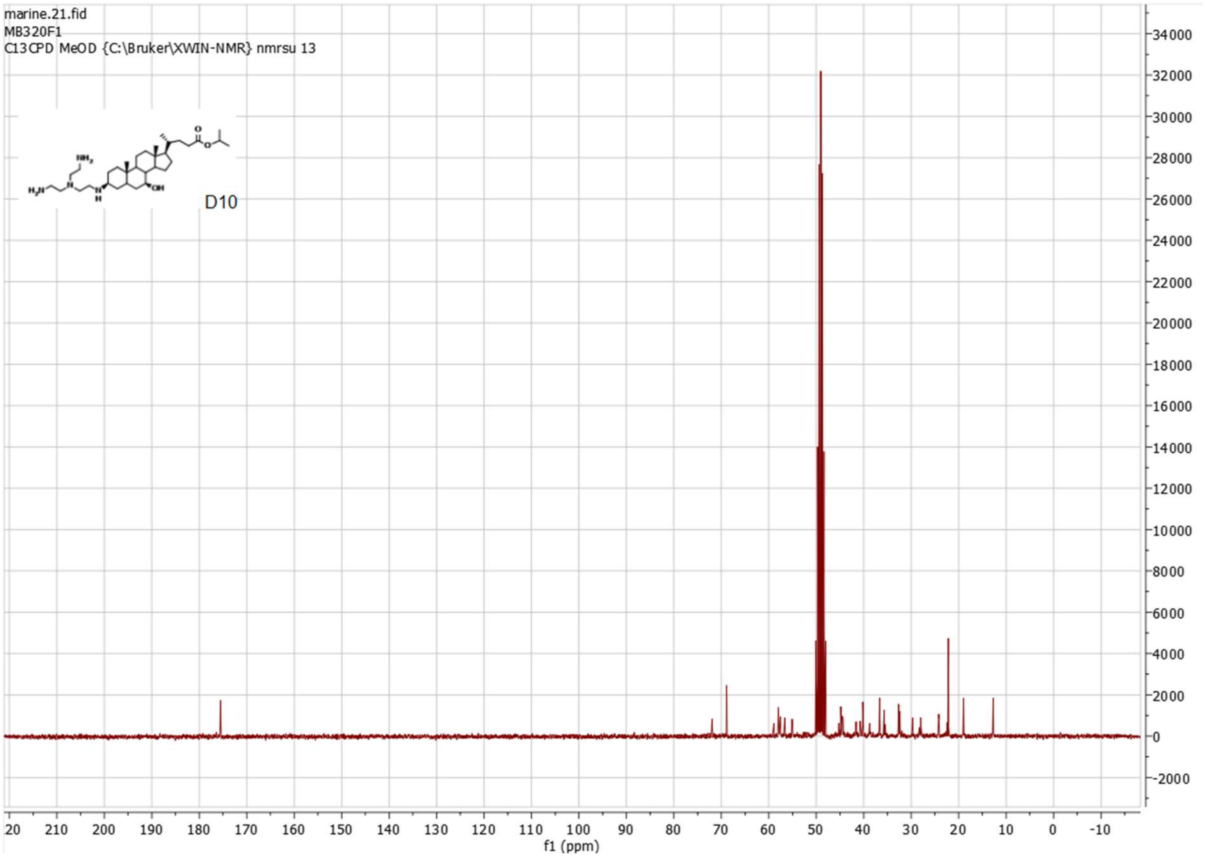
marine.21.fid
MB320F1
C13CPD MeOD {C:\Bruker\XWIN-NMR} nmrsu 13
D10
f1 (ppm)

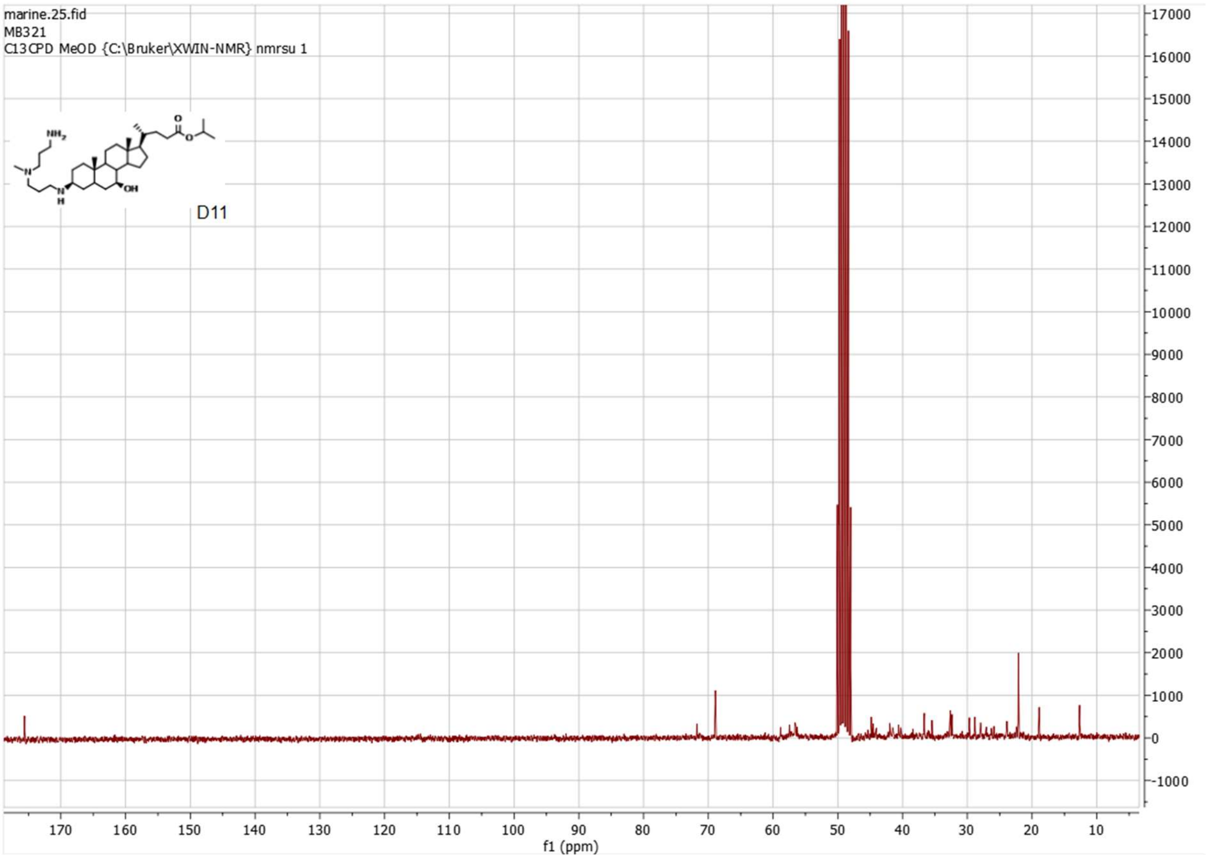

marine.25.fid
MB321
C13CPD MeOD {C:\Bruker\XWIN-NMR} nmrsu 1
NH2
N
N
H
O
O
OH
D11
f1 (ppm)


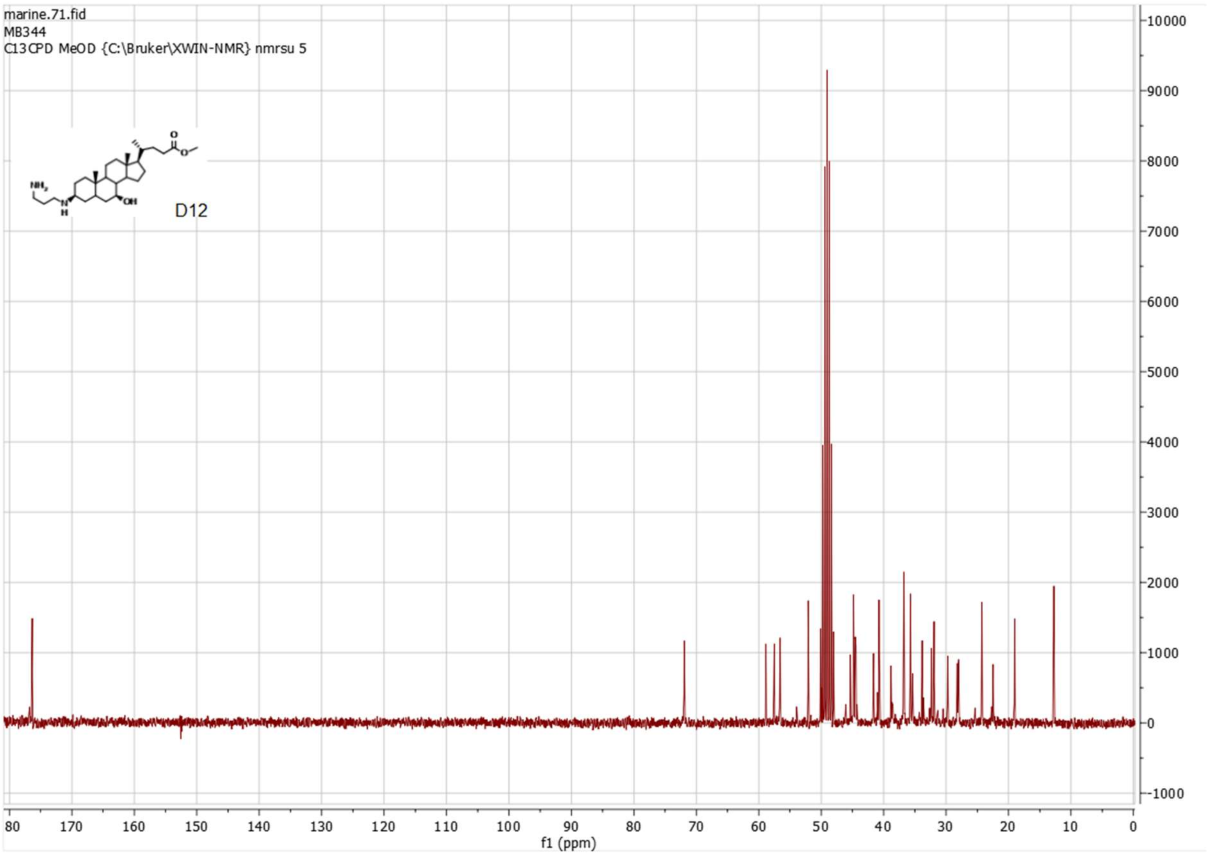

marine.71.fid
MB344
C13CPD MeOD {C:\Bruker\XWIN-NMR} nmrsu 5
NH2
N
H
O
O
OH
D12
f1 (ppm)

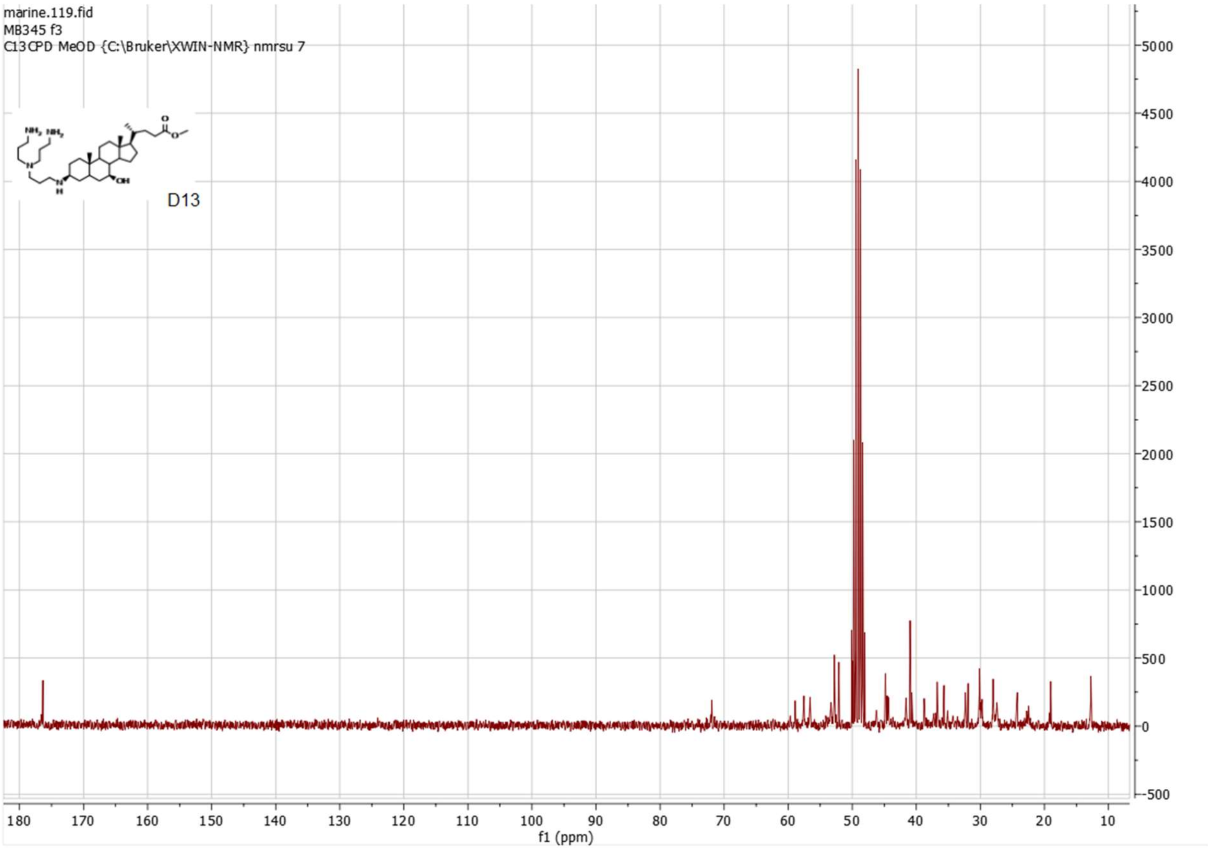
marine.119.fid
MB345 f3
C13CPD MeOD {C:\Bruker\XWIN-NMR} nmrsu 7
D13
f1 (ppm)

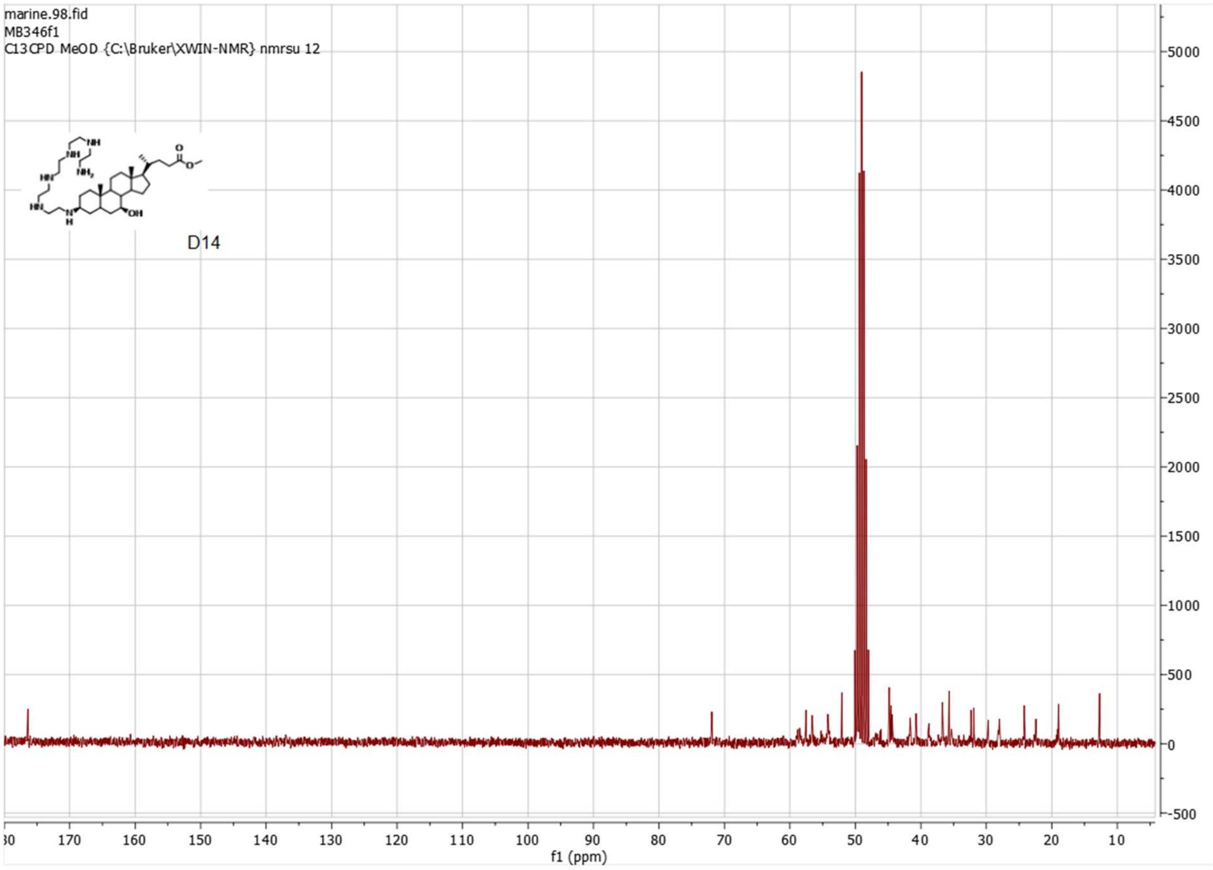
marine.98.fid
MB346f1
C13CPD MeOD {C:\Bruker\XWIN-NMR} nmrsu 12
D14
f1 (ppm)

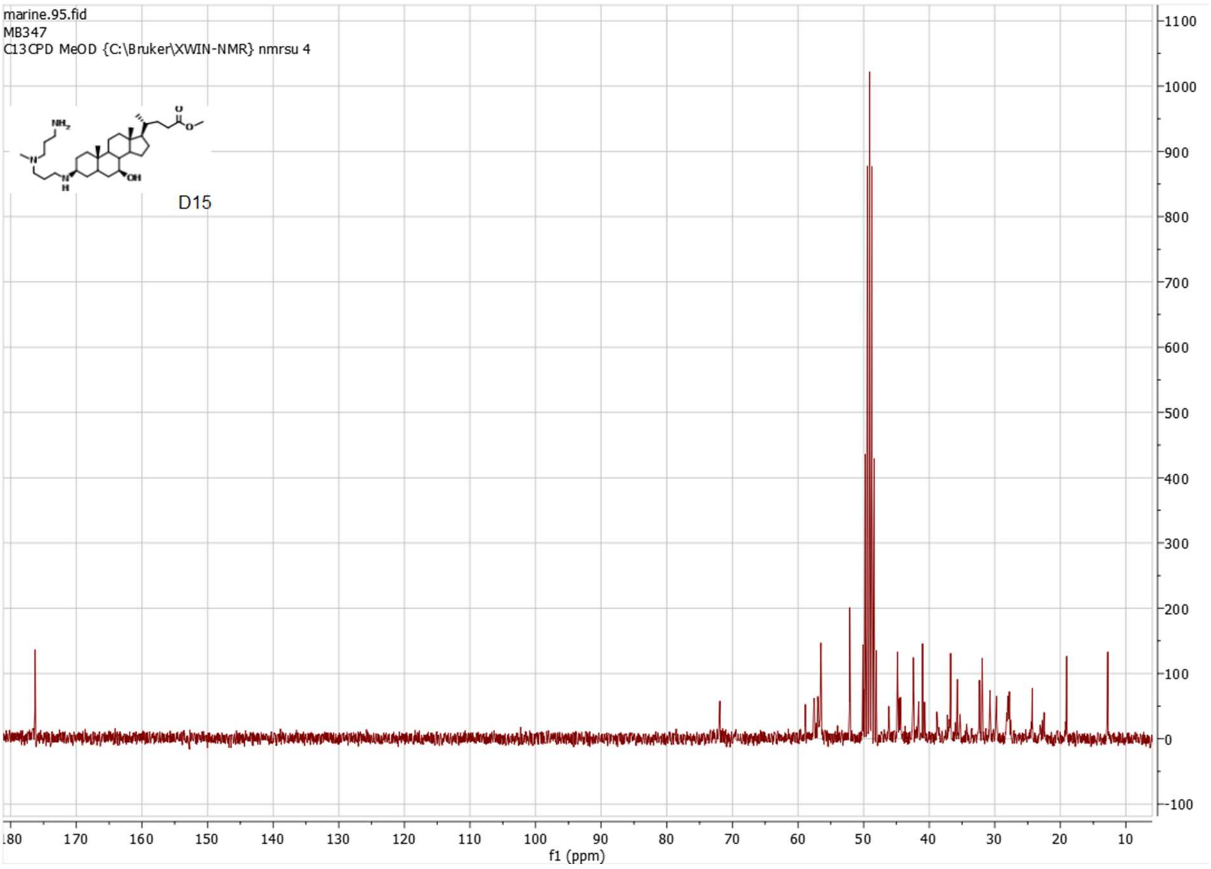
marine.95.fid
MB347
C13CPD MeOD {C:\Bruker\XWIN-NMR} nmrsu 4
D15
f1 (ppm)

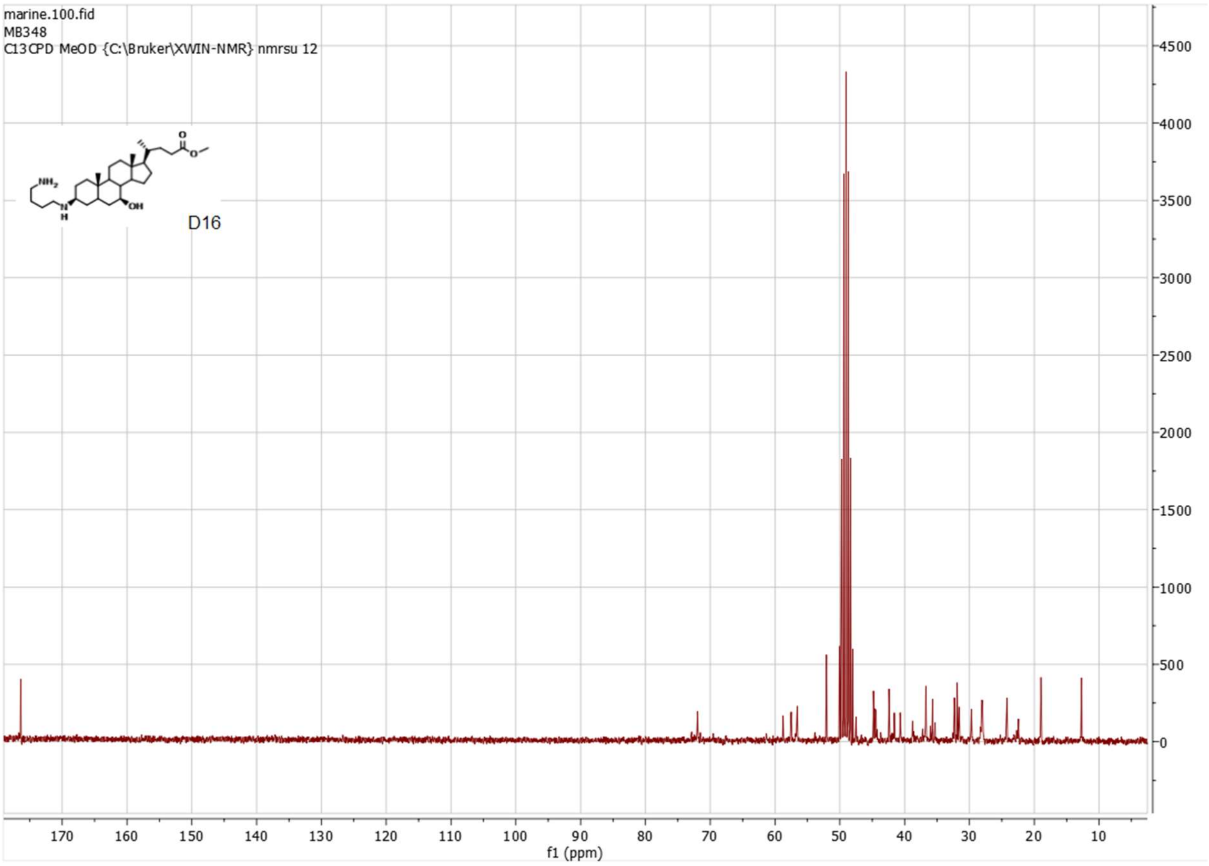
marine.100.fid
MB348
C13CPD MeOD {C:\Bruker\XWIN-NMR} nmrsu 12
D16
f1 (ppm)

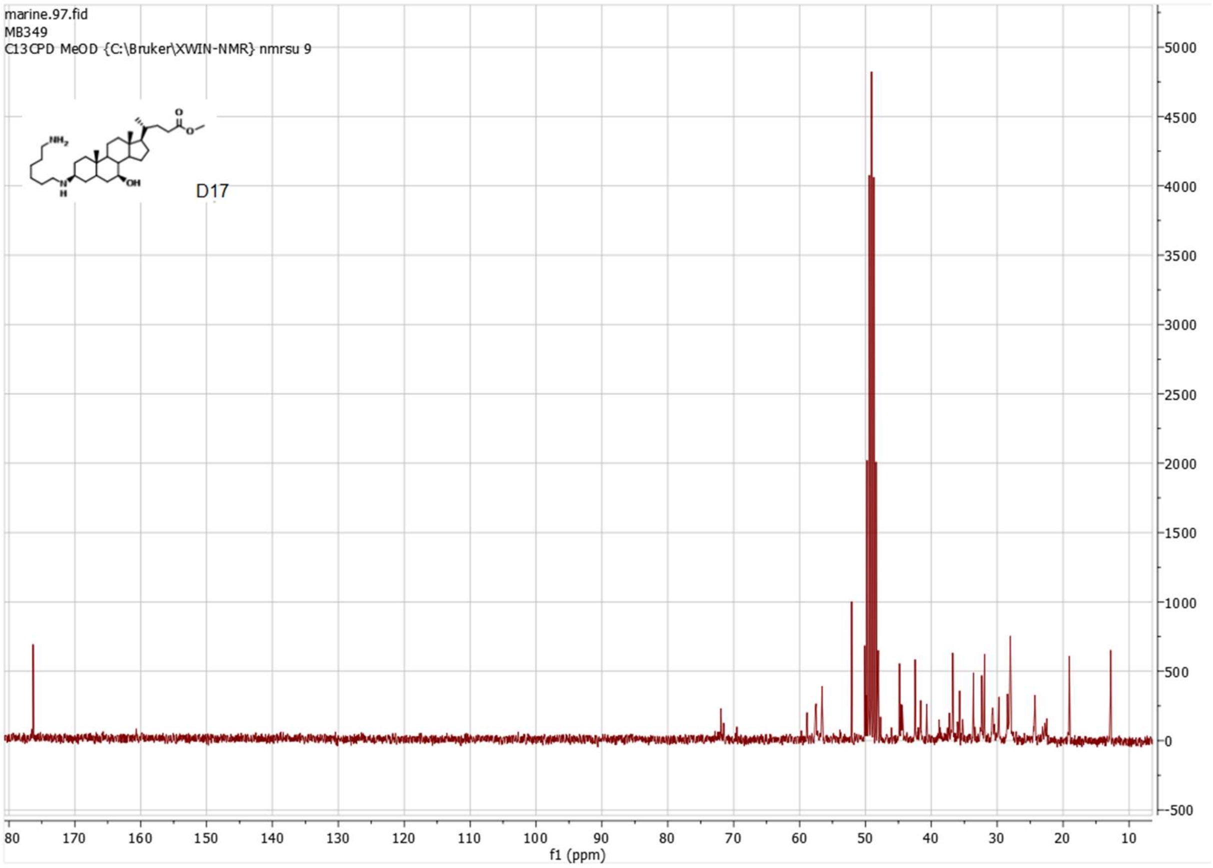
marine.97.fid
MB349
C13CPD MeOD {C:\Bruker\XWIN-NMR} nmrsu 9
D17
f1 (ppm)

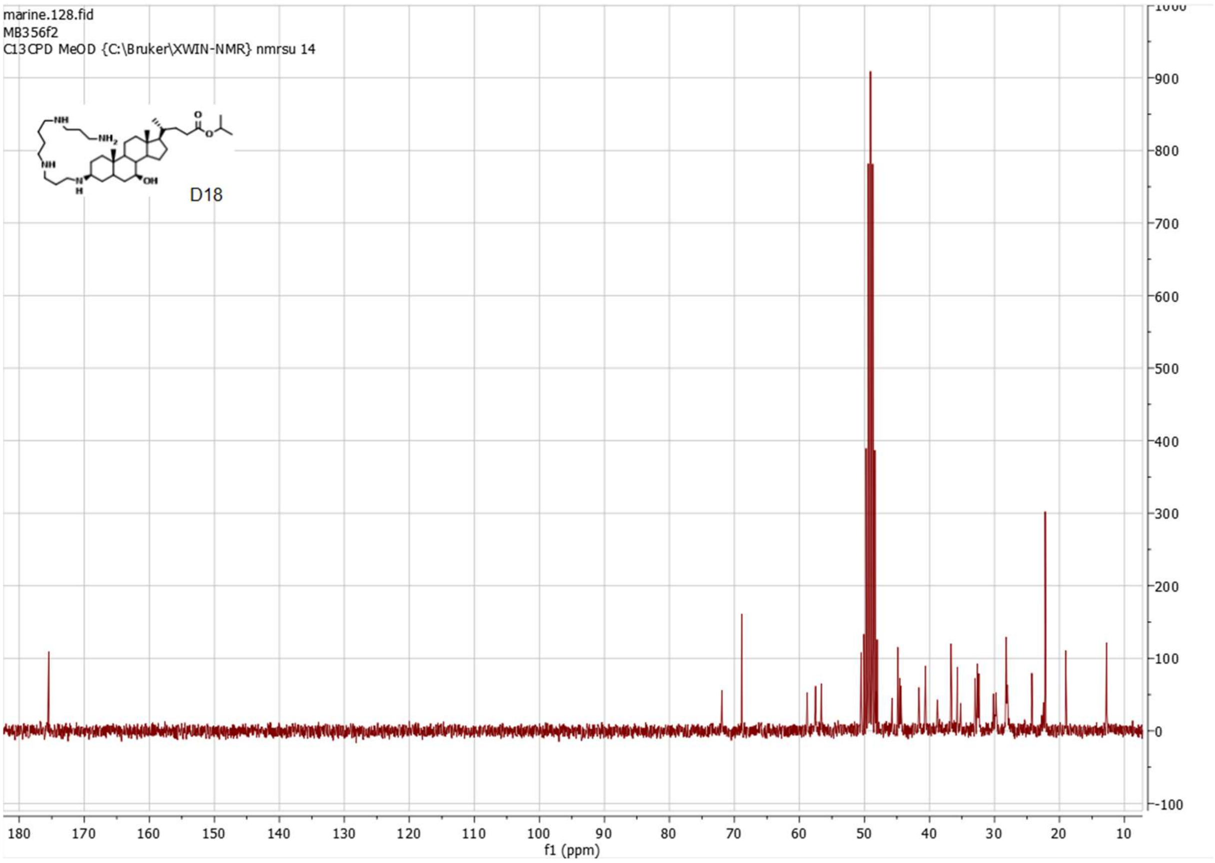
marine.128.fid
MB356f2
C13CPD MeOD {C:\Bruker\XWIN-NMR} nmrsu 14
D18
f1 (ppm)

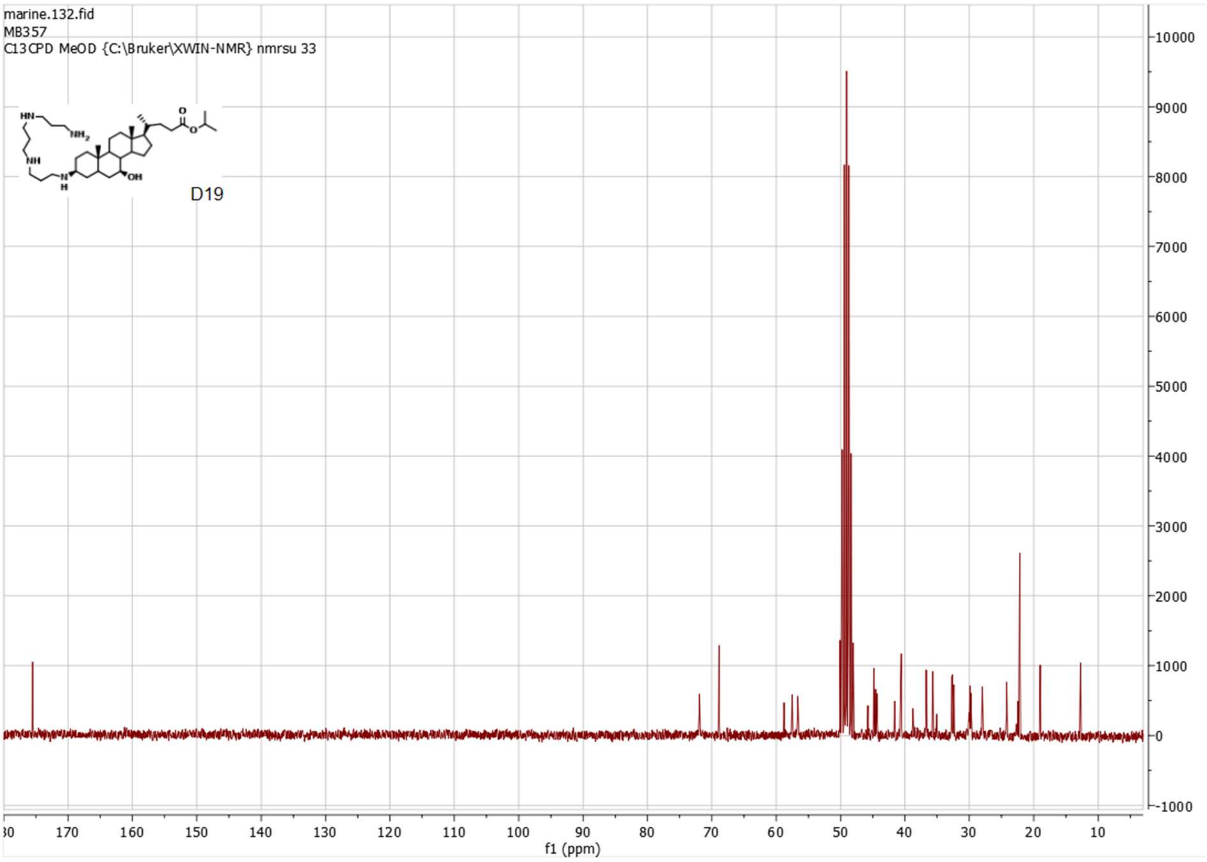

marine.132.fid
MB357
C13CPD MeOD {C:\Bruker\XWIN-NMR} nmrsu 33
D19
f1 (ppm)


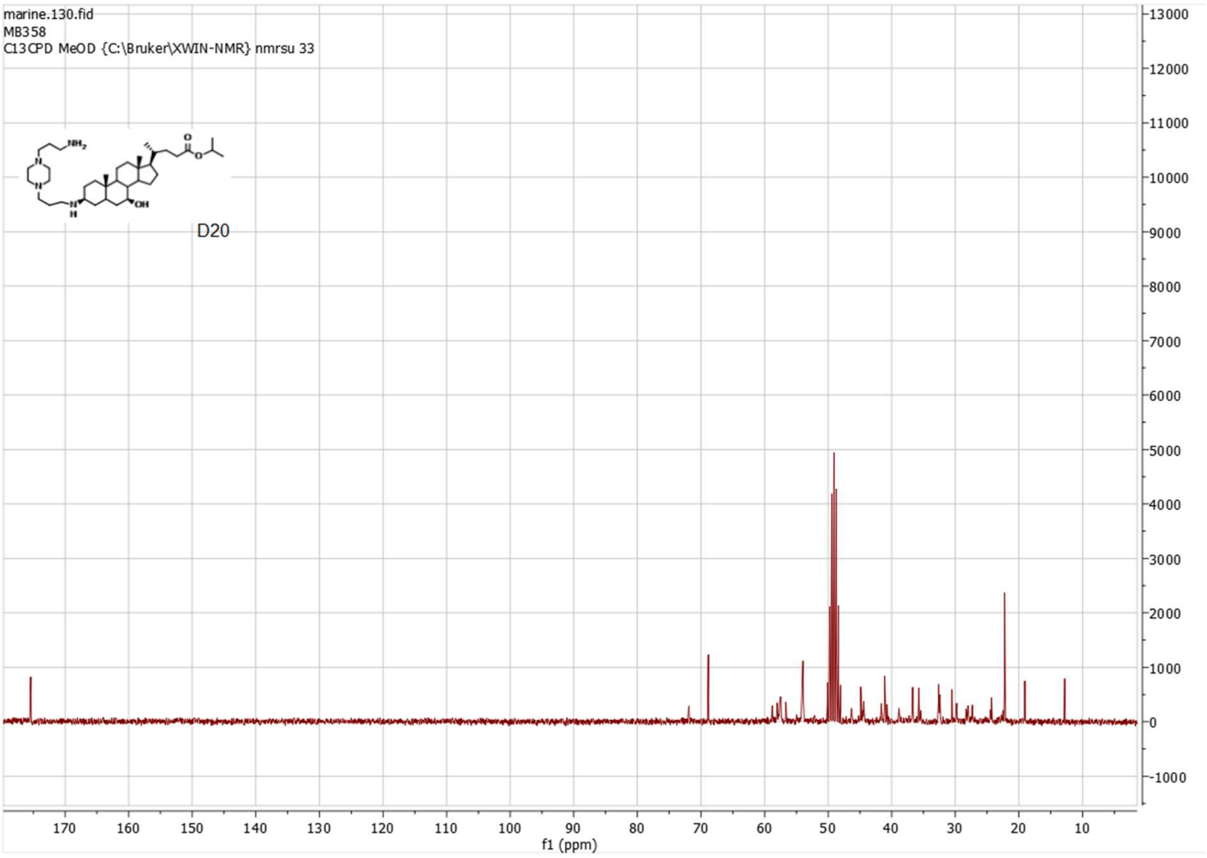

marine.130.fid
MB358
C13CPD MeOD {C:\Bruker\XWIN-NMR} nmrsu 33
D20
f1 (ppm)

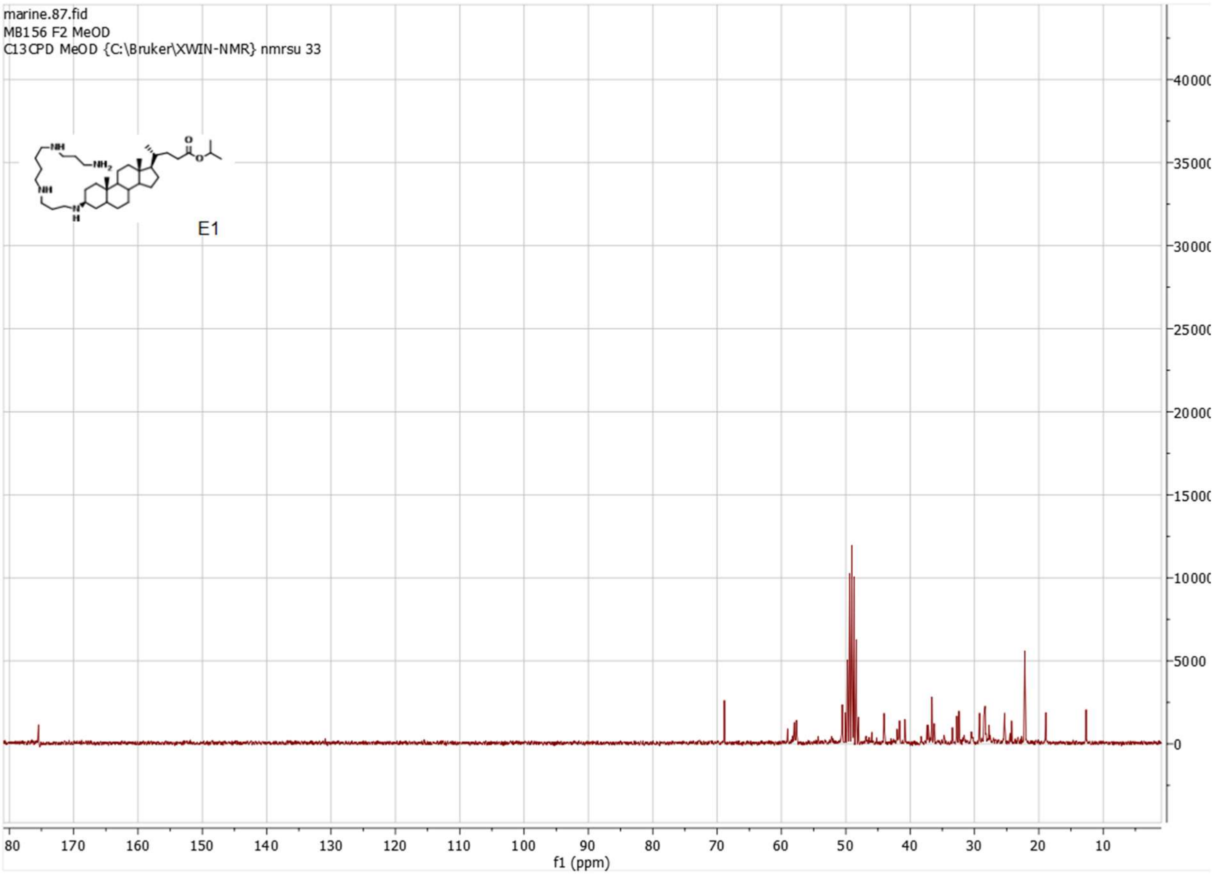
marine.87.fid
MB156 F2 MeOD
C13CPD MeOD {C:\Bruker\XWIN-NMR} nmrsu 33
E1
f1 (ppm)

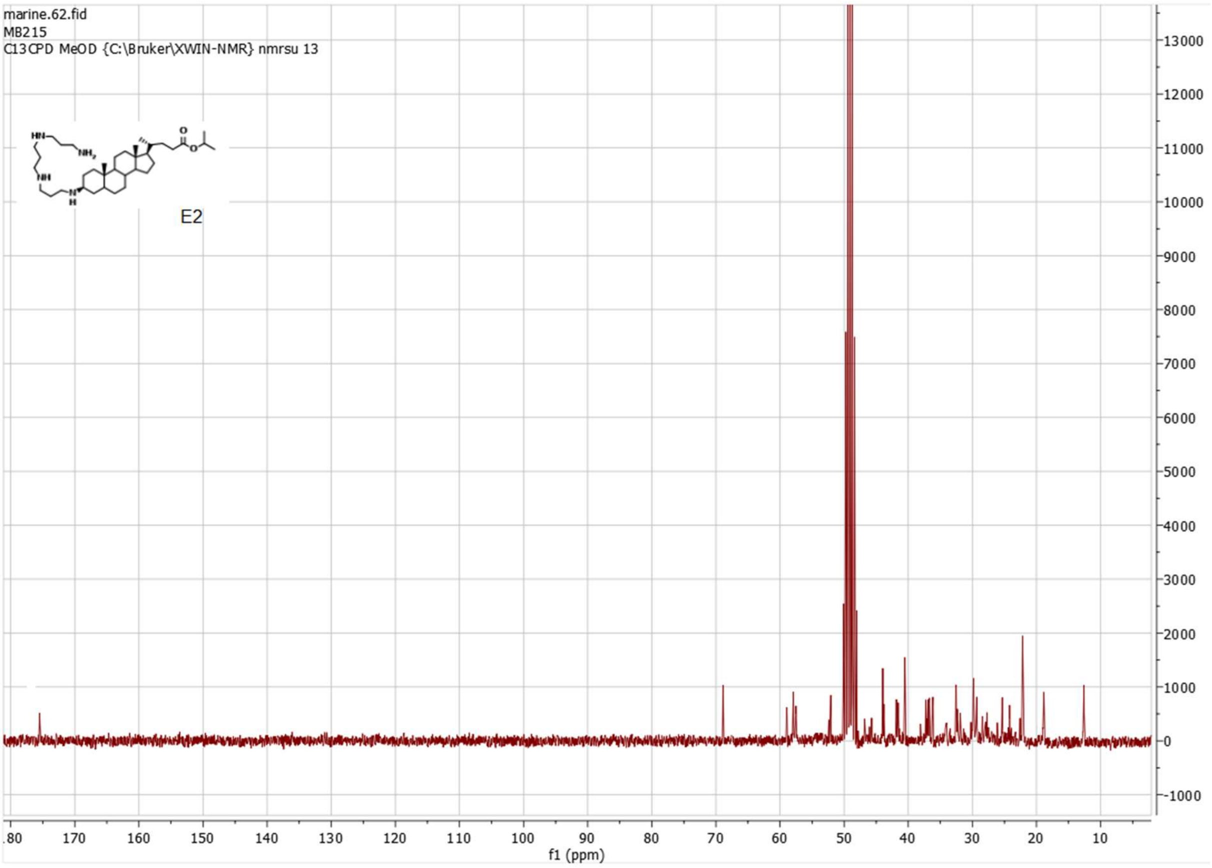
marine.62.fid
MB215
C13CPD MeOD {C:\Bruker\XWIN-NMR} nmrsu 13
E2
f1 (ppm)

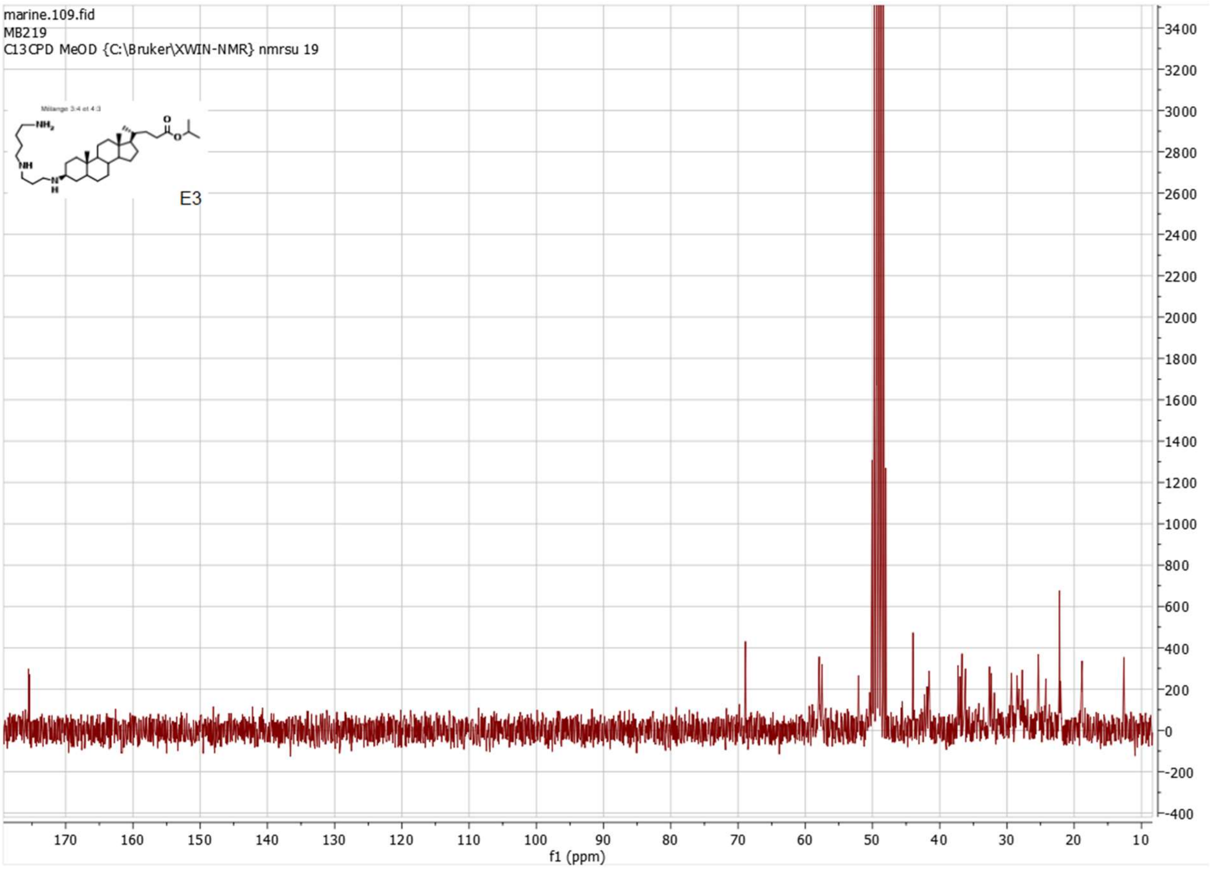
marine.109.fid
MB219
C13CPD MeOD {C:\Bruker\XWIN-NMR} nmrsu 19
E3
f1 (ppm)

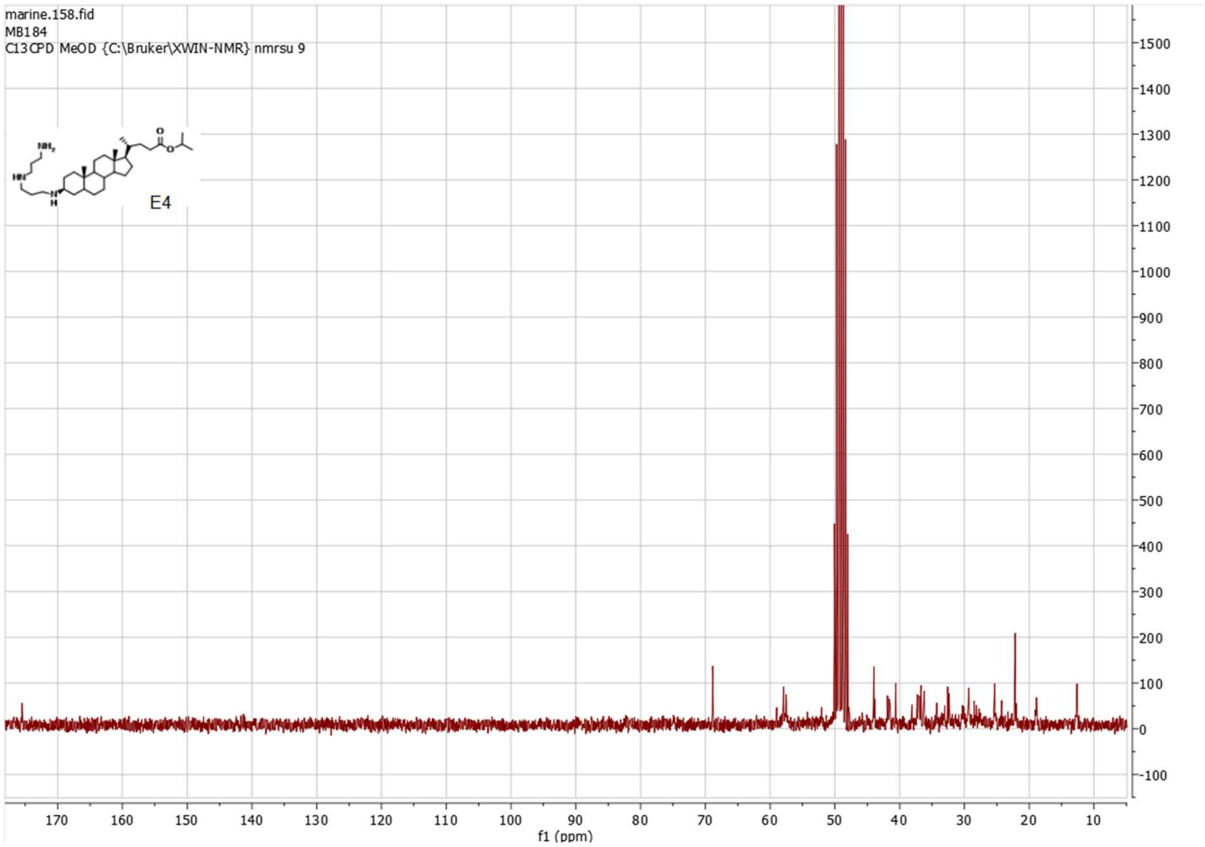
marine.158.fid
MB184
C13CPD MeOD {C:\Bruker\XWIN-NMR} nmrsu 9
E4
f1 (ppm)

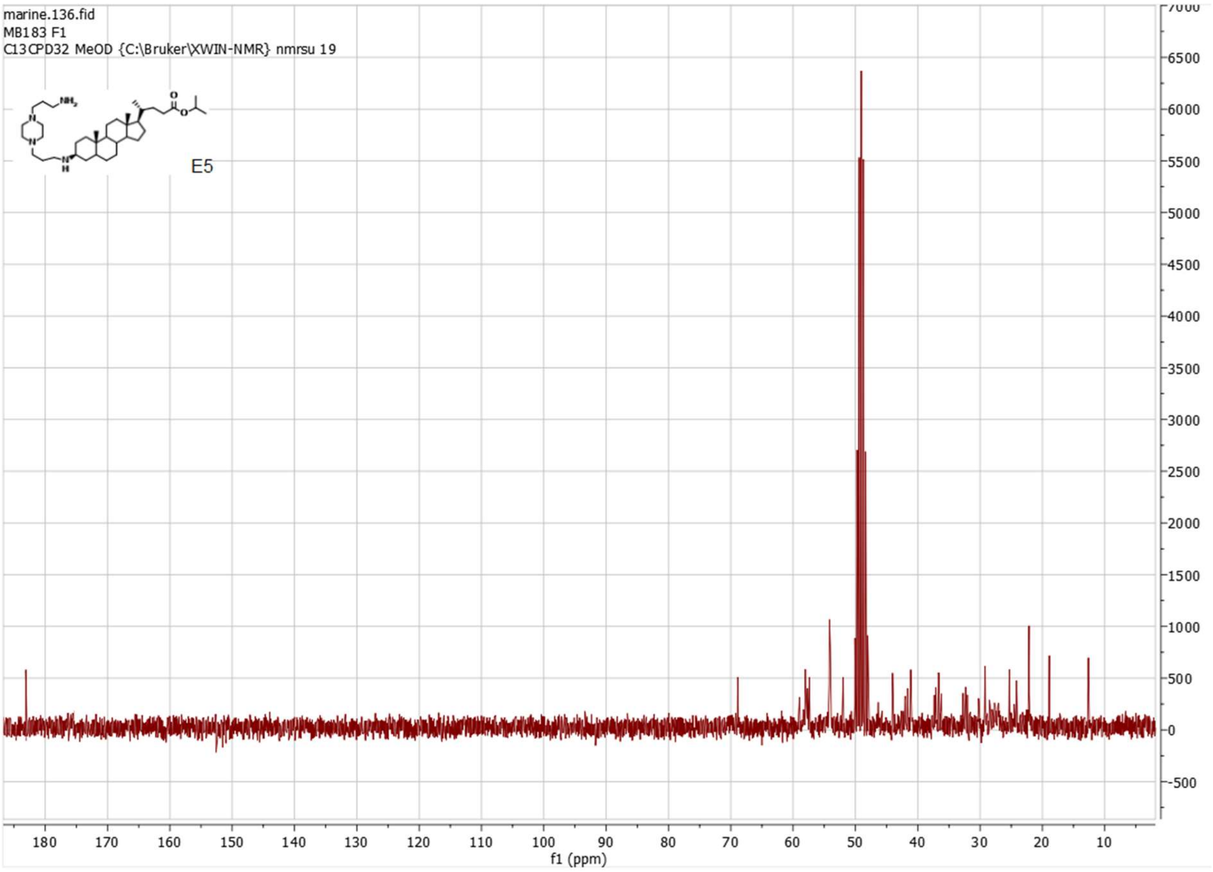
marine.136.fid
MB183 F1
C13CPD32 MeOD {C:\Bruker\XWIN-NMR} nmrsu 19
E5
f1 (ppm)

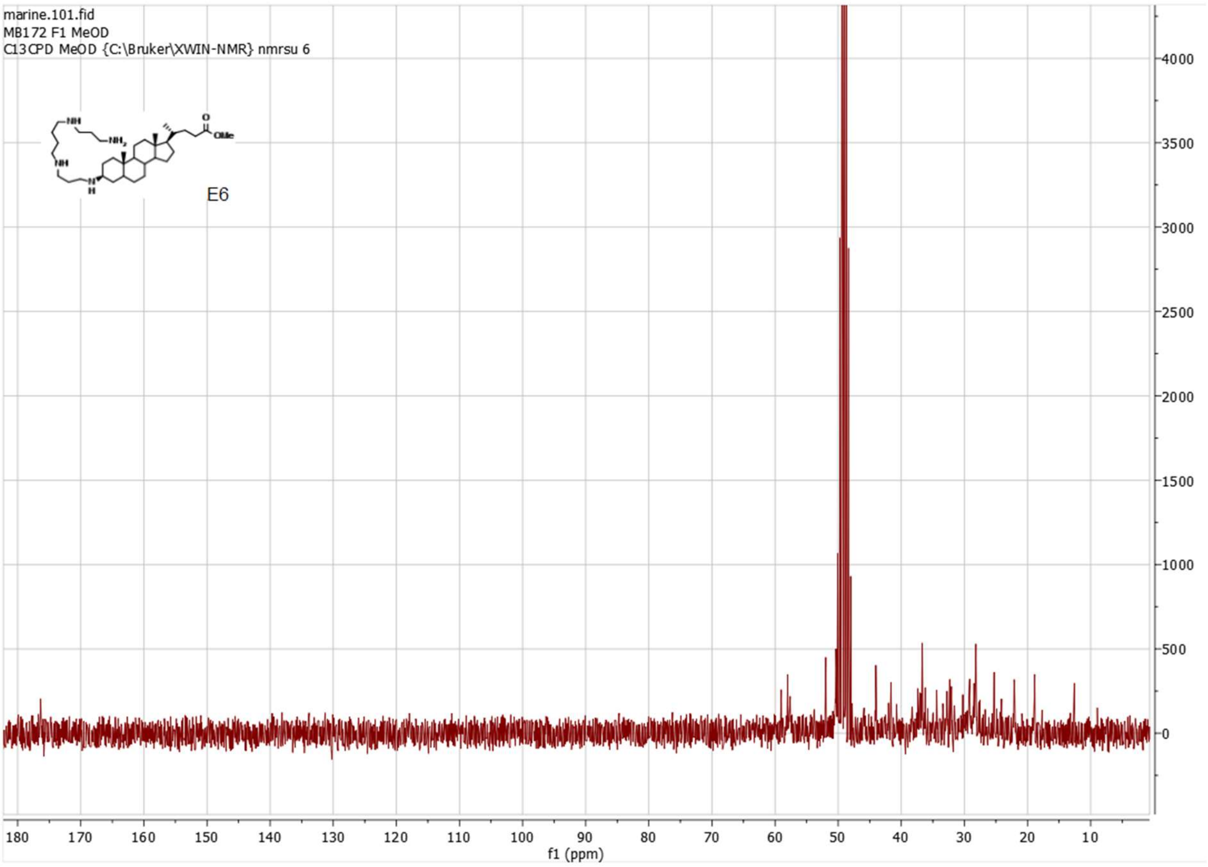
marine.101.fid
MB172 F1 MeOD
C13CPD MeOD {C:\Bruker\XWIN-NMR} nmrsu 6
E6
f1 (ppm)

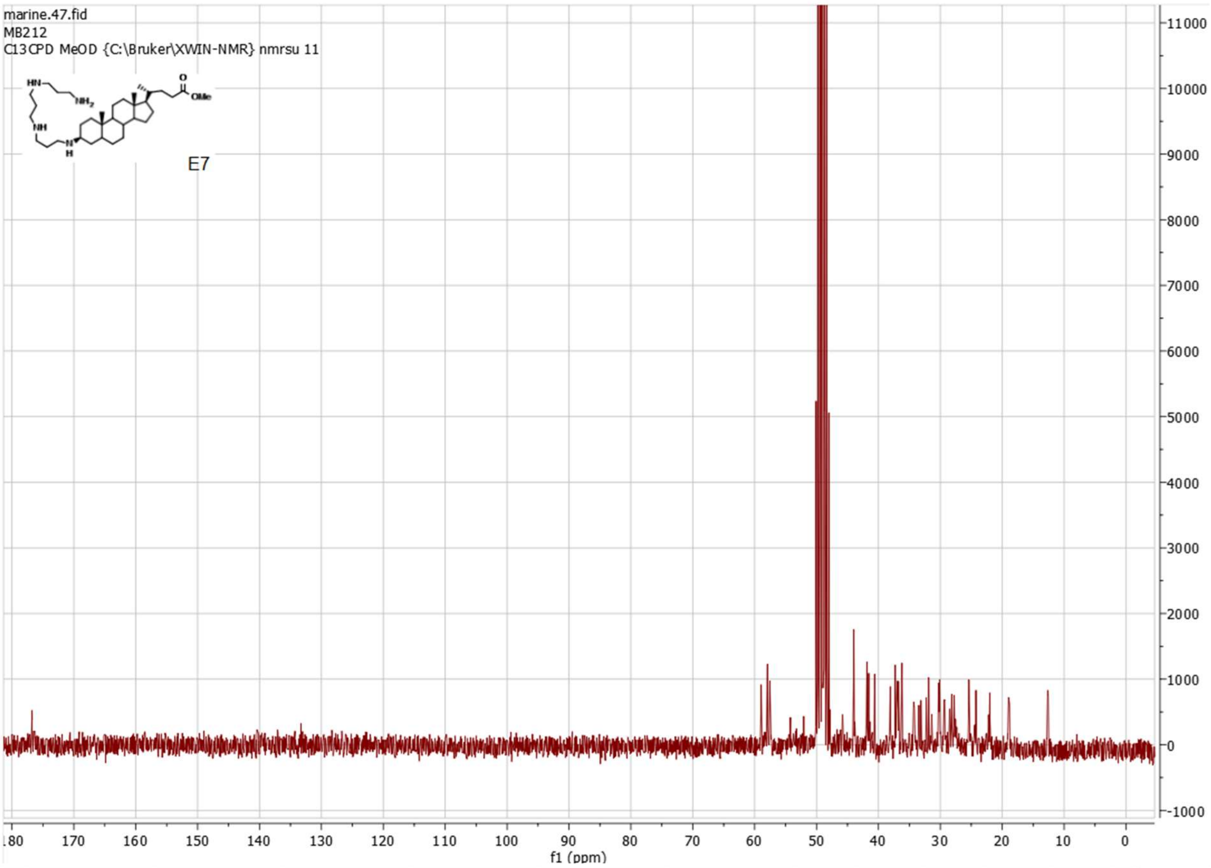
marine.47.fid
MB212
C13CPD MeOD {C:\Bruker\XWIN-NMR} nmrsu 11
E7
f1 (ppm)

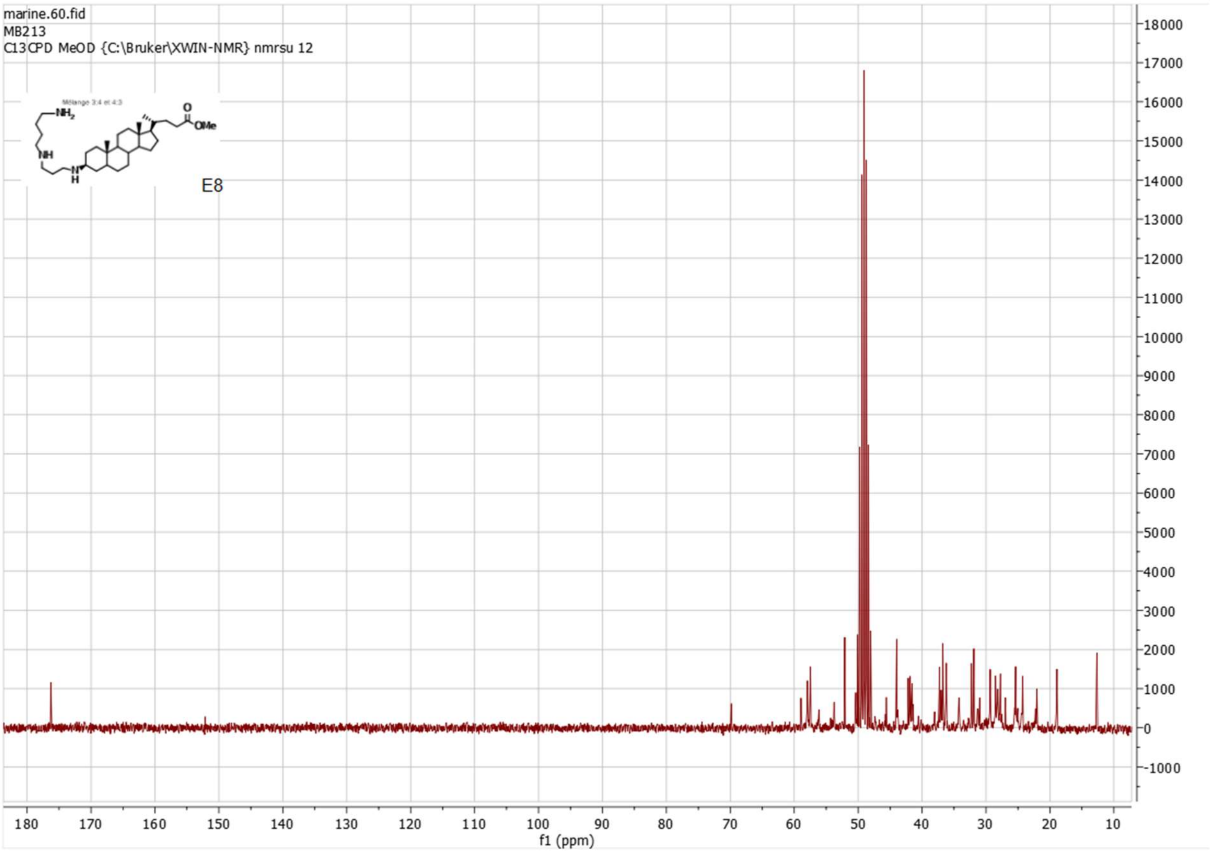
marine.60.fid
MB213
C13CPD MeOD {C:\Bruker\XWIN-NMR} nmrsu 12
E8
f1 (ppm)

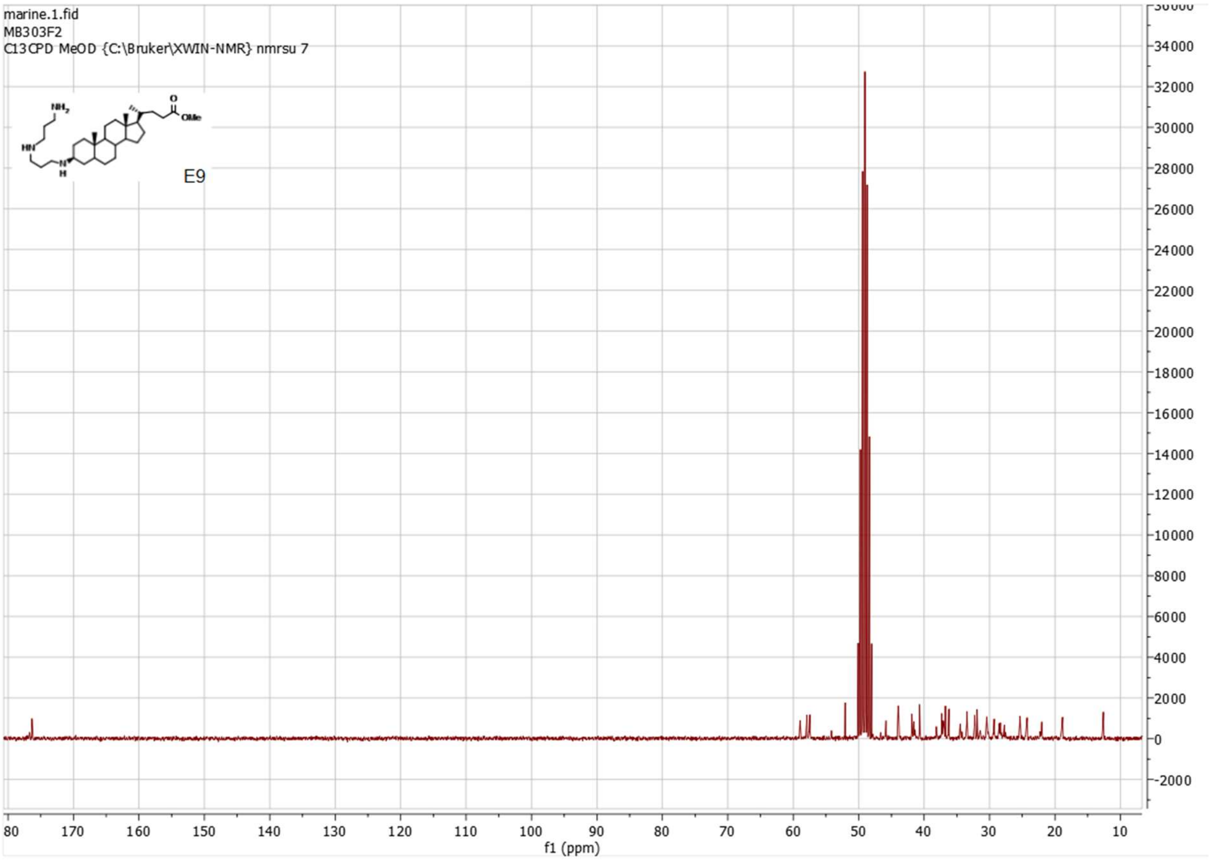
marine.1.fid
MB303F2
C13CPD MeOD {C:\Bruker\XWIN-NMR} nmrsu 7
E9
f1 (ppm)

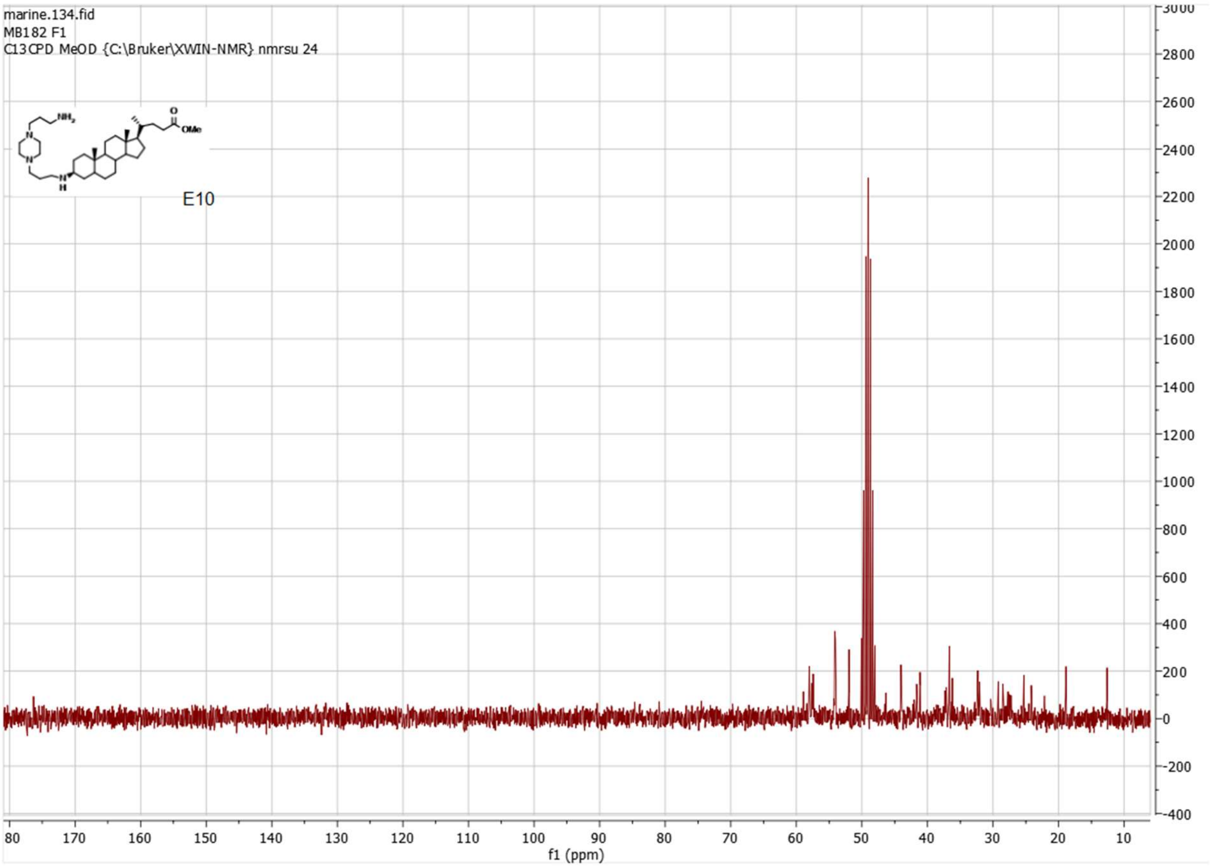
marine.134.fid
MB182 F1
C13CPD MeOD {C:\Bruker\XWIN-NMR} nmrsu 24
E10
f1 (ppm)

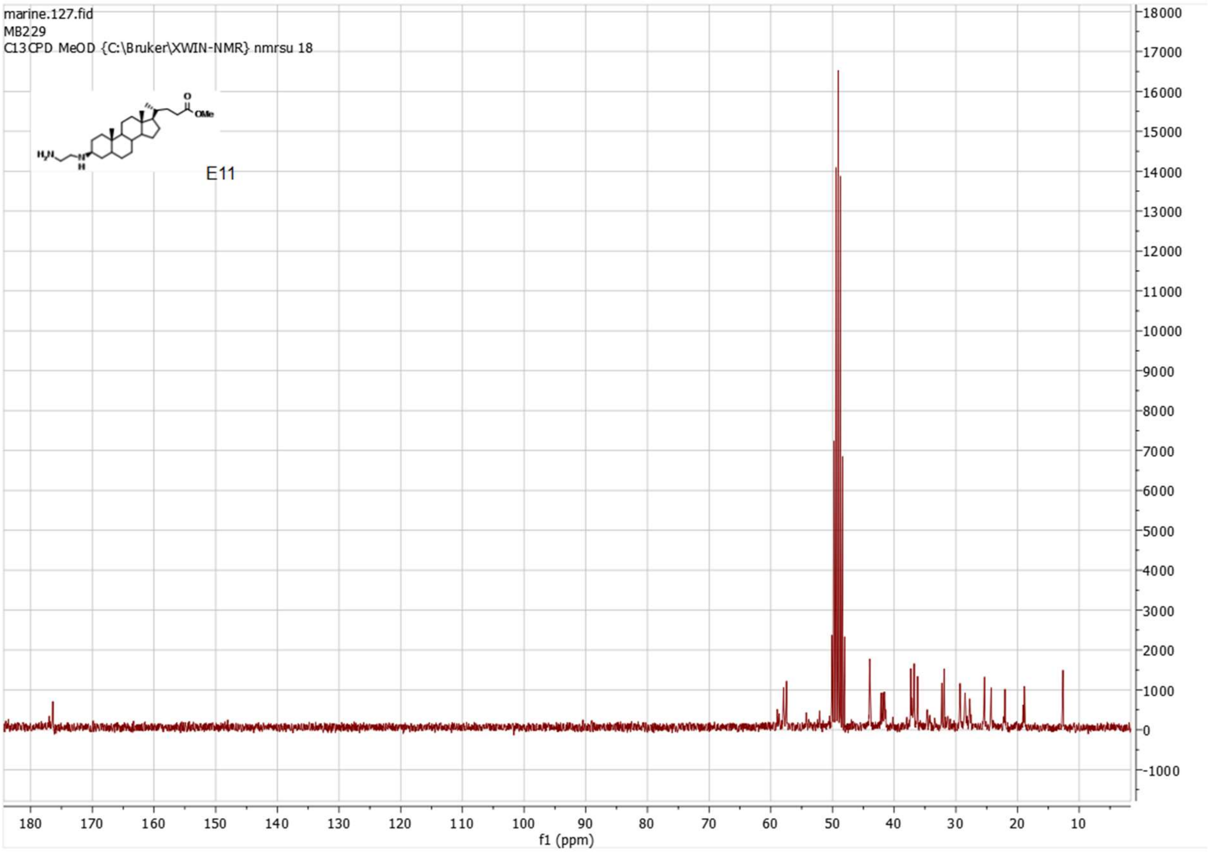

marine.127.fid
MB229
C13CPD MeOD {C:\Bruker\XWIN-NMR} nmrsu 18
OMe
E11
f1 (ppm)


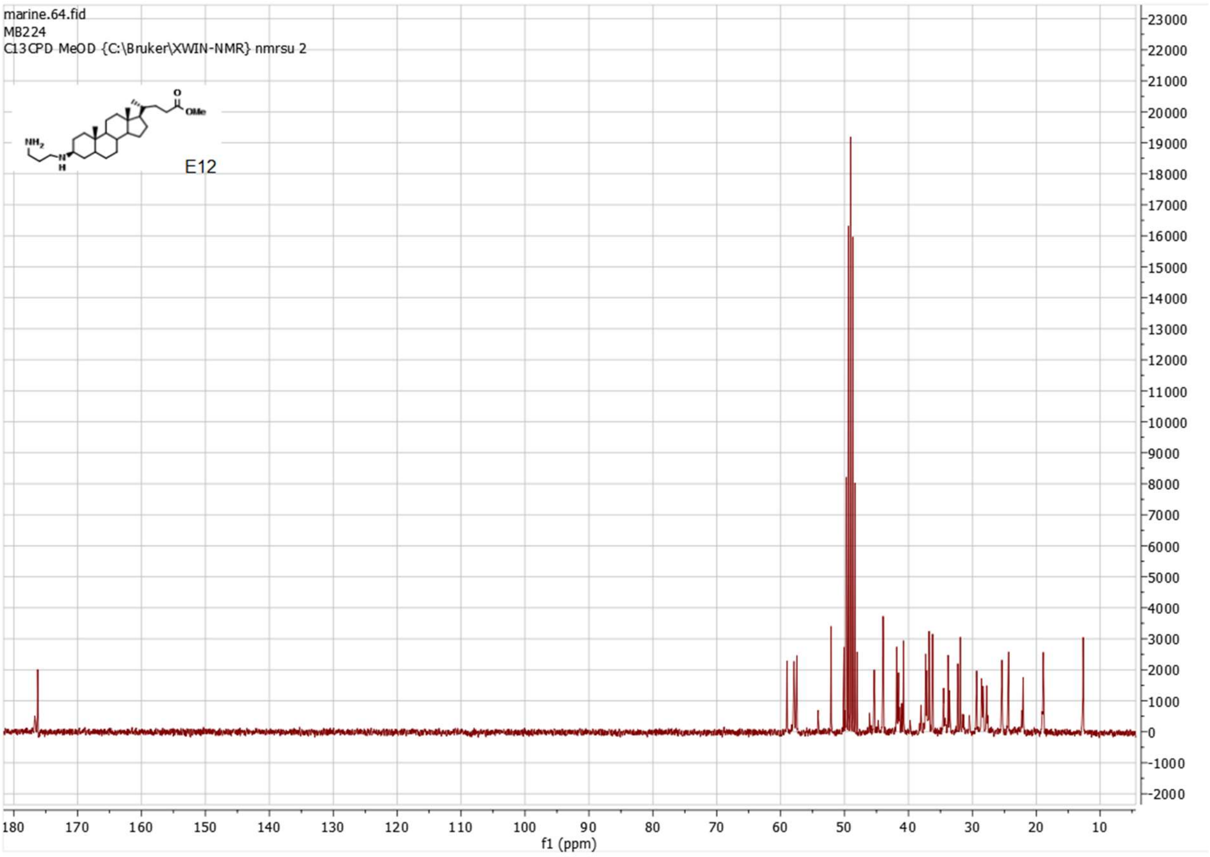

marine.64.fid
MB224
C13CPD MeOD {C:\Bruker\XWIN-NMR} nmrsu 2
OMe
E12
f1 (ppm)

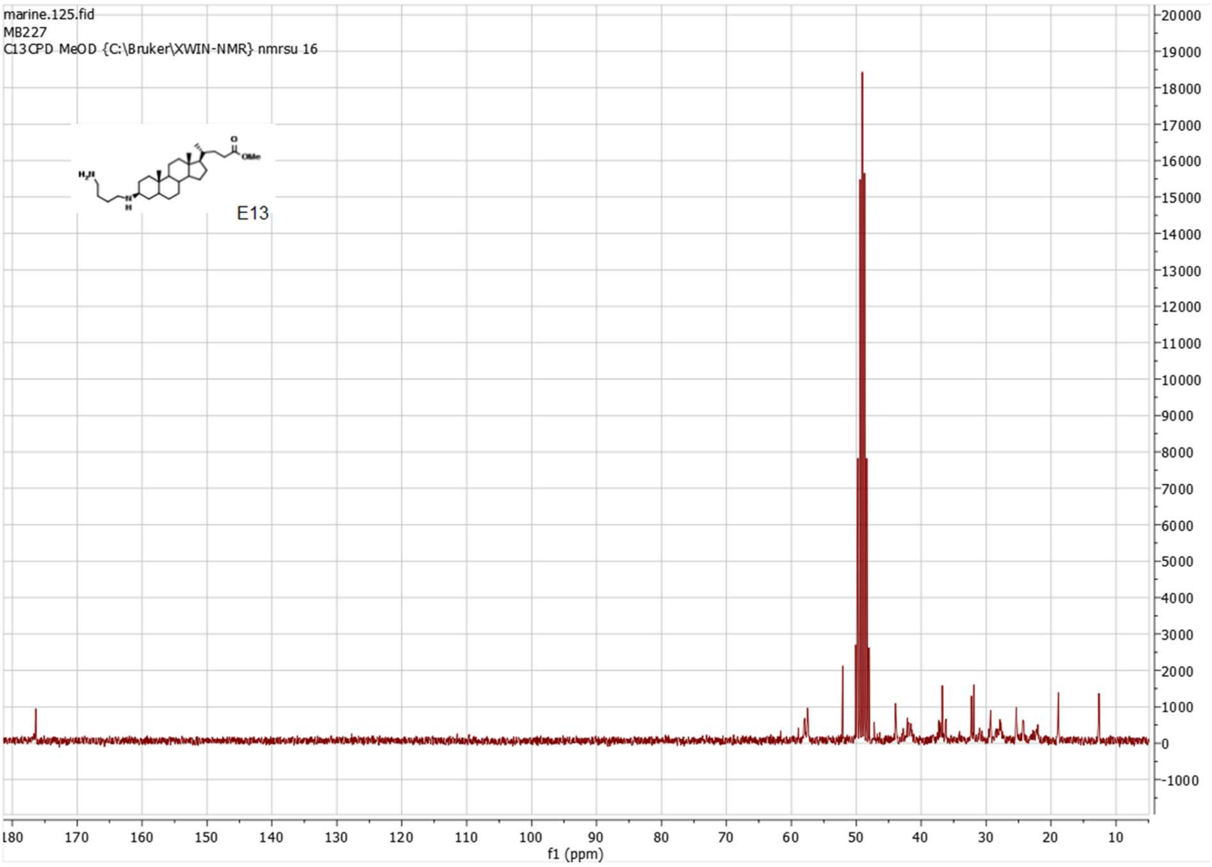
marine.125.fid
MB227
C13CPD MeOD {C:\Bruker\XWIN-NMR} nmrsu 16
E13
f1 (ppm)

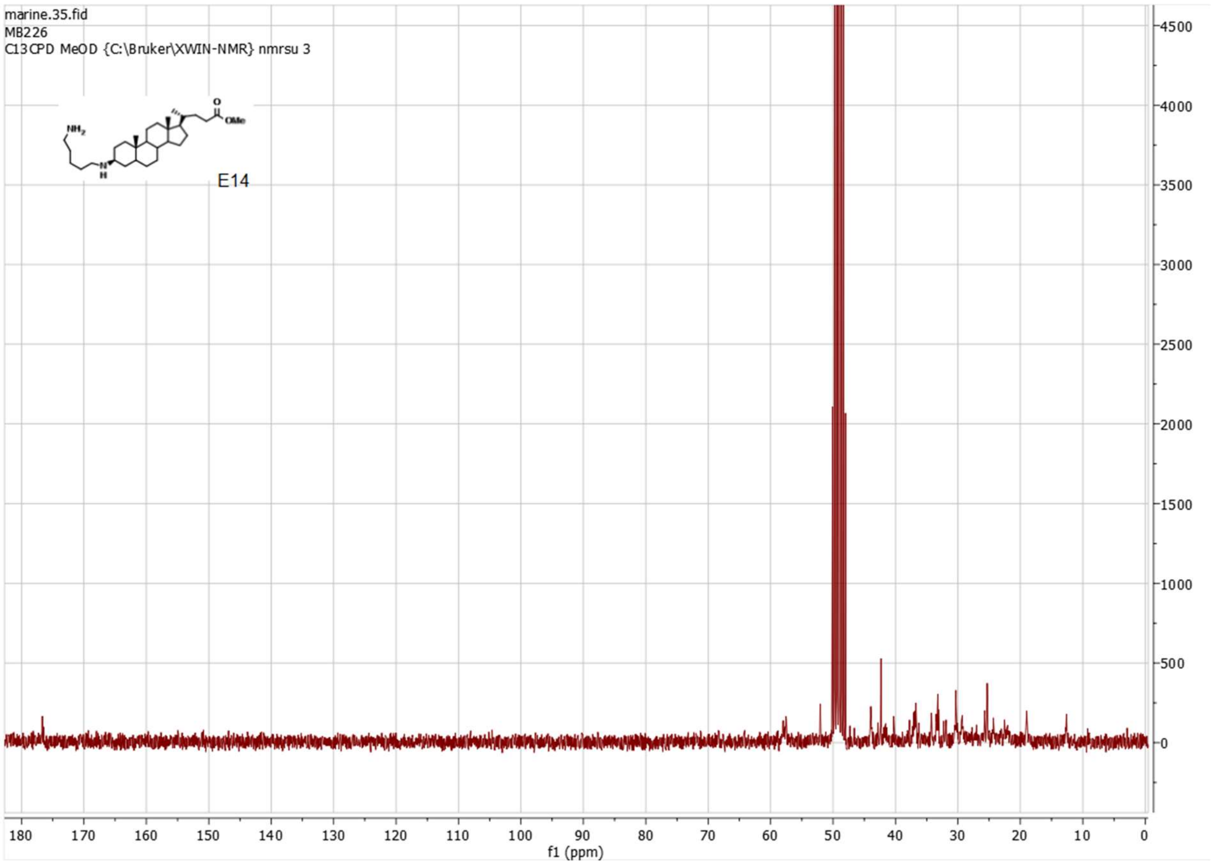
marine.35.fid
MB226
C13CPD MeOD {C:\Bruker\XWIN-NMR} nmrsu 3
E14
f1 (ppm)

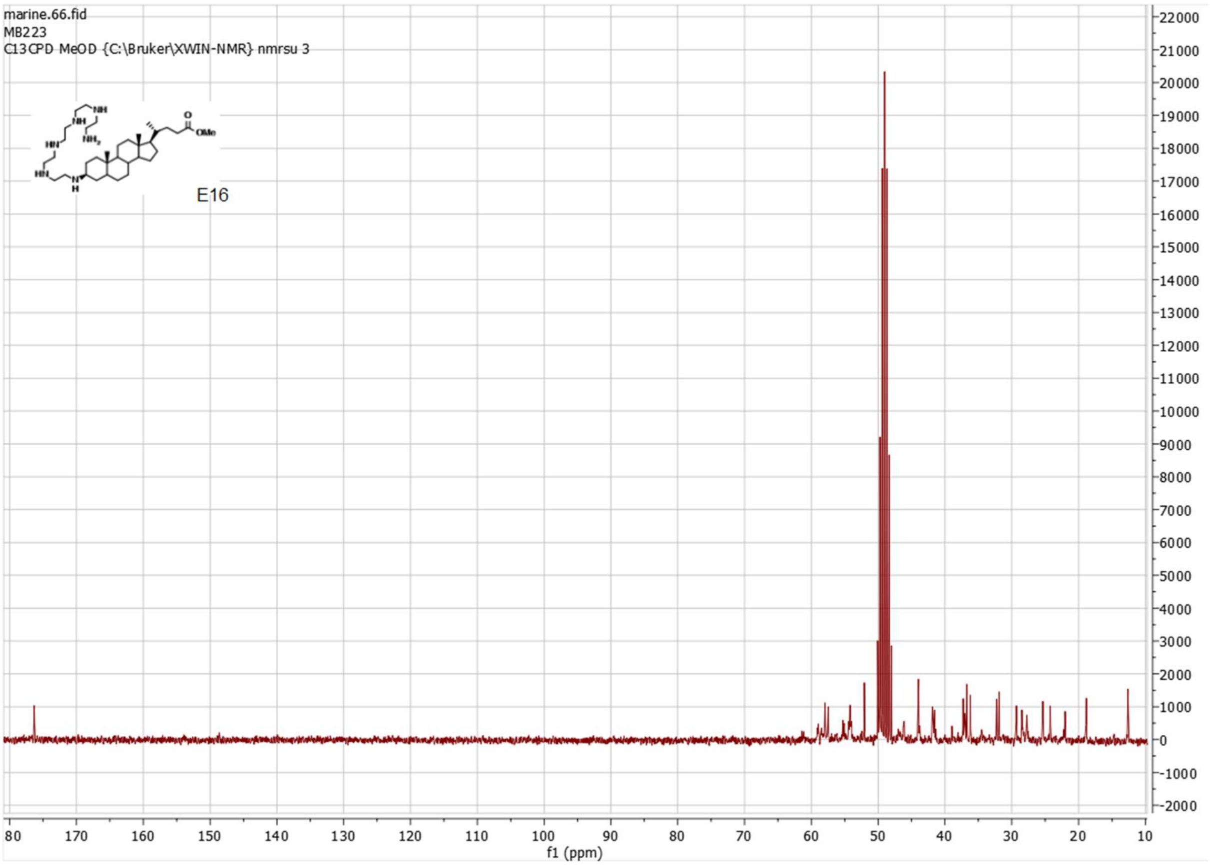
marine.66.fid
MB223
C13CPD MeOD {C:\Bruker\XWIN-NMR} nmrsu 3
E16
f1 (ppm)

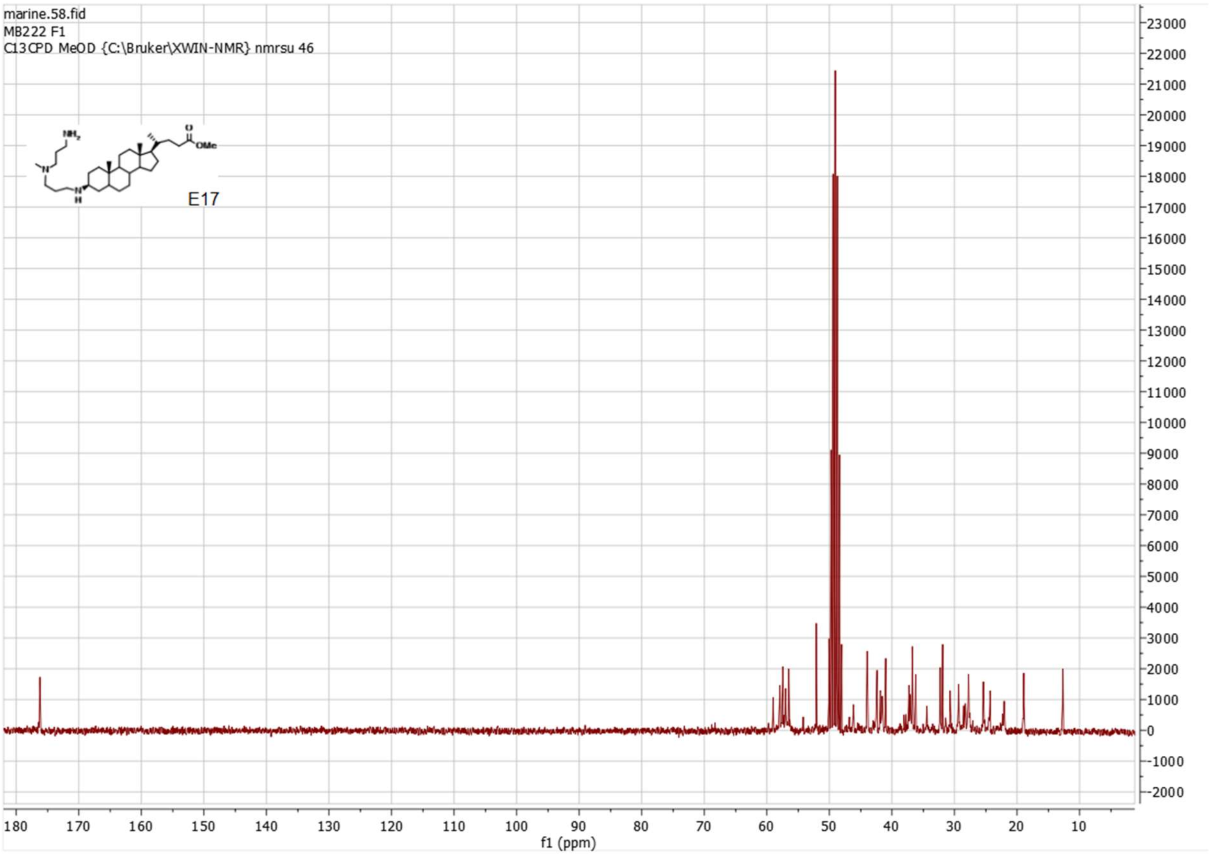
marine.58.fid
MB222 F1
C13CPD MeOD {C:\Bruker\XWIN-NMR} nmrsu 46
E17
f1 (ppm)

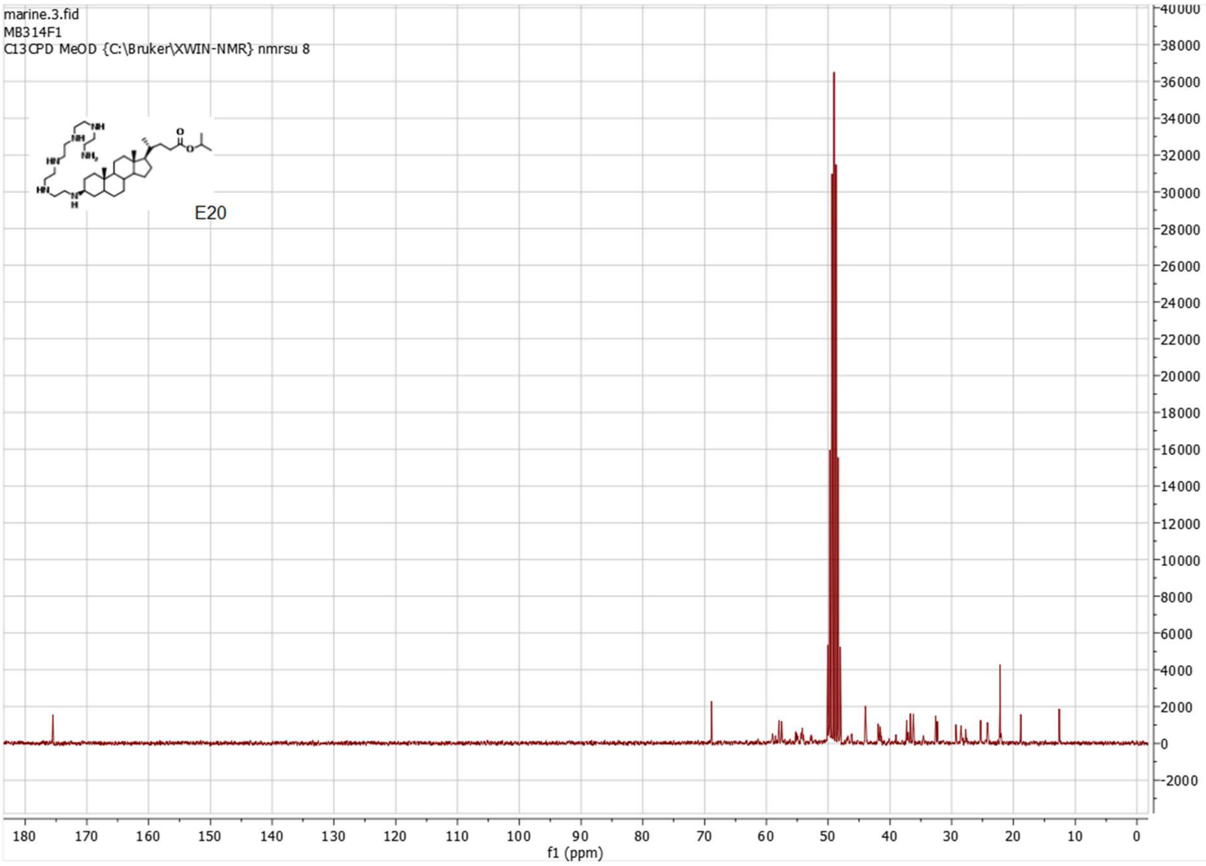
marine.3.fid
MB314F1
C13CPD MeOD {C:\Bruker\XWIN-NMR} nmrsu 8
E20
f1 (ppm)

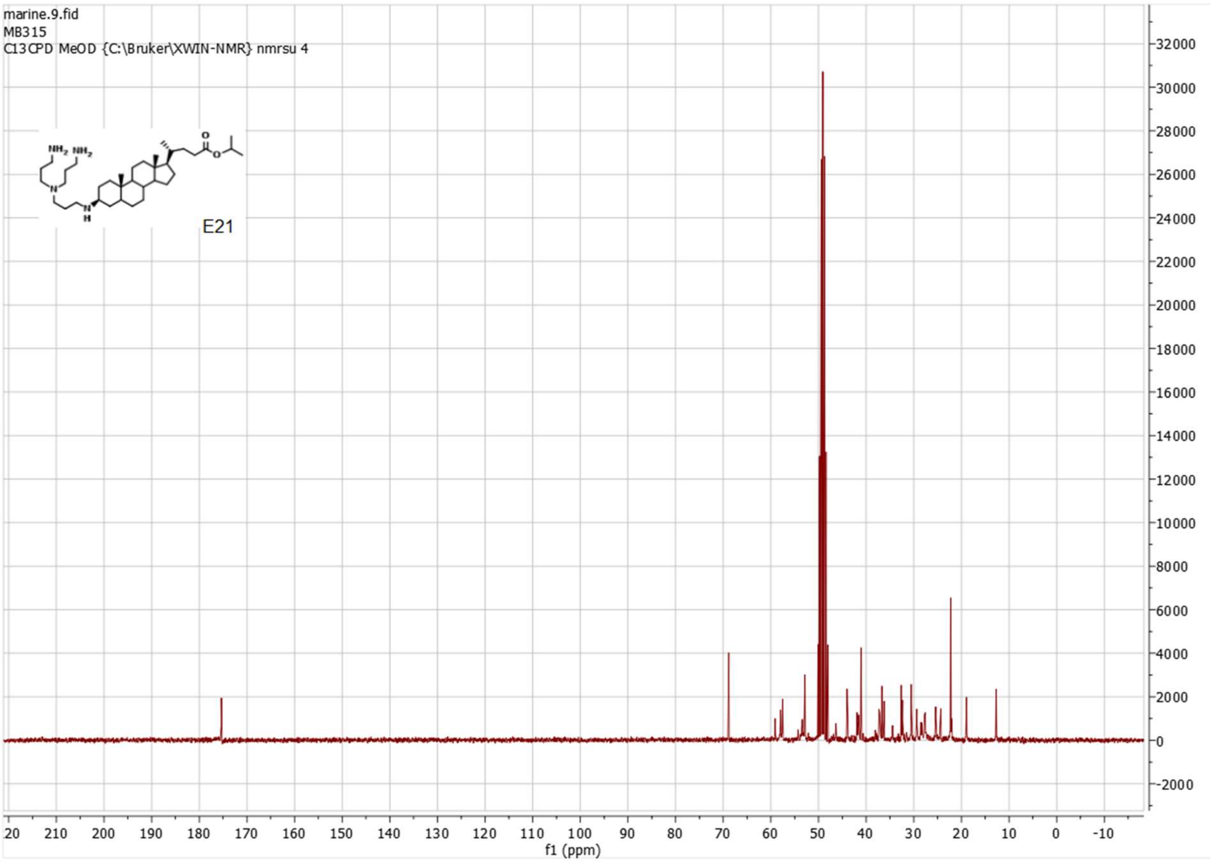
marine.9.fid
MB315
C13CPD MeOD {C:\Bruker\XWIN-NMR} nmrsu 4
E21
f1 (ppm)

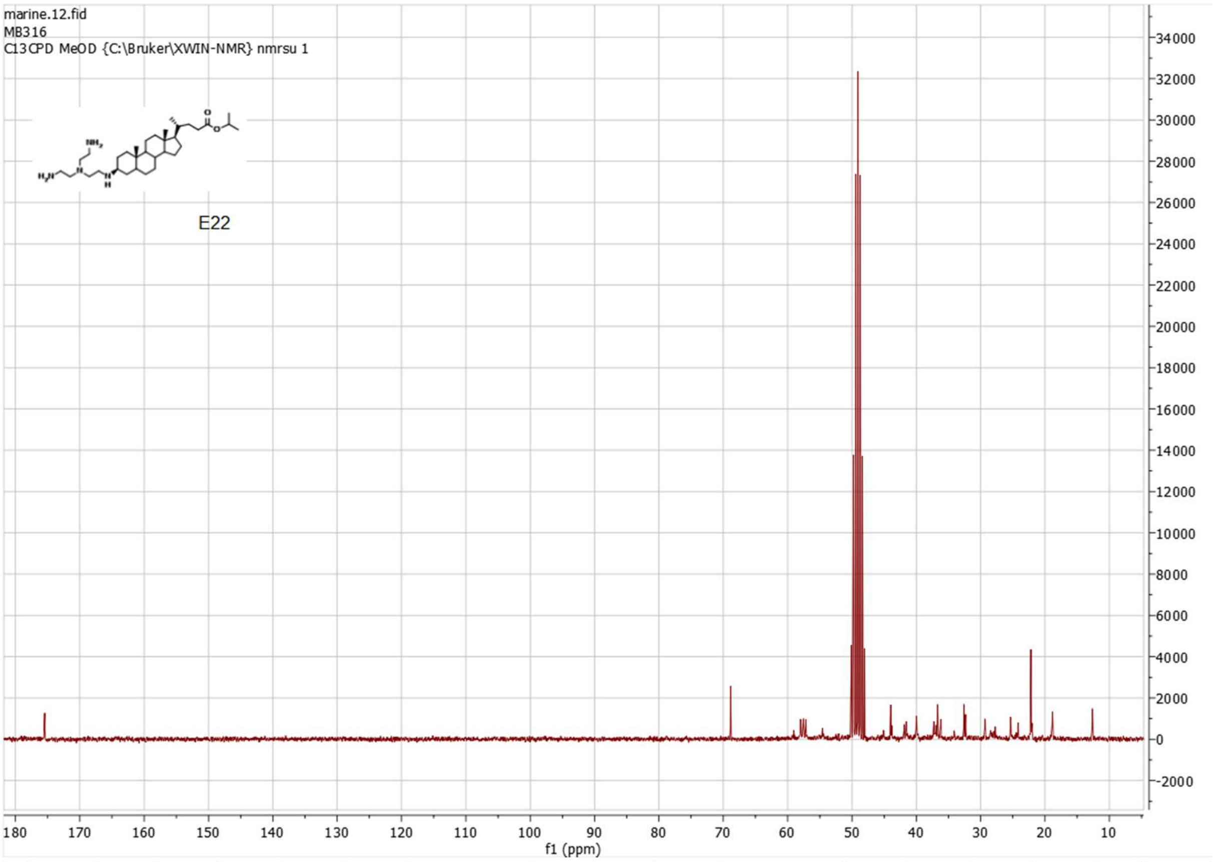
marine.12.fid
MB316
C13CPD MeOD {C:\Bruker\XWIN-NMR} nmrsu 1
E22
f1 (ppm)

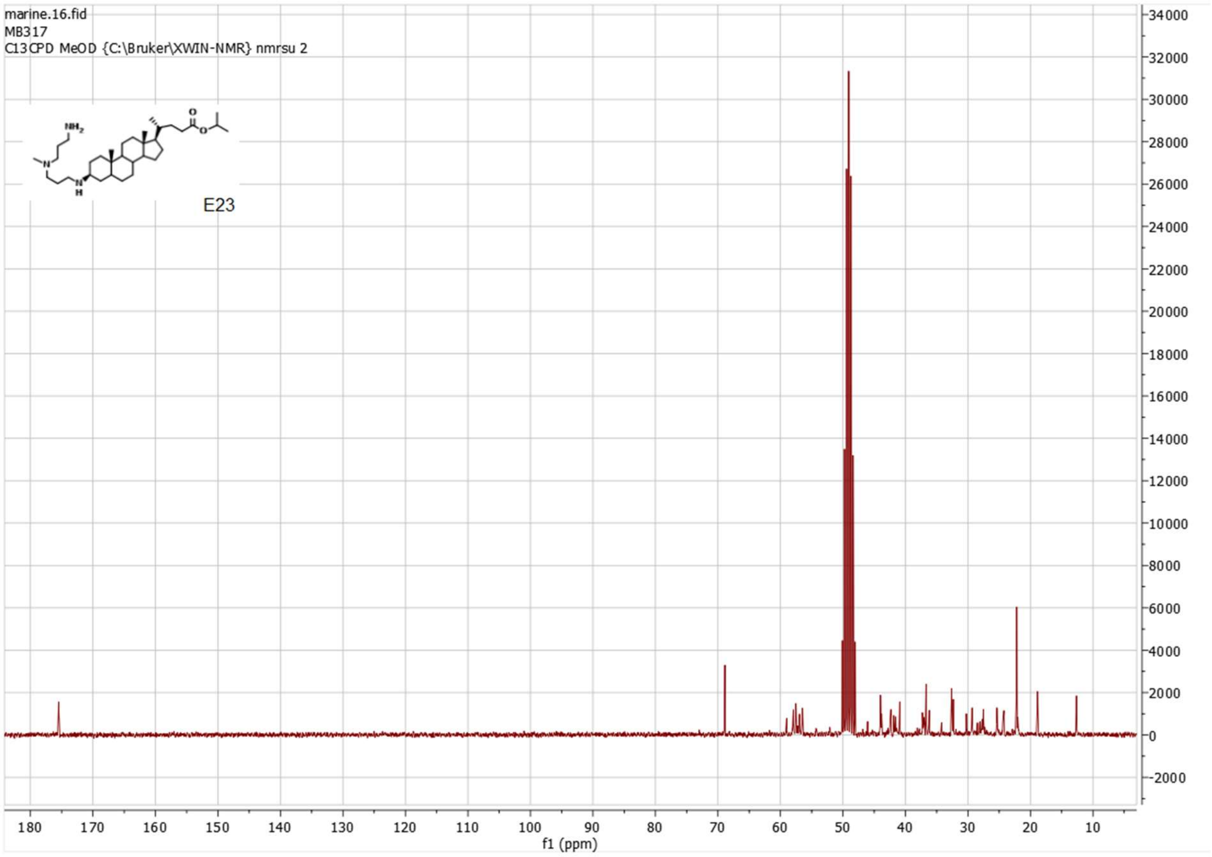
marine.16.fid
MB317
C13CPD MeOD {C:\Bruker\XWIN-NMR} nmrsu 2
E23
f1 (ppm)

**Table S1.** Summary of molecular descriptor groups calculated using the E-Dragon software

| Category | Descriptor group | Number of descriptors |
|---|---|---|
| Constitutional | Constitutional descriptors | 48 |
| | Functional group counts | 154 |
| | Ghose–Crippen descriptors | 120 |
| | Molecular properties | 29 |
| Topological | Topological descriptors | 119 |
| | Walk and path counts (molecular graph paths) | 47 |
| | Connectivity indices | 33 |
| | Information content indices | 47 |
| | 2D autocorrelations | 96 |
| | Edge adjacency indices (molecular adjacency matrix) | 107 |
| | Topological charge indices | 21 |
| | Eigenvalue-based indices (adjacency matrix) | 44 |
| Geometrical | Random walk molecular profiles | 41 |
| | Geometrical descriptors | 74 |
| | RDF descriptors (Radial Distribution Function) | 150 |
| | 3D-MoRSE descriptors (Molecular Electron Diffraction) | 160 |
| | WHIM descriptors (Weighted Holistic Invariant Molecular) | 99 |
| | GETAWAY descriptors (Geometry, Topology & Atom Weights) | 197 |
